# Total Syntheses of Scabrolide A and Nominal Scabrolide B

**DOI:** 10.1021/jacs.1c12401

**Published:** 2022-01-19

**Authors:** Zhanchao Meng, Alois Fürstner

**Affiliations:** Max-Planck-Institut für Kohlenforschung, 45470 Mülheim/Ruhr, Germany

## Abstract

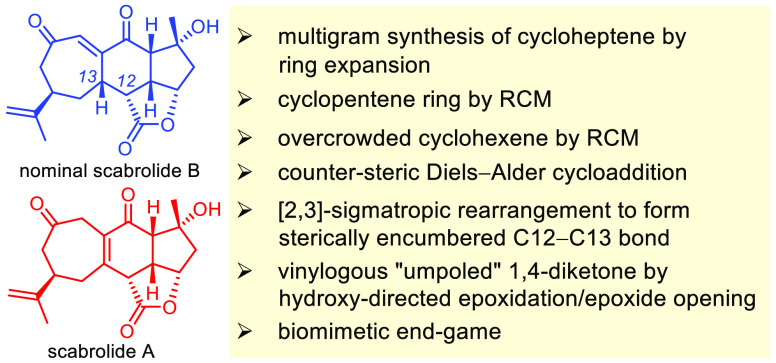

The marine natural product scabrolide A was obtained by isomerization of the vinylogous 1,4-diketone entity of nominal scabrolide B as the purported pivot point of the biosynthesis of these polycyclic norcembranoids. Despite the success of this maneuver, the latter compound itself turned out not to be identical with the natural product of that name. The key steps en route to the carbocyclic core of these targets were a [2,3]-sigmatropic rearrangement of an allylic sulfur ylide to forge the overcrowded C12–C13 bond, an RCM reaction to close the congested central six-membered ring, and a hydroxy-directed epoxidation/epoxide opening/isomerization sequence to set the “umpoled” 1,4-dicarbonyl motif and the correct angular configuration at C12.

Soft corals of the genus *Sinularia* produce a number of intriguing polycyclic norcembranoids of the yonarolide and scabrolide estate. These compounds are thought to derive from 5-episinuleptolide (**5**), which in turn descends from a furano-butenolide of type **4** (Scheme 1).^1−3^ Specifically, **5** is linked to nominal scabrolide B (**1**)^4^ as the purported key intermediate of the biosynthetic pathway by a sequence of transannular Michael addition and retro-oxa-Michael reactions.^1−3,5^ Subsequent isomerization of the vinylogous diketone of **1** into the presumably more stable tetrasubstituted enone gives rise to scabrolide A (**2**)^4,6,7^ and its dehydrated sibling yonarolide (**3**).^8−10^ In contrast to many other norcembranoids, the scabrolides exhibit only modest cytotoxicity; however, **2** was shown to inhibit IL-6 and IL-12 production *in vitro* and is hence of potential interest as an anti-inflammatory agent.^11^

**Scheme 1:**
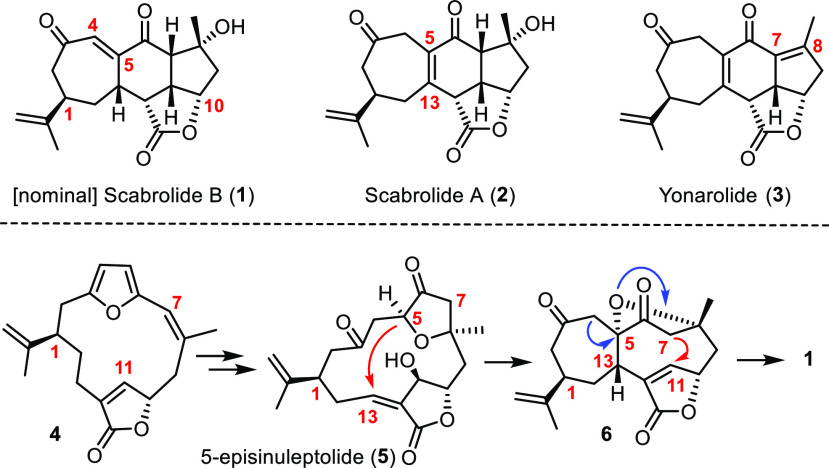
Structure and Proposed Biosynthesis of Tetracyclic Norcembranoids

For the architectural splendor of the caged tetracyclic backbone, the challenging oxygenation pattern comprising an “umpoled” 1,4-diketone motif, and the dense array of up to seven mostly contiguous chiral centers, these terpenoids represent formidable targets. Though known for (more than) two decades, it was only recently that a member of this family succumbed to total synthesis.^12−23^ Specifically, the Stoltz group reported an elegant approach to scabrolide A (**2**) based on late-stage formation of the seven-membered ring by a sequence of intramolecular enone/alkenylsilane [2 + 2] cycloaddition, followed by a mercury-mediated Tamao–Fleming type oxidation and a strain-driven oxidative fragmentation.^12^

As part of our program on marine natural products with unusual structures and bioactivities,^24−34^ including scarce metabolites isolated from *Sinularia* species,^35−37^ we pursued an entirely different approach in which the demanding cycloheptene was deliberately crafted at the outset. This tactical change is integral with a convergent strategy capitalizing on the pursuit of scabrolide B (**1**) as the primary target (Scheme 2). The priority of **1** follows from the biosynthetic logic which suggests that this compound can be rearranged into scabrolide A (**2**), whereas the inverse shift converting **2** into **1** is likely counter-thermodynamic. The vinylogous diketone of **1** should be unveiled by a late-stage oxidation of an olefin of type **A** to be formed by ring closing metathesis (RCM)^38^ of diene **B**. We surmised that a sigmatropic rearrangement, preferentially of the Claisen-type,^39,40^ might be suitable for the nontrivial assembly of this elaborate substrate in stereochemically correct format. Under this premise, the retrosynthetic analysis leads back to cycloheptene **D** and a bicyclic lactone of type **E**. Although some methodological amendment was necessary when reducing this blueprint to practice (see below), the underlying chemical reasoning ultimately proved successful, notably with regard to the final isomerization of **1** into **2**. In consideration thereof, it was all the more perplexing when we found that compound **1** itself as the presumed biosynthetic pivot point is actually not a (known) natural product;^41^ in any case, it definitely does not represent scabrolide B.

**Scheme 2:**
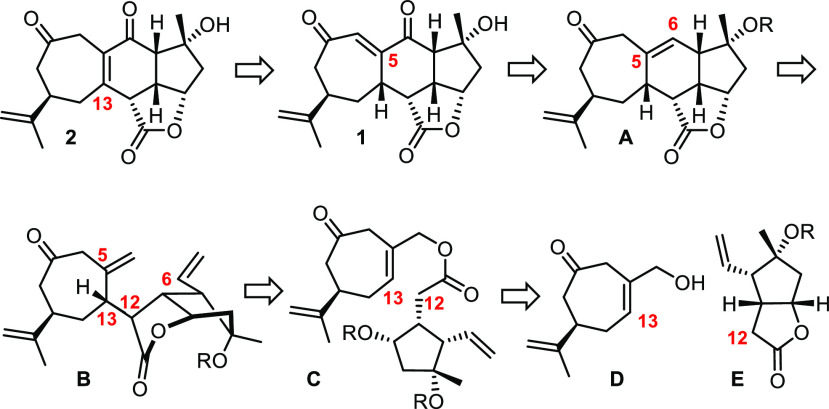
Retrosynthetic Analysis

(*R*)-Carvone (**7**) served as point of departure, which was transformed into **9** by the modification of a literature-known route (Scheme 3).^42^ To this end, the derived −OTMS cyanohydrin was reduced to the corresponding amine **8**, which underwent ring expansion upon diazotization; all of these steps were high yielding on multigram scale. The same is true for the subsequent stereoselective formation of epoxide **10**, which exclusively furnished the allylic alcohol **11** on treatment with bulky aluminum amide **13**.^43^ The derived crude mesylate, on exposure to aq. NaHCO_3_, cleanly rearranged into **12** as needed for the projected Claisen rearrangement.

**Scheme 3:**
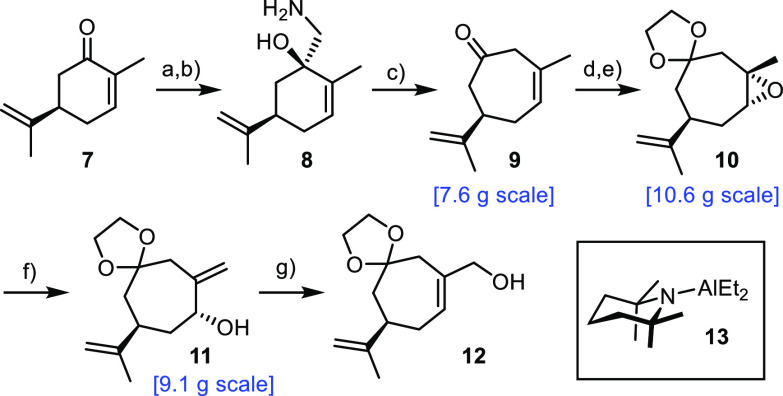
Reagents and conditions: (a) TMSCN, NMO, CH_2_Cl_2_; (b) LiAlH_4_, Et_2_O, 0 °C; (c) NaNO_2_, aq. HOAc, 0 °C, 71% (over three steps); (d) TMSOCH_2_CH_2_OTMS, TMSOTf (1 mol %), CH_2_Cl_2_, −78 °C → −20 °C; (e) *m*CPBA, CH_2_Cl_2_, −20 °C, 77% (over two steps); (f) *n*BuLi, 2,2,6,6-tetramethylpiperidine, Et_2_AlCl, toluene, 0 °C, quant.; (g) (i) MsCl, Et_3_N, CH_2_Cl_2_, 0 °C; (ii) aq. NaHCO_3_, 20 °C, 44%; the scales shown in this and the other Schemes refer to the single largest batch.

The required acid component derived from (*R*)-linalool (**14**), which was converted into cyclopentenone **15** by RCM, O-silylation, and subsequent ruthenium-catalyzed allylic oxidation (Scheme 4).^44^ We had taken note that related compounds had previously been shown to engage in unusual “counter-steric” Diels–Alder cycloadditions:^45^ indeed, reaction of **15** with excess butadiene furnished **16** as the only product after reduction of the ketone; its relative and absolute configuration were unambiguously established by X-ray diffraction (see the Supporting Information), which confirmed that the diene has added to the diastereotopic face of the activated dienophile shielded by the bulky −OTBS group. The dividend of this unusual outcome was harnessed in the subsequent oxidative cleavage of the double bond, which furnished a hemiketal that could be easily elaborated into lactone **18** as the stock form of the second key building block.^46^

**Scheme 4:**
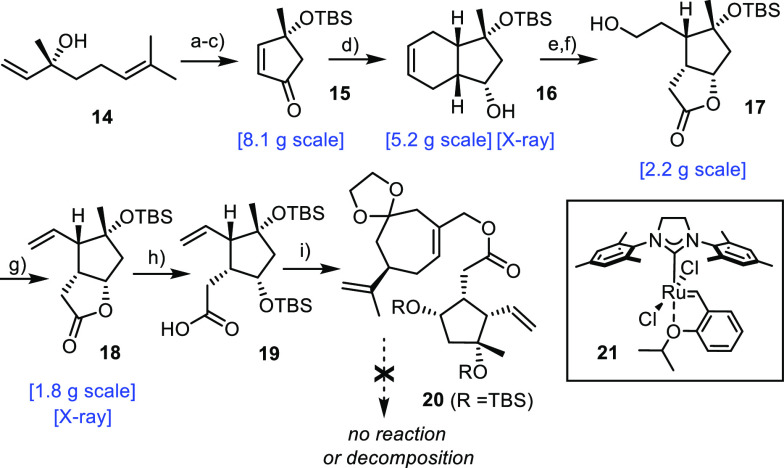
Reagents and conditions: (a) **21** (0.2 mol %); (b) NaH, TBSCl, THF, 65 °C; (c) RuCl_3_·H_2_O (1 mol %), Mg(OAc)_2_·4H_2_O, *t*BuOOH, 55% (over three steps); (d) (i) 1,3-butadiene, AlCl_3_, toluene; (ii) l-Selectride, THF, −78 °C, 69% (over two steps); (e) (i) O_3_, CH_2_Cl_2_; (ii) PPh_3_; (f) (i) PCC, CH_2_Cl_2_, 4 Å MS, 0 °C; (ii) NaBH_4_, 0 °C, 44% (over three steps); (g) 2-nitrophenylselenocyanate, *n*Bu_3_P, THF, 88%; (h) (i) NaOH, MeOH; (ii) TBSCl, DMF, imidazole; (i) **12**, DCC, Et_3_N, DMAP cat., CH_2_Cl_2_, 59% (over three steps); (j) KHMDS, Et_3_N, TMSCl, THF, −78 °C → 70 °C, or: LiHMDS, TMSCl, THF, −78 °C → 70 °C.

To test the envisaged fragment coupling, **18** was first hydrolyzed and the resulting hydroxy acid **19** instantly transformed into allylic ester **20**. Very much to our dismay, however, all attempts to subject this compound to an Ireland–Claisen rearrangement^39,40,47^ were met with failure, despite considerable experimentation. Confronted with this impasse at a critical point, various alternative ways were contemplated that might allow the two elaborate building blocks to be connected. In consideration of the stereochemical constraints to be met, a concerted process seemed most adequate; at the same time, the reaction must be rather insensitive to steric hindrance to allow the encumbered C12–C13 bond in question to be formed.^48^ In the end, we opted for a [2,3]-sigmatropic rearrangement of methyl sulfide **22** that is easily attained from **18** (Scheme 5).^49,50^ This compound does not carry much additional steric burden; actually, S-alkylation occurs away from the bulk and the resulting positive charge should facilitate the deprotonation of the C–H acidic site. The reactivity of the resulting sulfur ylide might provide the necessary driving force for the critical bond formation via a highly ordered transition state.

**Scheme 5:**
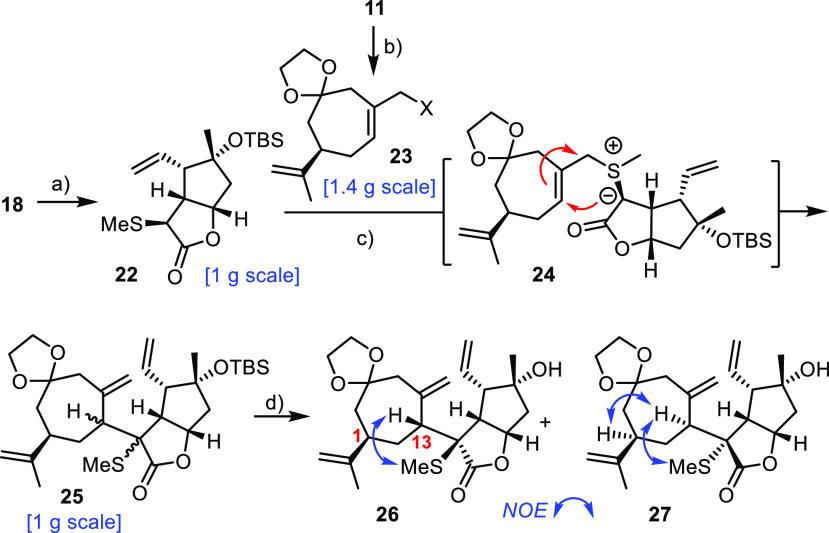
Reagents and conditions: (a) LiHMDS, MeSSO_2_Me, THF, −78 °C → −30 °C, 95%; (b) SOCl_2_, pyridine, Et_2_O, 0 °C, 89%; (c) (i) **23a** (X = Cl), NaI, acetone, reflux; (ii) AgBF_4_, 2,6-di-*tert*-butylpyridine, **22**, MeCN; (iii) *t*BuOK, MeCN; (d) TBAF, THF, reflux, **26** (31%) + **27** (33%) (over three steps)

To reduce this plan to practice, **11** was first converted into the corresponding allylic halides **23**.^51^ Direct S-alkylation of thioether **22** with bromide **23b** (X = Br) in DMF followed by treatment with aqueous K_2_CO_3_, as described in the literature, proved erratic.^52^ Therefore, we resorted to a procedure that had previously served our laboratory well:^53^ specifically, chloride **23a** (X = Cl) was converted into the corresponding iodide, which was then activated with AgBF_4_ in the presence of sulfide **22**; deprotonation of the resulting sulfonium salt with *t*BuOK entailed the envisaged [2,3]-sigmatropic rearrangement of the transient ylide **24** to give **25**; the crude material was desilylated to facilitate the separation of the two isomers. NOE data allowed the newly formed chiral centers C12 and C13 in **27** to be relayed to known C1; these assignments could be validated by crystallographic means. The remarkably long C12–C13 distance (1.596(2) Å) bears witness to the congestion about this central bond (Figure 1).^54^ An analogous NOE contact in **26** suggested that C12 and C13 are of opposite absolute configuration; this tentative conclusion was later confirmed by X-ray diffraction at the stage of the derived tetracyclic product **33** (see below).

**Figure 1:**
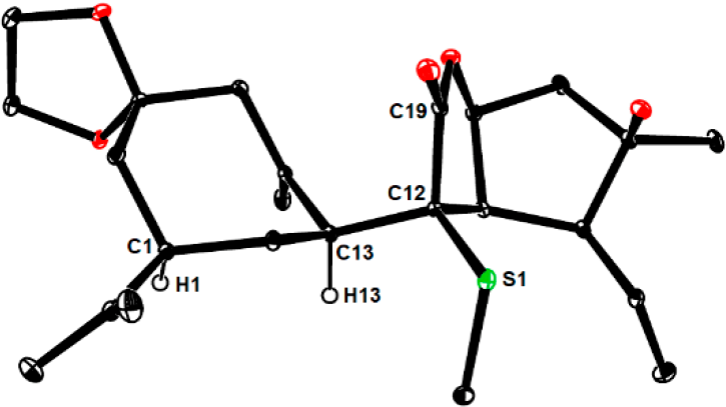
ORTEP representation of the structure of compound **27** in the solid state.

In line with ample literature precedent that divalent sulfur usually poisons ruthenium carbene catalysts,^38,55^ desulfurization of **26** had to precede closure of the six-membered ring by RCM. The very hindered nature of the C12–C13 bond formed by the [2,3]-sigmatropic rearrangement once again became apparent upon NMR inspection of the crude material formed on treatment of **26** with Bu_3_SnH/AIBN (Scheme 6):^56^ four isomeric compounds seemed to be present, suggestive of two diastereomers in two atropisomeric forms each. Gratifyingly, the composition was much simplified upon equilibration with DBU in MeCN, which allowed the desired product **28** to be obtained in respectable 79% yield. This diene could be cyclized with the aid of the ruthenium catalyst **21**,^57^ even though forcing conditions and hence a fairly high loading was necessary to achieve full conversion.^58,59^ The challenge associated with this transformation is further illustrated by the fact that the analogous diene 13-*epi*-**28** derived from **27** failed to afford the corresponding cyclohexene derivative.

**Scheme 6:**
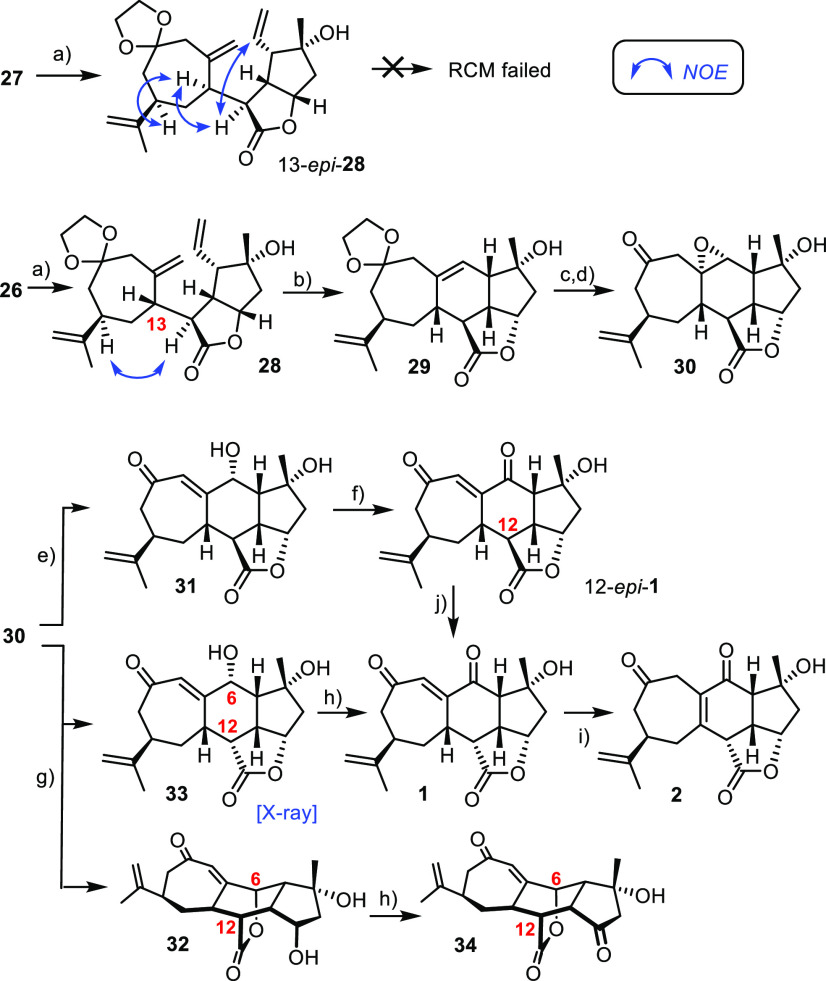
Reagents and conditions: (a) (i) Bu_3_SnH, AIBN, toluene, 85 °C; (ii) DBU, MeCN, reflux, 79% (**28**), 66% (13-*epi*-**28**); (b) **21** (10 mol %), toluene, 100 °C, 77%; (c) Montmorillonite K-10, CH_2_Cl_2_; (d) VO(acac)_2_ (10 mol %), *t*BuOOH, MS 4 Å, toluene, 0 °C → 20 °C; (e) Et_3_N, CH_2_Cl_2_, 55% (over three steps) (f) IBX, MeCN, 50 °C, 82%; (g) Et_3_N, MeOH; (h) IBX, MeCN, 50 °C, 34% (**1**) + 35% (**34**) (over four steps from **29**); (i) K_2_CO_3_, MeCN, 40 °C, 98%; (j) DBU, 0 °C, quant.

Cleavage of the ketal in **29** set the stage for a hydroxy-directed epoxidation of the trisubstituted alkene with *t*BuOOH catalyzed by VO(acac)_2_.^60,61^ For stability reasons, it was best to elaborate the resulting product further without delay: when treated with Et_3_N in CH_2_Cl_2_, the epoxide was opened and the vinylogous oxygenation pattern was unveiled, but the C12-stereocenter α to the lactone remained unchanged. Oxidation of compound **31** thus formed with IBX furnished 12-*epi*-**1**. In striking contrast, the use of Et_3_N/MeOH entailed epoxide opening as well as concomitant epimerization: given the pentacyclic skeleton of **30**, the ease of this transformation is deemed remarkable (20 °C, 30 min). The stereochemical outcome follows from the curvature of the compound: reprotonation of a transient lactone enolate derived from **30** will occur from the top face to give **33** (Figure 2), since all neighboring substituents on the central six-membered ring are downward-oriented. The disadvantage, however, was competing translactonization with formation of **32**, which could not be suppressed.^21^ For the sake of convenience, this product mixture was subjected to the final oxidation because the resulting nominal scabrolide B (**1**)^62^ could be readily separated from the isomeric compound **34**. Importantly, the issue of translactonization can be circumvented altogether by passing through 12-*epi*-**1**: on treatment with either Et_3_N in MeOH or preferentially DBU, clean inversion of the C12-stereocenter was observed and product **1** was reached without incident.

**Figure 2:**
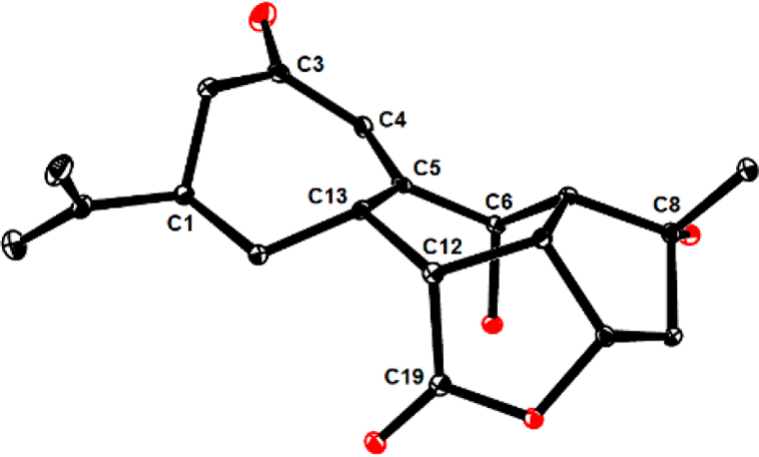
ORTEP representation of the structure of compound **33** in the solid state.

We were surprised by the striking mismatch of the spectral data of synthetic **1** and authentic scabrolide B (Figure 3).^4^ Although a detailed analysis of the recorded spectra left no doubt about the constitution and configuration of our compound, additional confirmation was sought to avoid any ambiguity. In the end, we managed to obtain single crystals of the precursor alcohol **33** suitable for X-ray diffraction analysis (Figure 2): as expected, all H atoms on the central cyclohexanone ring reside on the same face. This finding, in turn, confirms the configuration assigned to C13 set by the sigmatropic rearrangement and proves that the C12 center was epimerized during the Et_3_N/MeOH treatment.

**Figure 3:**
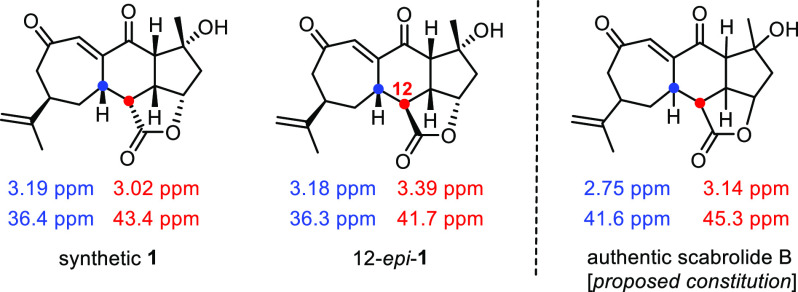
Selected NMR data showing the mismatch between the synthetic samples of nominal scabrolide B (and 12-*epi*-**1**) and the isolated natural product; for the full data sets, see the Supporting Information.

Equally significant is the fact that synthetic **1**, on exposure to K_2_CO_3_ in MeCN, rearranged quantitatively to scabrolide A (**2**); the spectra of our sample were in full accord with those of the natural product reported in the literature.^4^ We can hence confirm that scabrolide A (**2**) is the thermodynamic product and the assigned structure is correct, as had already been shown by the Stoltz group.^12^ Moreover, the ease of isomerization of compound **1** into **2** lends credence to the proposed biosynthesis;^2,3^ at the same time, however, it reduces the chance to extract compound **1** from a *Sinularia* species in a future isolation campaign as a proper natural product, even though this possibility does exist.^41^ The question as to the correct structure of scabrolide B, however, which differs from synthetic **1** as well as from the C12-epimer 12-*epi*-**1**, has to remain open at this point; all chiral centers and even the constitution of the compound isolated from the natural source need to be carefully reassessed.

## Supporting Information Available

Experimental section including supporting crystallographic information, characterization data, and NMR spectra of new compounds (PDF)

## Accession Codes

CCDC 2121819–2121821 and 2132679 contain the supplementary crystallographic data for this paper. These data can be obtained free of charge via www.ccdc.cam.ac.uk/data_request/cif, or by emailing data_request@ccdc.cam.ac.uk, or by contacting The Cambridge Crystallographic Data Centre, 12 Union Road, Cambridge CB2 1EZ, UK; fax: +44 1223 336033.
