# Supplementary material for: Total Syntheses of Scabrolide A and Nominal Scabrolide B
Source: J Am Chem Soc. 2022 Jan 19;144(4):1528–33. doi: 10.1021/jacs.1c12401 (PMC8815080; doi:10.1021/jacs.1c12401)
Supplement: Supplementary file 1 — ja1c12401_si_001.pdf [file ja1c12401_si_001.pdf]

Supporting Information

Total Syntheses of Scabrolide A and Nominal Scabrolide B

Zhanchao Meng, and Alois Fürstner*

Max-Planck-Institut für Kohlenforschung, 45470 Mülheim/Ruhr, Germany

Email: fuerstner@kofo.mpg.de

Table of Contents

Supporting Crystallographic Information	S2
General	S7
Experimental Details and Characterization Data. General.	S8
Tables	S25
Comparison of the Spectra of Synthetic and Authentic Scabrolide A	S29
Copies of NMR Spectra of New Compounds	S31
References	S114

Supporting Crystallographic Information

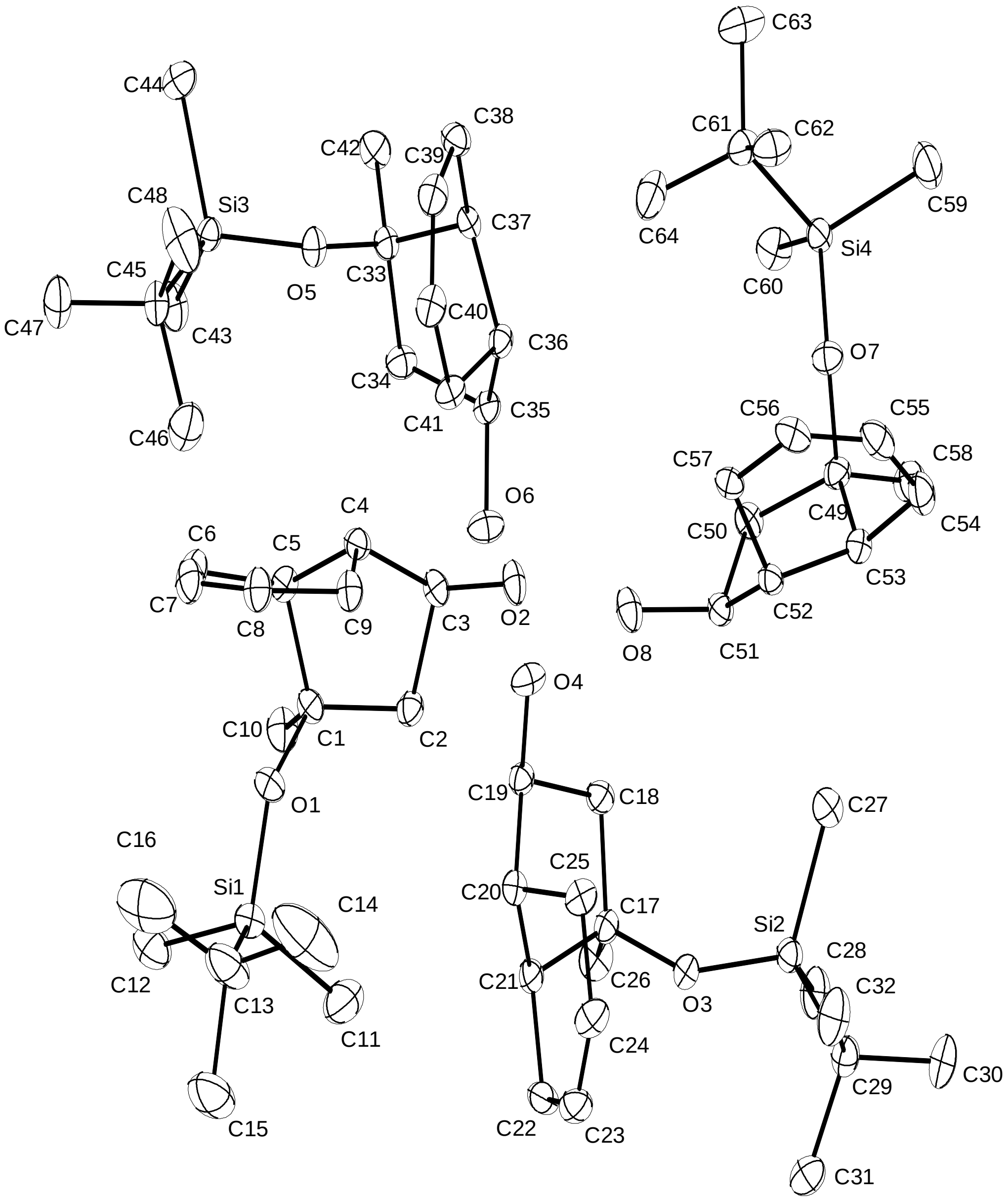

Figure S1. Molecular structure of the four independent molecules of compound **16** in the solid state; atomic displacement ellipsoids are shown at the 50% probability level, H-atoms omitted for clarity.

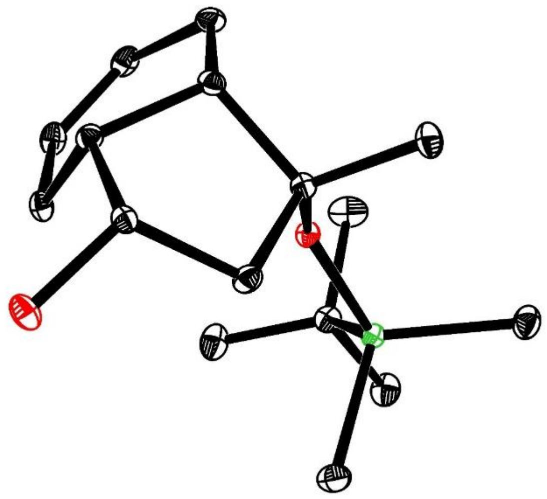

Figure S2. Molecular structure of one of the four independent molecules of compound **16** in the solid state, which shows the relative and absolute configuration of this product; atomic displacement ellipsoids are shown at the 50% probability level, H-atoms omitted for clarity.

Crystal Data for Compound 16: $C_{16}H_{30}O_2Si$, M = 282.49 g · mol^{-1}, colorless plates, crystal size 0.28 x 0.065 x 0.03 mm, monoclinic $P2_1$ (no. 4), T = 100(2) K, a = 8.277(2) Å, b = 29.148(9) Å, c = 14.223(3) Å , β = 94.02(2)°, V = 3422.8(16) Å^3, Z = 8, ρ = 1.096 Mg · m^{-3}, $\mu(Mo\text{-}K_\alpha)$ = 0.135 mm^{-1}, λ = 0.71073 Å, Gaussian absorption correction (T_{min} = 0.98, T_{max} = 1.00), Bruker AXS Enraf-Nonius KappaCCD with a FR591 rotating Mo-anode X-ray source, 2.766 < θ < 28.281°, 49159 measured reflections, 16893 independent reflections, 14093 reflections with $I > 2\sigma(I)$, R_{int} = 0.045, absolute structure parameter = –0.01(4), 713 parameters, residual electron density +0.3 (1.37 Å from Si1) / –0.3 (0.62 Å from Si1) e · Å^{-3}.

The structure was solved by *SHELXT* and refined by full-matrix least-squares (*SHELXL*) against F^2 to R_1 = 0.047 [$I > 2\sigma(I)$], wR_2 = 0.096. **CCDC-2121820.**

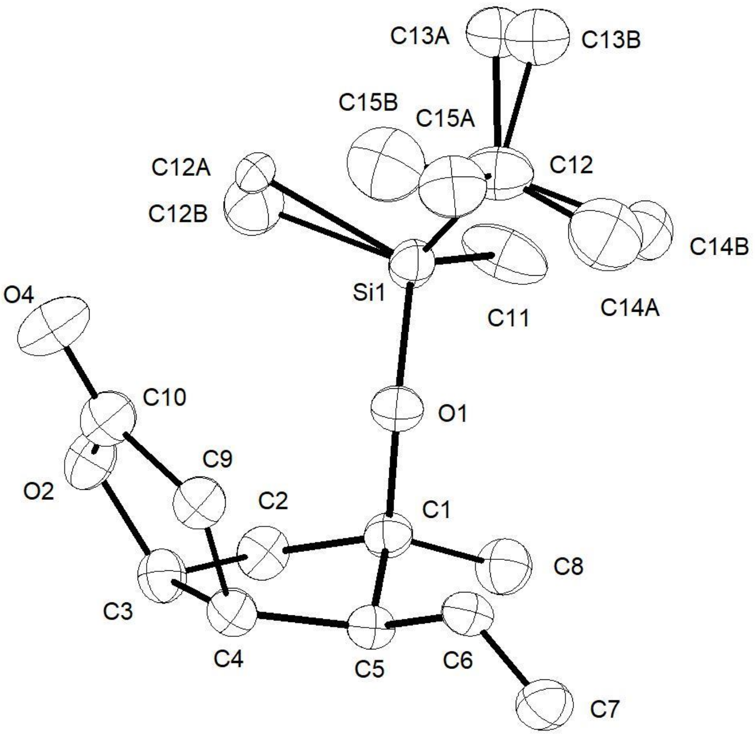

Figure S3. Molecular structure of **18** in the solid state; atomic displacement ellipsoids are shown at the 50% probability level, H-atoms omitted for clarity.

Crystal Data for Compound 18: $C_{16} H_{28} O_3 Si$, M = 296.47 g · mol^{-1}, colorless prisms, crystal size 0.282 x 0.130 x 0.120 mm, tetragonal, $P4_1$ (no. 76), T = 100(2) K, a = 10.3297(2) Å, b = 10.3297(2) Å, c = 16.2988(6) Å, V = 1739.13(9) Å^3, Z = 4, ρ = 1.132 Mgm^{-3}, μ(Cu-K$_\alpha$) = 1.228 mm^{-1}, λ = 1.54178 Å, Gaussian absorption correction (T_{min} = 0.98, T_{max} = 1.00), Bruker AXS Enraf-Nonius KappaCCD with a FR591 rotating Mo-anode X-ray source, 5.069 < θ < 62.817°, 57629 measured reflections, 2652 independent reflections, 2618 reflections with $I > 2\sigma(I)$, R_{int} = 0.0379, absolute structure parameter = 0.022(9), 229 parameters, residual electron density +0.2 (0.60 Å from C12) / –0.2 (0.53 Å from Si1) e · Å^{-3}.

The structure was solved by *SHELXT* and refined by full-matrix least-squares (*SHELXL*) against F^2 to R_1 = 0.030 [$I > 2\sigma(I)$], wR_2 = 0.075. **CCDC-2121821.**

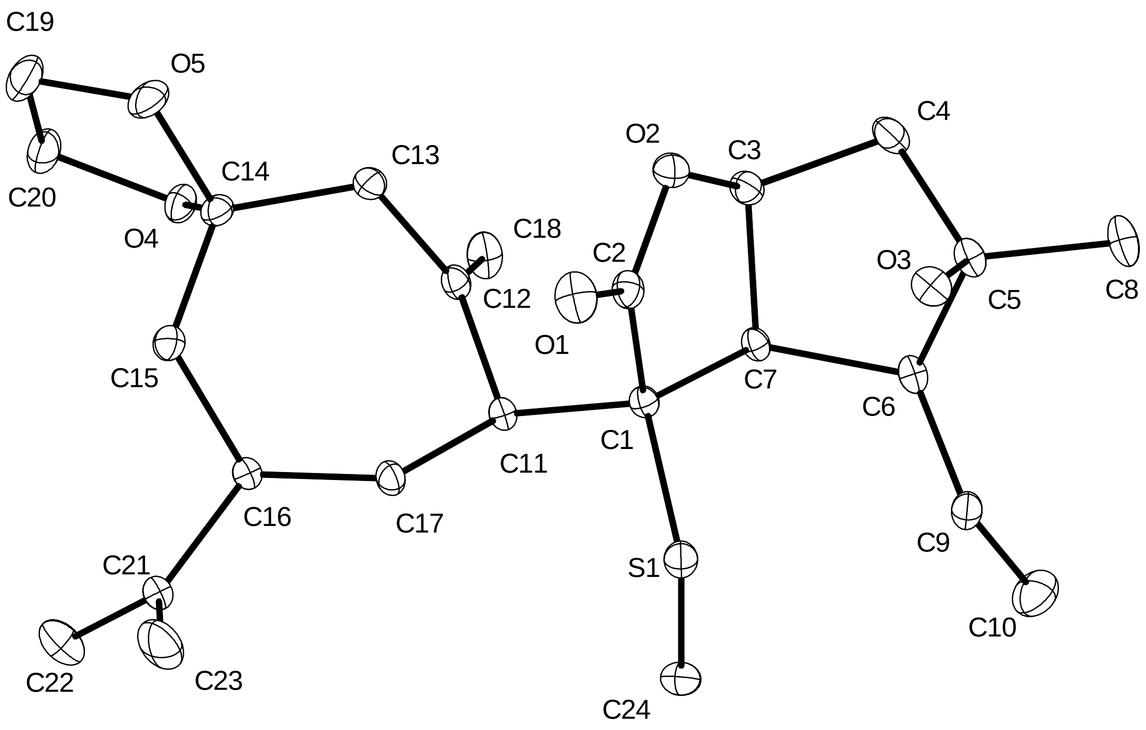

Figure S4. The molecular structure of compound **27**; atomic displacement ellipsoids are shown at the 50% probability level, H-atoms omitted for clarity.

X-ray Crystal Structure Analysis of Compound 27: $C_{24} H_{34} O_5 S$, M_r = 434.57 g mol^{-1}, colorless block, crystal size 0.20 x 0.086 x 0.069 mm^3, monoclinic, space group $P2_1$ [4], a = 7.7623(4) Å, b = 14.9928(8) Å, c = 9.5949(5) Å, β = 99.718(2)°, V = 1100.62(10) Å^3, T = 100(2) K, Z = 2, D_{calc} = 1.311 g·cm^3, λ = 0.71073 Å, $\mu(Mo\text{-}K_\alpha)$ = 0.180 mm^{-1}, Gaussian absorption correction ($T_{\min}$ = 0.97224, $T_{\max}$ 0.99028), Bruker-AXS Kappa Mach3 with APEX-II detector and IµS microfocus X-ray source, 2.153 < θ < 36.317°, 57484 measured reflections, 10508 independent reflections, 10064 reflections with $I > 2\sigma(I)$, R_{int} = 0.0292.

The structure was solved by *SHELXT* and refined by full-matrix least-squares (*SHELXL*) against F^2 to R_1 = 0.0272 [$I > 2\sigma(I)$], wR_2 = 0.0725 [all data], 275 parameters and 1 restraints, absolute structure parameter Flack (x) = –0.007(10) [4613 quotients]. **CCDC-2132679**

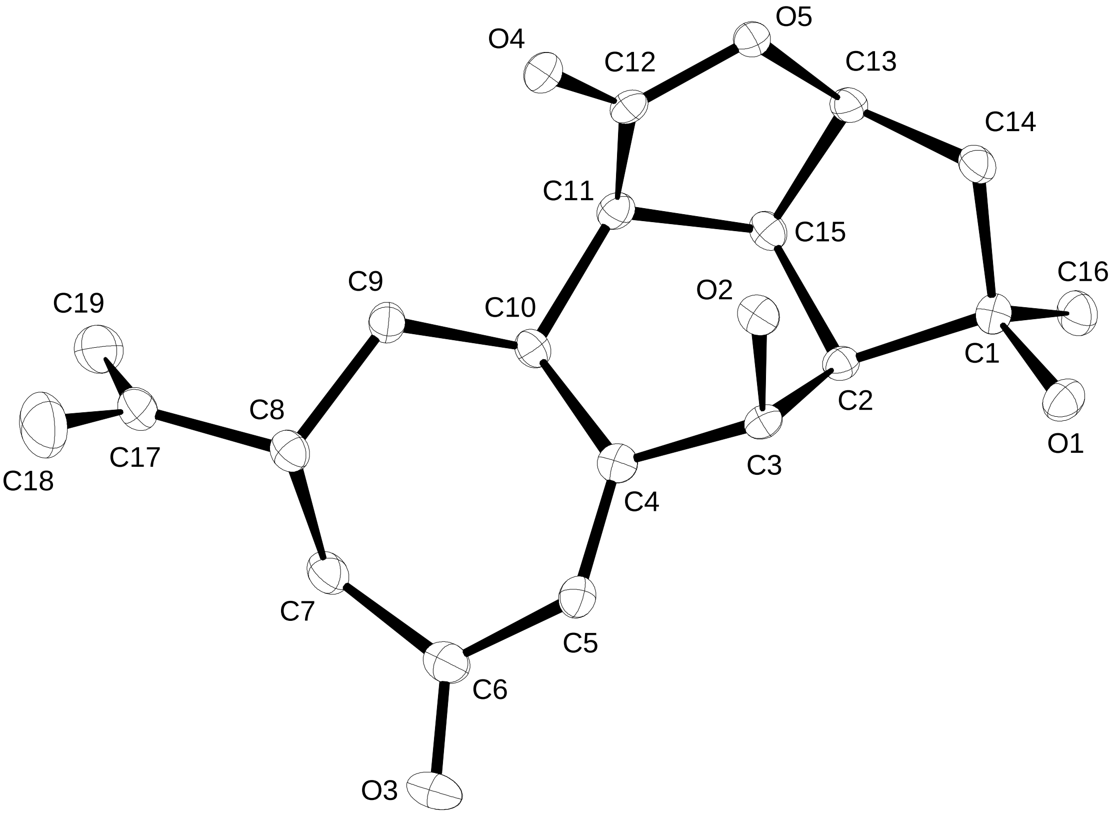

Figure S5. Molecular structure of **33** in the solid state; atomic displacement ellipsoids are shown at the 50% probability level, H-atoms omitted for clarity.

Crystal Data for Compound 33: $C_{19} H_{24} O_5$, M = 332.38 g · mol^{-1}, colorless needles, crystal dimensions 0.29 x 0.045 x 0.030 mm, orthorhombic, $P2_12_12_1$ (no. 19), T = 100(2) K, a = 6.1630(7), b = 13.351(2), c = 19.538(3) Å , V = 1607.6(4) Å^3, Z = 4, ρ = 1.373 Mg · m^{-3}, $\mu(Mo\text{-}K_\alpha)$ = 0.099 mm^{-1}, λ = 0.71073 Å. Gaussian absorption correction ($T_{\min}$ = 0.98, $T_{\max}$ = 1.00), Bruker AXS Enraf-Nonius KappaCCD with a FR591 rotating Mo-anode X-ray source, 3.052 < θ < 30.057, 24974 measured reflections, 4725 independent reflections, 3652 reflections with $I > 2\sigma(I)$, R_{int} = 0.0624, absolute structure parameter = –0.1(5), 313 parameters, residual electron density +0.3 (0.81 Å from C15) / -0.2 (0.61 Å from C15) e · Å^{-3}.

The structure was solved by *SHELXT* and refined by full-matrix least-squares (*SHELXL*) against F^2 to R_1 = 0.046 [$I > 2\sigma(I)$], wR_2 = 0.088. **CCDC-2121819.**

General. Unless stated otherwise, all reactions were carried out in flame-dried glassware using anhydrous solvents under argon. “Ambient temperature” refers to a temperature in the range between 19-25 °C, which as not determined more accurately. The solvents were purified by distillation over the indicated drying agents and were transferred under argon: THF, Et_2O (Mg/anthracene), CH_2Cl_2 (CaH_2), toluene (Na/K), MeOH (Mg, stored over MS 3 Å); DMF, MeCN, Et_3N, pentane and pyridine were dried by an adsorption solvent purification system based on molecular sieves. Thin layer chromatography (TLC): Macherey-Nagel precoated plates (POLYGRAM®SIL/UV254); Preparative TLC: Macherey-Nagel precoated plates (SIL G-100 UV 254; silica gel layer: 1.0 mm); Flash chromatography: Merck silica gel 60 (40–63 μm) with predistilled or HPLC grade solvents; Celite® was dried at 170 °C for 48 h under high vacuum (1×10^{-3} mbar) and stored under argon. NMR: Spectra were recorded on Bruker DPX 300, AV 400, AV 500 or AVIII 600 spectrometers in the solvents indicated; chemical shifts (δ) are given in ppm relative to TMS, coupling constants (*J*) in Hz. The solvent signals were used as references and the chemical shifts converted to the TMS scale ($CDCl_3$: δ_C = 77.0 ppm; residual $CHCl_3$ in $CDCl_3$: δ_H = 7.26 ppm). IR: Alpha Platinum ATR instrument (Bruker), wavenumbers ($\tilde{\nu}$) in cm^{-1}. MS (EI): Finnigan MAT 8200 (70 eV), ESI-MS: ESQ3000 (Bruker); accurate mass determinations: Bruker APEX III FTMS (7 T magnet) or Mat 95 (Finnigan). Optical rotations ($[\alpha]_D$) were measured with a A-Krüss Otronic Model P8000-t polarimeter. LC-MS analyses were conducted on a Shimadzu LCMS2020 instrument (pumps LC-20AD, autosampler SIL-20AC, column oven CTO-20AC, diode array detector SPD-M20A, controller CBM-20A, ESI detector and software Labsolutions) with an ZORBAX Eclipse Plus C18 1.8 μm, 3.0 or 4.6 mm ID × 50 mm (Agilent). A binary gradient of MeCN or MeOH in water or aq. ammonium bicarbonate buffer (pH 9) was used at a flow rate of 0.5 (3.0 mm ID) or 0.8 (4.6 mm ID) mL/min. The oven temperature was kept at 35 °C and the detection wave length at 205 nm. Preparative LC was performed with a Shimadzu LC-20A prominence system (pumps LC-20AP, column oven CTO-20AC, diode array detector SPD-M20A, fraction collector FRC-10A, controller CBM-20A and software LC-solution); conditions for each compound are specified below. Unless stated otherwise, all commercially available compounds (Alfa Aesar, Aldrich, TCI, Strem Chemicals) were used as received.

Experimental Details and Characterization Data

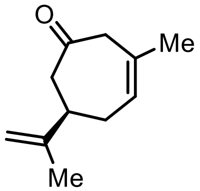

(*R*)-3-Methyl-6-(prop-1-en-2-yl)cyclohept-3-en-1-one (9).[1,2] TMSCN (10.7 mL, 85.5 mmol) was added to a solution of (*R*)-carvone (**7**) (10.0 mL, 63.8 mmol) and NMO (2.25 g, 19.2 mmol) in CH_2Cl_2 (64 mL) at ambient temperature. The resulting mixture was stirred for 1 d before the solvent was removed and the residue purified by chromatography on silica gel (hexanes/*tert*-butyl methyl ether, 20:1 to 10:1) to afford the desired cyanohydrin, which was used in the next step without further characterization.

This product was dissolved in Et_2O (10 mL) and the resulting solution slowly added to a solution of $LiAlH_4$ (4.78 g, 126.0 mmol) in Et_2O (50 mL) at 0 °C. The suspension was stirred at this temperature for 2 h. The reaction was then quenched by the successive addition of water (3.9 mL), 15% aqueous NaOH (3.9 mL) and again water (12 mL). The solid material was removed by passing the suspension through a pad of Celite®, which was carefully rinsed with EtOAc (300 mL). After the evaporation of the combined filtrates under vacuum, the resulting solid material was used for the next step without further purification.

A solution of this solid material in aqueous HOAc (10% *v/v*, 115 mL) was treated with a solution of $NaNO_2$ (1.3 M in H_2O, 77.0 mL, 100 mmol) at 0 °C. The mixture was stirred for 4 h at 0 °C before NaOH (3 M) was added until a pH ≈ 8 was reached. The aqueous phase was extracted with *tert*-butyl methyl ether (3 × 200 mL), the combined organic layers were washed with brine (30 mL) and dried over Mg_2SO_4. After filtration and removal of the solvent under vacuum, the residue was purified by flash chromatography on silica gel (hexanes/*tert*-butyl methyl ether, 20:1 to 10:1) to afford the title compound as a yellow oil (7.6 g, 71% over 3 steps). $[\alpha]_D^{20}$ = 29.8 (c = 4.0, $CHCl_3$); ^{1}H NMR (400 MHz, $CDCl_3$): δ = 5.56 (ddt, *J* = 6.8, 5.1, 1.6 Hz, 1H), 4.75 (dt, *J* = 1.7, 0.9 Hz, 1H), 4.73 (p, *J* = 1.5 Hz, 1H), 3.34–3.27 (m, 1H), 2.99 (d, *J* = 14.8 Hz, 1H), 2.81–2.70 (m, 1H), 2.59 (d, *J* = 8.1 Hz, 2H), 2.35–2.16 (m, 2H), 1.78 (p, *J* = 1.4 Hz, 3H), 1.72 (dd, *J* = 1.4, 0.8 Hz, 3H) ppm; ^{13}C NMR (101 MHz, $CDCl_3$): δ = 208.0, 148.1, 130.2, 124.4, 110.0, 48.8, 48.1, 43.1, 33.0, 26.0, 20.4 ppm; IR (film) $\tilde{\nu}$ = 2970, 2913, 1702, 1645, 1437, 1377, 1248, 1123, 892, 752 cm^{-1}; MS (EI): *m*/*z* (%): 107 (21.9), 122 (37.7); HRMS (ESI): *m/z:* calcd. for $C_{11}H_{16}ONa$ [M+Na^+]: 187.10933, found: 187.10950.

(1*S*,5*R*,7*R*)-1-Methyl-5-(prop-1-en-2-yl)-8-oxaspiro[bicyclo[5.1.0]octane-3,2'-[1,3]dioxolane (10). TMSOTf (0.11 mL, 0.608 mmol) was added to a solution of 1,2-bis(trimethylsiloxy)ethane (17.9 mL, 73.0 mmol) and ketone **9** (10.0 g, 60.9 mmol) in CH_2Cl_2 (6 mL) at –78 °C. After stirring at this temperature for 1 h, the mixture was warmed to –20 °C and stirred for 15 min before pyridine (2.5 mL, 30.9 mmol) was added. The mixture was then quickly poured into a cold solution of sat. $NaHCO_3$ (100 mL). The aqueous phase was extracted with EtOAc (3 x 200 mL), the combined organic layers were washed with brine (50 mL), dried with anhydrous Na_2SO_4, filtered and evaporated, and the residue was dried under high vacuum.

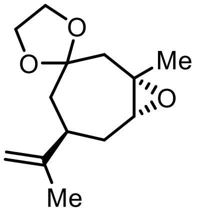

The crude material was dissolved in CH_2Cl_2 (300 mL) and the resulting solution chilled to –20 °C. $NaHCO_3$ (25.5 g, 304 mmol) and *meta*-chloroperoxybenzoic acid (70%, 15.1 g, 61.3 mmol) were successively added and the solution stirred for 2h. The reaction was quenched with sat. Na_2CO_3 (200 mL), the resulting mixture was extracted with CH_2Cl_2 (3 x 300 mL), the combined organic layers were washed with brine (50 mL), dried with anhydrous Na_2SO_4 and filtered. After removing the solvent, the crude material was purified by flash chromatography on silica gel (hexanes/*tert*-butyl methyl ether, 20:1 to 10:1) to afford the title compound as a colorless oil (10.6 g, 77%). $[\alpha]_D^{20}$ = –12.3 (c = 0.3, $CHCl_3$); ^1H NMR (400 MHz, $CDCl_3$): δ = 4.70 (dt, *J* = 1.7, 0.9 Hz, 1H), 4.66 (p, *J* = 1.5 Hz, 1H), 4.02–3.88 (m, 4H), 2.93 (t, *J* = 7.2 Hz, 1H), 2.43 (tdd, *J* = 12.3, 2.5, 1.1 Hz, 1H), 2.27 (dddd, *J* = 13.9, 7.0, 2.1, 1.1 Hz, 1H), 2.17 (ddd, *J* = 14.1, 2.7, 0.8 Hz, 1H), 1.97 (d, *J* = 14.2 Hz, 1H), 1.88 (dq, *J* = 13.5, 2.4 Hz, 1H), 1.69 (dd, *J* = 1.5, 0.8 Hz, 3H), 1.72–1.59 (m, 1H), 1.45–1.32 (m, 1H), 1.38 (d, *J* = 0.7 Hz, 3H) ppm; ^{13}C NMR (101 MHz, $CDCl_3$): δ = 149.6, 109.3, 109.1, 64.9, 64.0, 61.4, 56.5, 46.8, 43.9, 38.7, 36.4, 25.2, 19.9 ppm; IR (film) $\tilde{\nu}$ = 2966, 2938, 2880, 1645, 1376, 1335, 1230, 1135, 1081, 888, 745 cm^{-1}; MS (EI)*:* *m*/*z* (%): 139 (100), 224 (10.6); HRMS (ESI): *m/z:* calcd. for $C_{13}H_{20}O_3$ [M^+]: 224.14070, found: 224.14056.

(8*R*,10*R*)-7-Methylene-10-(prop-1-en-2-yl)-1,4-dioxaspiro[4.6]undecan-8-ol (11). *n*-BuLi (1.6 M in hexanes, 54.0 mL, 86.4 mmol) was added to a solution of 2,2,6,6-tetramethylpiperidine (15.0 mL, 88.9 mmol) in toluene (100 mL) at 0 °C and the resulting mixture was stirred for 30 min. Diethylaluminum chloride (25% in toluene, 42 mL, 83.7 mmol) was then added, causing the formation of a turbid

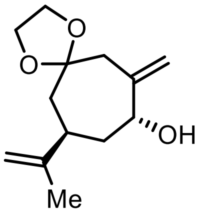

mixture. After stirring for further 40 min, a solution of epoxide **10** (9.16 g, 40.8 mmol) in toluene (40 mL) was added and stirring was continued for 20 min before the reaction was quenched by the slow addition of sat. $NaHCO_3$ (20 mL) at 0 °C. The resulting mixture was extracted with EtOAc (3 x 200 mL), the combined organic phases were washed with brine (30 mL), dried over anhydrous Na_2SO_4 and filtered. After evaporation of the solvent, the residue was purified by flash chromatography on silica gel (hexanes/EtOAc, 3:1 to 1:1) to afford the title compound as a colorless oil (9.13 g, quant.). $[\alpha]_D^{20}$ = 45.5 (c = 1.5, $CHCl_3$); ^1H NMR (400 MHz, $CDCl_3$): δ = 5.27–5.21 (m, 1H), 5.04 (dt, *J* = 1.7, 0.9 Hz, 1H), 4.69 (dt, *J* = 1.8, 0.9 Hz, 1H), 4.63 (t, *J* = 1.6 Hz, 1H), 4.38–4.23 (m, 1H), 4.02–3.85 (m, 4H), 2.66 (dd, *J* = 13.8, 1.0 Hz, 1H), 2.37 (dq, *J* = 13.9, 0.8 Hz, 1H), 2.21–2.08 (m, 2H), 1.81–1.75 (m, 2H), 1.69 (dd, *J* = 1.4, 0.8 Hz, 4H), 1.51 (ddd, *J* = 13.2, 11.3, 10.1 Hz, 1H) ppm; ^{13}C NMR (101 MHz, $CDCl_3$): δ = 150.2, 146.9, 115.5, 109.8, 108.9, 74.4, 64.4, 64.1, 44.6, 42.7, 41.4, 38.1, 20.0 ppm; IR (film) $\tilde{\nu}$ = 3410, 2934, 2879, 1646, 1104, 1021, 890, 723 cm^{-1}; MS (EI): *m*/*z* (%): 139 (100), 224 (6); HRMS (ESI): *m/z:* calcd. for $C_{13}H_{20}O_3Na$ [*M*+Na^+]: 247.13046, found: 247.13061.

(*R*)-(10-(Prop-1-en-2-yl)-1,4-dioxaspiro[4.6]undec-7-en-7-yl)methanol (12). Et_3N (1.8 mL, 12.9 mmol) and methanesulfonyl chloride (0.38 mL, 4.91 mmol) were successively added to a solution of the allylic alcohol **11** (1.000 g, 4.46 mmol) in CH_2Cl_2 (14.0 mL) at 0 °C. The mixture was stirred for 1 h before sat. aq. $NaHCO_3$ (20 mL) was introduced. The resulting mixture was stirred at ambient temperature for 2 d before it was extracted with EtOAc (3 x 50 mL). The combined organic layers were washed with brine (10 mL), dried over $MgSO_4$ and filtered. The solvent was removed under vacuum and the residue purified by flash chromatography on silica gel (hexanes/EtOAc, 5:1 to 2:1) to afford the title compound as a colorless oil (440 mg, 44 %). $[\alpha]_D^{20}$ = 82.2 (c = 1.0, $CHCl_3$); ^1H NMR (400 MHz, $CDCl_3$): δ = 5.92 (dddd, *J* = 6.9, 5.7, 2.2, 1.1 Hz, 1H), 4.70 (dt, *J* = 1.8, 0.9 Hz, 1H), 4.66 (p, *J* = 1.5 Hz, 1H), 4.02–3.86 (m, 6H), 2.70 (dd, *J* = 14.4, 2.1 Hz, 1H), 2.40 (ddd, *J* = 14.4, 2.3, 1.1 Hz, 1H), 2.32–2.22 (m, 1H), 2.22–2.12 (m, 2H), 2.06–1.85 (m, 3H), 1.70 (dd, *J* = 1.5, 0.8 Hz, 3H) ppm; ^{13}C NMR (101 MHz, $CDCl_3$): δ = 150.2, 136.7, 128.5, 109.1, 107.7, 68.9, 64.7, 64.2, 46.3, 41.9, 38.6, 32.7, 20.2 ppm; IR (film) $\tilde{\nu}$ = 3425, 2919, 2883, 1450, 1273, 1112, 1068, 951, 783 cm^{-1}; MS (EI): *m*/*z* (%): 139 (100), 224 (0.34); HRMS (ESI): *m/z:* calcd. for $C_{13}H_{20}O_3$ [M^+]: 224.14070, found: 224.14051.

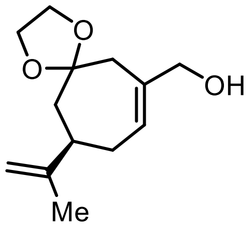

(*R*)-7-(Chloromethyl)-10-(prop-1-en-2-yl)-1,4-dioxaspiro[4.6]undec-7-ene (23a). $SOCl_2$ (3.8 mL, 52.4 mmol) was added dropwise to a solution of allylic alcohol **11** (1.48 g, 6.60 mmol) and pyridine (11.2 mL, 138 mmol) in diethylether (660 mL) at 0 °C. After 30 min, the reaction was quenched with sat. $NaHCO_3$ (50 mL), and the aqueous phase was extracted with diethyl ether (2 x 300). The combined organic layers were washed with brine (50 mL), dried with anhydrous Na_2SO_4, filtered and evaporated. The crude material was purified by flash chromatography on silica gel (hexanes/*tert*-butyl methyl ether, 10:1) to provide the title compound as a colorless oil (1.42 g, 89 %). $[\alpha]_D^{20}$ = 4.2 (c = 1.3, $CHCl_3$); ^{1}H NMR (400 MHz, $CDCl_3$): δ = 6.03 (tdq, *J* = 6.3, 2.0, 1.0 Hz, 1H), 4.77–4.67 (m, 1H), 4.67 (d, *J* = 1.6 Hz, 1H), 4.05 (q, *J* = 1.1 Hz, 2H), 4.01–3.91 (m, 4H), 2.69–2.63 (m, 1H), 2.47 (ddd, *J* = 14.8, 2.2, 1.0 Hz, 1H), 2.37–2.28 (m, 1H), 2.25–2.13 (m, 2H), 2.01–1.96 (m, 1H), 1.95–1.88 (m, 1H), 1.71 (dd, *J* = 1.5, 0.8 Hz, 3H) ppm; ^{13}C NMR (101 MHz, $CDCl_3$): δ = 150.0, 134.0, 131.0, 109.2, 107.8, 64.9, 64.2, 52.4, 46.4, 41.4, 38.7, 33.1, 20.2 ppm; IR (film) $\tilde{\nu}$ = 2944, 2882, 1644, 1439, 1258, 1113, 1064, 891, 680 cm^{-1}; MS (EI): *m*/*z* (%): 139 (100), 207 (28.6); HRMS (ESI): *m/z:* calcd. for $C_{13}H_{19}O_2ClNa$ [*M*+Na$^+$]: 265.09658, found: 265.09676.

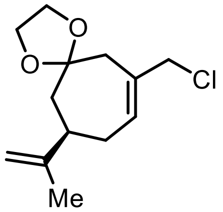

Note: The low concentration prevents the undesired dimerization of the allylic alcohol.

(*R*)-4-((*tert*-Butyldimethylsilyl)oxy)-4-methylcyclopent-2-en-1-one (15).[3,4] The second generation Hoveyda-Grubbs catalyst **21** (81.0 mg, 0.129 mmol) was added to (*R*)-linalool (**14**) (11.6 mL, 64.8 mmol) at ambient temperature and the resulting mixture was stirred until the evolution of gas had ceased. To quench the catalyst, the argon line was removed and the mixture was exposed to air for 20 min. The resulting crude product was added to a solution of NaH (4.66 g, 194 mmol) in THF (220 mL) at 0 °C. After stirring for 5 min, TBSCl (14.09 g, 92.9 mmol) was introduced and stirring was continued for 10 min before the mixture was warmed to 65 °C. After 4 h, the mixture was cooled to ambient temperature and then quickly poured into a cold solution of sat. $NaHCO_3$ (500 mL). The aqueous phase was extracted with Et_2O (2 x 500 mL), the combined organic layers were washed with brine (100 mL), dried over $MgSO_4$, filtered and concentrated in vacuum. The crude product was rapidly purified by flash chromatography on silica gel (2% triethylamine in hexanes: diethyl ether → 20:1) to provide a yellow oil.

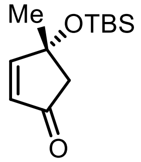

This product was dissolved in CH_2Cl_2 (140 mL) and H_2O (14 mL) and $RuCl_3{\cdot}H_2O$ (271 mg, 1.31 mmol), and $Mg(OAc)_2{\cdot}4H_2O$ (27.8 g, 130 mmol, 2.0) were introduced. *tert*-Butyl hydrogen peroxide (TBHP, 70% in H_2O, 140 mL, 1.02 mol) was then added dropwise via a dropping funnel over a period of 8 h, and stirring was continued for an additional 15 h before the reaction was quenched with sat. aq. Na_2SO_3 (500 mL). The aqueous phase was extracted with *tert*-butyl methyl ether (3 x 300 mL), the combined organic layers were washed with brine (100 mL), dried over $MgSO_4$, filtered and evaporated. The residue was purified by flash chromatography on silica gel (hexanes:*tert*-butyl methyl ether, 50:1 to 20:1) to afford the title compound as a colorless oil (8.1 g, 55% over three steps). $[\alpha]_D^{20}$ = 19.0 (c = 4.3, $CHCl_3$); ^{1}H NMR (400 MHz, $CDCl_3$): δ = 7.40 (d, *J* = 5.6 Hz, 1H), 6.03 (d, *J* = 5.7 Hz, 1H), 2.64–2.23 (m, 2H), 1.48 (s, 3H), 0.84 (s, 9H), 0.07 (s, 3H), 0.06 (s, 3H) ppm; ^{13}C NMR (101 MHz, $CDCl_3$): δ = 207.1, 167.7, 131.8, 78.3, 51.4, 28.9, 25.5, 17.8, -2.5, -2.5 ppm; MS (EI): *m/z* (%): 149 (100), 191 (1); HRMS (ESI): *m/z:* calcd. for $C_{12}H_{22}O_2SiNa$ [*M*+Na$^+$]: 249.12813, found: 249.12838.

(1*S*,3*R*,3a*S*,7a*R*)-3-((*tert*-Butyldimethylsilyl)oxy)-3-methyl-2,3,3a,4,7,7a-hexahydro-1*H*-inden-1-ol (16).

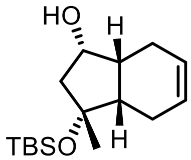

A1C1$_3$ (3.25 g, 24.4 mmol) was added in portions to a solution of enone **15** (6.0 g, 26.5 mmol) in toluene (210 mL) at 0 °C. After 40 min, a solution of 1,3-butadiene (48 mL) in toluene (50 mL) was added via cannula and the resulting mixture was stirred at ambient temperature for 1 h. The reaction was quenched with sat. aq. $NaHCO_3$ (500 mL), the aqueous phase was extracted with *tert*-butyl methyl ether (3 x 500 mL), the combined organic layers were washed with brine, dried with $MgSO_4$, filtered, and evaporated. The residue was purified by flash chromatography on silica gel (hexanes:*tert*-butyl methyl ether, 20:1) to afford the desired cycloadduct as a colorless oil.

This product was dissolved in THF (100 mL) and the solution chilled to −78 °C before L-Selectride (1 M in THF, 33 mL, 33 mmol) was added. The mixture was stirred for 1 h at this temperature before the reaction was carefully quenched with H_2O (5 mL) and H_2O_2 (35% *w/w* in H_2O, 10.5 mL, 108 mmol). After stirring for another 2 h, sat. aq. $Na_2S_2O_3$ (100 mL) was added, and the resulting mixture was extracted with *tert*-butyl methyl ether (3 x 200 mL). The combined organic layers were washed with brine (50 mL), dried with $MgSO_4$, filtered and evaporated. The residue was purified by flash chromatography on silica gel (hexanes:*tert*-butyl methyl ether, 20:1 to 10:1) to afford the title compound as a white solid material (5.2 g, 69% over 2 steps). mp = 78.5–79.9 °C

(*tert*-butyl methyl ether); $[\alpha]_D^{20}$ = 13.7 (c = 0.83, $CHCl_3$); ^{1}H NMR (600 MHz, $CDCl_3$): δ = 5.86–5.79 (m, 2H), 4.14 (dddd, *J* = 7.9, 6.8, 6.0, 4.4 Hz, 1H), 2.23–2.17 (m, 1H), 2.15–2.13 (m, 2H), 2.13–2.10 (m, 2H), 2.08 (d, *J* = 7.4 Hz, 1H), 2.07 (dd, *J* = 14.0, 7.1 Hz, 1H), 2.00 (dd, *J* = 14.1, 4.4 Hz, 1H), 1.85–1.79 (m, 1H), 1.32 (s, 3H), 0.86 (s, 9H), 0.11 (s, 3H), 0.09 (s, 3H) ppm; ^{13}C NMR (151 MHz, $CDCl_3$): δ = 128.0, 127.0, 82.4, 74.9, 50.0, 46.8, 40.8, 29.7, 26.1, 23.2, 22.6, 18.3, −1.8, −2.2 ppm; IR (film) $\tilde{\nu}$ = 3354, 2958, 2926,2893, 1252, 1161, 1043, 833, 770 cm^{-1}; MS (EI)*: m/z* (%): 91 (100), 171 (16); HRMS (ESI): *m/z:* calcd. for $C_{16}H_{30}O_2SiNa$ [*M*+Na$^+$]: 305.19073, found: 305.19073.

(3a*R*,4*S*,5*R*,6a*S*)-5-((*tert*-Butyldimethylsilyl)oxy)-4-(2-hydroxyethyl)-5-methylhexahydro-2*H*-cyclopenta[b]furan-2-one (17). Ozone gas was bubbled through a solution of compound **16** (4.50 g, 15.9 mmol) in CH_2Cl_2 (100 mL) until a deep blue color persisted. The solution was then purged with oxygen for 10 min before PPh_3 (4.20 g, 16.0 mmol) was added. The resulting mixture was warmed to ambient temperature and stirred for 1 h before the solvent was removed. The crude material was purified by flash chromatography on silica gel (hexanes:acetone, 10:1 to 2:1) to afford an unstable colorless oil.

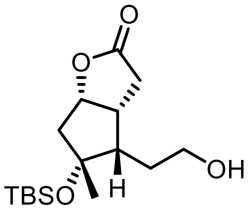

MS 4 Å (3.0 g) and PCC (8.00 g, 21.3 mmol) were successively added to a solution of this compound in CH_2Cl_2 (50 mL) at 0 °C. The mixture was stirred at this temperature for 1 h before it was quickly passed through a pad of silica, eluting with hexane:acetone (1:0 to 2:1).

Evaporation of the combined filtrates gave a yellow oil, which was dissolved in THF (50 mL). $NaBH_4$ (1.2 g, 31.7 mmol) was added in portions to this solution at 0 °C and the mixture was stirred at this temperature for 15 min before the reaction was quenched with sat. NH_4Cl (50 mL). The resulting mixture was extracted EtOAc (3 x 200 mL), the combined organic layers were washed with brine (50 mL), dried with $MgSO_4$, filtered, and evaporated. The residue was purified by flash chromatography on silica gel (hexanes:acetone, 4:1 to 1:1) to afford the title compound as a white solid (2.21 g, 44% over 3 steps). ^{1}H NMR (400 MHz, $CDCl_3$): δ = 5.03 (ddd, *J* = 8.2, 7.5, 0.7 Hz, 1H), 3.76 (dt, *J* = 10.7, 5.8 Hz, 1H), 3.70–3.57 (m, 1H), 3.13 (dtd, *J* = 11.6, 8.0, 6.0 Hz, 1H), 2.79 (dd, *J* = 18.4, 6.1 Hz, 1H), 2.45 (dd, *J* = 18.4, 11.7 Hz, 1H), 2.29 (d, *J* = 15.4 Hz, 1H), 1.94–1.78 (m, 3H), 1.78–1.67 (m, 1H), 1.43 (s, 1H), 1.33 (s, 3H), 0.86 (s, 9H), 0.11 (s, 3H), 0.11 (s, 3H) ppm;

^{13}C NMR (101 MHz, $CDCl_3$): δ = 178.0, 83.7, 83.1, 61.4, 49.5, 47.9, 40.4, 30.3, 27.9, 26.4, 26.0, 18.4, −1.9, −2.4 ppm; IR (film) $\tilde{\nu}$ = 3411, 2952, 2930, 2857, 1760, 1253, 1195, 1001, 835, 777 cm^{-1}; MS (EI): *m*/*z* (%): 165 (54), 239 (25); HRMS (ESI): *m/z:* calcd. for $C_{16}H_{30}O_4SiNa$ [*M*+Na^+]: 337.18056, found: 337.18056.

(3a*R*,4*S*,5*R*,6a*S*)-5-((*tert*-Butyldimethylsilyl)oxy)-5-methyl-4-vinylhexahydro-2*H*-cyclopenta[b]furan-2-one (18). *o*-Nitrophenylselenocyanate (2.30 g, 10.1 mmol) and *n*-Bu_3P (2.6 mL, 10.4 mmol) were successively added to a solution of alcohol **17** (2.21 g, 7.03 mmol) in THF (46 mL) at ambient temperature and the reaction mixture was stirred at this temperature for 3 h. Sodium bicarbonate (2.95 g, 35.1 mmol) was added at 0 °C, followed by aqueous hydrogen peroxide (35% *w/w* in H_2O, 5.0 mL, 51.4 mmol). The resulting mixture was warmed to ambient temperature and stirred for 2 h. The reaction was quenched with sat. $Na_2S_2O_3$ (100 mL), the aqueous phase was extracted with EtOAc (3 x 200 mL), the combined extracts were washed with brine (30 mL) and dried with $MgSO_4$. After filtration and evaporation of the solvent, the crude product was purified by flash chromatography on silica gel (toluene:EtOAc, 30:1 to 15:1) to afford the title compound as a yellow solid (1.83 g, 88 %). mp = 88.4–89.0 °C (toluene/EtOAc); $[\alpha]_D^{20}$ = –24.0 (c = 1.7, $CHCl_3$); ^1H NMR (400 MHz, $CDCl_3$): δ = 5.94 (ddd, *J* = 17.2, 10.3, 8.8 Hz, 1H), 5.26 (ddd, *J* = 10.3, 2.1, 0.5 Hz, 1H), 5.16–5.11 (m, 1H), 5.04 (ddd, *J* = 8.1, 7.2, 0.8 Hz, 1H), 3.12 (dtd, *J* = 11.7, 8.3, 5.5 Hz, 1H), 2.88 (dd, *J* = 18.6, 5.6 Hz, 1H), 2.47 (dd, *J* = 18.6, 11.8 Hz, 1H), 2.39–2.23 (m, 2H), 1.87 (ddd, *J* = 15.2, 7.2, 0.6 Hz, 1H), 1.29 (s, 3H), 0.87 (s, 8H), 0.11 (s, 3H), 0.11 (s, 3H) ppm; ^{13}C NMR (101 MHz, $CDCl_3$): δ = 177.9, 134.2, 119.1, 84.0, 83.5, 57.6, 47.7, 42.6, 30.7, 26.3, 26.0, 18.4, –2.0, –2.3 ppm; IR (film) $\tilde{\nu}$ = 2955, 2929, 1766, 1174, 1420, 1178, 1113, 998, 835 cm^{-1}; MS (EI): *m*/*z* (%): 147 (100), 195 (32.9); HRMS (ESI): *m/z:* calcd. for $C_{16}H_{28}O_3SiNa$ [*M*+Na^+]: 319.16999, found: 319.17031.

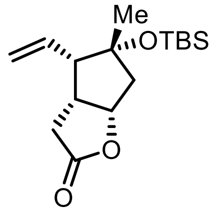

((*R*)-10-(Prop-1-en-2-yl)-1,4-dioxaspiro[4.6]undec-7-en-7-yl)methyl 2-((1*R*,2*S*,3*R*,5*S*)-3,5-bis((*tert*-butyldimethylsilyl)oxy)-3-methyl-2-vinylcyclopentyl)acetate (20). NaOH (3 M, 0.25 mL, 0.75 mmol) was added to a solution of lactone **18** (200 mg, 0.675 mmol) in MeOH (1.6 mL) at ambient temperature. After stirring for 5 h at this temperature, the solvent was removed under high vacuum. The resulting sodium salt was redissolved in DMF (0.7 mL). Imidazole (460 mg, 6.76 mmol) and TBDMSCl (813 mg, 5.39 mmol) were successively added to this solution at ambient temperature. After stirring for 10 h, the mixture was diluted with *tert*-butyl methyl ether (10 mL) and the reaction was quenched with sat. aq. NH_4Cl (5 mL). The resulting mixture was extracted with *tert*-butyl methyl ether (3 x10 mL), the combined organic layers were washed with brine (5 mL), dried over anhydrous Na_2SO_4, filtered and concentrated. The residue was passed through a pad of silica and used in the next step without further purification.

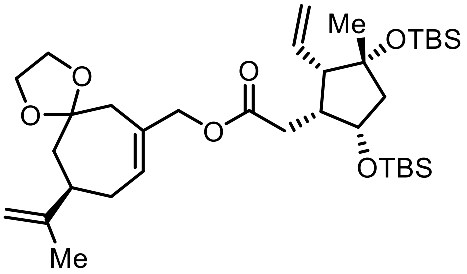

Et_3N (0.5 mL, 3.59 mmol), DCC (433 mg, 2.10 mmol) and DMAP (85.5 mg, 0.70 mmol) were successively added to a solution of carboxylic acid **19** thus formed and allylic alcohol **12** (440 mg, 1.96 mmol) in CH_2Cl_2 (3.5 mL) at ambient temperature. After 2 h, the mixture was diluted with H_2O (10 mL), the aqueous phase was extracted with EtOAc (3 x 10 mL), the combined organic layers were washed with brine (5 mL), dried over anhydrous Na_2SO_4 and filtered. After evaporation of the solvent, the crude material was purified by flash chromatography on silica gel (hexanes:*tert*-butyl methyl ether, 20:1 to 10:1) to afford the title compound as a colorless oil (263 mg, 59%). $[\alpha]_D^{20}$ = 45.0 (c = 1.1, $CHCl_3$); ^{1}H NMR (600 MHz, $CDCl_3$): δ = 5.96 (td, *J* = 6.1, 3.1 Hz, 1H), 5.94–5.89 (m, 1H), 4.99 (dd, *J* = 10.2, 2.4 Hz, 1H), 4.88 (ddd, *J* = 17.2, 2.4, 0.7 Hz, 1H), 4.70 (dt, *J* = 1.8, 0.8 Hz, 1H), 4.66 (t, *J* = 1.6 Hz, 1H), 4.50–4.37 (m, 2H), 4.20 (ddd, *J* = 7.5, 6.0, 4.9 Hz, 1H), 4.00–3.89 (m, 4H), 2.61–2.47 (m, 3H), 2.42–2.37 (m, 1H), 2.34–2.27 (m, 3H), 2.22–2.09 (m, 3H), 2.02–1.82 (m, 3H), 1.75–1.69 (m, 3H), 1.36–1.19 (m, 3H), 0.86 (s, 9H), 0.85 (s, 9H), 0.07 (s, 3H), 0.04 (s, 3H), 0.02 (s, 3H), –0.02 (s, 3H) ppm; ^{13}C NMR (151 MHz, $CDCl_3$): δ = 173.7, 150.2, 137.4, 132.6, 129.5, 116.1, 109.1, 107.8, 80.6, 72.6, 69.5, 64.9, 64.1, 59.1, 51.4, 46.5, 43.3, 41.6, 38.1, 32.9, 30.9, 30.7, 25.9, 25.8, 20.2, 18.2, 18.0, –2.1, –2.2, –4.7, –5.2 ppm; IR (film) $\tilde{\nu}$ = 2929, 2855, 1699, 1472, 1254, 1069, 834, 754 cm^{-1}; MS (ESI): *m/z*: 635 [*M*+H$^+$], 657 [M+Na$^+$]; HRMS (ESI): *m/z:* calcd. for $C_{35}H_{62}O_5Si_2Na$ [*M*+Na$^+$]: 657.39772, found: 657.39802.

(3*S*,3a*R*,4*S*,5*R*,6a*S*)-5-((*tert*-Butyldimethylsilyl)oxy)-5-methyl-3-(methylthio)-4-vinylhexahydro-2*H*-cyclopenta[b]furan-2-one (22) . LiHMDS (1.0 M in THF, 6.8 ml, 6.8 mmol) was added

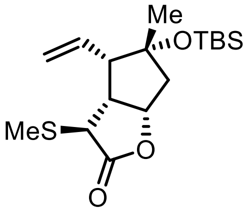

to a solution of **18** (1.83 g, 6.17 mmol) in THF (31.0 ml) at −78 °C. The resulting solution was stirred at this temperature for 40 min before a solution of methyl methanethiolsulfonate (0.29 mL, 3.07 mmol) in THF (1.5 mL) was added. Stirring was continued at this temperature for 30 min, before the mixture was warmed to –30 °C. After stirring for another 2 h, the mixture was diluted with sat. $NaHCO_3$ (10 mL), the aqueous phase was extracted with *tert*-butyl methyl ether (3 x 100 mL), the combined organic layers were washed with brine (30 mL), dried with $MgSO_4$ and filtered. After evaporation of the solvent, the residue was purified by flash chromatography on silica gel (hexanes:*tert*-butyl methyl ether, 20:1 to 10:1) to afford the title compound as a colorless oil (1.02 g, 95% based on methyl methanethiolsulfonate). $[\alpha]_D^{20}$ = −44.1 (c = 0.7, $CHCl_3$); ^1H NMR (400 MHz, $CDCl_3$): δ = 5.94 (ddd, *J* = 17.1, 10.3, 8.7 Hz, 1H), 5.34 (ddd, *J* = 10.4, 1.9, 0.6 Hz, 1H), 5.24 (ddd, *J* = 17.2, 1.9, 0.9 Hz, 1H), 5.00 (ddd, *J* = 8.0, 7.2, 0.8 Hz, 1H), 3.72 (d, *J* = 4.9 Hz, 1H), 2.90 (dddd, *J* = 8.7, 8.0, 4.9, 0.6 Hz, 1H), 2.39 (t, *J* = 8.8 Hz, 1H), 2.33–2.29 (m, 1H), 2.28 (s, 3H), 1.85 (ddd, *J* = 15.3, 7.1, 0.6 Hz, 1H), 1.30 (s, 3H), 0.87 (s, 9H), 0.11 (s, 3H), 0.10 (s, 3H) ppm; ^{13}C NMR (101 MHz, $CDCl_3$): δ = 176.1, 133.1, 120.0, 83.3, 82.1, 57.7, 50.8, 47.5, 43.1, 26.1, 26.0, 18.5, 14.9, −2.0, −2.3 ppm; IR (film) $\tilde{\nu}$ = 2954, 2928, 2856, 1763, 1193, 1110, 998, 777 cm^{-1}; MS (EI)*:* *m*/*z* (%): 209 (78.5), 285 (68.3); HRMS (ESI): *m/z:* calcd. for $C_{17}H_{30}O_3SiSNa$ [*M*+Na^+]: 365.15772, found: 365.15802.

(3*S*,3a*R*,4*S*,5*R*,6a*S*)-5-Hydroxy-5-methyl-3-((8*R*,10*R*)-7-methylene-10-(prop-1-en-2-yl)-1,4-dioxaspiro[4.6]undecan-8-yl)-3-(methylthio)-4-vinylhexahydro-2*H*-cyclopenta[b]furan-2-

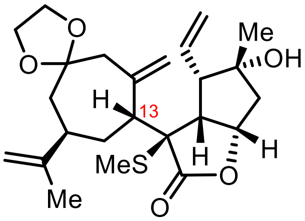

one (26) and Isomer 27. NaI (1.360 g, 9.1 mmol) was added to a solution of allylic chloride **23** (X = Cl, 2.200 g, 9.06 mmol) in acetone (10 mL) at ambient temperature. The mixture was stirred at 50 °C for 12 h. After cooling to ambient temperature, the suspension was filtered, the filtrate was evaporated, and the residue was quickly passed through a pad of silica, eluting with hexanes: *tert*-butyl methyl ether (10:1) to provide the desired allyl iodide **23c** (X = I).

$AgBF_4$ (1.57 g, 8.06 mmol) was added to a solution of **23c** in CH_3CN (6.7 mL), followed by 2,6-*tert*-butyl-4-methylpyridine (2.07 g, 10.1 mmol) and methyl sulfide **22** (690 mg, 2.01 mmol). The mixture was vigorously stirred for 3 h at ambient temperature before it was filtered through a pad of Celite®, which was carefully rinsed with CH_3CN. After evaporation of the combined filtrates, the crude material was redissolved in CH_3CN (10 mL). *t*-BuOK (1.40 g, 12.5 mmol) was added to the above solution at ambient temperature and the mixture was stirred for 2 h before the reaction was quenched with sat. $NaHCO_3$ (10 mL). The resulting mixture was extracted with EtOAc (3 x 50 mL), the combined organic layers were washed with brine (10 mL), dried with $MgSO_4$ and filtered. After evaporation of the solvent, the residue was purified by flash chromatography on silica gel (hexanes:*tert*-butyl methyl ether, 10:1 to 4:1) to afford compound **25** as a colorless oil.

This material was dissolved in THF (6.7 mL). TBAF (1 M in THF, 1.6 mL, 1.60 mmol) was added and the resulting solution was stirred at 80 °C for 4 h. The reaction was cooled to ambient temperature, the solvent was removed, and the residue was purified by flash chromatography on silica gel (hexanes:acetone, 10:1 to 4:1) to afford the title compound **26** (275 mg, 31% over 3 steps) and isomer **27** (286 mg, 33% over 3 steps) as white solid each. $[\alpha]_D^{24}$ = 5.7 (c = 0.35, $CHCl_3$); ^1H NMR (400 MHz, $CDCl_3$): δ = 6.23 (dt, *J* = 17.0, 9.9 Hz, 1H), 5.28 (dd, *J* = 10.2, 1.9 Hz, 1H), 5.17 (ddd, *J* = 17.0, 1.8, 0.9 Hz, 1H), 5.13 (s, 1H), 5.10 (d, *J* = 1.1 Hz, 1H), 4.82 (q, *J* = 1.4 Hz, 1H), 4.74 (q, *J* = 1.1 Hz, 1H), 4.64 (td, *J* = 7.8, 3.5 Hz, 1H), 4.03–3.98 (m, 1H), 3.96–3.86 (m, 3H), 3.23 (dd, *J* = 9.1, 8.0 Hz, 1H), 3.00 (s, 1H), 2.91 (dd, *J* = 12.8, 3.4 Hz, 1H), 2.71–2.56 (m, 3H), 2.49 (dp, *J* = 11.6, 4.1 Hz, 1H), 2.31–2.26 (m, 1H), 2.24 (s, 3H), 2.17 (d, *J* = 3.5 Hz, 1H), 2.12 (dd, *J* = 15.2, 7.6 Hz, 1H), 2.00 (dd, *J* = 14.1, 12.4 Hz, 1H), 1.89 (ddd, *J* = 14.8, 12.7, 6.9 Hz, 1H), 1.80 (dd, *J* = 1.4, 0.7 Hz, 3H), 1.63–1.55 (m, 1H), 1.23 (s, 3H) ppm; ^{13}C NMR (101 MHz, $CDCl_3$): δ = 176.6, 148.4, 144.0, 134.3, 119.8, 118.5, 110.7, 110.0, 80.6, 78.6, 64.6, 64.0, 59.4, 58.3, 49.9, 48.2, 46.9, 41.1, 37.6, 36.3, 27.6, 27.4, 22.6, 12.6 ppm; IR (film) $\tilde{\nu}$ = 3466, 2966, 2830, 1186, 1111, 1104, 894, 758 cm^{-1}; MS (EI): *m/z* (%): 139 (60), 434 (6); HRMS (ESI): *m/z:* calcd. for $C_{24}H_{34}O_5SNa$ [M+Na^+]: 457.20192, found: 457.20248.

Spectral and analytical data of isomer **27:** $[\alpha]_D^{20}$ = –167.4 (c = 0.53, $CHCl_3$); ^1H NMR (600 MHz, $CDCl_3$): δ = 6.19 (dt, *J* = 17.0, 10.1 Hz, 1H), 5.24 (d, *J* = 1.7 Hz, 1H), 5.21 (dd, *J* = 10.1, 1.9 Hz, 1H), 5.19 (dt, *J* = 1.8, 0.9 Hz, 1H), 5.14 (ddd, *J* = 16.9, 1.9, 0.8 Hz, 1H), 4.78 (td, *J* = 7.8, 4.1 Hz,

1H), 4.65 (dt, J = 1.7, 0.8 Hz, 1H), 4.60 (p, J = 1.5 Hz, 1H), 4.02–3.99 (m, 1H), 3.98–3.93 (m, 2H), 3.92–3.88 (m, 1H), 3.36 (t, J = 8.1 Hz, 1H), 2.85 (s, 1H), 2.77 (dd, J = 12.1, 6.0 Hz, 1H), 2.65–2.58 (m, 1H), 2.42 (d, J = 13.3 Hz, 1H), 2.30 (ddd, J = 13.3, 2.4, 0.8 Hz, 1H), 2.19 (ddd, J = 14.8, 7.8, 0.7 Hz, 1H), 2.15 (s, 3H), 2.12–2.05 (m, 2H), 2.04 (ddd, J = 11.5, 9.3, 2.2 Hz, 1H), 1.86–1.72 (m, 2H), 1.78–1.69 (m, 1H), 1.68 (dd, J = 1.5, 0.8 Hz, 3H), 1.24 (s, 3H) ppm; ^{13}C NMR (151 MHz, $CDCl_3$): δ = 175.1, 150.7, 139.8, 134.4, 122.9, 119.2, 109.1, 108.9, 81.2, 78.6, 64.7, 64.5, 58.4, 58.1, 51.3, 51.1, 46.8, 46.5, 42.2, 40.0, 34.5, 28.0, 20.4, 13.5 ppm; IR (film) $\tilde{\nu}$ = 3478, 2969, 2880, 1751, 1173, 1105, 998, 916, 754 cm^{-1}; MS (ESI): 435 [M+H$^+$], 457 [M+Na$^+$]; HRMS (ESI): *m/z:* calcd. for $C_{24}H_{34}O_5SNa$ [M+Na$^+$]: 457.20192, found: 457.20190.

(3*S*,3a*S*,4*S*,5*R*,6a*S*)-5-Hydroxy-5-methyl-3-((8*S*,10*R*)-7-methylene-10-(prop-1-en-2-yl)-1,4-dioxaspiro[4.6]undecan-8-yl)-4-vinylhexahydro-2*H*-cyclopenta[b]furan-2-one (28). Bu_3SnH

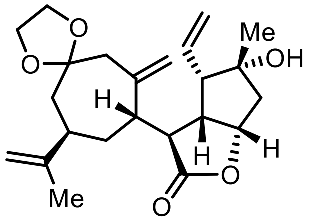

(1.2 mL, 4.46 mmol) was added to a solution of compound **26** (200 mg, 0.462 mmol) and AIBN (22.7 mg, 0.138 mmol) in toluene (15 mL) at ambient temperature. After purging the solution with argon for 15 min, the mixture was stirred at 85 °C for 30 min. The reaction was cooled to ambient temperature and the solvent was removed under high vacuum. The residue was dissolved in CH_3CN (5 mL). DBU (0.7 mL, 4.68 mmol) was added, and the resulting mixture was stirred at 80 °C for 30 min. After cooling to ambient temperature, the solvent was removed under high vacuum and the residue was purified by flash chromatography on silica (hexanes:acetone, 10:1 to 5:1) to afford the title compound as a colorless oil (142 mg, 79% over 2 steps). $[\alpha]_D^{24}$ = –16.3 (c = 0.7, $CHCl_3$); ^{1}H NMR (400 MHz, $CDCl_3$): δ = 5.95 (dt, J = 17.0, 10.0 Hz, 1H), 5.31 (dd, J = 10.2, 2.1 Hz, 1H), 5.17 (ddd, J = 17.0, 2.1, 0.7 Hz, 1H), 5.02 (d, J = 1.3 Hz, 1H), 4.95 (t, J = 1.1 Hz, 1H), 4.92 (dd, J = 7.4, 5.9 Hz, 1H), 4.70 (dt, J = 1.8, 0.9 Hz, 1H), 4.66 (t, J = 1.6 Hz, 1H), 3.98–3.84 (m, 4H), 3.00 (dd, J = 8.9, 2.8 Hz, 1H), 2.82 (ddd, J = 9.8, 7.3, 2.8 Hz, 1H), 2.61 (td, J = 8.3, 5.0 Hz, 1H), 2.55 (d, J = 14.2 Hz, 1H), 2.50 (dtt, J = 11.1, 5.1, 2.1 Hz, 1H), 2.41–2.31 (m, 2H), 2.21 (d, J = 15.0 Hz, 1H), 2.10–2.01 (m, 1H), 1.90–1.78 (m, 2H), 1.72 (dd, J = 1.4, 0.8 Hz, 3H), 1.72–1.66 (m, 1H), 1.35–1.29 (m, 1H), 1.23 (s, 3H) ppm; ^{13}C NMR (101 MHz, $CDCl_3$): δ = 178.7, 150.2, 144.8, 133.4, 120.4, 116.3, 110.4, 109.1, 82.2, 80.2, 64.4, 64.0, 56.7, 47.6, 47.0, 46.4, 45.8, 45.8, 40.7, 37.7, 36.4, 25.7, 21.0 ppm; IR (film) $\tilde{\nu}$ = 3467, 2961, 2880, 1746,

1187, 1111, 1109, 1004, 894 cm^{-1}; MS (ESI): *m*/*z*: 389 [*M*+H$^+$], 411 [*M*+Na$^+$]; HRMS (ESI): *m/z:* calcd. for $C_{23}H_{32}O_5Na$ [*M*+Na$^+$]: 411.21419, found: 411.21475.

(3*S*,3a*S*,4*S*,5*R*,6a*S*)-5-Hydroxy-5-methyl-3-((8*R*,10*R*)-7-methylene-10-(prop-1-en-2-yl)-1,4-dioxaspiro[4.6]undecan-8-yl)-4-vinylhexahydro-2*H*-cyclopenta[b]furan-2-one (13-*epi*-28) :

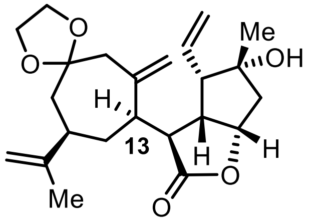

Prepared analogously from **27** as a colorless oil (53.0 mg, 66%). $[\alpha]_D^{20}$ = 15.7 (c = 0.3, $CHCl_3$); ^{1}H NMR (600 MHz, $CDCl_3$): δ = 5.86 (ddd, *J* = 17.1, 10.3, 9.2 Hz, 1H), 5.25 (dd, *J* = 10.3, 2.0 Hz, 1H), 5.16 (ddd, *J* = 17.1, 2.0, 0.8 Hz, 1H), 5.10 (dd, *J* = 1.7, 1.0 Hz, 1H), 5.03 (d, *J* = 1.6 Hz, 1H), 4.87 (dd, *J* = 7.5, 6.5 Hz, 1H), 4.65 (dq, *J* = 1.7, 0.8 Hz, 1H), 4.59 (p, *J* = 1.5 Hz, 1H), 4.05–3.85 (m, 4H), 3.02 (ddd, *J* = 8.8, 7.6, 4.1 Hz, 1H), 3.00 (t, *J* = 3.9 Hz, 1H), 2.77 (ddd, *J* = 12.3, 6.0, 3.8 Hz, 1H), 2.50 (d, *J* = 13.2 Hz, 1H), 2.37 (t, *J* = 9.0 Hz, 1H), 2.29 (ddd, *J* = 13.2, 2.2, 0.8 Hz, 1H), 2.16 (d, *J* = 15.1 Hz, 1H), 2.07–2.01 (m, 1H), 1.88 (ddd, *J* = 13.9, 12.3, 9.6 Hz, 1H), 1.89–1.82 (m, 1H), 1.80–1.77 (m, 2H), 1.72 (dd, *J* = 13.9, 6.1 Hz, 1H), 1.67 (dd, *J* = 1.5, 0.8 Hz, 3H), 1.23 (s, 3H), 1.21 (d, *J* = 1.2 Hz, 1H) ppm; ^{13}C NMR (151 MHz, $CDCl_3$): δ = 178.8, 150.7, 143.1, 133.0, 120.0, 119.2, 109.2, 108.6, 82.5, 80.3, 77.1, 77.0, 76.8, 64.6, 64.2, 56.1, 48.5, 48.0, 47.5, 47.0, 46.2, 42.9, 40.4, 35.4, 25.6, 20.1 ppm; IR (film) $\tilde{\nu}$ = 3469, 2963, 2927, 1751, 1374, 1270, 1198, 1104, 998, 931 cm^{-1}; MS (ESI): *m*/*z*: 389 [M+H$^+$], 411 [M+ Na$^+$]; HRMS (ESI): *m/z:* calcd. for $C_{23}H_{32}O_5Na$ [*M*+Na$^+$]: 411.21419, found: 411.21419.

(2a*S*,2a1*S*,4*R*,4a*S*,9*R*,10a*S*,10b*S*)-4-Hydroxy-4-methyl-9-(prop-1-en-2-yl)-2a1,3,4,4a,6,8,9,10,10a,10b-decahydrospiro[cyclohepta[5,6]indeno[1,7-bc]furan-7,2'-

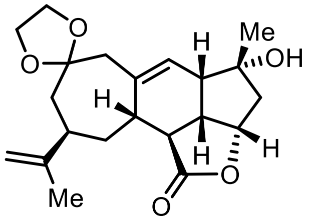

[1,3]dioxolan]-1(2a*H*)-one (29). Hoveyda-Grubbs second-generation catalyst **21** (21.0 mg, 33.5 μmol) was added to a solution of diene **28** (130 mg, 0.335 mmol) in toluene (111 mL) at ambient temperature. The mixture was stirred at 100 °C for 12 h. After reaching ambient temperature, the solvent was removed under high vacuum, and the crude material was purified by flash chromatography on silica gel (hexanes:acetone, 10:1 to 3:1) to afford the title compound as a colorless oil (93 mg, 77%). $[\alpha]_D^{20}$ = –80.0 (c = 0.2, $CHCl_3$); ^{1}H NMR (400 MHz, $CDCl_3$): δ = 5.53 (dd, *J* = 5.2, 2.4 Hz, 1H), 4.90 (ddd, *J* = 8.4, 6.7, 5.2 Hz, 1H), 4.64 (q, *J* = 1.4 Hz, 2H), 4.06–3.84 (m, 4H), 3.59 (dd, *J* = 13.6, 11.0 Hz, 1H), 2.77–2.61 (m, 5H), 2.55 (tt, *J* = 5.3, 1.5 Hz, 1H), 2.42–2.31 (m, 3H), 1.97–1.88 (m, 1H), 1.86–1.73 (m, 3H), 1.71 (t, *J* = 1.2 Hz, 3H), 1.40

(s, 3H) ppm; ^{13}C NMR (101 MHz, $CDCl_3$): δ = 175.7, 150.3, 143.5, 124.6, 109.0, 108.4, 81.3, 80.0, 64.6, 64.4, 49.1, 48.6, 47.4, 45.7, 43.4, 38.6, 38.1, 36.2, 32.3, 31.5, 20.9 ppm; IR (film) $\tilde{\nu}$ = 2932, 2892, 1098, 1069, 1028, 974, 922 cm^{-1}; MS (ESI): *m*/*z*: 361 [*M*+H^+], 383 [*M*+Na^+]; HRMS (ESI): *m/z:* calcd. for $C_{21}H_{28}O_5Na$ [*M*+Na^+]: 383.18289, found: 383.18307.

Isomer 12-*epi*-1. Montmorillonite K-10 (70 mg) was added to a solution of alkene **29** (18.1 mg, 50 *µ*mol) in CH_2Cl_2 (1.6 mL) at ambient temperature. After stirring for 2 h at this temperature, the mixture was filtered through a pad of Celite®, which was rinsed with EtOAc (10 mL). After evaporation of the combined filtrates, the crude material was dissolved in toluene (1.3 mL). MS 4 Å (20 mg) and $VO(acac)_2$ (1.4 mg, 0.0053 mmol) were successively added. After stirring of the suspension for 10 min, *t*BuOOH (ca. 5.5 M in decane, 0.03 mL, 0.165 mmol) was added at 0 °C. After another 5 min, the cooling bath was removed and the mixture stirred at ambient temperature for 6 h before it was filtered through a pad of Celite®. The reaction was quenched with sat. aq. $Na_2S_2O_3$ (2 mL), the aqueous phase was extracted with EtOAc (4 x 5 mL), the combined extracts were washed with brine (1 mL), dried over anhydrous Na_2SO_4, filtered and evaporated. The residue was used in the next step without purification.

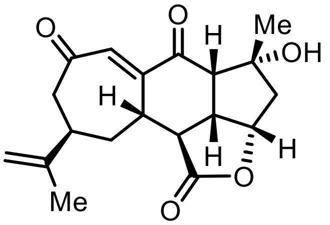

Et_3N (0.02 mL, 0.144 mmol) was added to a solution of the crude product in CH_2Cl_2 (0.8 mL). After stirring for 30 min at ambient temperature, the solvent was removed under high vacuum. The residue was purified by flash chromatography on silica gel (hexanes:acetone, 4:1 to 2:1) to afford compound **31** as a white solid material (9.2 mg, 55% over 3 steps). $[\alpha]_{D}^{20}$ = 26.2 (c = 0.5, $CHCl_3$); ^{1}H NMR (400 MHz, $CDCl_3$): δ = 6.01 (t, *J* = 1.7 Hz, 1H), 4.82–4.57 (m, 4H), 4.05 (dd, *J* = 14.4, 11.6 Hz, 1H), 2.94 (tdd, *J* = 11.8, 5.7, 2.1 Hz, 1H), 2.71–2.53 (m, 6H), 2.42 (dd, *J* = 12.9, 9.4 Hz, 1H), 2.30 (dd, *J* = 12.9, 6.9 Hz, 1H), 2.20–2.09 (m, 2H), 1.84–1.70 (m, 1H), 1.77 (s, 3H), 1.40 (d, *J* = 0.9 Hz, 3H) ppm; ^{13}C NMR (101 MHz, $CDCl_3$): δ = 204.2, 174.6, 153.7, 146.9, 130.8, 110.9, 82.7, 77.8, 75.7, 46.8, 46.4, 46.2, 44.8, 40.2, 38.8, 37.0, 33.5, 32.5, 20.1 ppm. IR (film) $\tilde{\nu}$ = 3363, 2970, 2925, 1781, 1649, 1420, 1378, 1259, 1010, 968, 755 cm^{-1}; MS (ESI): *m*/*z*: 333 [M+H^+], 355 [M+Na^+]; HRMS (ESI): *m/z:* calcd. for $C_{19}H_{24}O_5Na$ [*M*+ Na^+]: 355.15159, found: 355.15167.

IBX (31.0 mg, 0.11 mmol) was added to a solution of **31** in CH_3CN (1.4 mL) at ambient temperature. The mixture was stirred at 50 °C for 1.5 h and then cooled to ambiemt temperature,

before it was passed through a pad of silica gel, which was rinsed with EtOAc (10 mL). The combined filtrates were evaporated, the residue was dissolved in CH_2Cl_2 (3 mL), and the solution passed through a pad of cotton to provide the title compound as a white solid material (7.5 mg, 82%). $[\alpha]_D^{20}$ = –5.2 (c = 0.8, $CHCl_3$); ^{1}H NMR (600 MHz, $CDCl_3$): δ = 6.70 (dd, *J* = 2.7, 1.8 Hz, 1H), 5.01 (ddd, *J* = 9.4, 7.0, 4.7 Hz, 1H), 4.83 (p, *J* = 1.4 Hz, 1H), 4.83 (dt, *J* = 2.1, 0.8 Hz, 1H), 3.39 (dd, *J* = 13.5, 12.3 Hz, 1H), 3.18 (dddd, *J* = 12.2, 10.9, 3.8, 2.7 Hz, 1H), 3.08 (dt, *J* = 13.5, 6.8 Hz, 1H), 2.86 (ddd, *J* = 14.3, 6.2, 3.9 Hz, 1H), 2.81 (d, *J* = 6.6 Hz, 1H), 2.78 (dd, *J* = 14.7, 11.6 Hz, 1H), 2.69 (ddd, *J* = 14.9, 3.4, 1.8 Hz, 1H), 2.59 (dtd, *J* = 11.6, 7.5, 6.2, 3.4 Hz, 1H), 2.52 (dd, *J* = 16.3, 9.5 Hz, 1H), 2.42 (dd, *J* = 16.3, 4.7 Hz, 1H), 2.24 (s, 1H), 1.82 (t, *J* = 1.0 Hz, 3H), 1.69 (ddd, *J* = 14.3, 10.9, 7.5 Hz, 1H), 1.62 (s, 3H) ppm; ^{13}C NMR (151 MHz, $CDCl_3$): δ = 202.9, 200.4, 174.1, 150.1, 146.5, 136.7, 111.5, 84.4, 80.0, 58.9, 48.1, 47.1, 46.8, 41.8, 38.5, 36.3, 33.7, 33.0, 20.9 ppm; IR (film) $\tilde{\nu}$ = 3468, 2969, 2929, 1771, 1664, 1363, 1169, 1113, 993, 897 cm^{-1}; MS (ESI): *m/z*: 353 [M+ Na$^+$]; HRMS (ESI): *m/z:* calcd. for $C_{19}H_{22}O_5Na$ [*M*+ Na$^+$]: 353.13594, found: 353.13561.

Nominal Scabrolide B (1). Montmorillonite K-10 (100 mg) was added to a solution of alkene **29** (16 mg, 0.044 mmol) in CH_2Cl_2 (1.2 mL) at ambient temperature. After stirring for 2 h, the mixture was filtered through a pad of Celite®, which was carefully rinsed with EtOAc (5 mL). The combined filtrates were evaporated and the residue redissolved in toluene (1.1 mL). 4 Å MS (20 mg) and $VO(acac)_2$ (2.4 mg, 9 μmol) were added at ambient temperature. After 10 min, the mixture was cooled to 0 °C before *t*BuOOH (ca. 5.5 M in decane, 0.04 mL, 0.22 mmol) was introduced. After 5 min, the cooling bath was removed and the mixture stirred at ambient temperature for 6 h before it was filtered through a pad of Celite®. The reaction was quenched with sat. aq. $Na_2S_2O_3$ (2 mL) before the mixture was extracted with EtOAc (4 x 5 mL). The combined organic layers were washed with brine (1 mL), dried over anhydrous Na_2SO_4, filtered and evaporated. The residue was used in the next step without further purification.

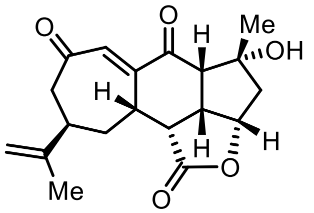

Et_3N (0.03 mL, 0.22 mmol) was added to a solution of the crude product in MeOH (0.44 mL). After stirring for 30 min at ambient temperature, the solvent was removed under high vacuum. The residue was purified by flash chromatography on silica gel (hexanes:acetone, 4:1 to 2:1) to afford a mixture of compounds **32** and **33**, which was subjected to oxidation without delay.

IBX (33.7 mg, 0.12 mmol) was added to a solution of this material in CH_3CN (1.0 mL) and the resulting mixture was stirred at ambient temperature for 1 h and at 50 °C for another 1.5 h. After reaching ambient temperature, the suspension was filtered through a pad of silica, which was carefully rinsed with EtOAc (10 mL). The combined filtrates were evaporated and the residue was purified by preparative TLC on silica gel (CH_2Cl_2:MeOH, 20:1) to afford nominal scabrolide B (**1**) (5.0 mg, 34% over 4 steps) and transesterification product **34** (5.1 mg, 35% over 4 steps) as white solid materials each. Analytical data of **1**: $[\alpha]_{D}^{20}$ = 20.0 (c = 0.18, $CHCl_3$); for the ^{1}H NMR and ^{13}C NMR data, see Tables S3 and S4; the spectra are obscured by partial enolization of the C6-carbonyl group (a detailed analysis is provided below); IR (film) ν = 2919, 2850, 1763, 1680, 1149, 1083, 1006, 894 cm^{-1}; MS (ESI)*: m/z*: 348 [*M*+NH_4^+], 353 [M+ Na^+]; HRMS (ESI): *m/z:* calcd. for $C_{19}H_{22}O_5Na$ [*M*+ Na^+]: 353.13594, found: 353.13625.

The compound is rather unstable and shows notable decomposition already after 18 h when kept in $CDCl_3$ solution (see copies of spectra).

Isomer 34: Trace impurities (≤ 5%) could not be removed by chromatographic means; $[\alpha]_{D}^{20}$ = –64.2 (c = 0.6, $CHCl_3$^{1}H NMR (400 MHz, $CDCl_3$): δ = 6.20–6.10 (m, 1H), 5.45 (d, *J* = 1.5 Hz, 1H), 4.80 (t, *J* = 1.4 Hz, 1H), 4.75 (q, *J* = 1.0 Hz, 1H), 3.06 (ddd, *J* = 3.3, 2.2, 0.8 Hz, 1H), 3.01 (ddd, *J* = 11.8, 5.4, 2.6 Hz, 1H), 2.96 (ddt, *J* = 10.0, 3.4, 1.0 Hz, 1H), 2.70–2.64 (m, 3H), 2.60–2.47 (m, 3H), 2.15 (s, 1H), 2.00 (ddd, *J* = 14.0, 7.3, 5.5 Hz, 1H), 1.82–1.76 (m, 1H), 1.74 (dd, *J* = 1.4, 0.7 Hz, 3H), 1.43 (d, *J* = 1.0 Hz, 3H) ppm; ^{13}C NMR (151 MHz, $CDCl_3$): δ = 210.6, 201.3, 170.7, 151.2, 146.3, 128.7, 111.3, 79.9, 74.6, 53.3, 50.6, 48.9, 46.3, 45.9, 38.4, 37.7, 34.8, 31.2, 20.4 ppm; IR (film) ν = 3488, 2924, 1699, 1618, 1239, 1031, 905, 704 cm^{-1}; MS (ESI)*: m/z*: 353 [M+ Na^+]; HRMS (ESI): *m/z:* calcd. for $C_{19}H_{22}O_5Na$ [*M*+ Na^+]: 353.13594, found: 353.13562.

Scabrolide A (2). K_2CO_3 (4.2 mg, 0.030 mmol) was added to a solution of nominal scabrolide B (**1**) (5.0 mg, 0.015 mmol) in CH_3CN (0.38 mL) at ambient temperature. The mixture was sonicated at 40 °C for 3 h before it was filtered through a pad of silica to remove the remaining solid material. The filtrate was evaporated and the residue purified by flash chromatography on slica (hexane/acetone, 2:1 to 1:1) to afford Scabrolide A (4.9 mg, 98%) as a white solid material. Preparative HPLC condition: YMC Triart-C18, 5 µm, 150 mm × 10 mm, MeOH/H_2O = 40:60, 4.7 mL/min, λ = 220 nm, t = 7.5 min. $[\alpha]_{D}^{20}$ = –172.5 (c = 0.04, $CHCl_3$); for the ^{1}H NMR and ^{13}C NMR

data, see Tables S1 and S2; IR (film) ν = 2950, 2930, 1761, 1654, 1375, 1178, 1118, 902, 671 cm^{-1}; MS (ESI): *m*/*z*: 331 [*M*+H$^+$], 353 [M+ Na$^+$]; HRMS (ESI): *m/z:* calcd. for $C_{19}H_{22}O_5Na$ [*M*+ Na$^+$]: 353.13594, found: 353.13632.

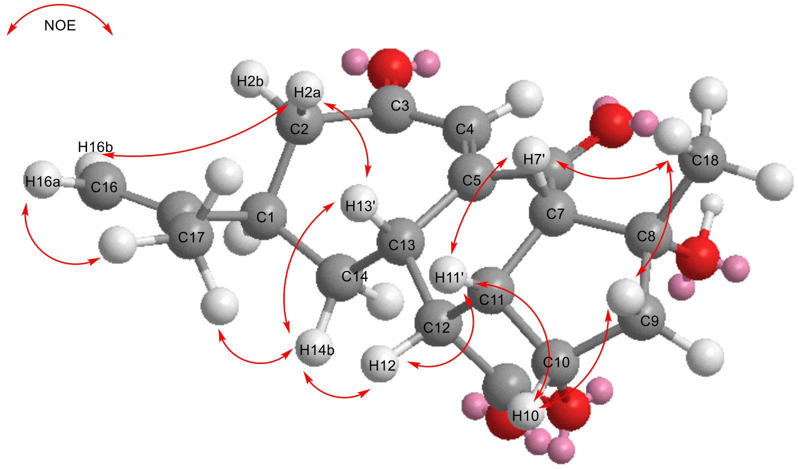

Nominal Scabrolide B (**1**)

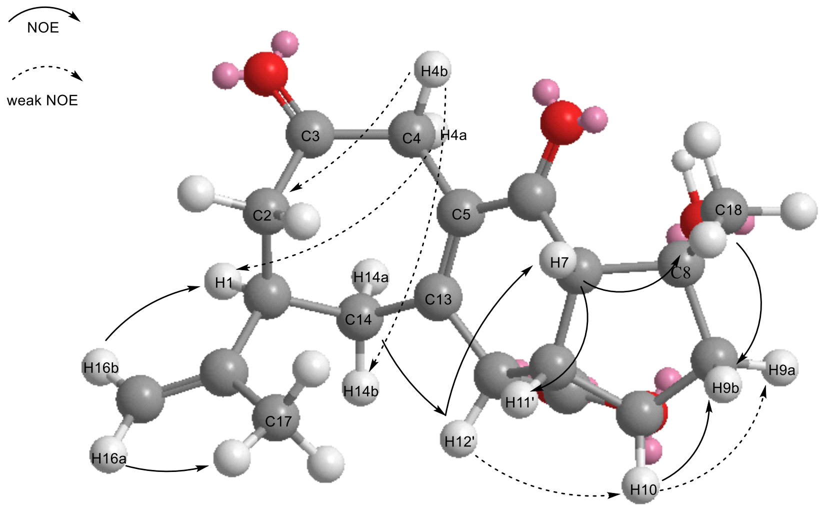

Scabrolide A (**2**)

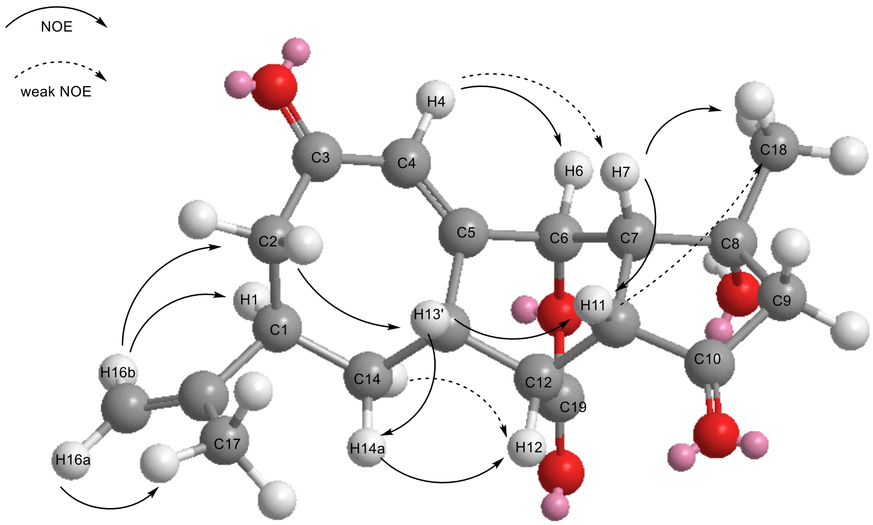

Transesterification product **34**

Figure S6. Characteristic NOE Interactions of Key Compounds

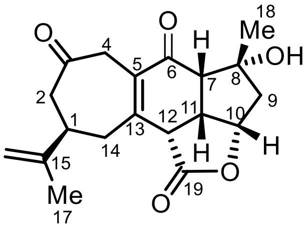

Table S1. Comparison of the ^{1}H NMR data of synthetic Scabrolide A (**2**) with those of the natural product reported in the literature; numbering scheme as shown in the insert

Position	Reported Data[5]	Synthetic Sample
	δ (ppm), *J* (Hz)	δ (ppm), *J* (Hz)
1	3.07 (q, 6.4)	3.11-3.07, m
2	2.63, m	2.66-2.58, m
3		
4a	3.42 (d, 17.2)	3.43 (dd, 17.3, 1.1)
4b	3.70 (d, 17.2)	3.70 (d, 17.2)
5		
6		
7	2.62 (d, 10.0)	2.60 (d, 10.5)
8		
9a	2.30 (d, 15.2)	2.30 (d, 15.0)
9b	1.92 (dd, 15.2, 5.6)	1.93 (dd, 15.0, 5.6)
10	5.11 (t, 6.4)	5.11 (dd, 7.2, 5.5)
11a	3.62 (dd, 11.2, 6.4)	3.64-3.58, m
12a	3.51 (d, 11.2)	3.51 (d, 11.3)
13		
14	2.88, m	2.93-2.83
15		
16a	4.85, s	4.85 (q, 1.4)
16b	4.83, s	4.84 (t, 1.1)
17	1.82, s	1.83, s
18	1.49, s	1.50, s
19		

Table S2. Comparison of the ^{13}C NMR data of synthetic Scabrolide A (**2**) with those of the natural product reported in the literature; numbering scheme as shown in the insert

Position	Reported Data[5]	Synthetic Sample	Δδ
	δ (ppm)	δ (ppm)	
1	41.6	41.6	0
2	46.3	46.3	0
3	208.3	208.1	0.2
4	39.5	39.5	0
5	132.7	132.8	–0.1
6	193.1	193.0	0.1
7	54.5	54.5	0
8	82.9	83.0	–0.1
9	47.4	47.5	–0.1
10	82.2	82.1	0.1
11	40.9	40.9	0
12	44.6	44.6	0
13	151.7	151.7	0
14	37.2	37.2	0
15	147.1	147.1	0
16	110.8	110.8	0
17	21.3	21.3	0
18	26.1	26.2	–0.1
19	173.7	173.5	0.2

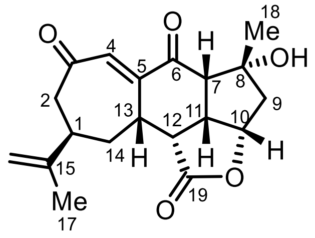

Nominal Scabrolide B (**1**)

Table S3. Comparison of the ^{1}H NMR data of synthetic **1** with those of natural Scabrolide B as reported in the literature; numbering scheme as shown in the insert

Position	Reported Data[5]	Synthetic Sample
	δ (ppm), *J* (Hz)	δ (ppm), *J* (Hz)
1	2.81, m	2.74
2a	2.90 (ddd, 16.0, 4.0, 2.5)	2.81
2b	2.60 (dd, 16.0, 6.0)	2.75
3		
4	6.34 (d, 3.0)	6.76
5		
6		
7	2.93 (d, 7.2)	2.70 (ddd, 8.3, 6.8, 1.3)
8		
9a	3.36 (dd, 16.0, 2.5)	2.25
9b	2.21 (ddd, 16.0, 9.0, 1.5)	2.25
10	4.98 (td, 9.0, 2.5)	4.91
11	3.45 (t, 10.5)	3.58
12	3.14 (td, 10.5, 7.8)	3.02
13	2.75, m	3.21
14a	1.71 (tdd, 10.0, 5.0, 1.2)	2.72
14b	3.30 (tdd, 10.0, 4.0, 2.5)	2.28
15		
16a	4.95, s	4.93
16b	4.72, s	4.72
17	1.83, s	1.83
18	1.63, s	1.39, s
19		

Table S4. Comparison of the ^{13}C NMR data of synthetic **1** with those of natural Scabrolide B as reported in the literature; numbering scheme as shown in the insert

Position	Reported Data[5]	Synthetic Sample	Δ (R-S)
	δ (ppm)	δ (ppm)	δ (ppm)
1	38.9	38.3	0.6
2	45.0	46.9	–1.9
3	202.2	202.1	0.1
4	130.5	135.6	–5.1
5	150.8	148.5	2.3
6	202.5	202.9	–0.4
7	62.4	53.6	8.8
8	81.3	81.2	0.1
9	47.4	45.7	1.7
10	79.5	81.0	–1.5
11	45.3	43.3	2.3
12	45.3	43.4	1.9
13	41.6	36.5	5.1
14	30.5	35.3	–4.8
15	146.4	145.7	0.7
16	112.7	113.4	–0.7
17	21.8	22.6	–0.8
18	30.0	28.9	1.1
19	175.9	174.4	1.5

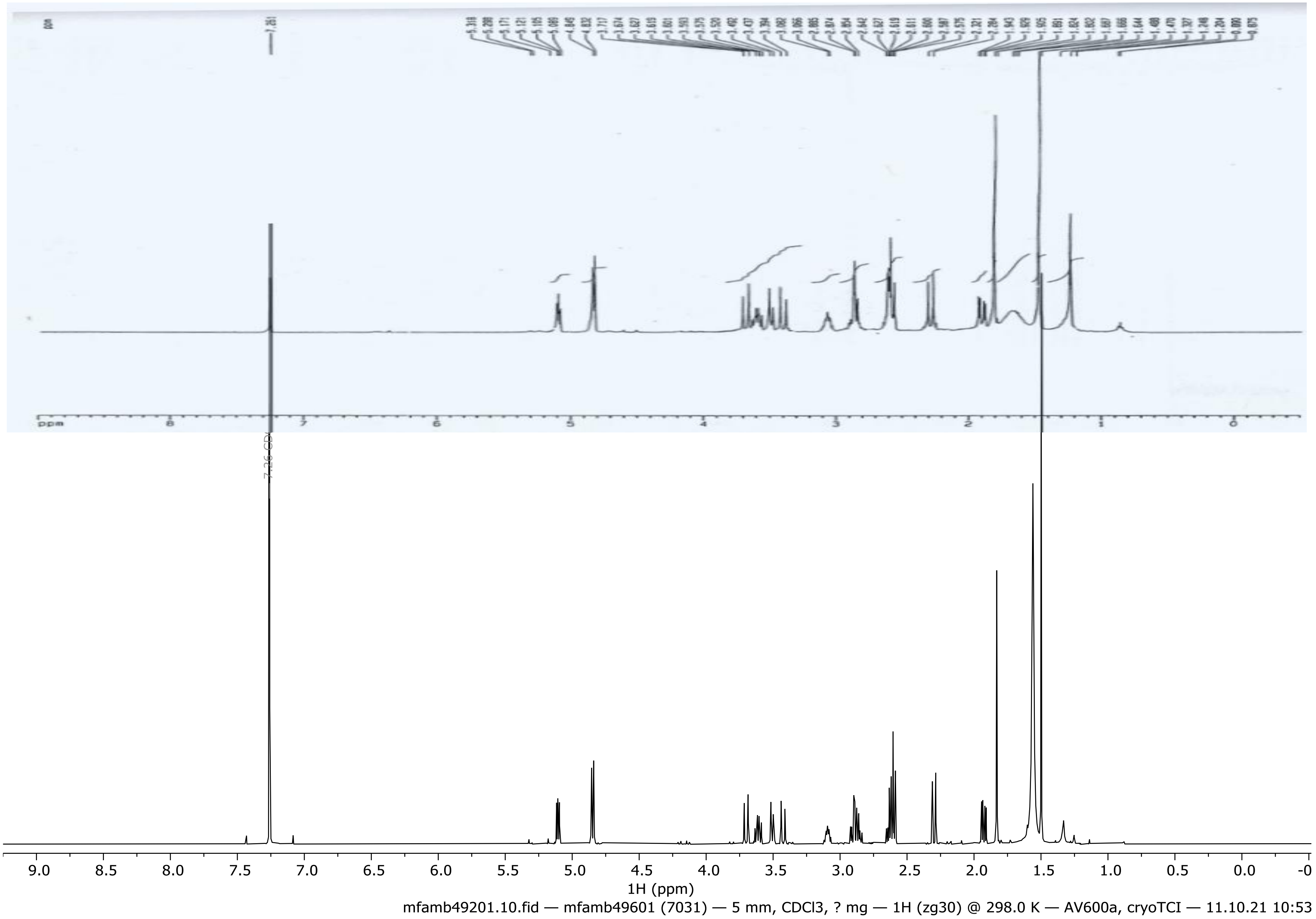

^{1}H NMR: Top: ^{1}H NMR of Scabrolide A (ref. 5); bottom: synthetic Scabrolide A

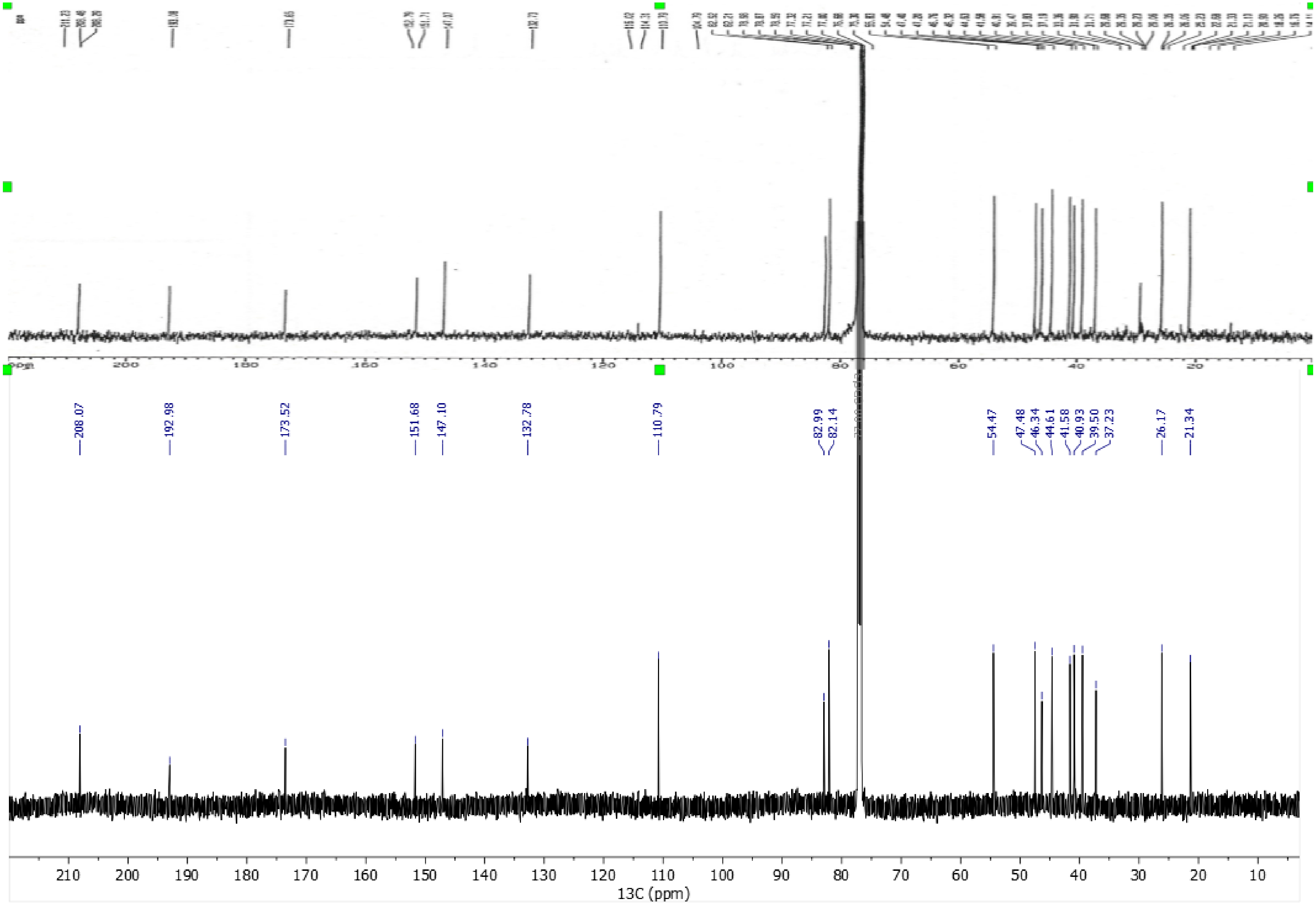

^{13}C NMR: Top: ^{13}C NMR of Scabrolide A (ref. 5); bottom: synthetic Scabrolide A

^{1}H NMR Spectrum of 9 (400 MHz, $CDCl_3$)

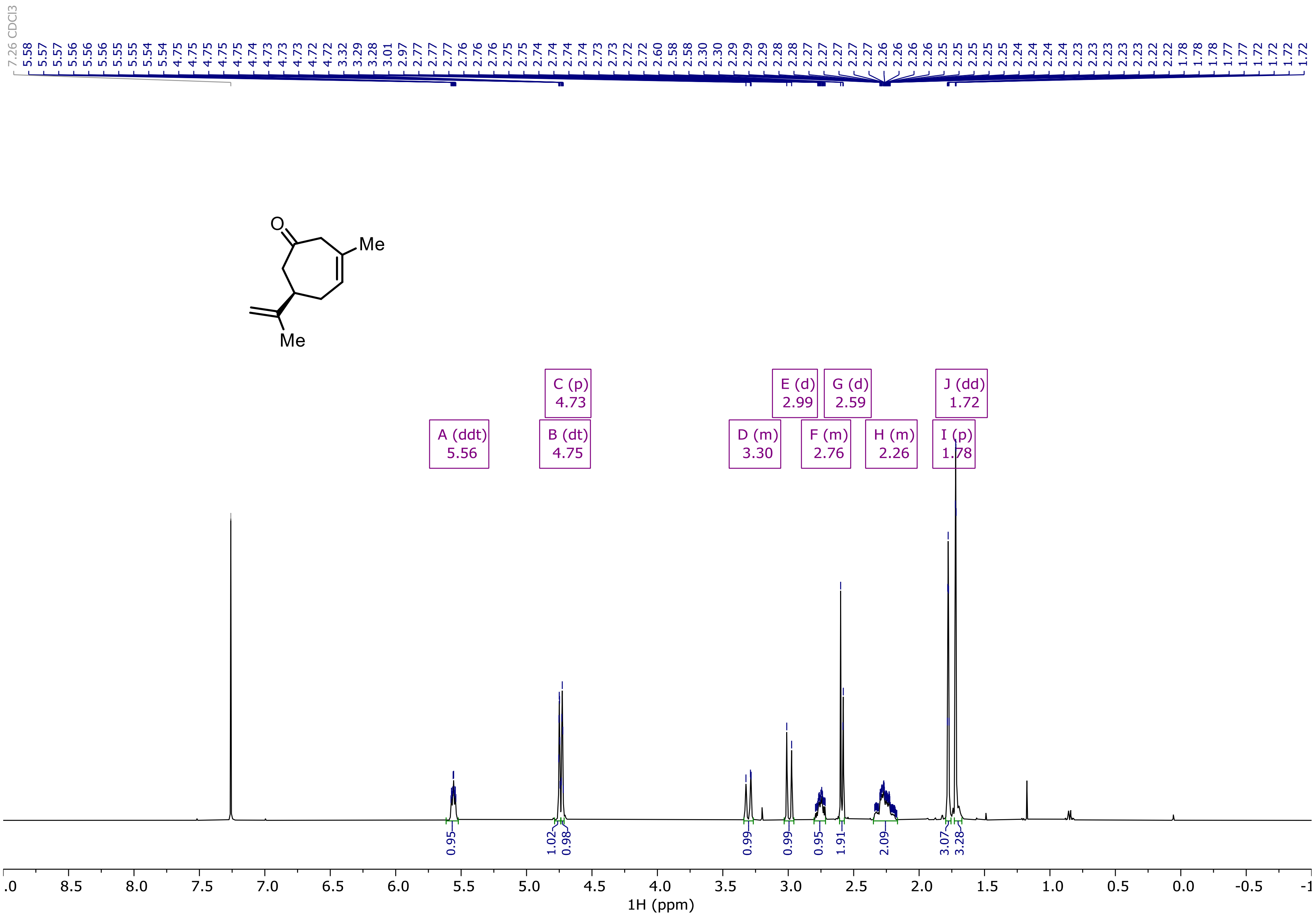

^{13}C NMR Spectrum of 9 (101 MHz, $CDCl_3$)

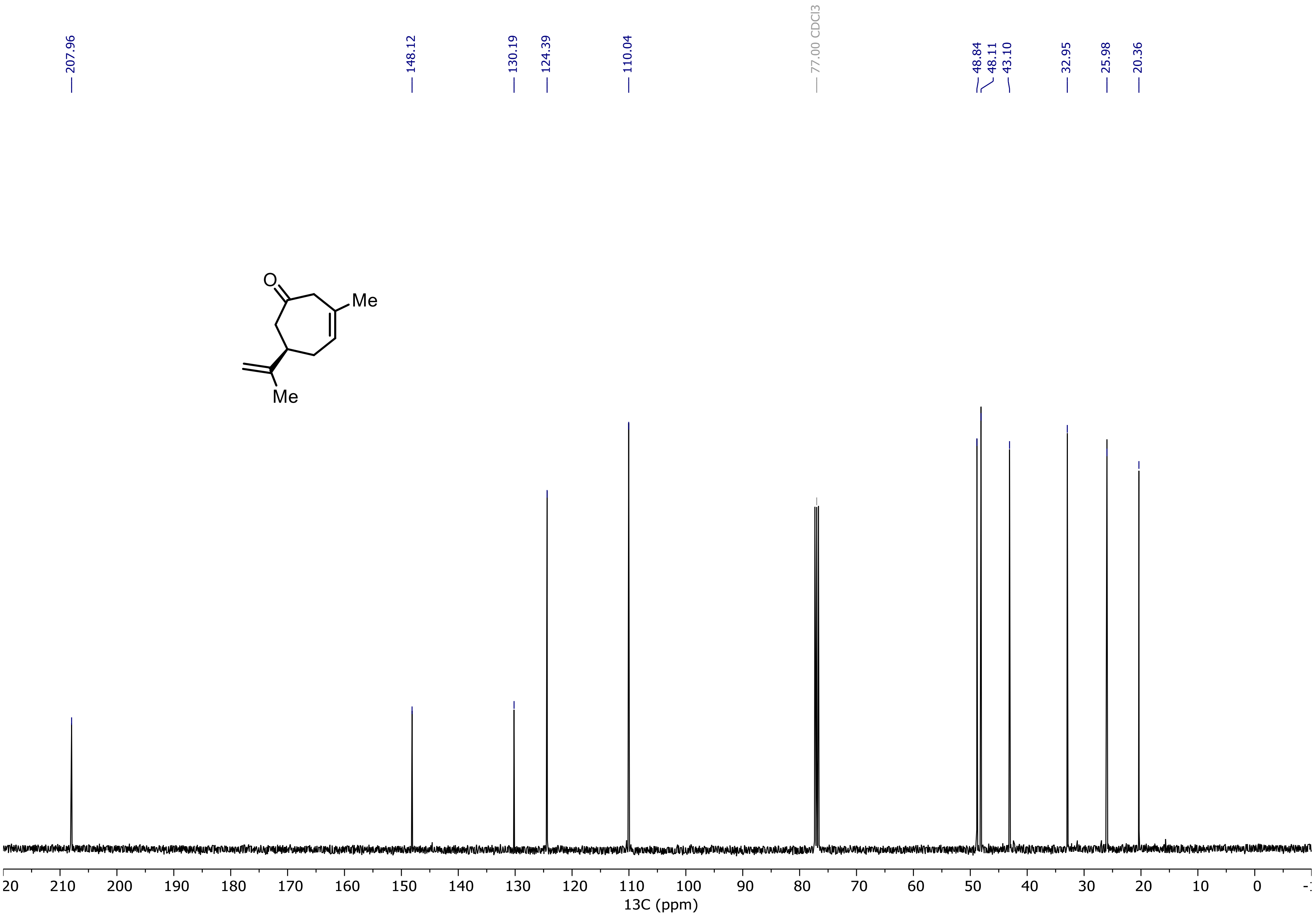

^{1}H NMR Spectrum of 10 (400 MHz, $CDCl_3$)

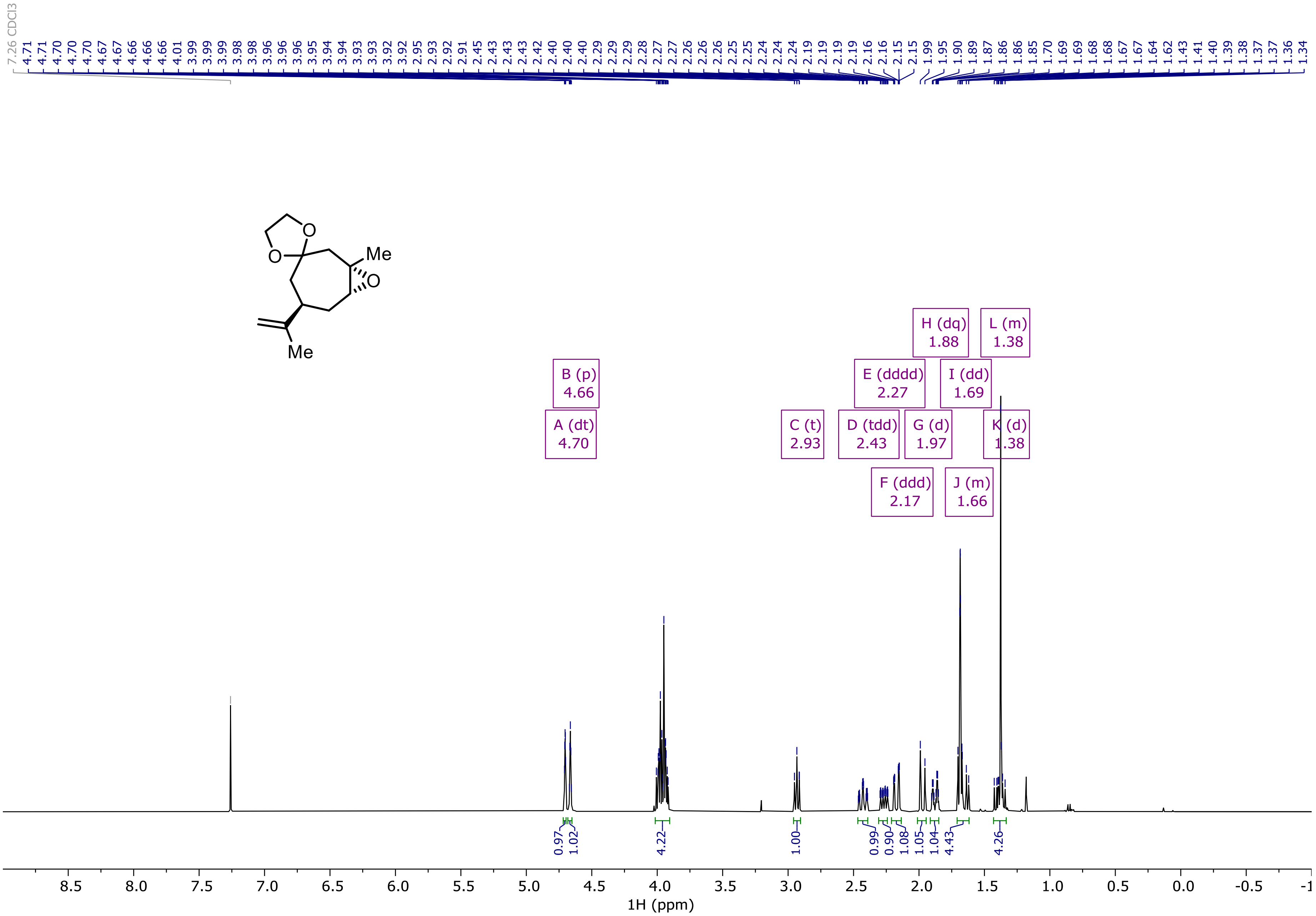

^{13}C NMR Spectrum of 10 (101 MHz, $CDCl_3$)

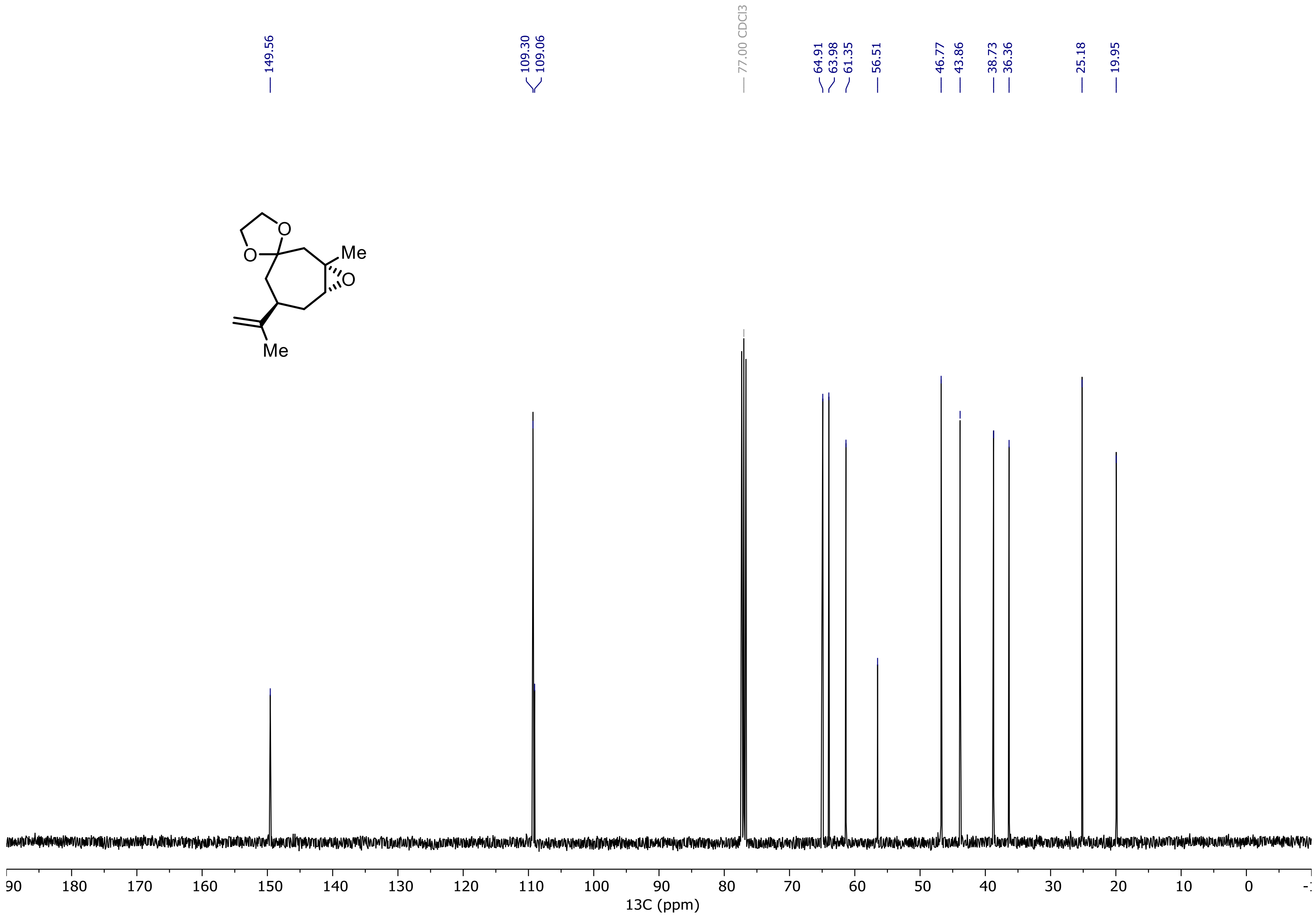

^{1}H NMR Spectrum of 11 (400 MHz, $CDCl_3$)

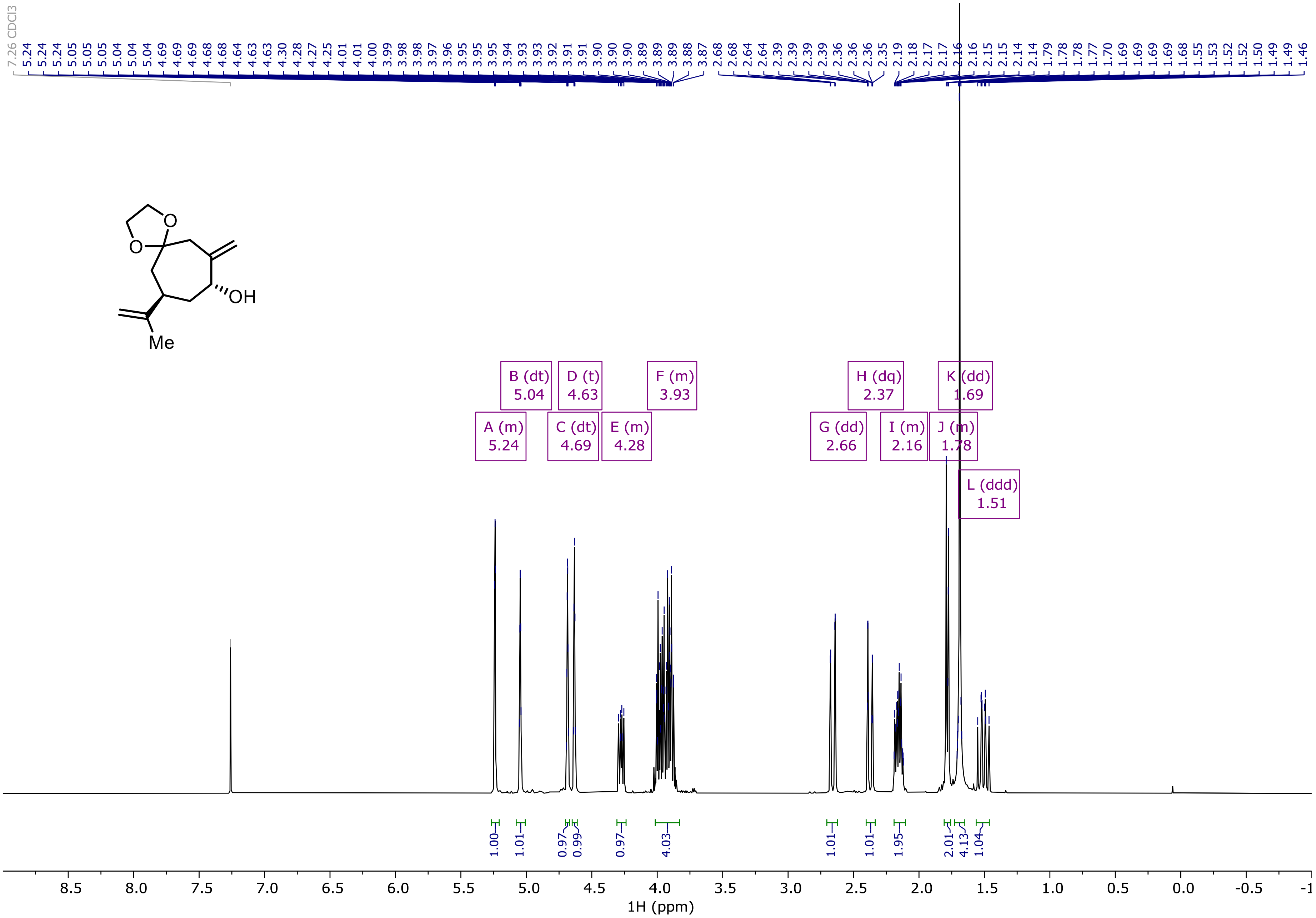

^{13}C NMR Spectrum of 11 (101 MHz, $CDCl_3$)

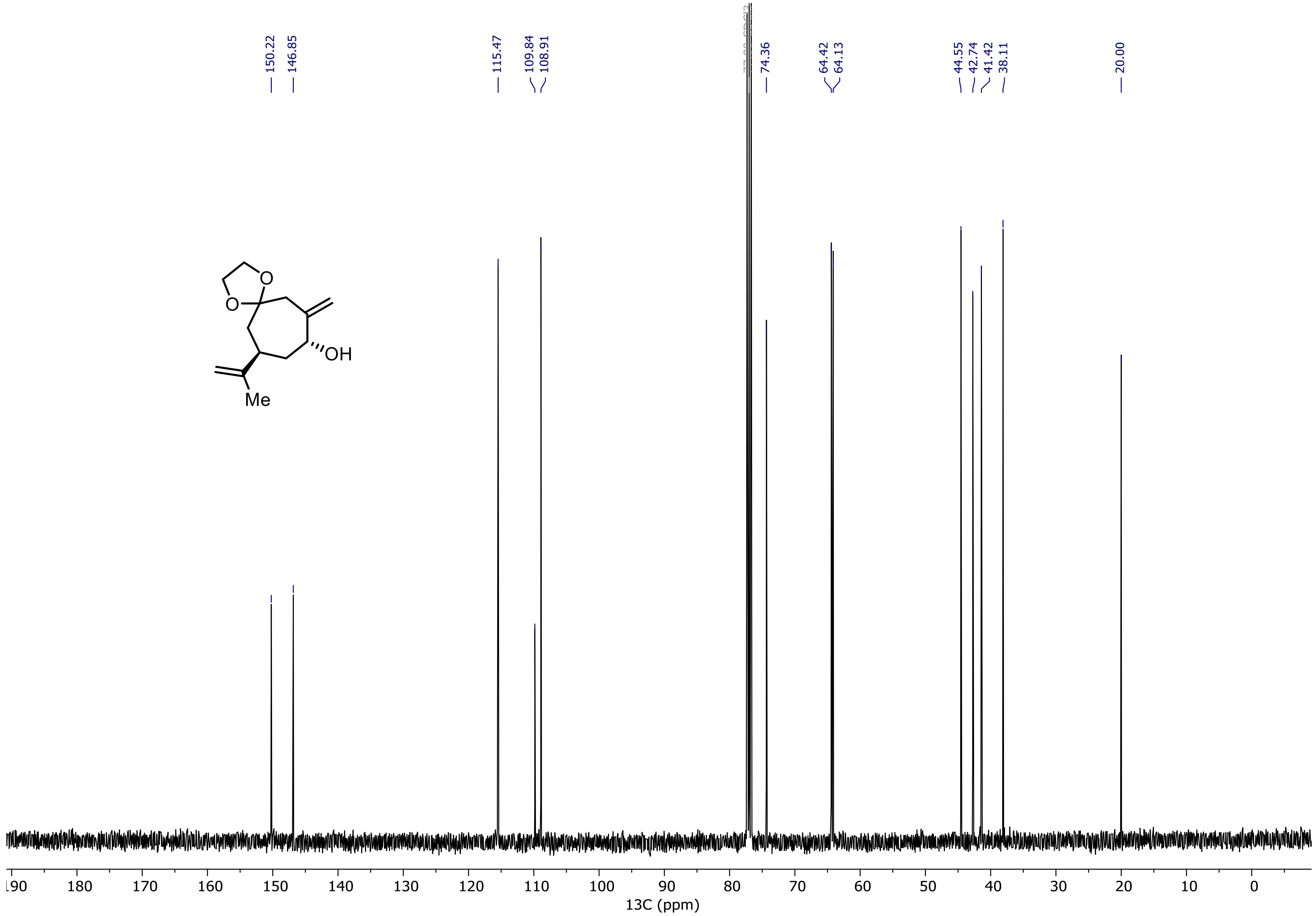

^{1}H NMR Spectrum of 23a (400 MHz, $CDCl_3$)

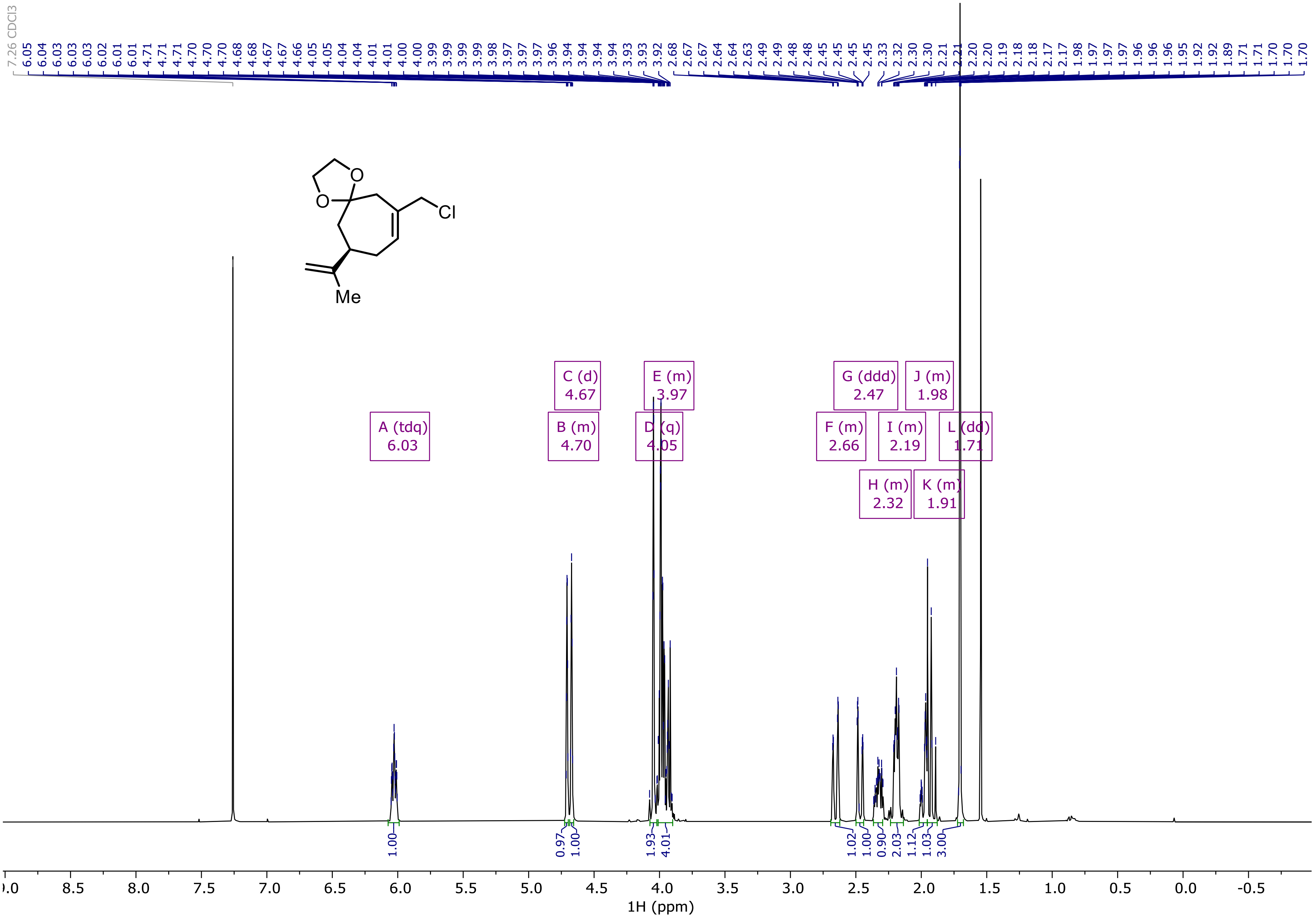

^{13}C NMR Spectrum of 23a (101 MHz, $CDCl_3$)

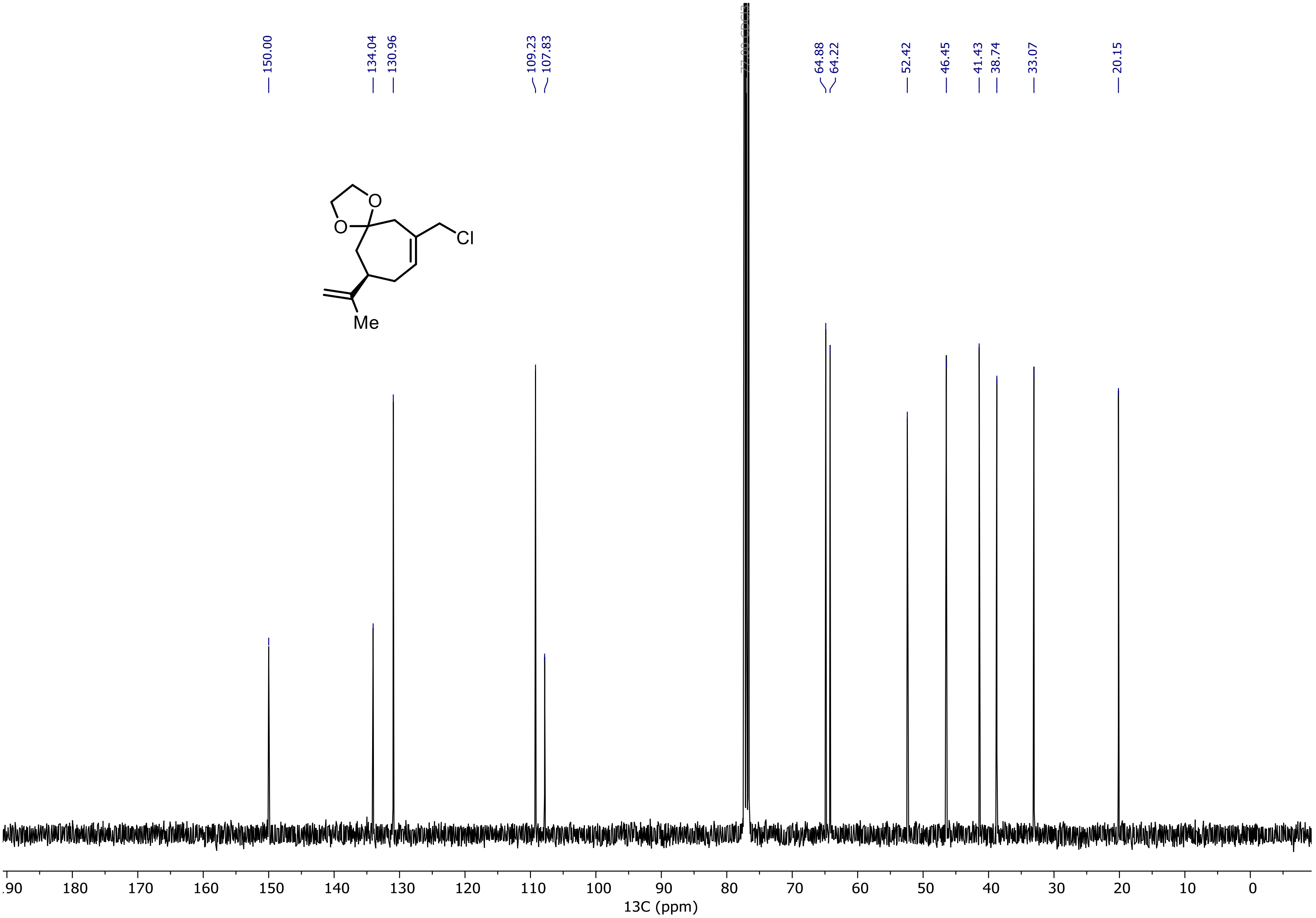

^{1}H NMR Spectrum of 12 (400 MHz, $CDCl_3$)

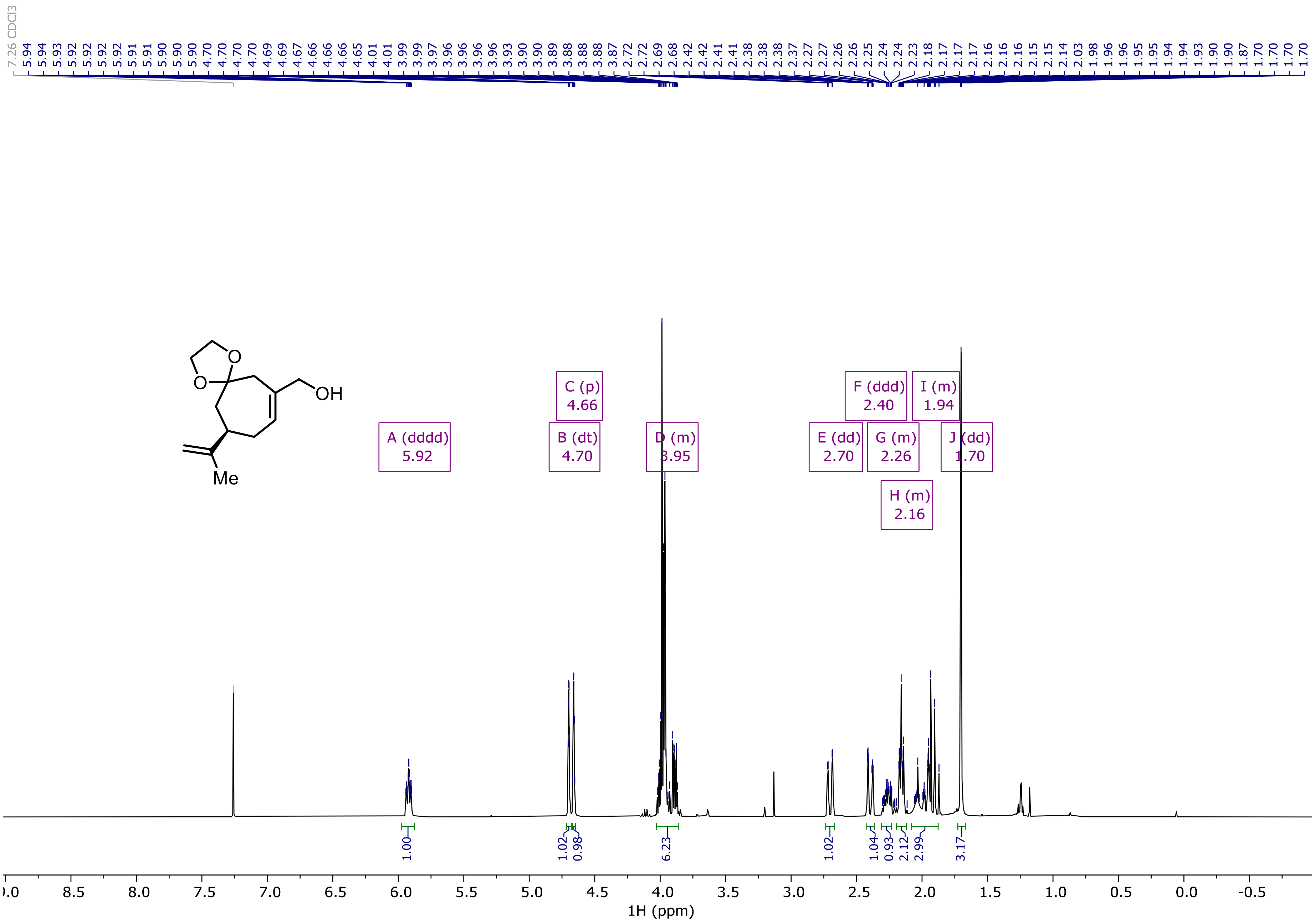

^{13}C NMR Spectrum of 12 (101 MHz, $CDCl_3$)

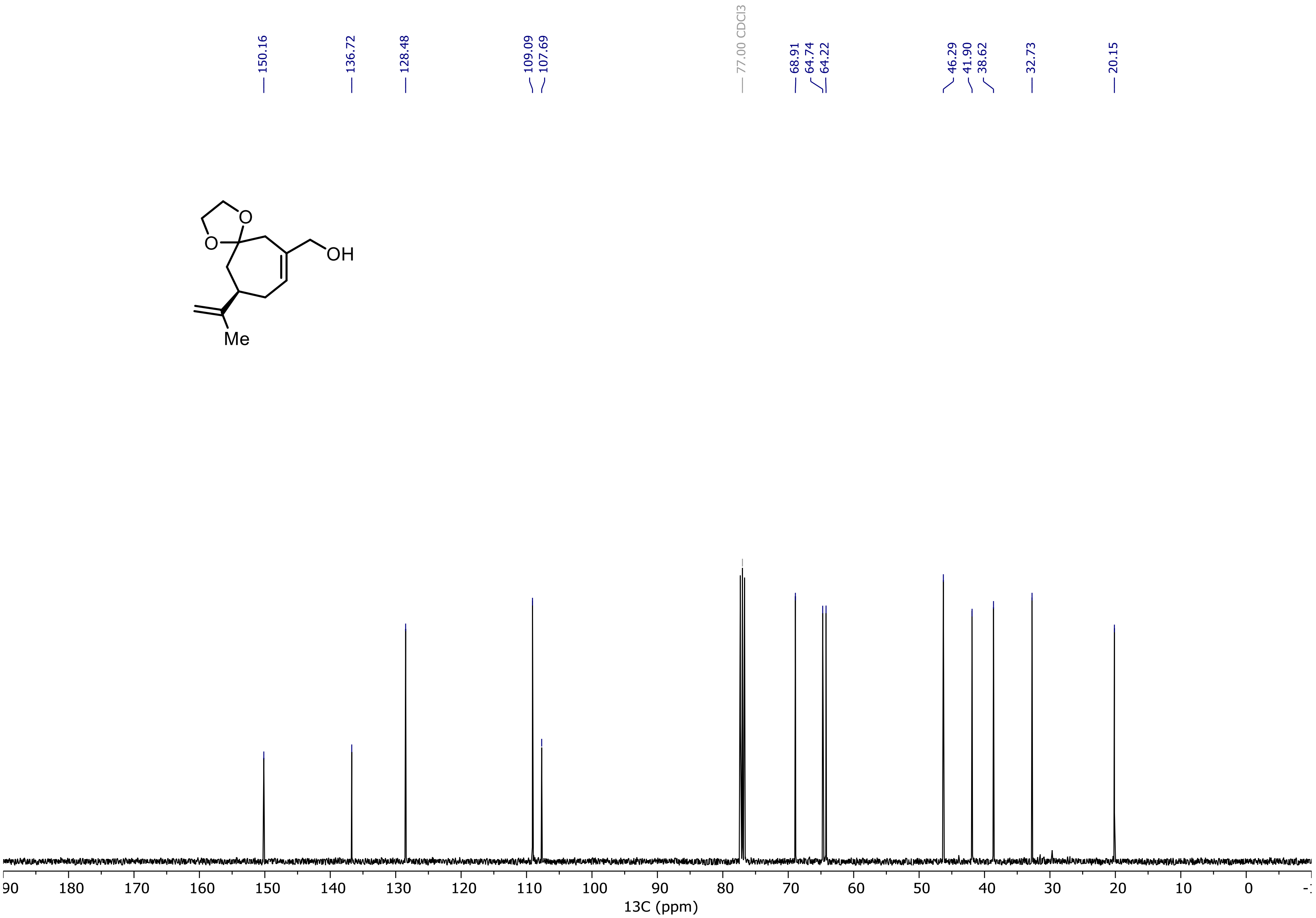

^{1}H NMR Spectrum of 15 (400 MHz, $CDCl_3$)

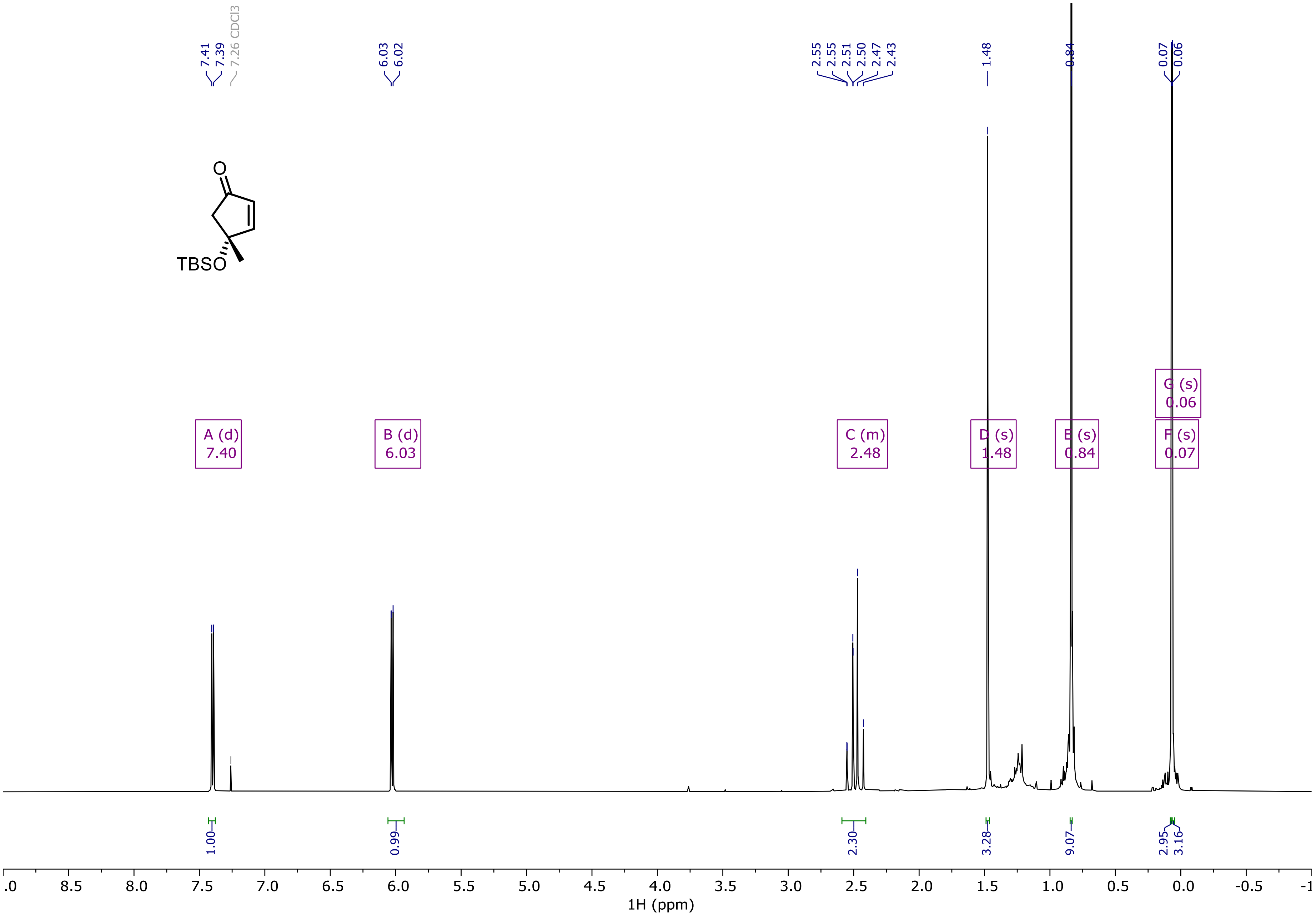

^{13}C NMR Spectrum of 15 (101 MHz, $CDCl_3$)

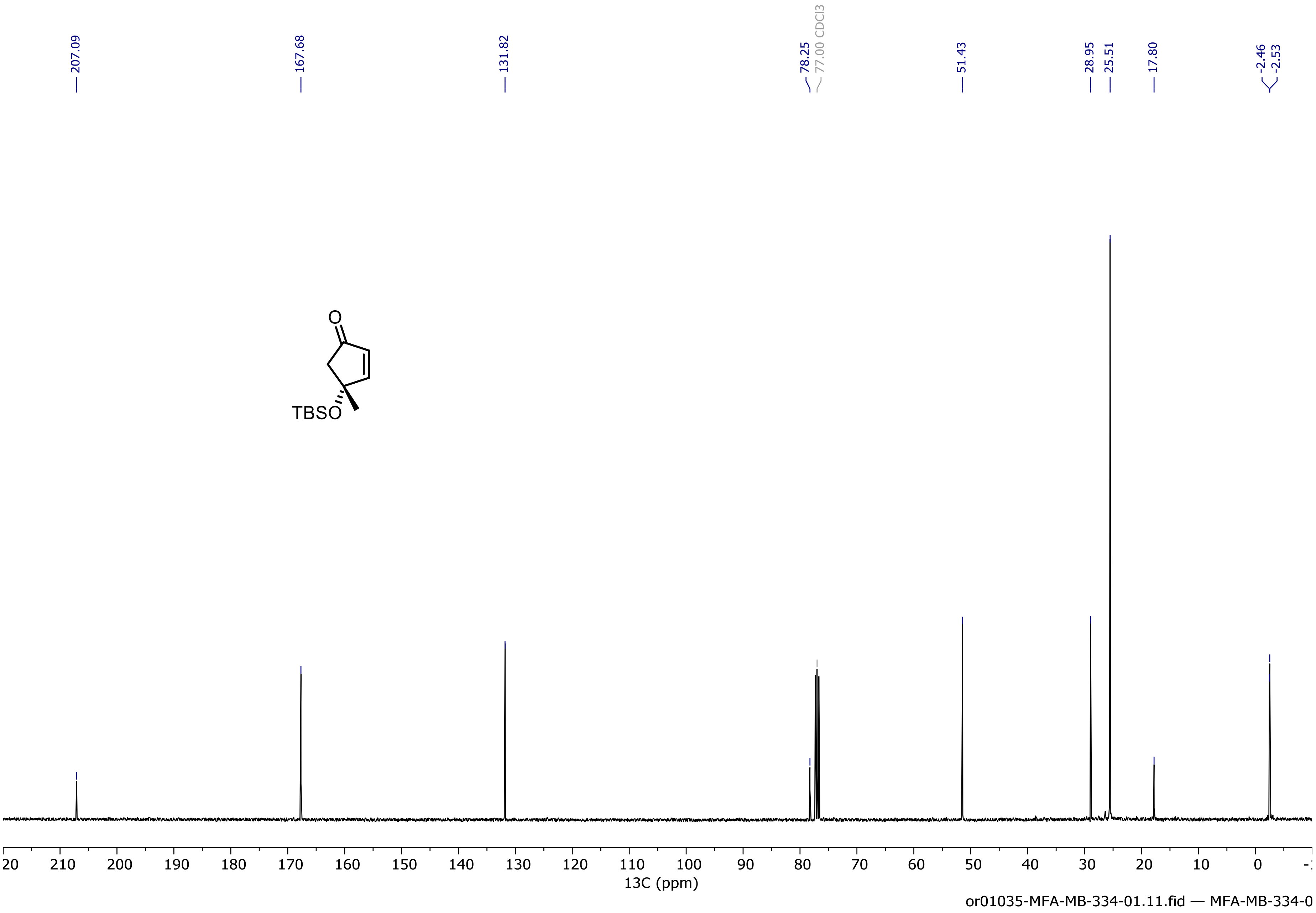

^{1}H NMR Spectrum of 16 (600 MHz, $CDCl_3$)

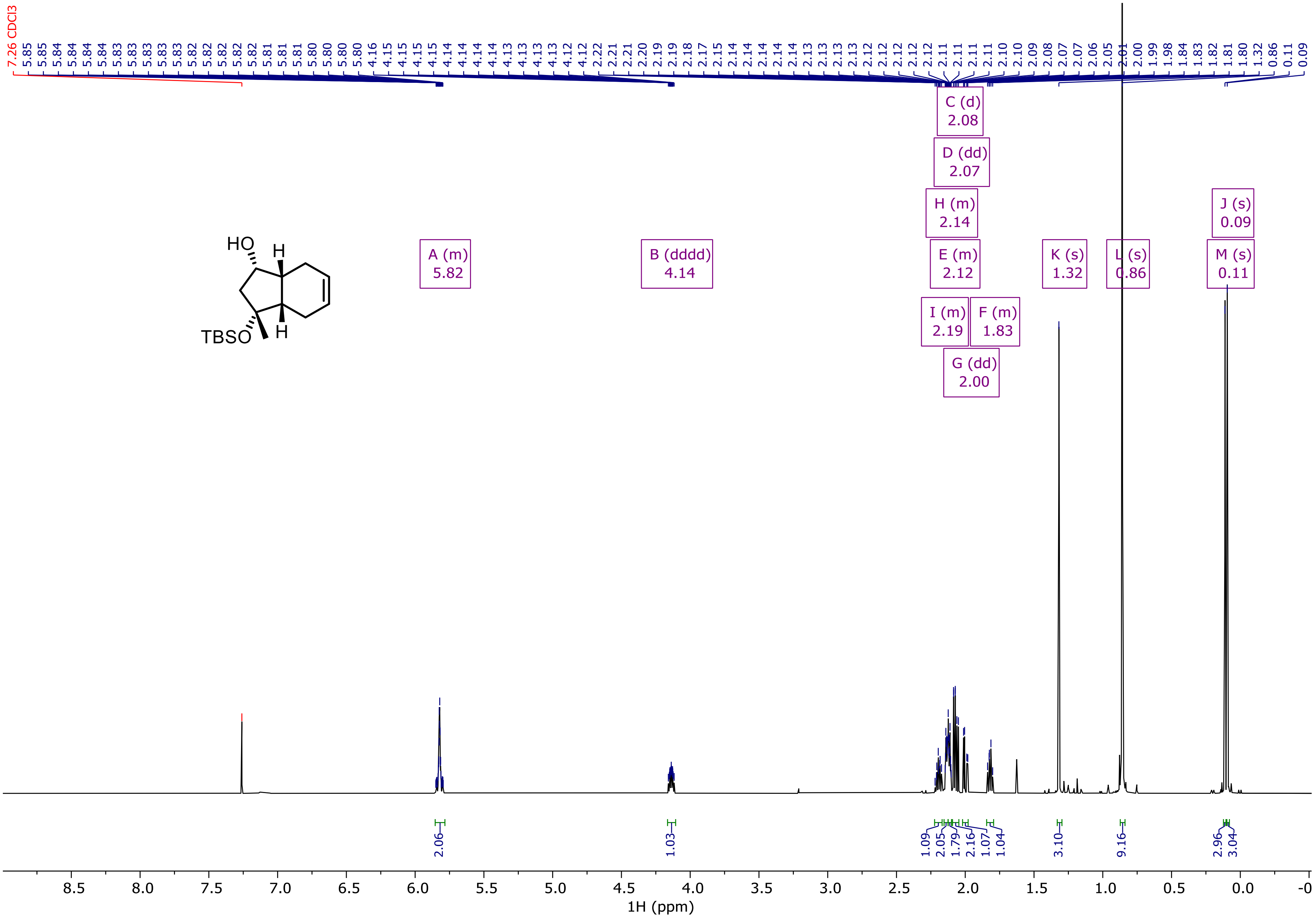

^{13}C NMR Spectrum of 16 (101 MHz, $CDCl_3$)

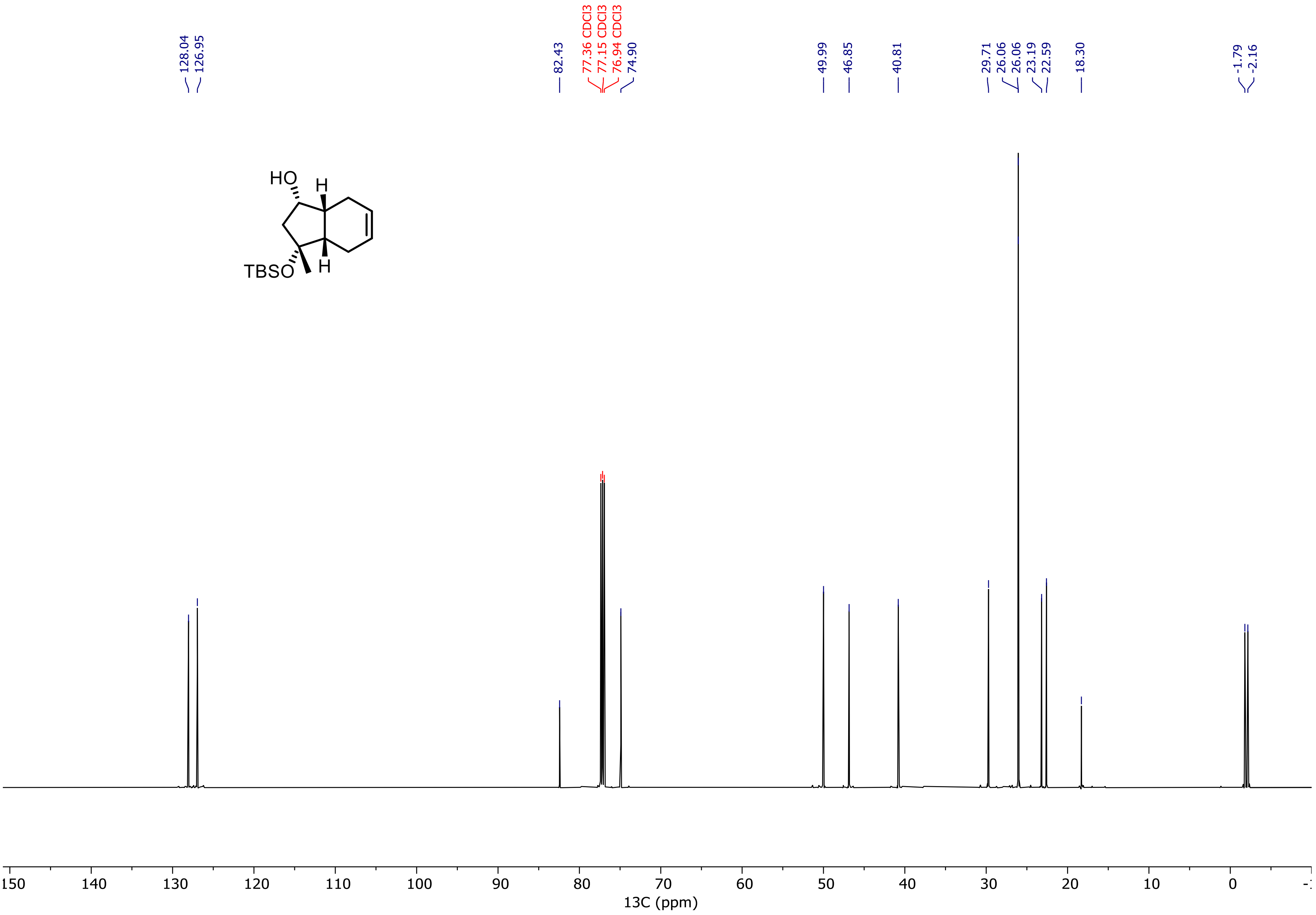

^{1}H NMR Spectrum of 17 (400 MHz, CDCl$_3$)

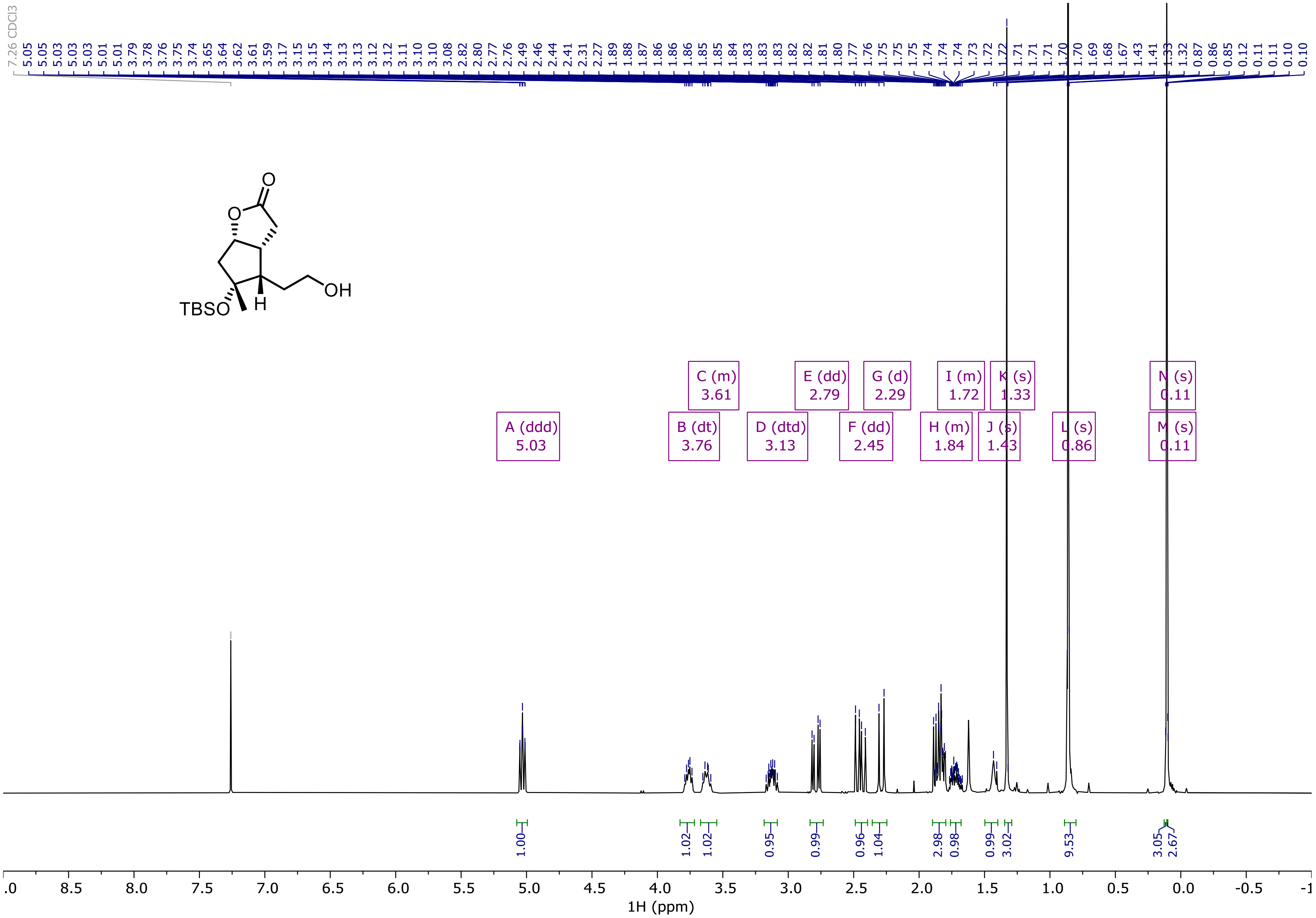

^{13}C NMR Spectrum of 17 (101 MHz, $CDCl_3$)

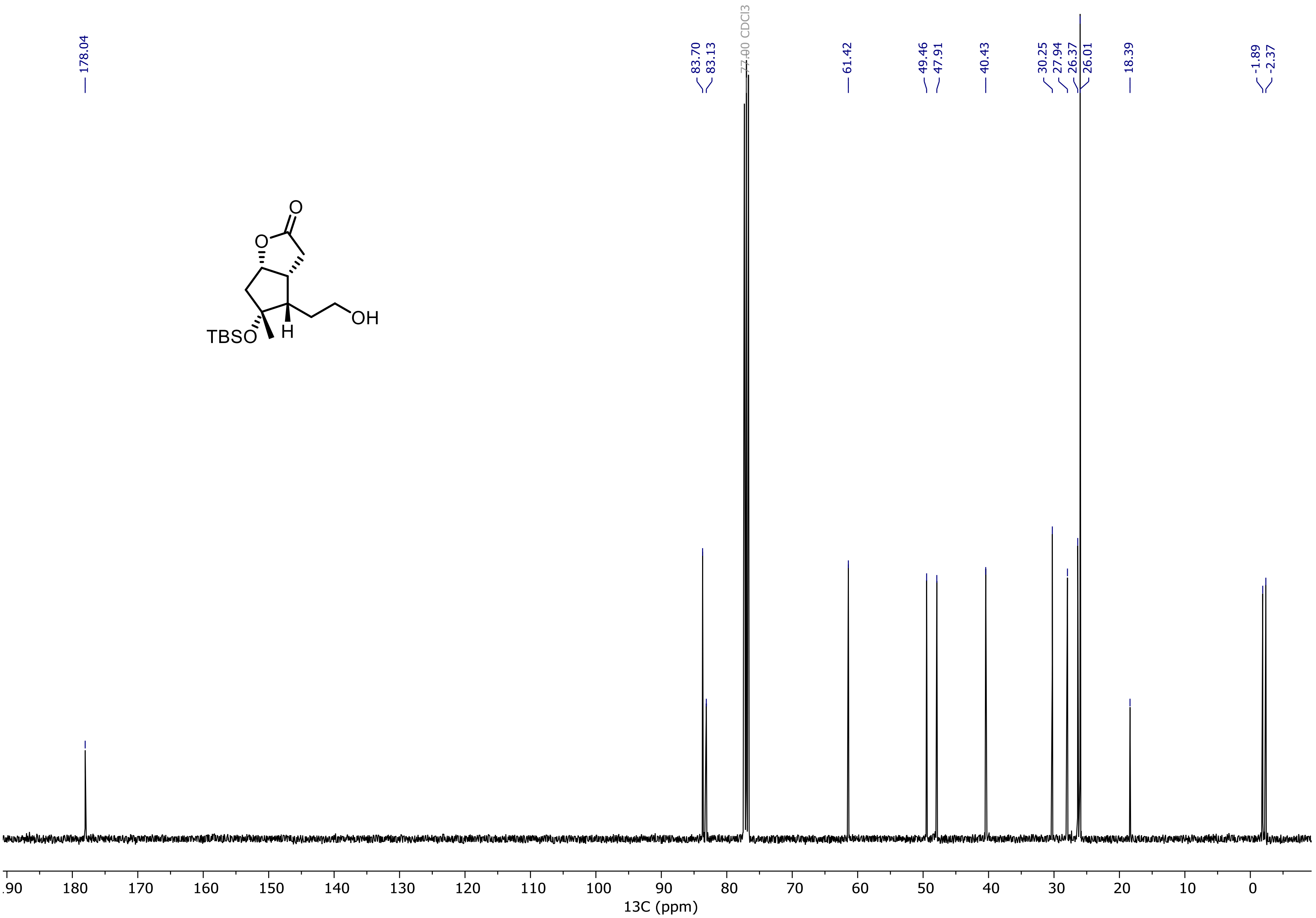

^{1}H NMR Spectrum of 18 (400 MHz, $CDCl_3$)

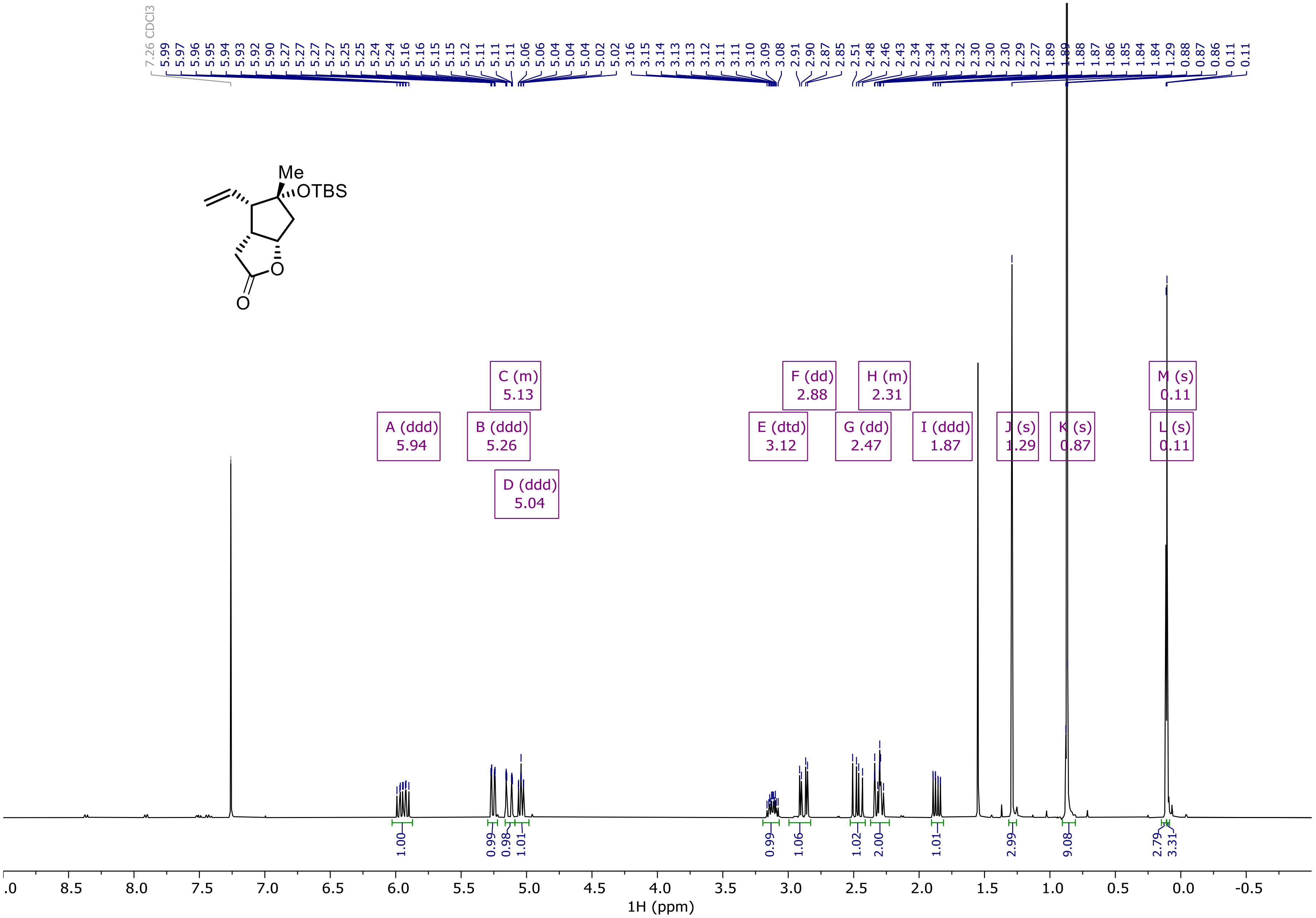

^{13}C NMR Spectrum of 18 (101 MHz, $CDCl_3$)

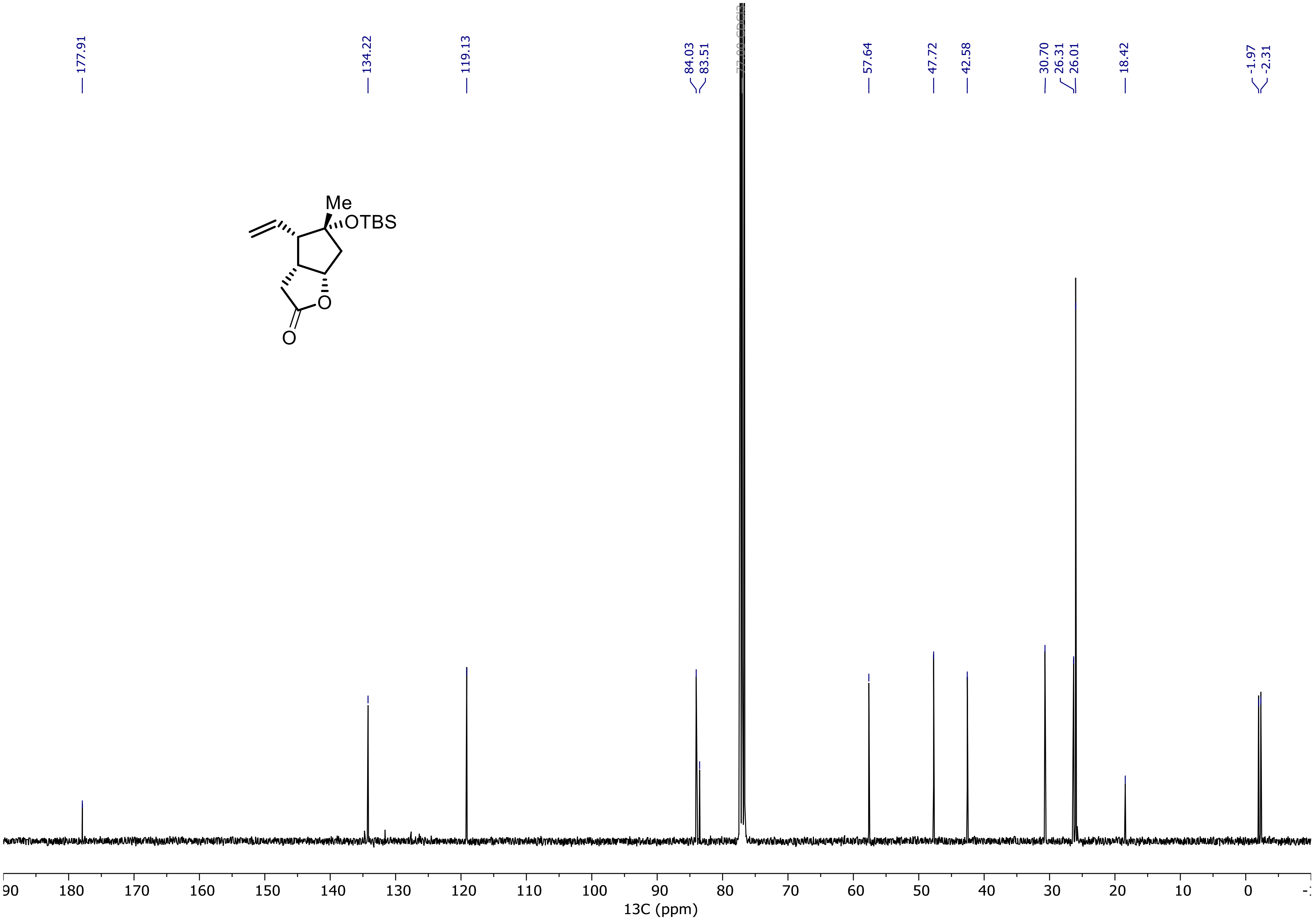

^{1}H NMR Spectrum of 20 (600 MHz, $CDCl_3$)

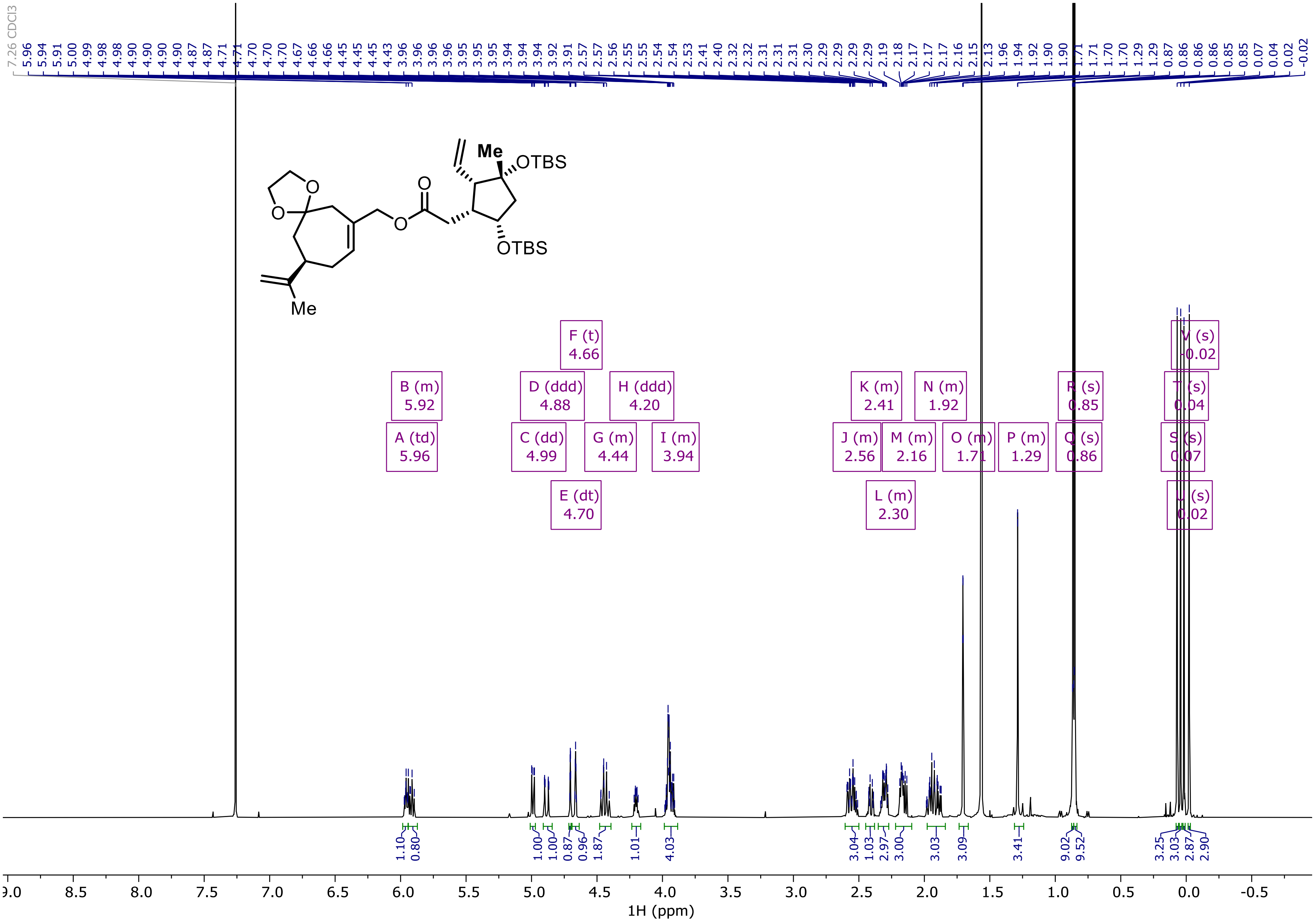

^{13}C NMR Spectrum of 20 (151 MHz, $CDCl_3$)

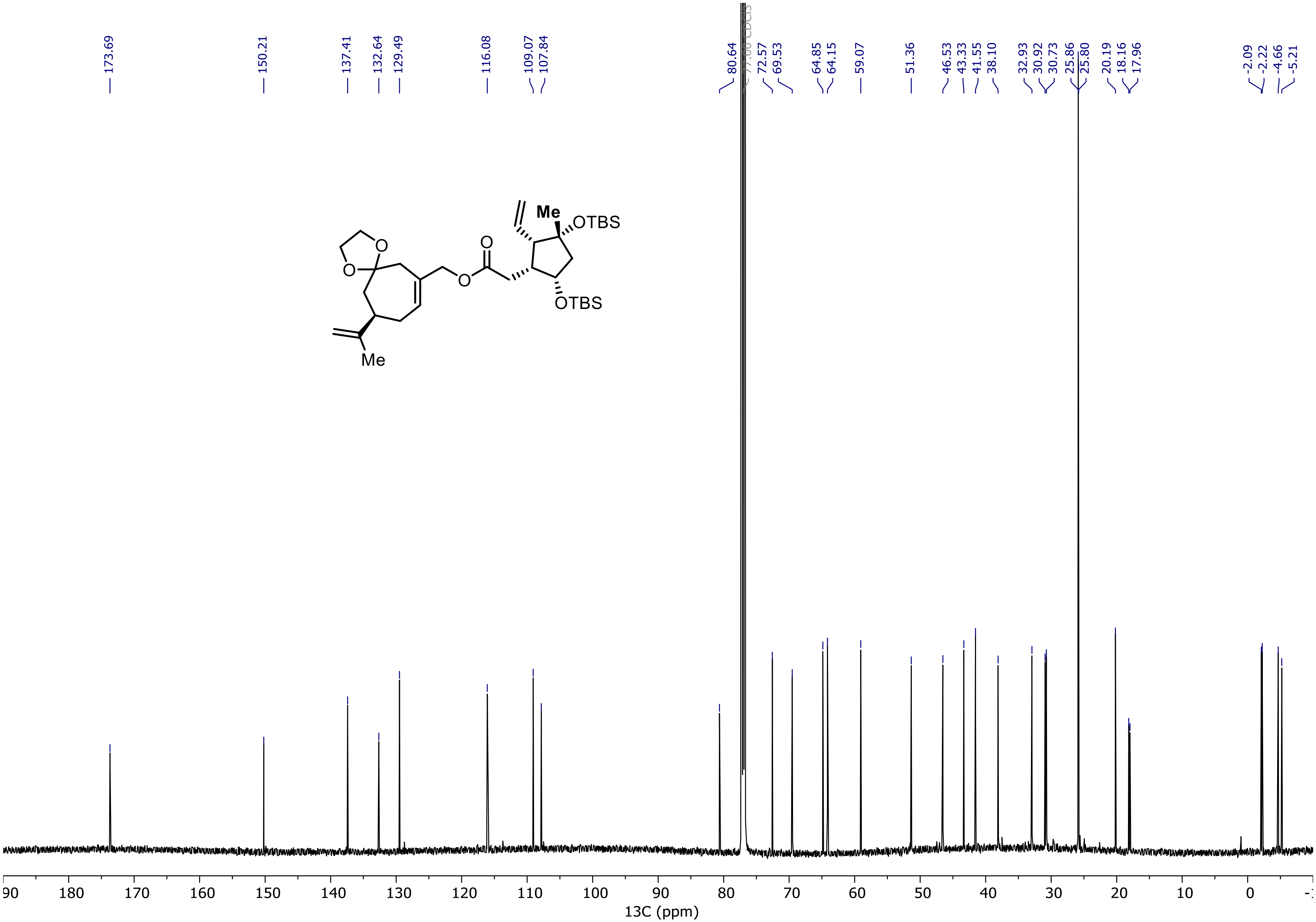

^{1}H NMR Spectrum of 22 (400 MHz, $CDCl_3$)

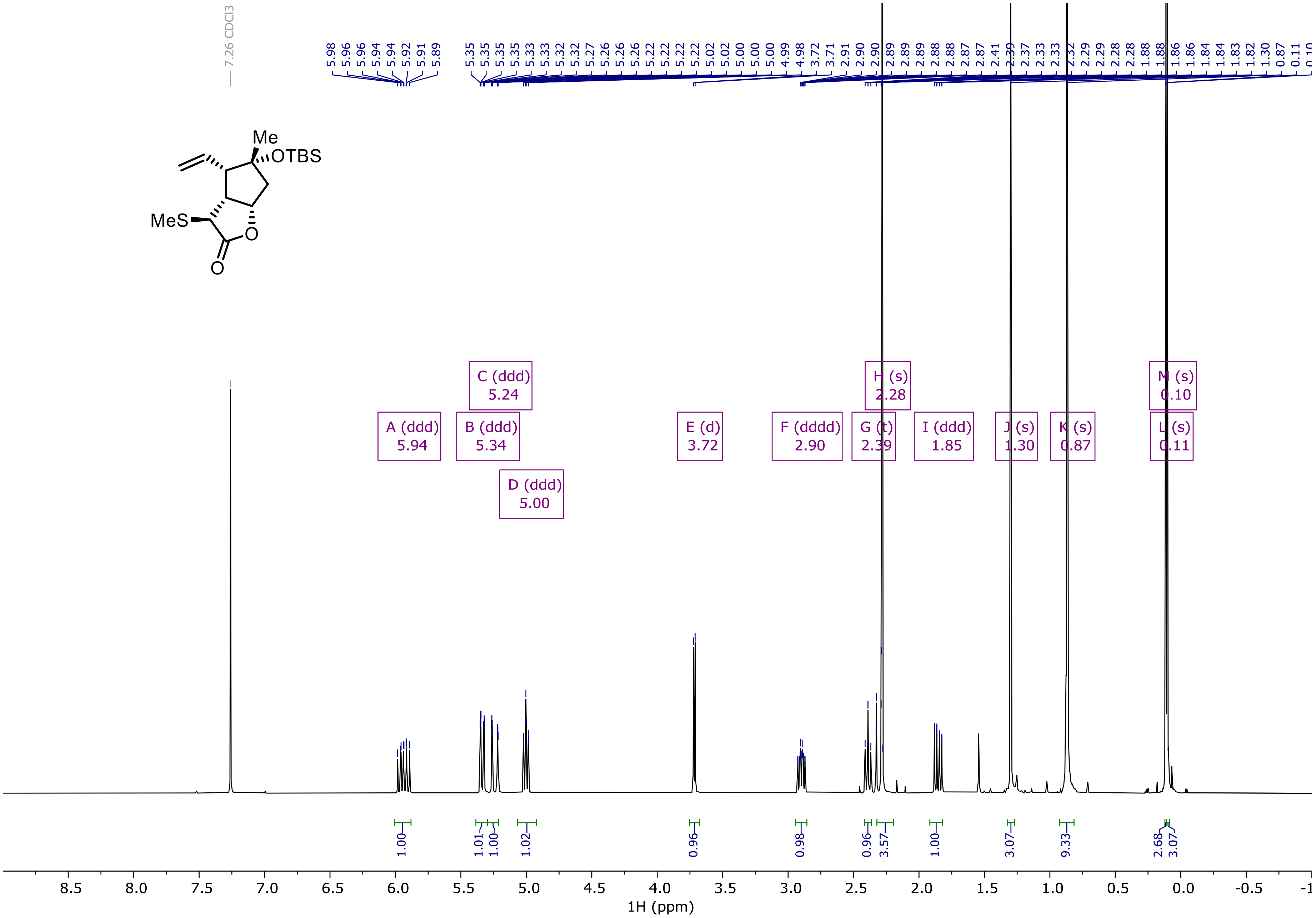

^{13}C NMR Spectrum of 22 (101 MHz, $CDCl_3$)

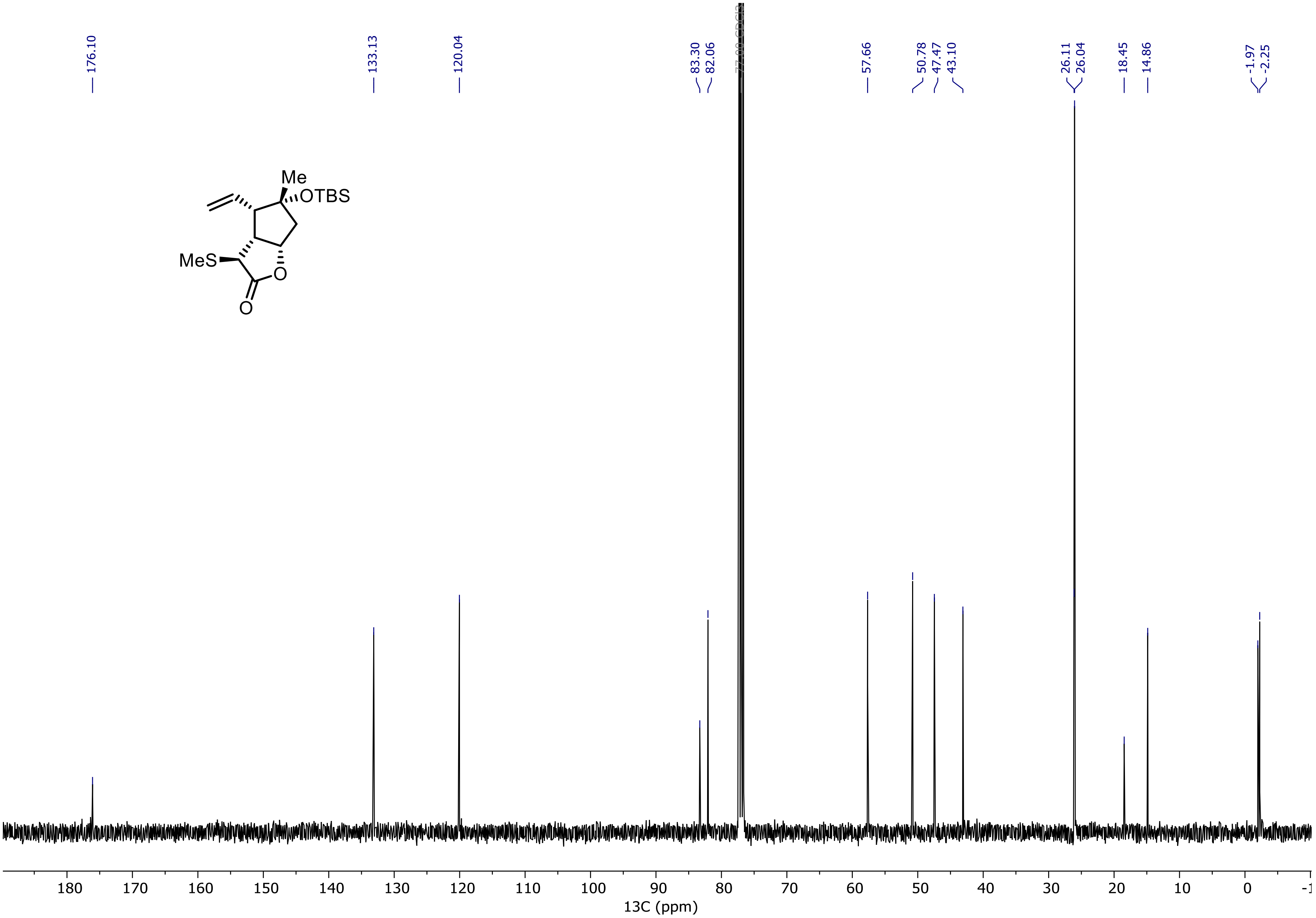

^{1}H NMR Spectrum of 26 (400 MHz, $CDCl_3$)

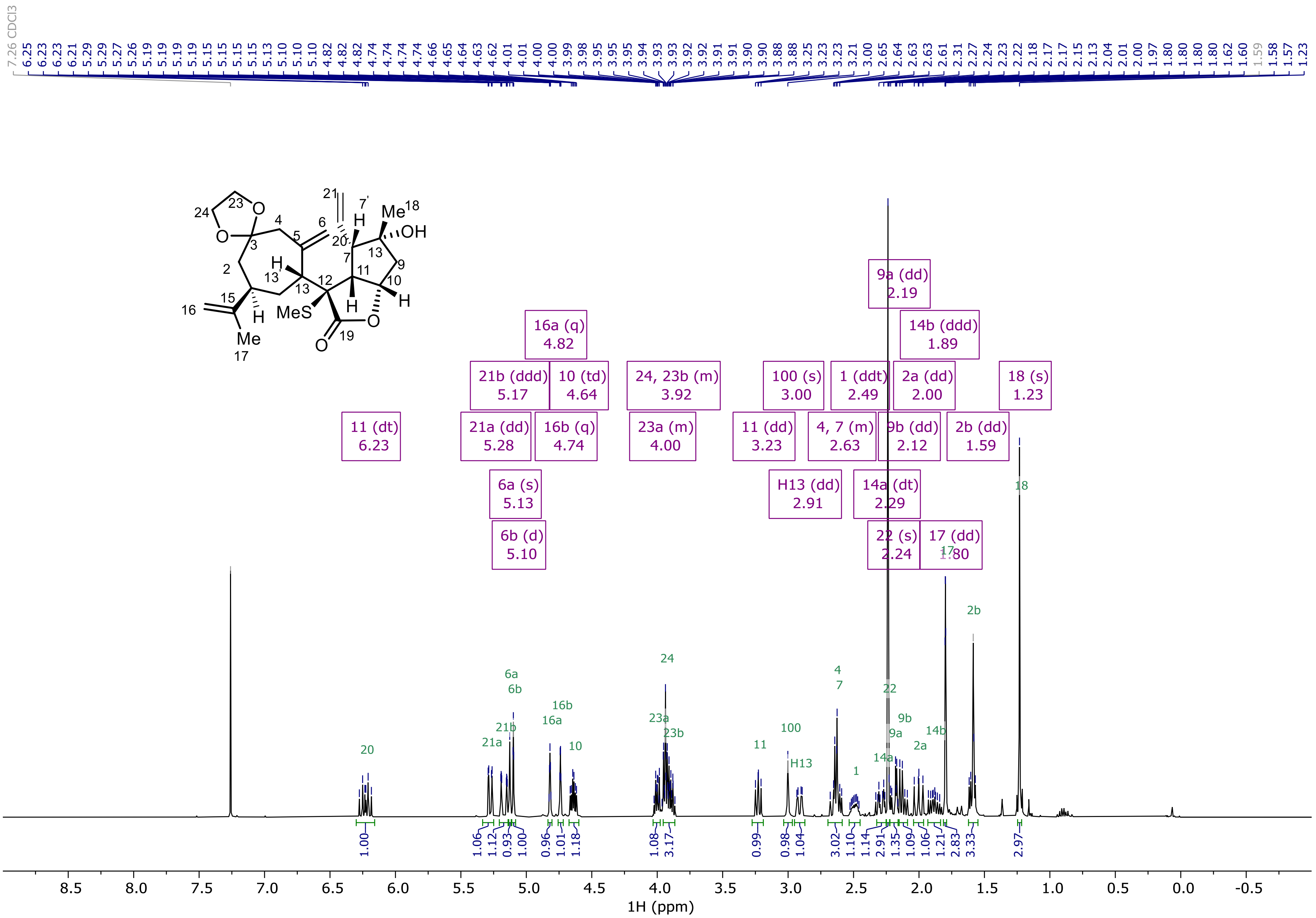

^{13}C NMR Spectrum of 26 (101 MHz, $CDCl_3$)

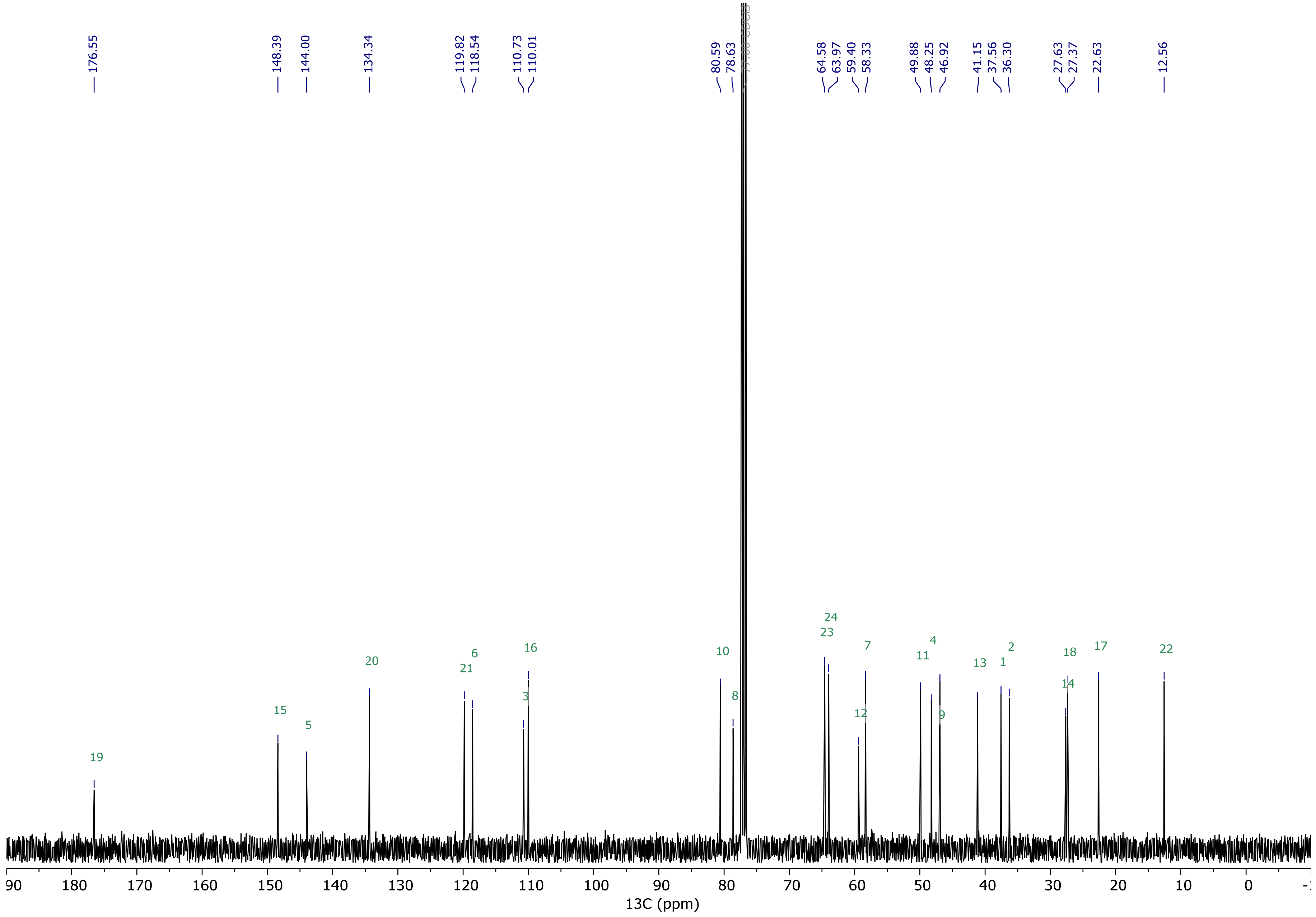

COSY Spectrum of 26

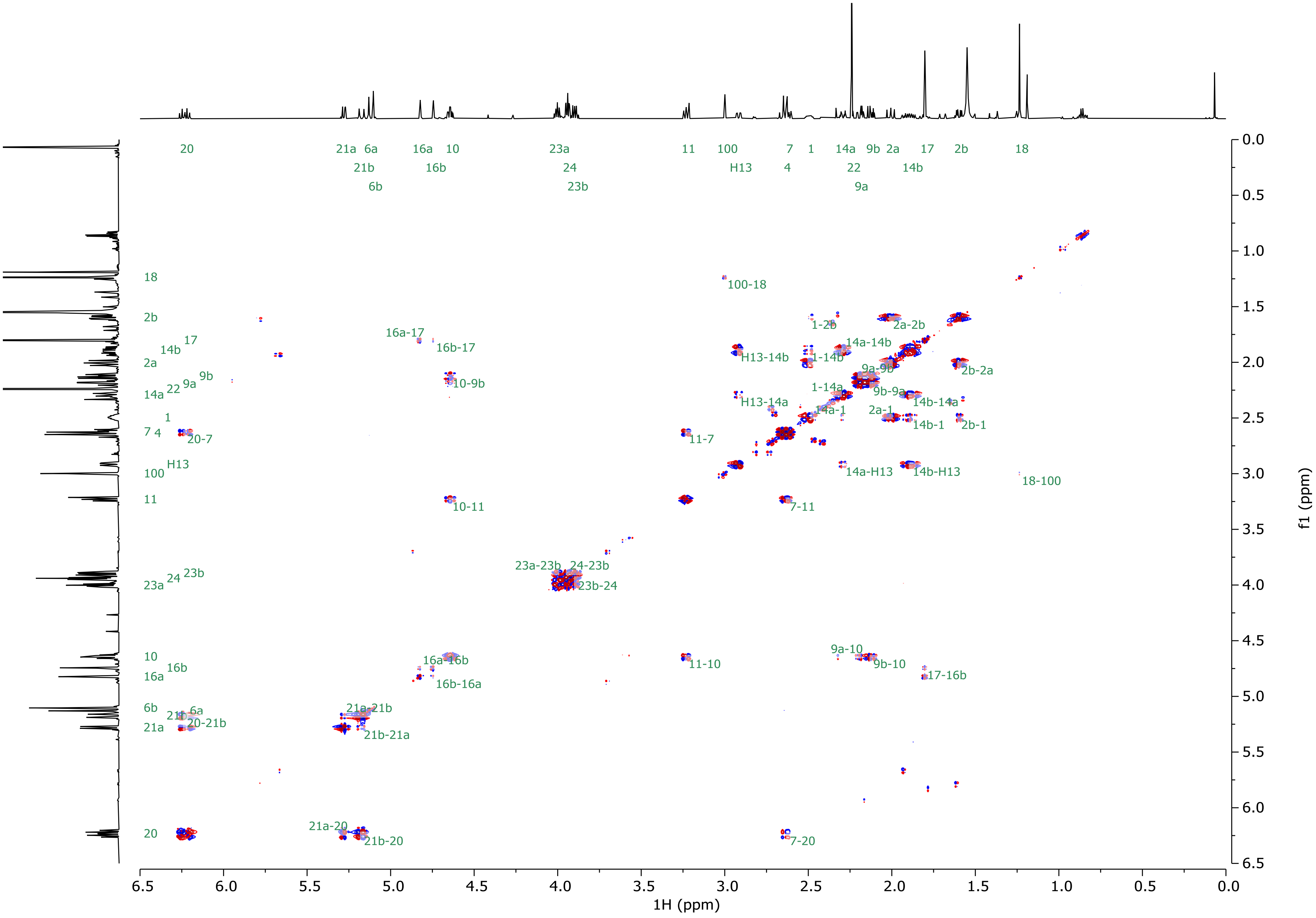

HMQC Spectrum of 26

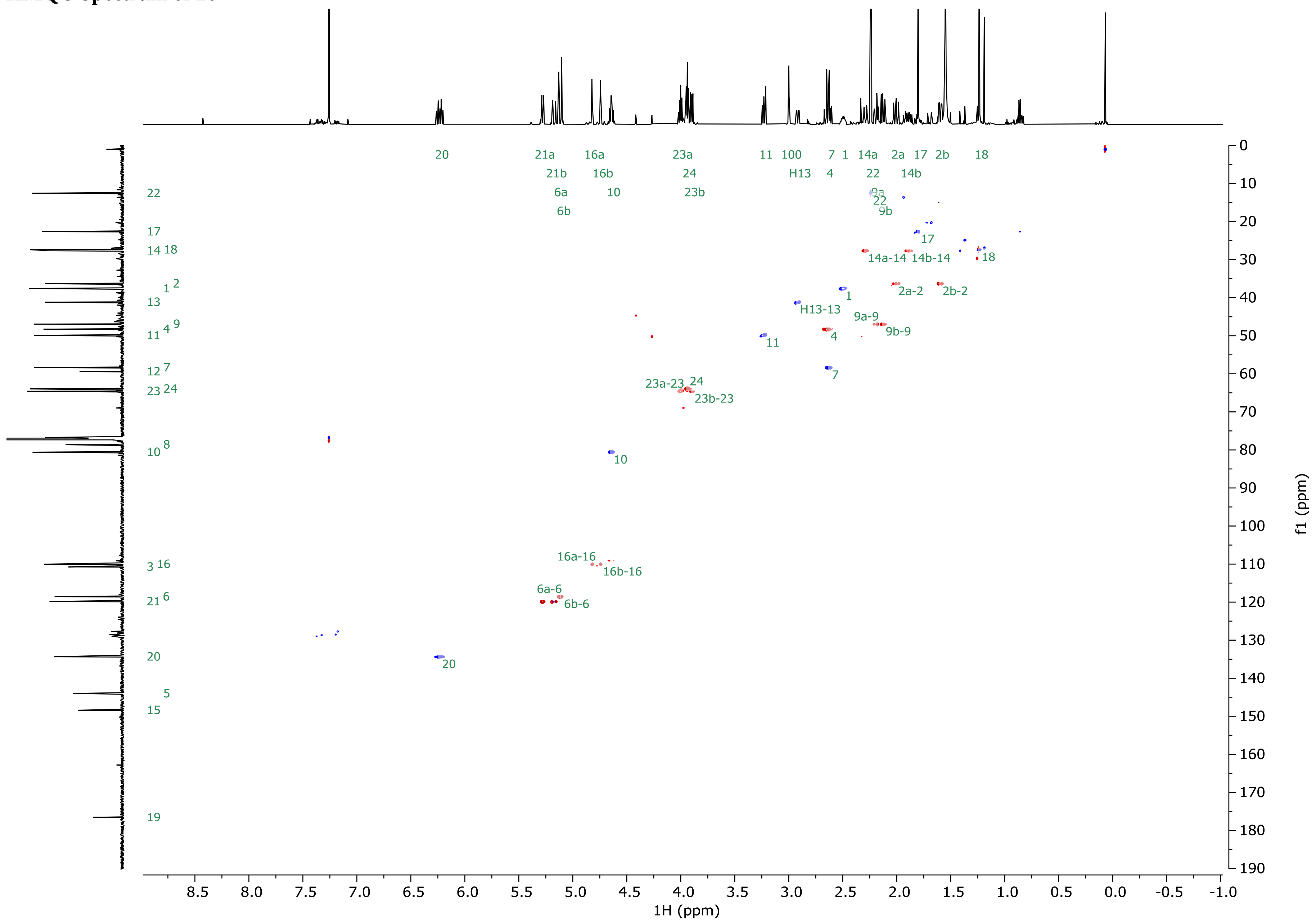

HMBC Spectrum of 26

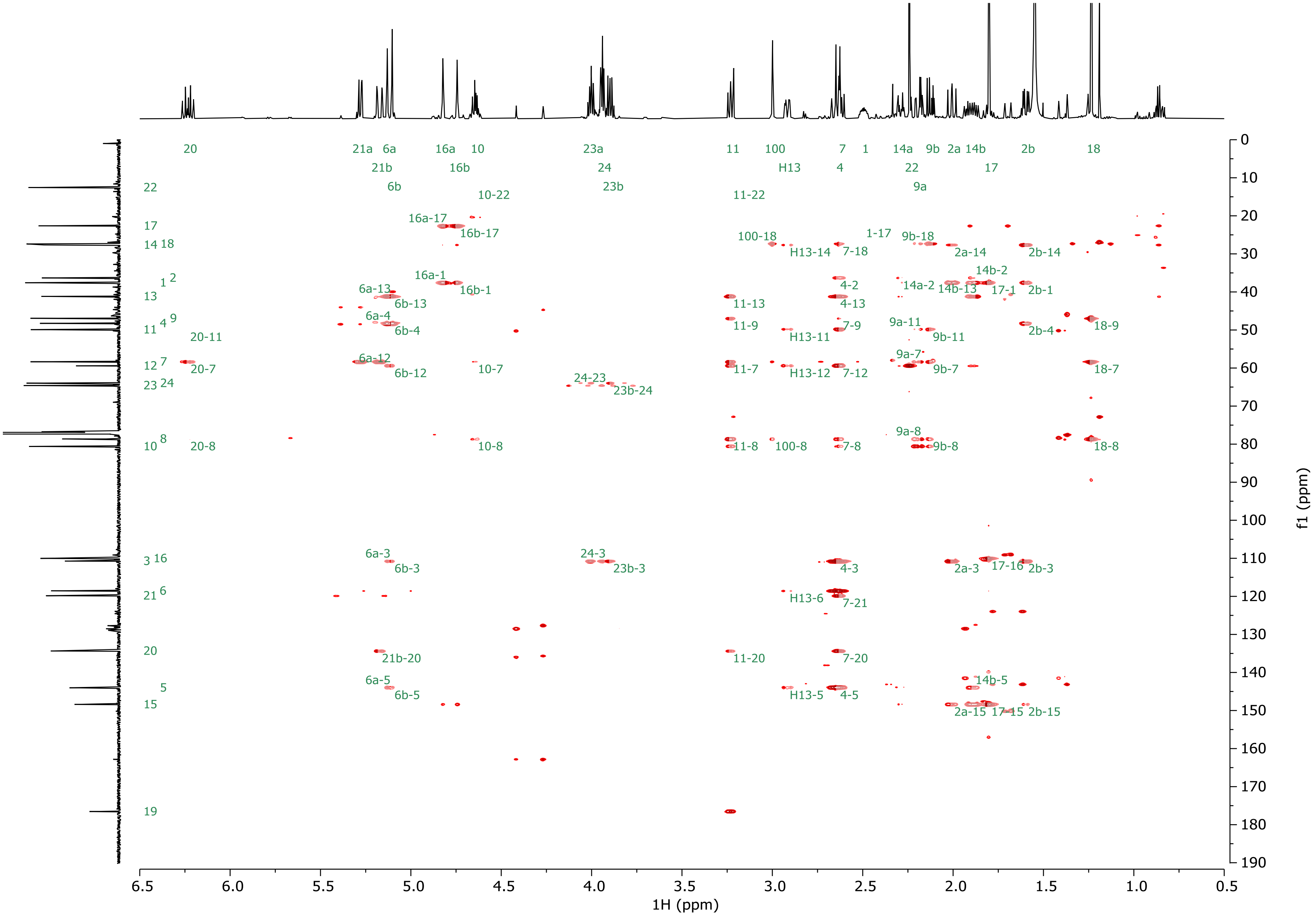

NOESY Spectrum of 26

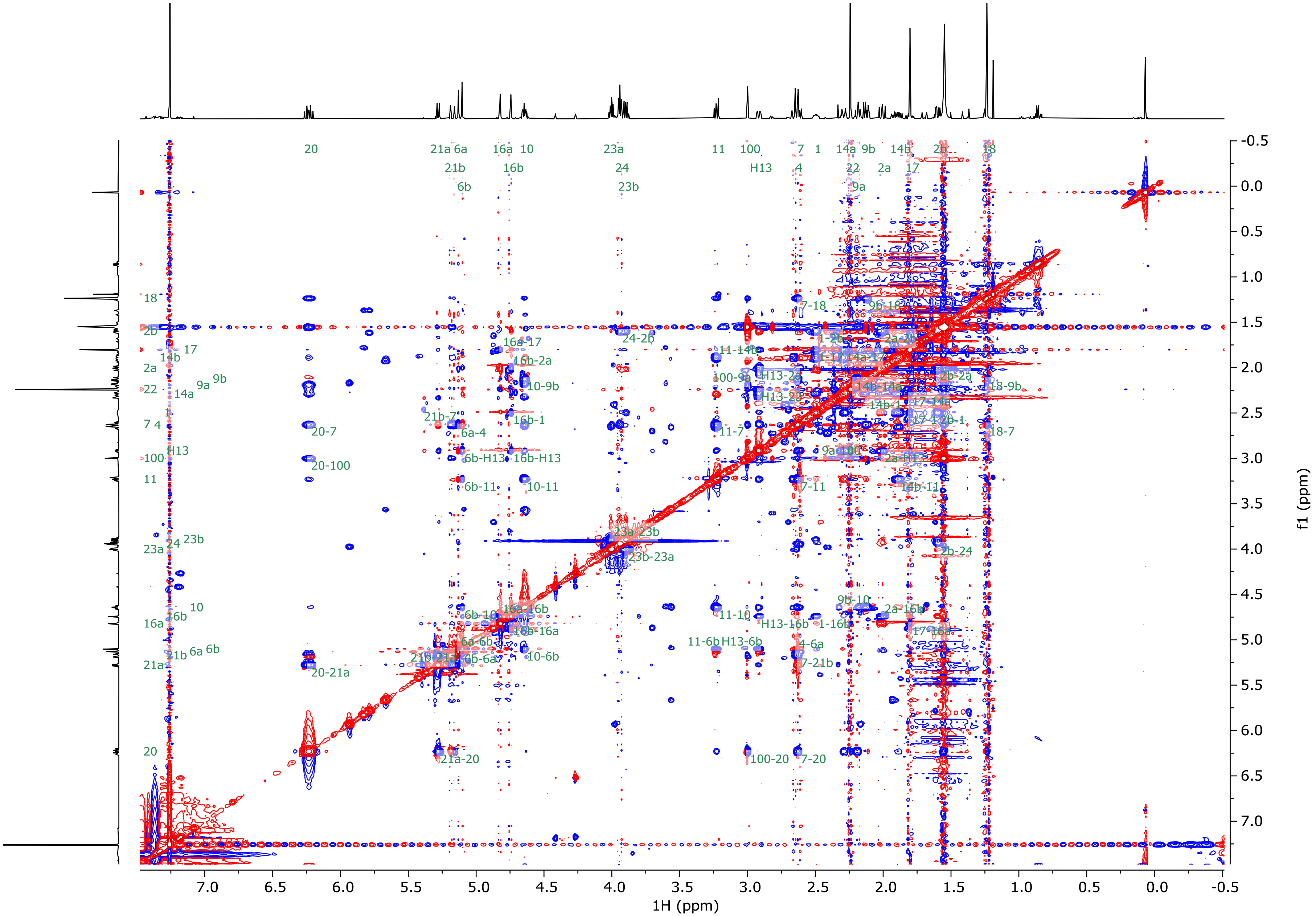

^{1}H NMR Spectrum of 27 (600 MHz, $CDCl_3$)

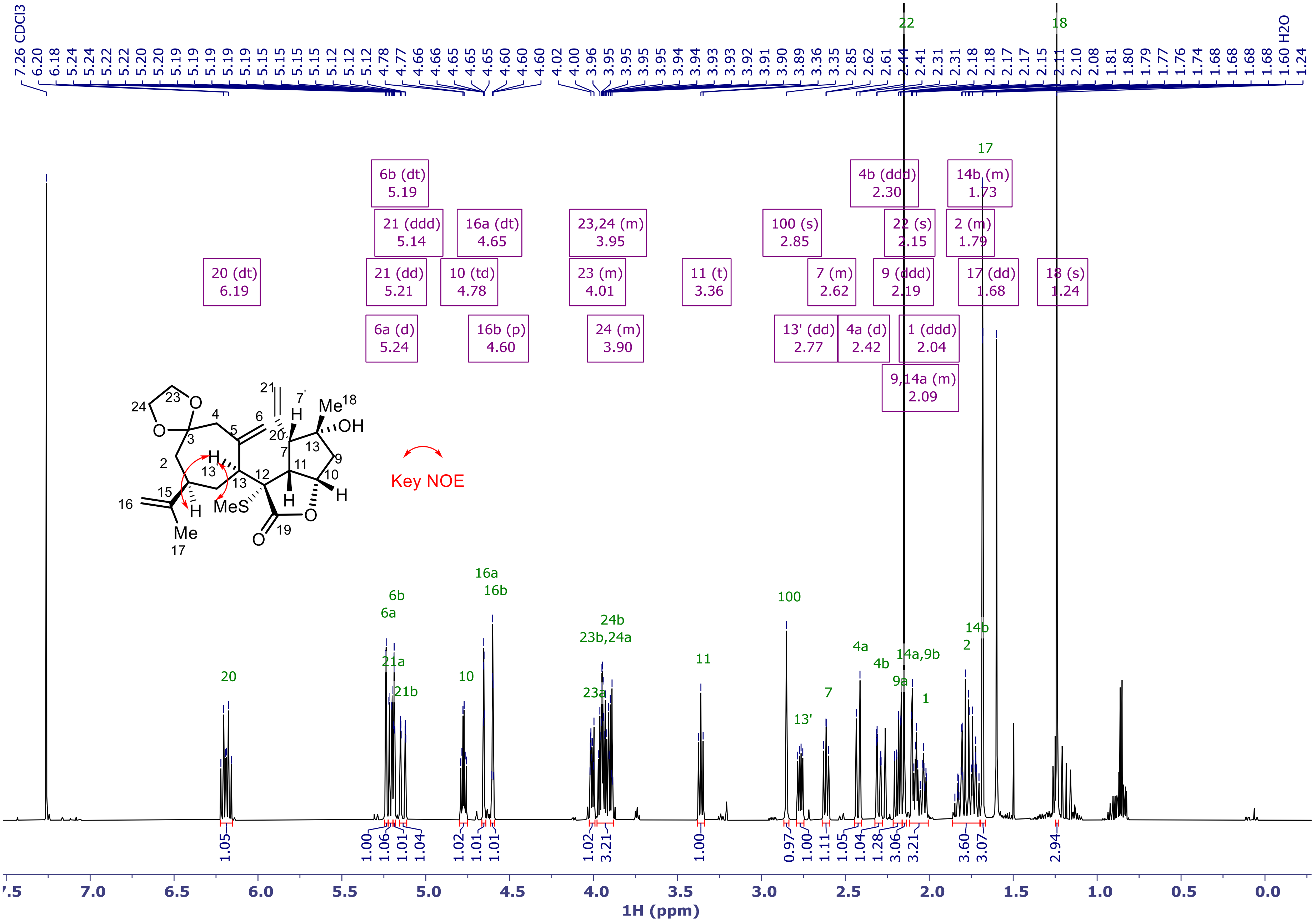

^{13}C NMR Spectrum of 27 (151 MHz, $CDCl_3$)

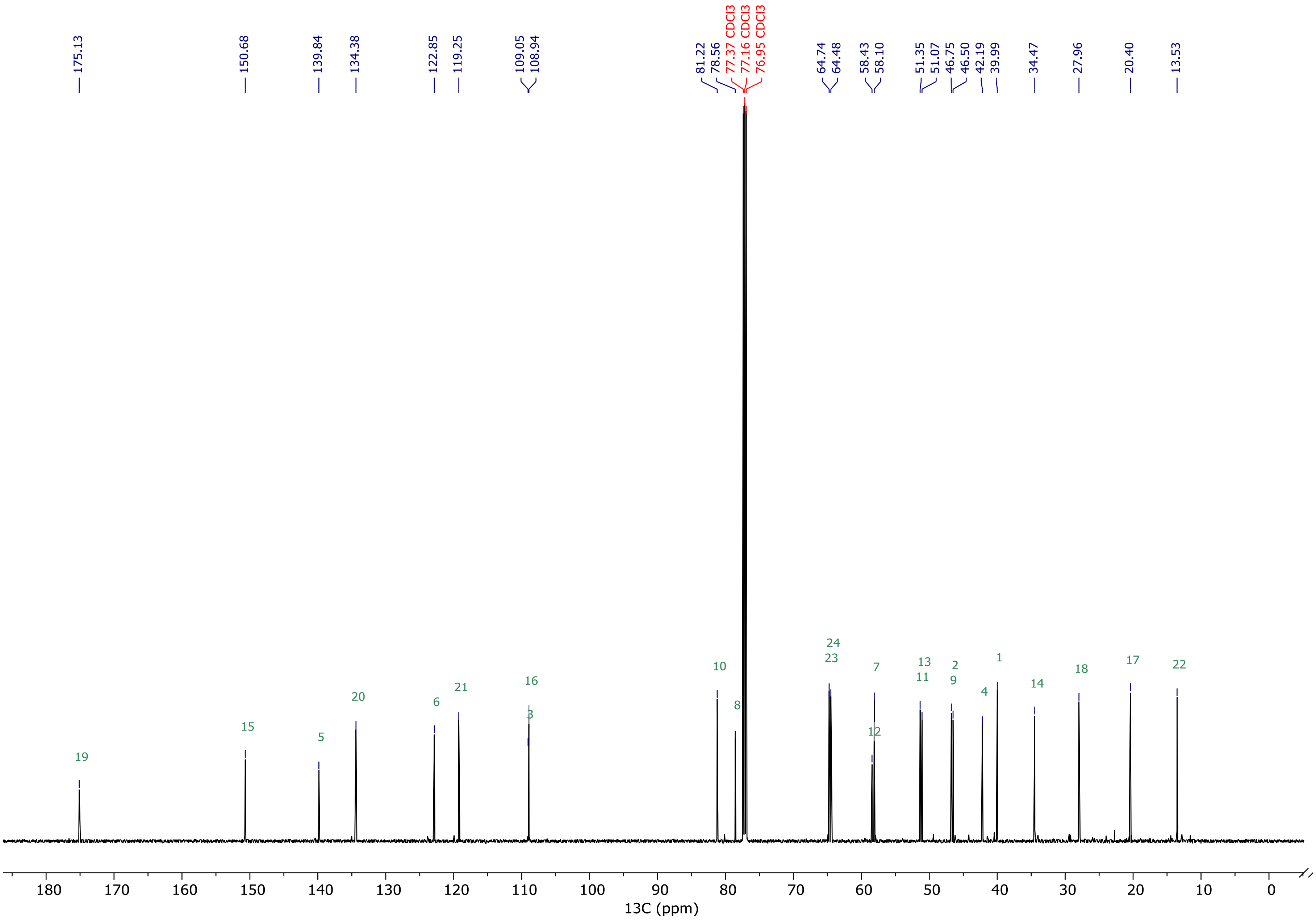

COSY of 27 (151 MHz, $CDCl_3$)

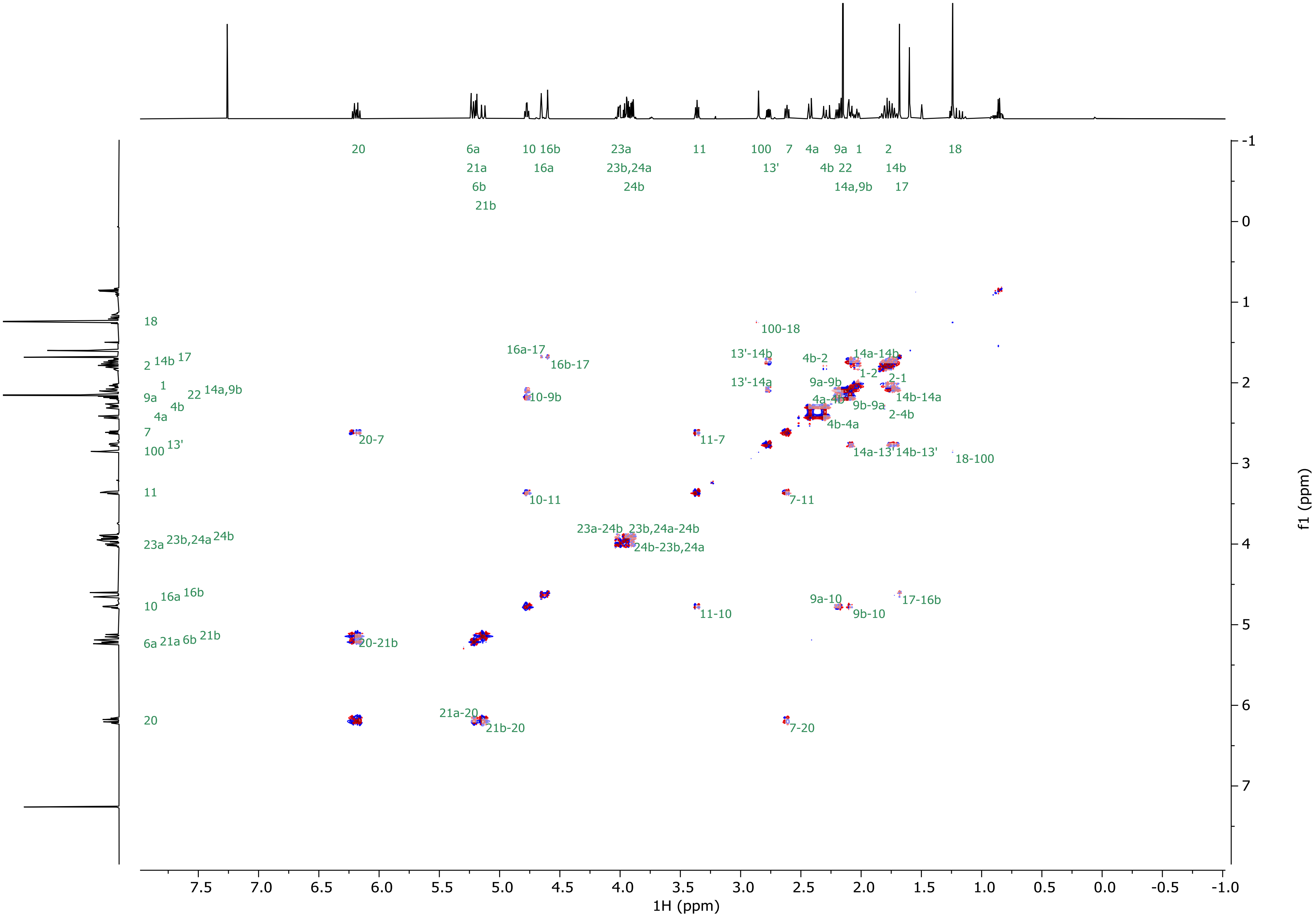

HSQC of 27 (151 MHz, $CDCl_3$)

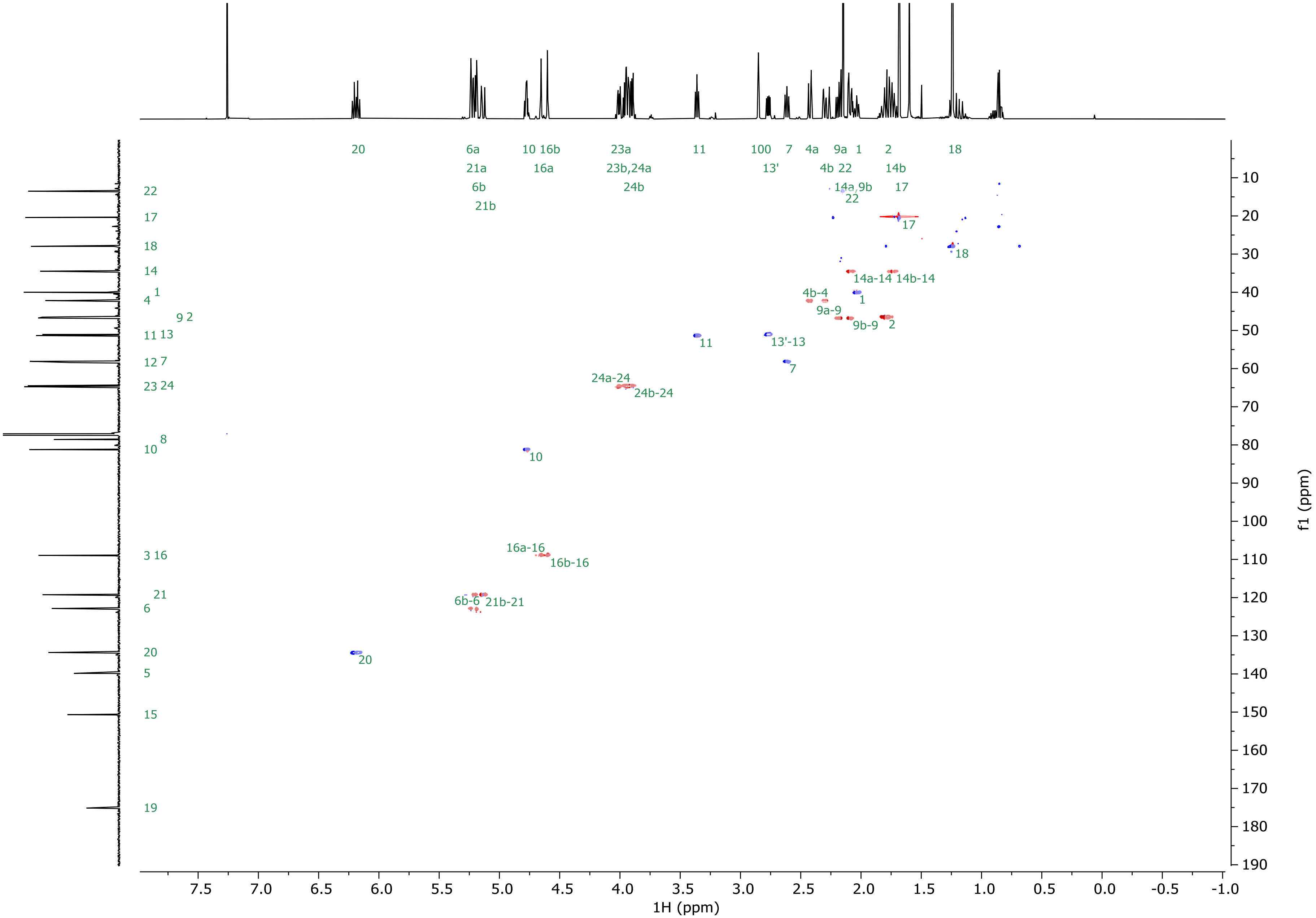

HMBC of 27 (151 MHz, $CDCl_3$)

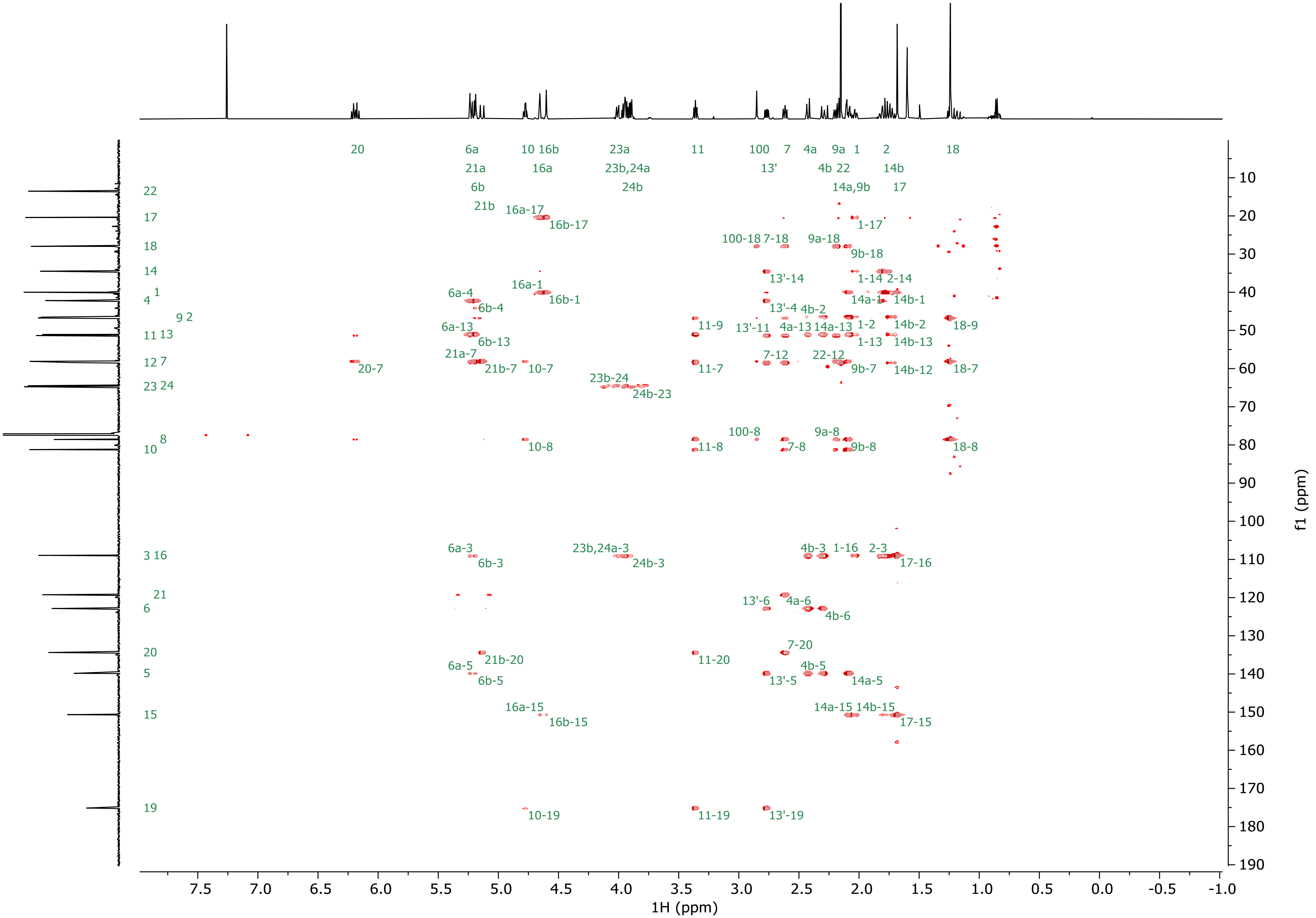

NOESY of 27 (151 MHz, $CDCl_3$)

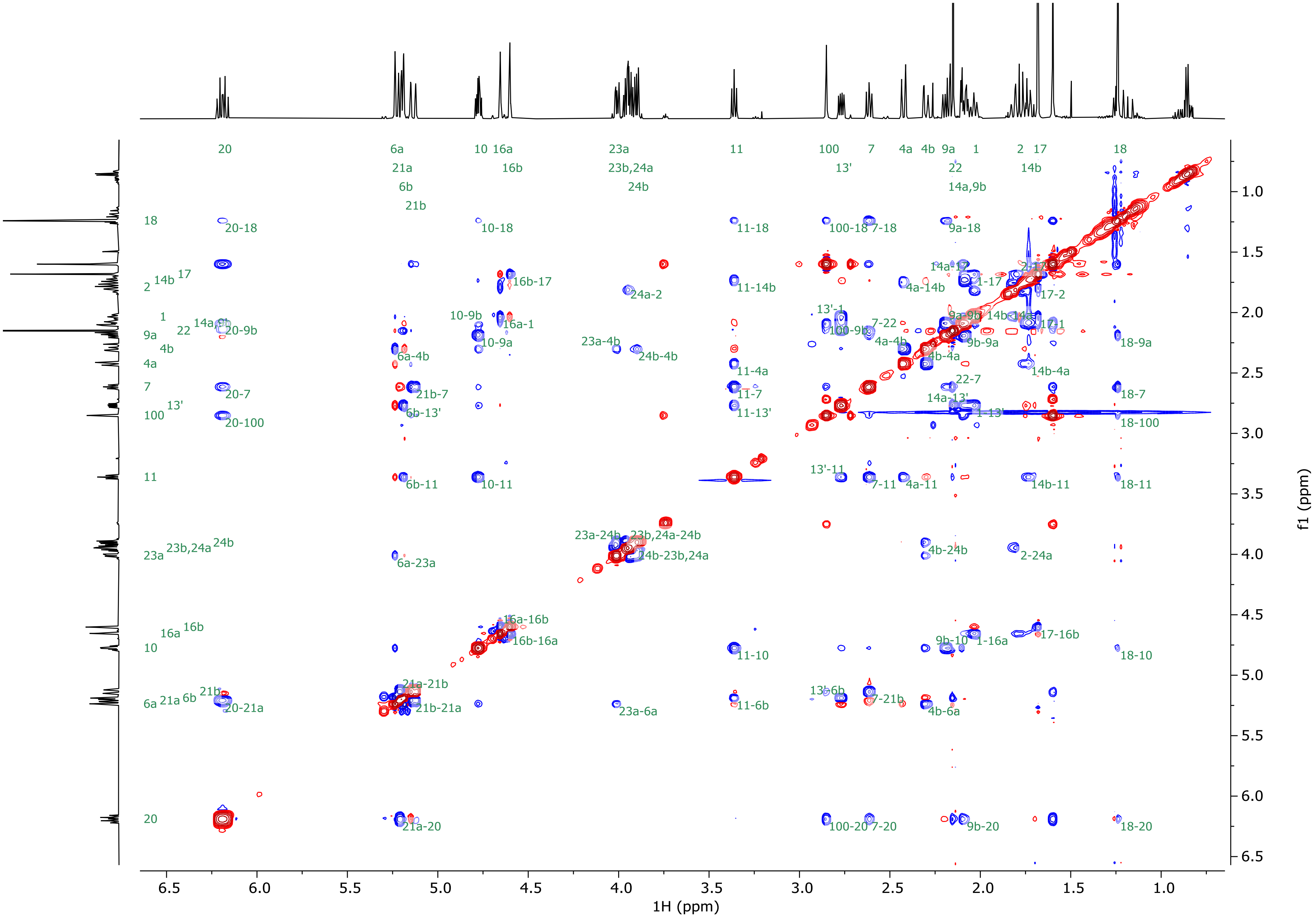

^{1}H NMR Spectrum of 28 (400 MHz, $CDCl_3$)

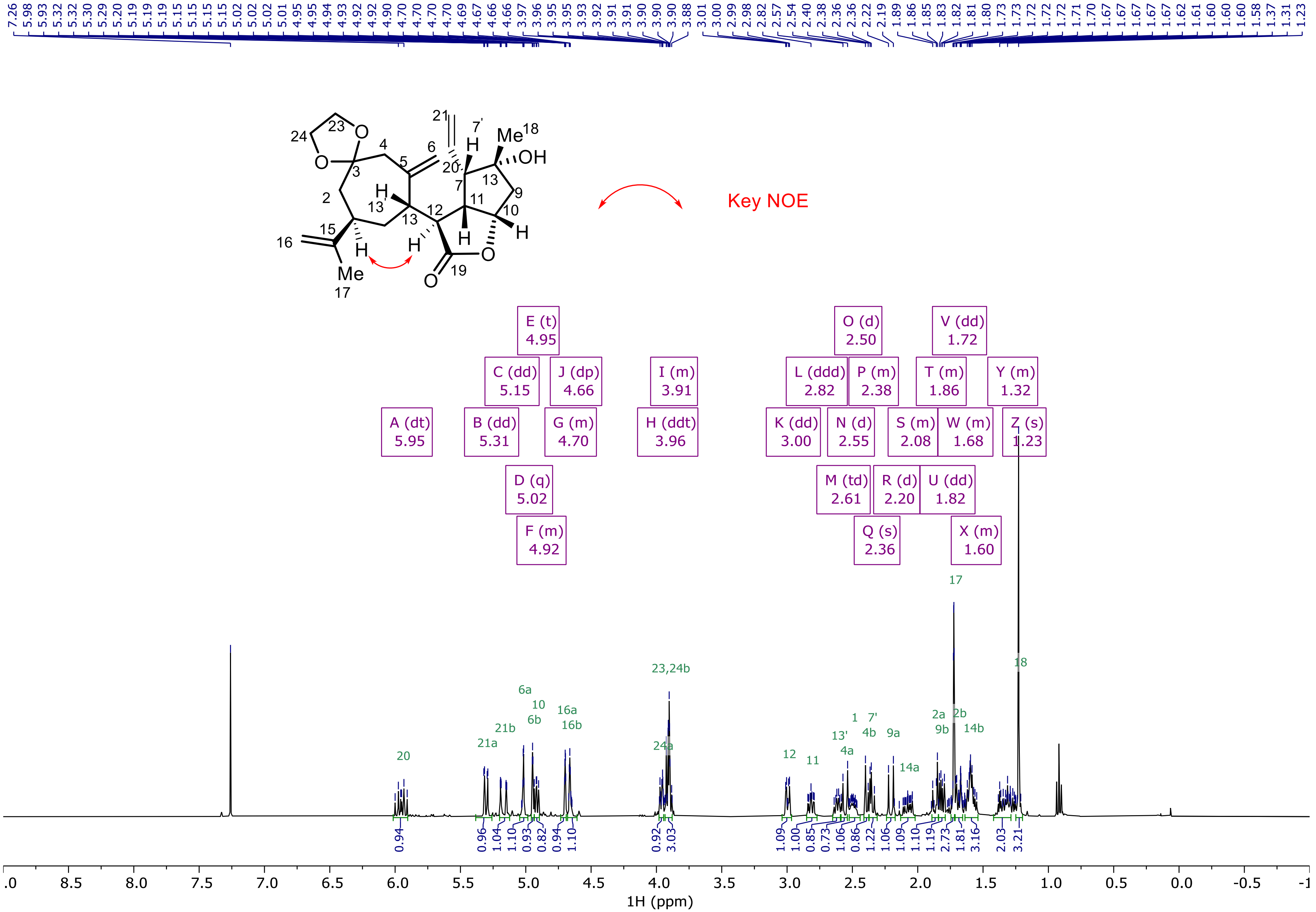

^{13}C NMR Spectrum of 28 (101 MHz, $CDCl_3$)

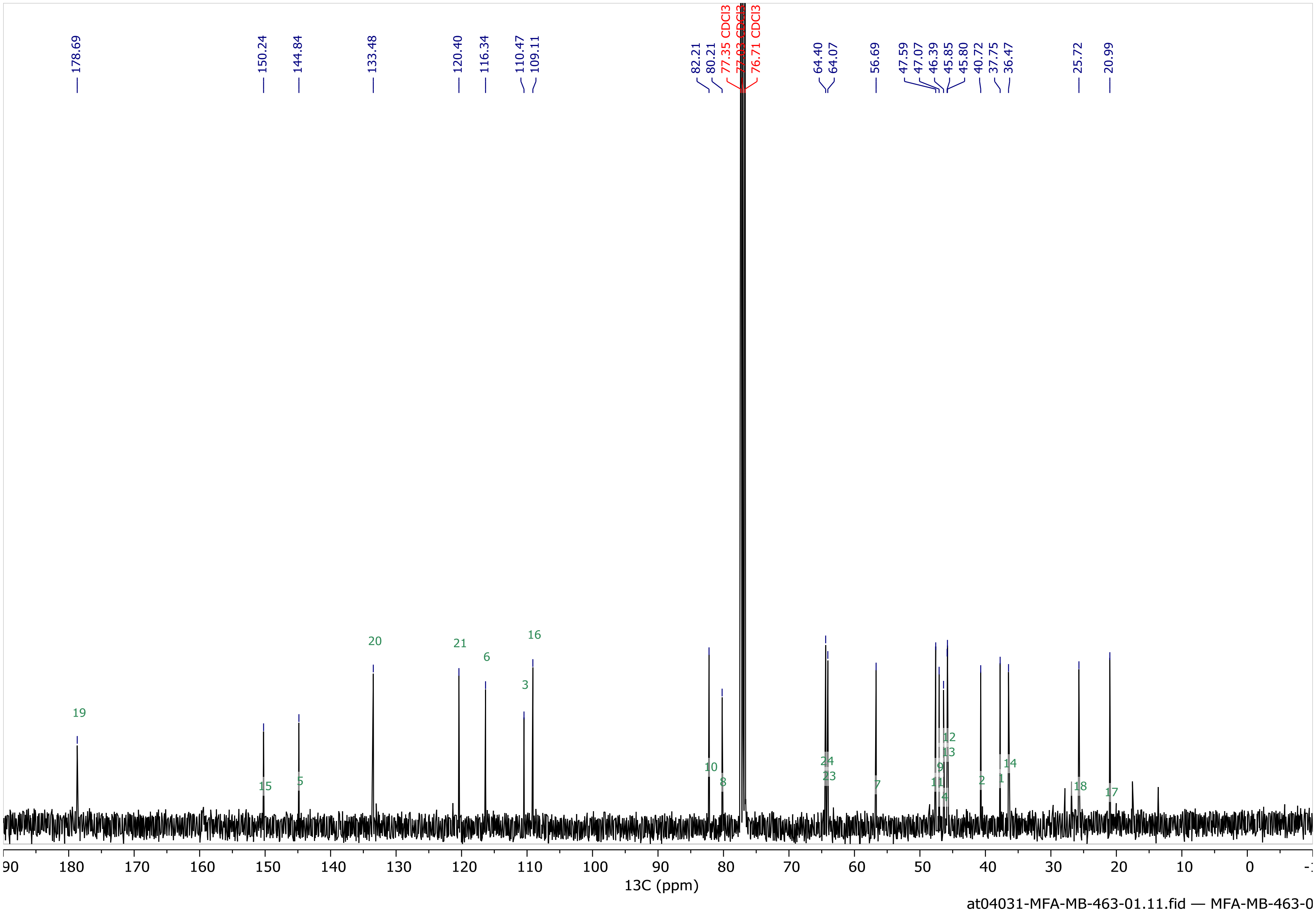

COSY Spectrum of 28

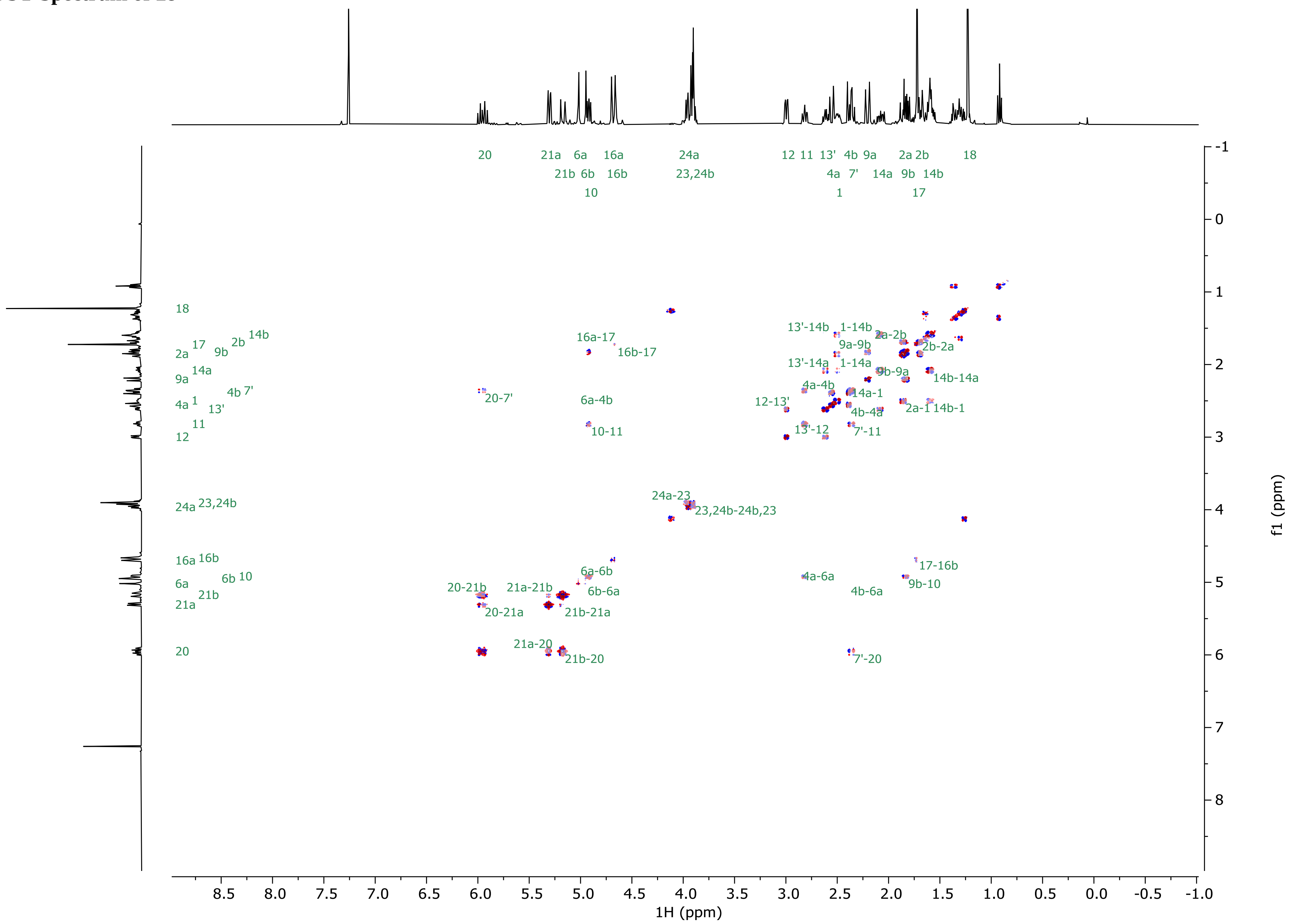

HSQC Spectrum of 28

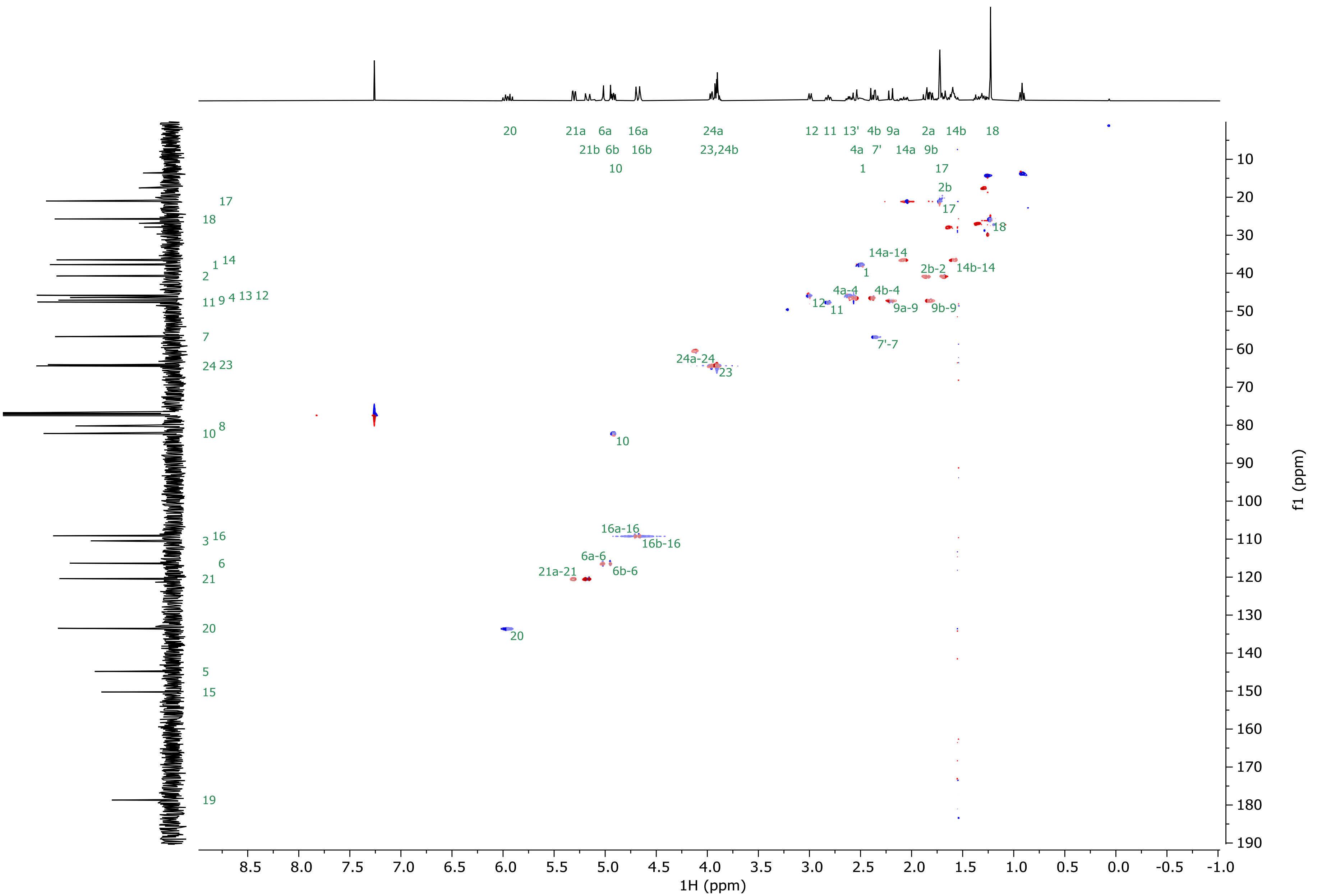

HMBC Spectrum of 28

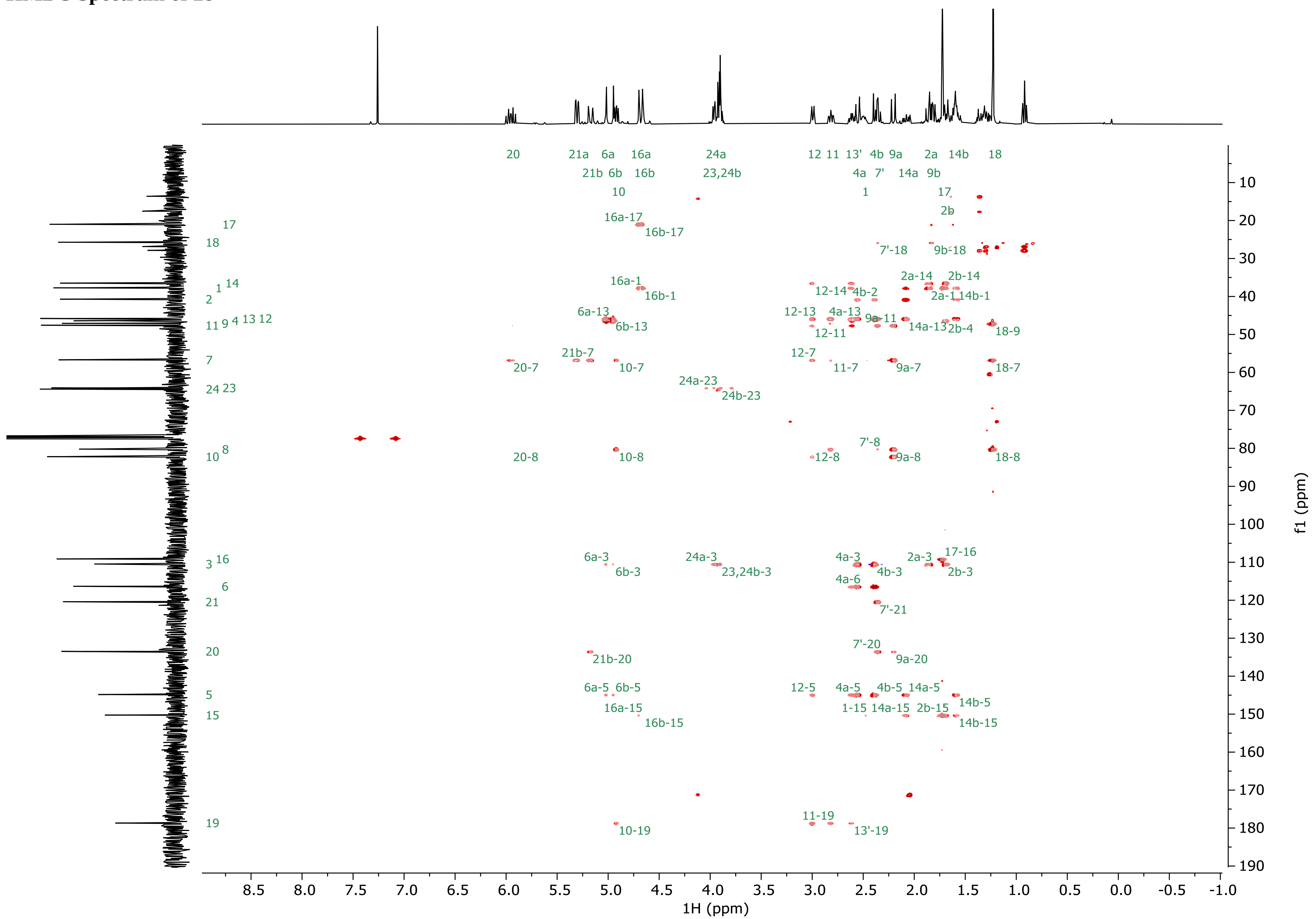

NOESY Spectrum of 28

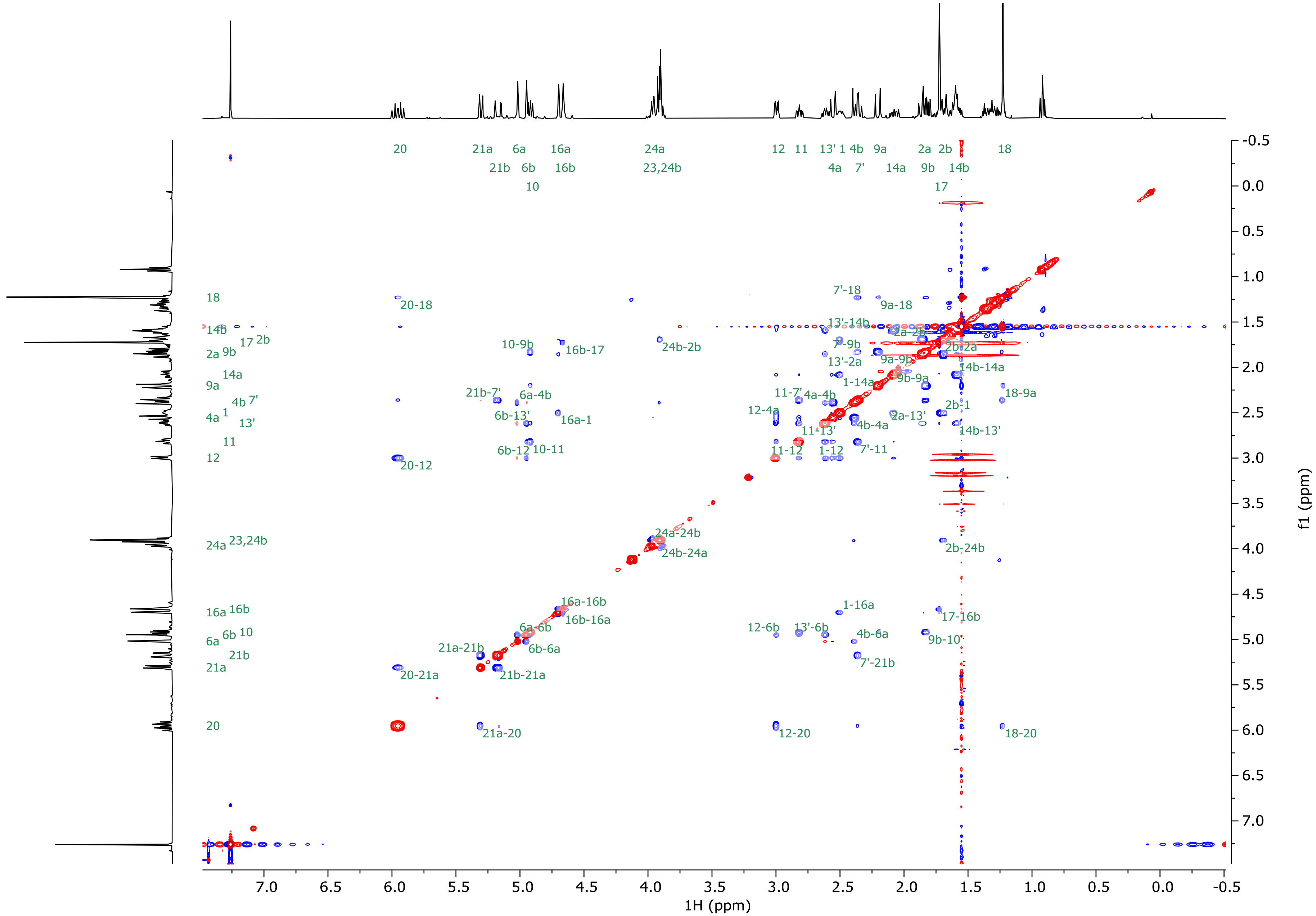

^{1}H NMR Spectrum of 13-*epi*-28 (600 MHz, $CDCl_3$)

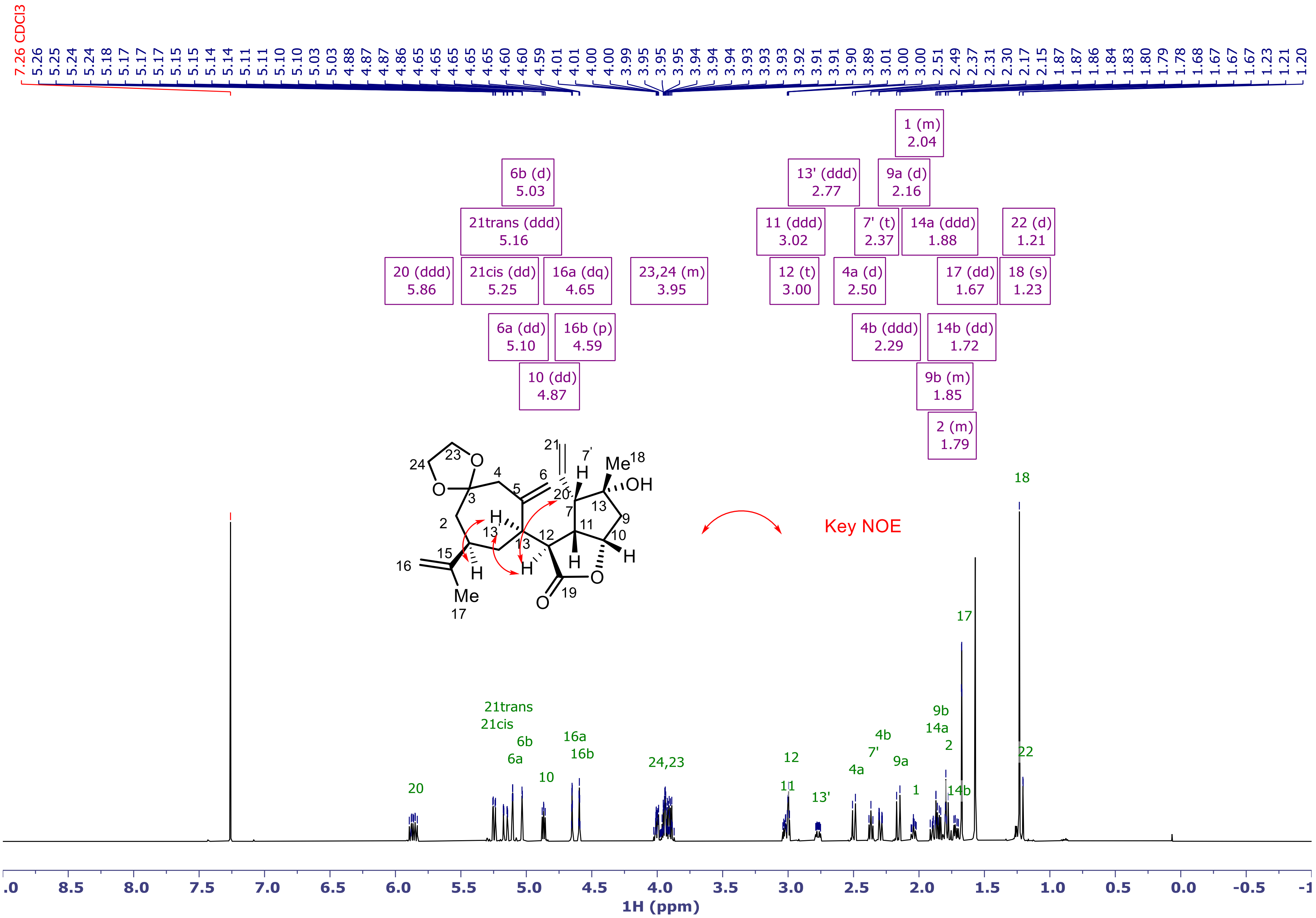

^{13}C NMR Spectrum of 13-*epi*-28 (151 MHz, $CDCl_3$)

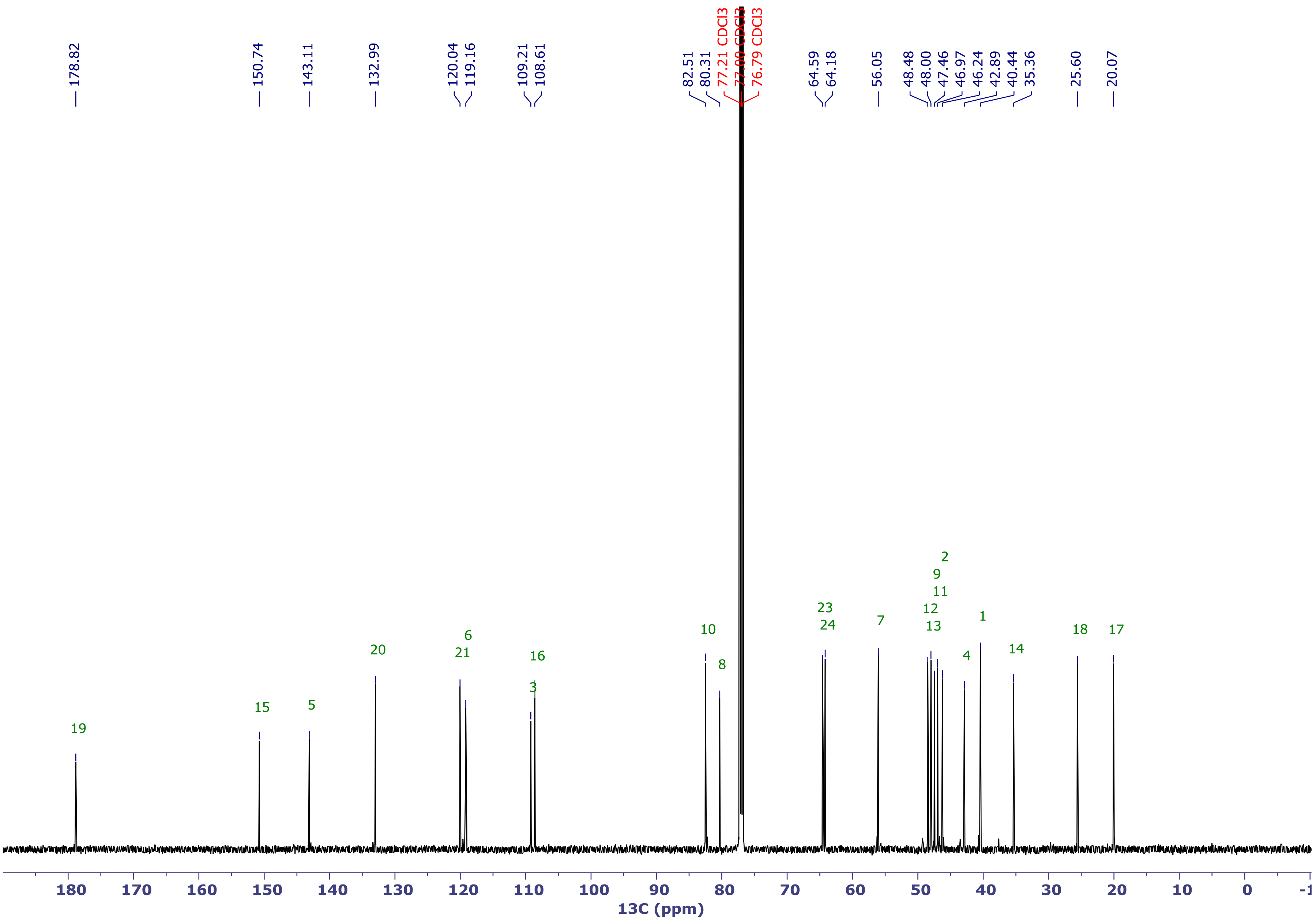

HSQC Spectrum of 13-*epi*-28

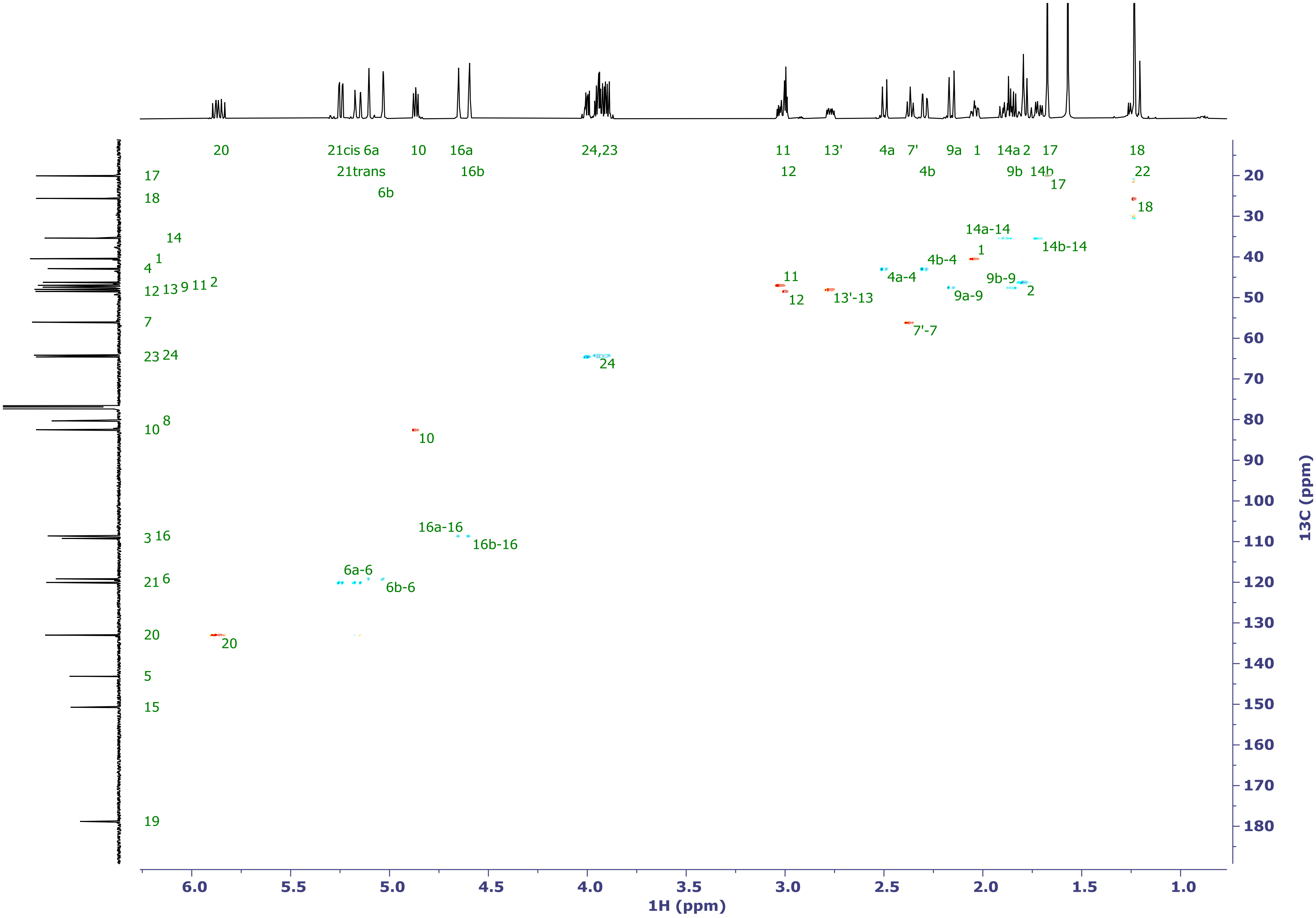

HMBC Spectrum of 13-*epi*-28

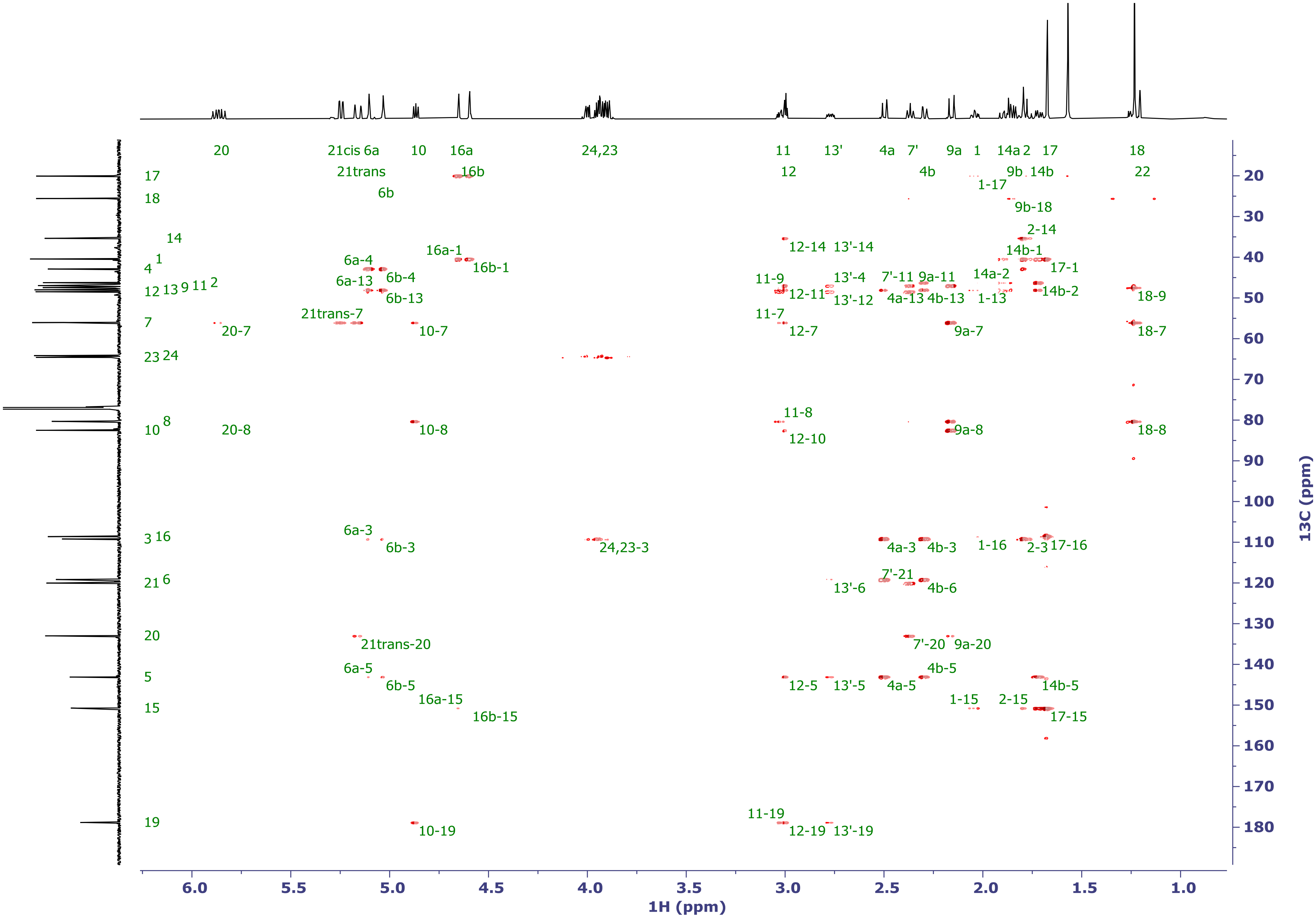

NOESY Spectrum of 13-*epi*-28

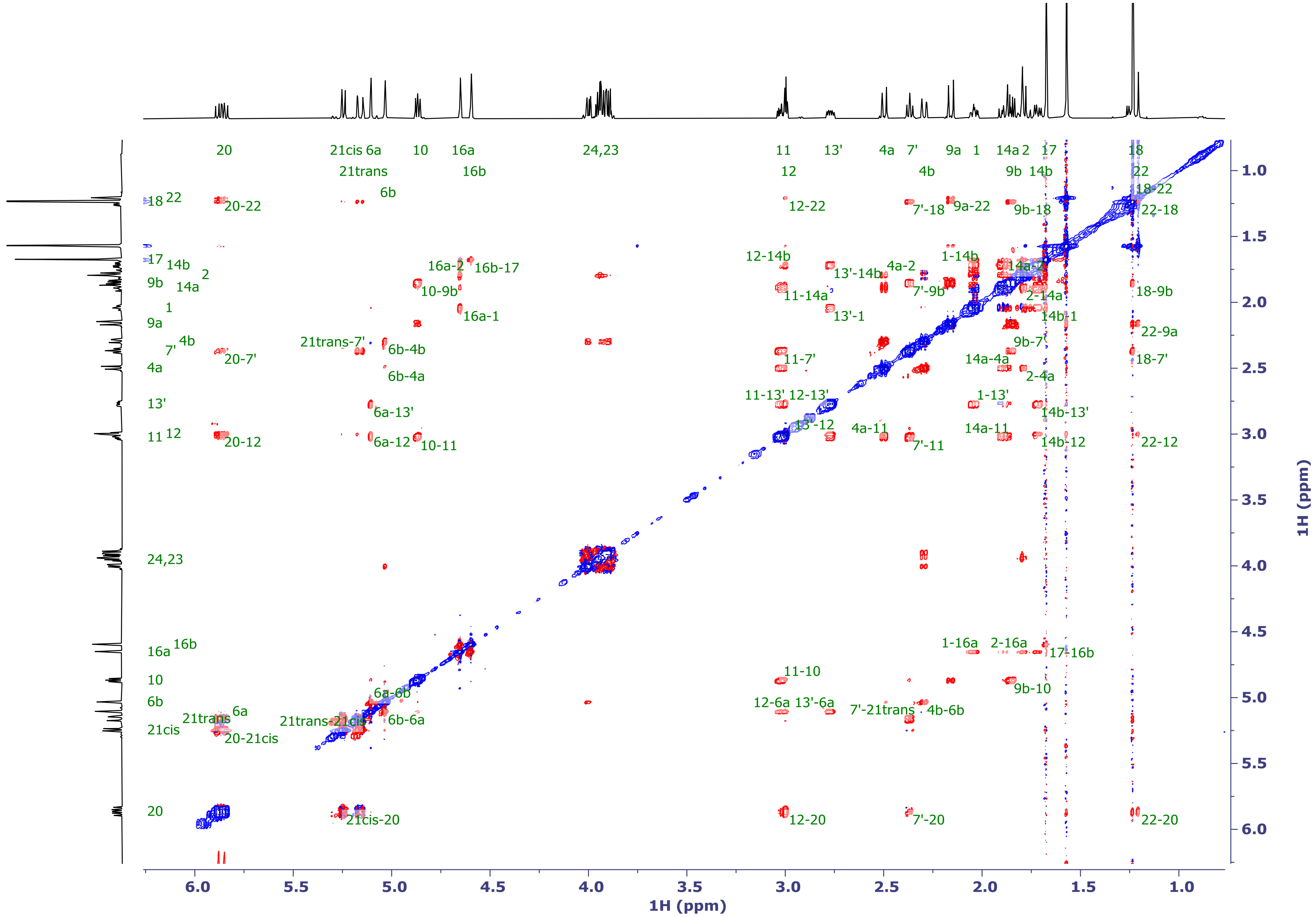

^{1}H NMR Spectrum of 29 (400 MHz, CDCl$_3$)

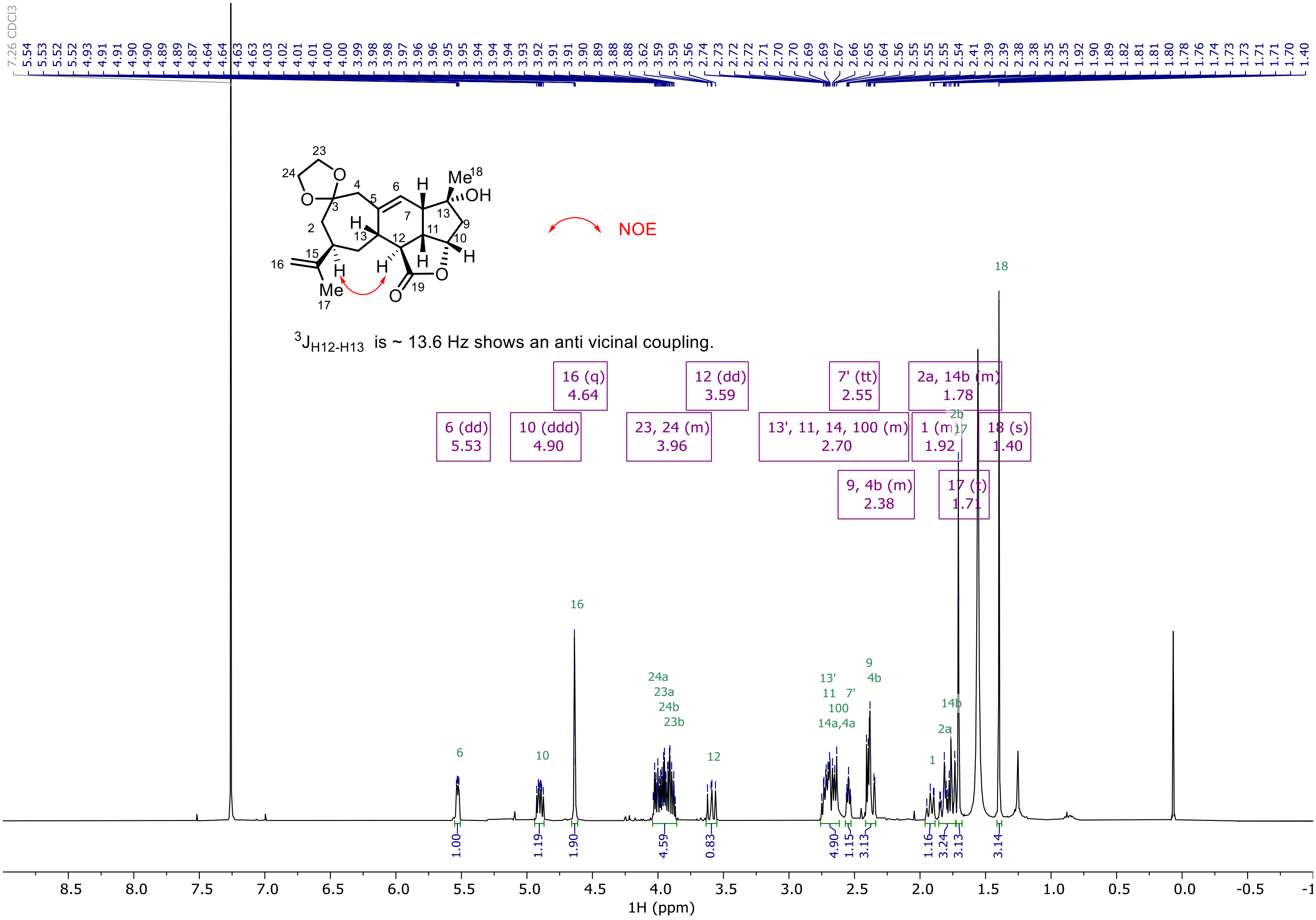

^{13}C NMR Spectrum of 29 (101 MHz, $CDCl_3$)

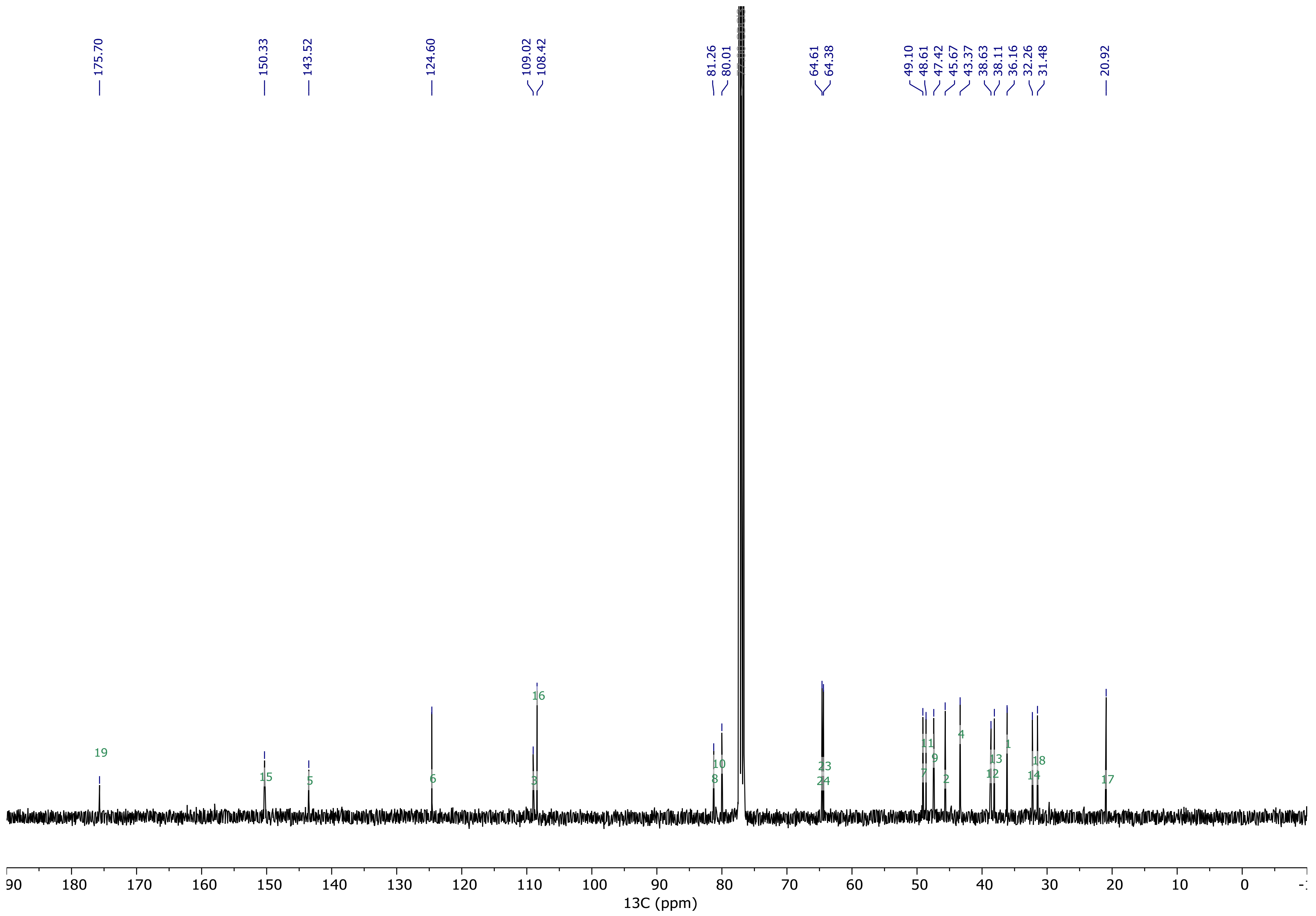

COSY Spectrum of 29

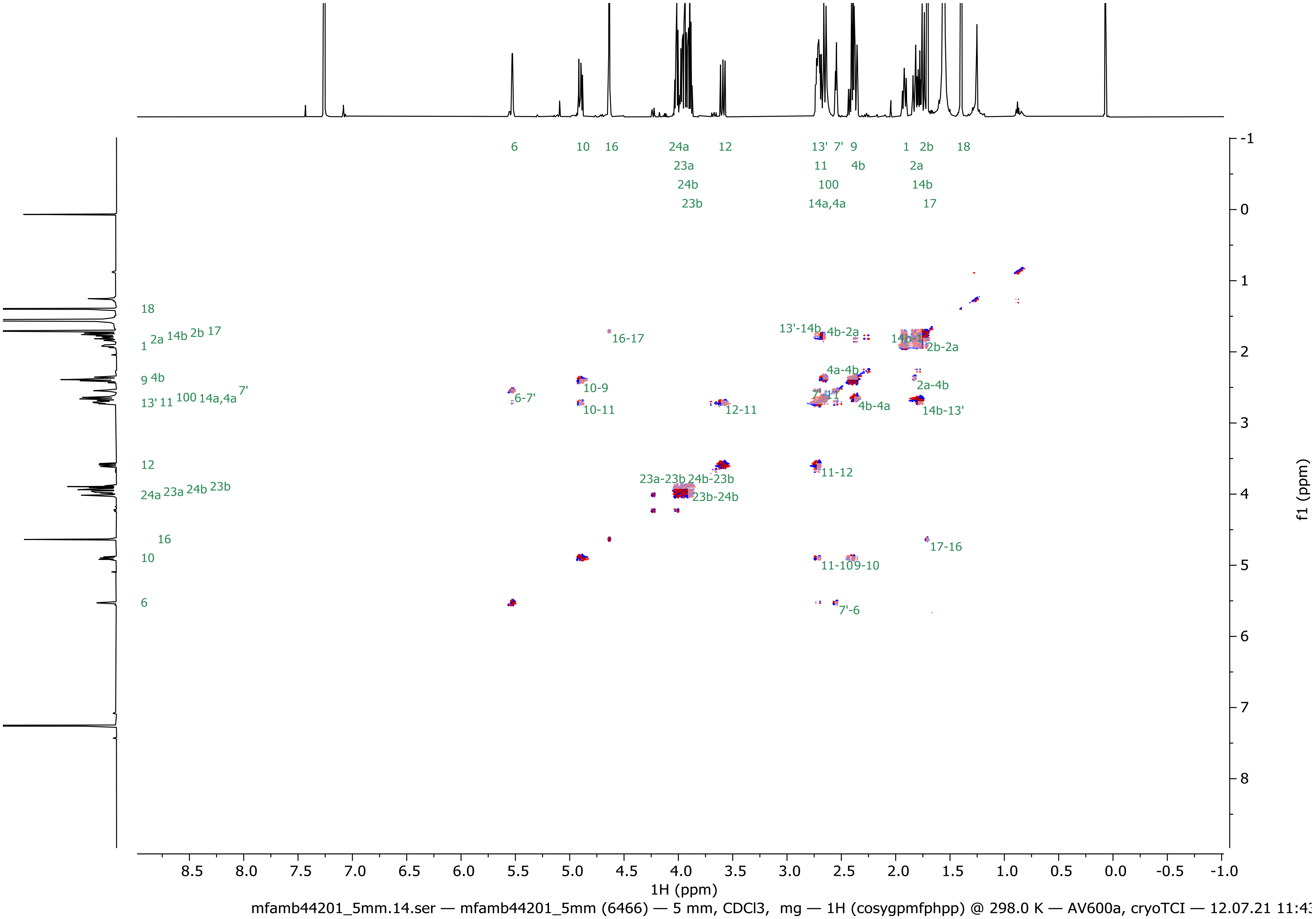

HSQC Spectrum of 29

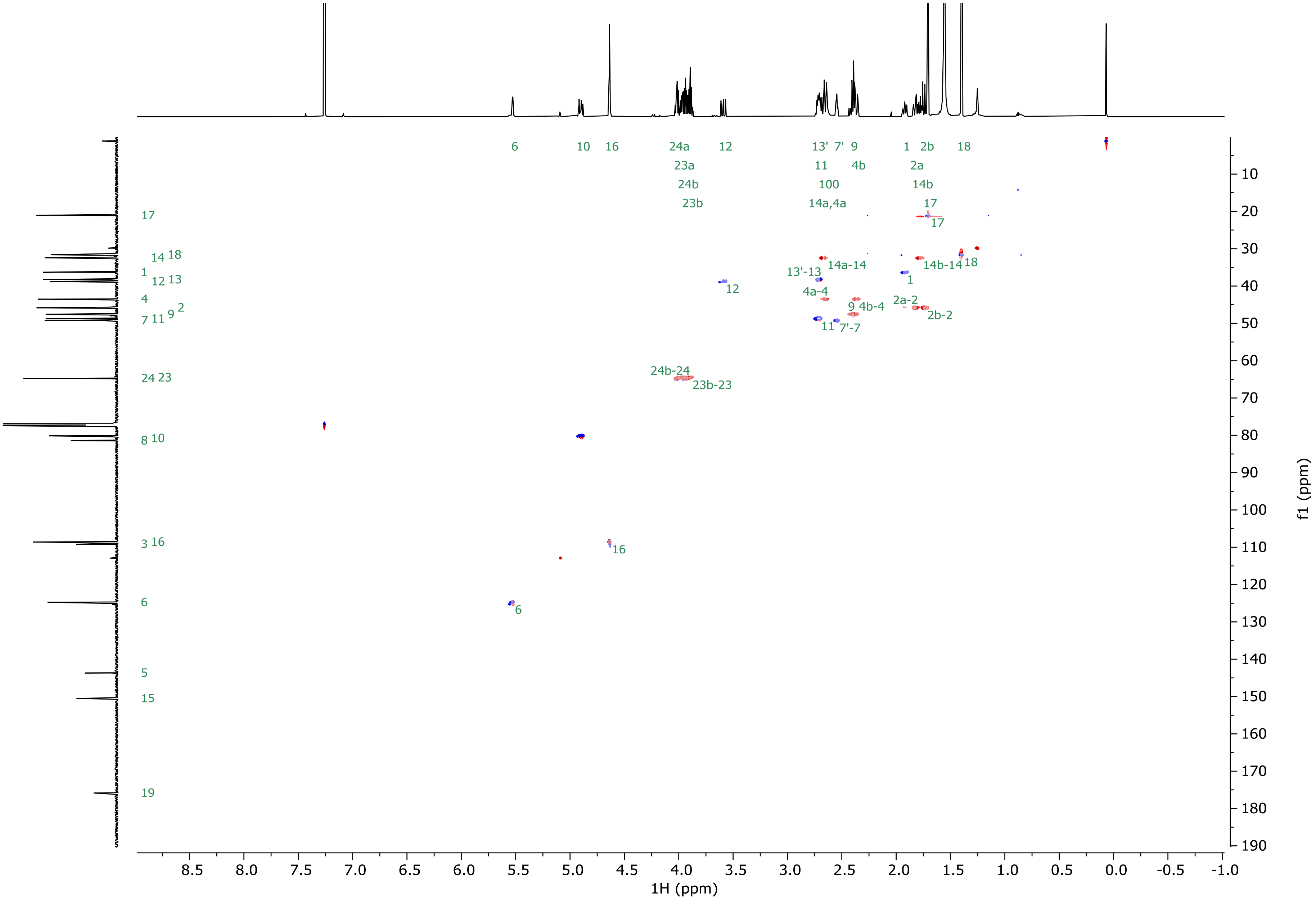

mfamb44201_5mm.12.ser — mfamb44201_5mm (6466) — 5 mm, CDCl3, mg — 1H-13C (hsqcedetgpsisp2.4) @ 298.0 K — AV600a, cryoTCI — 12.07.21 10:4

HMBC Spectrum of 29

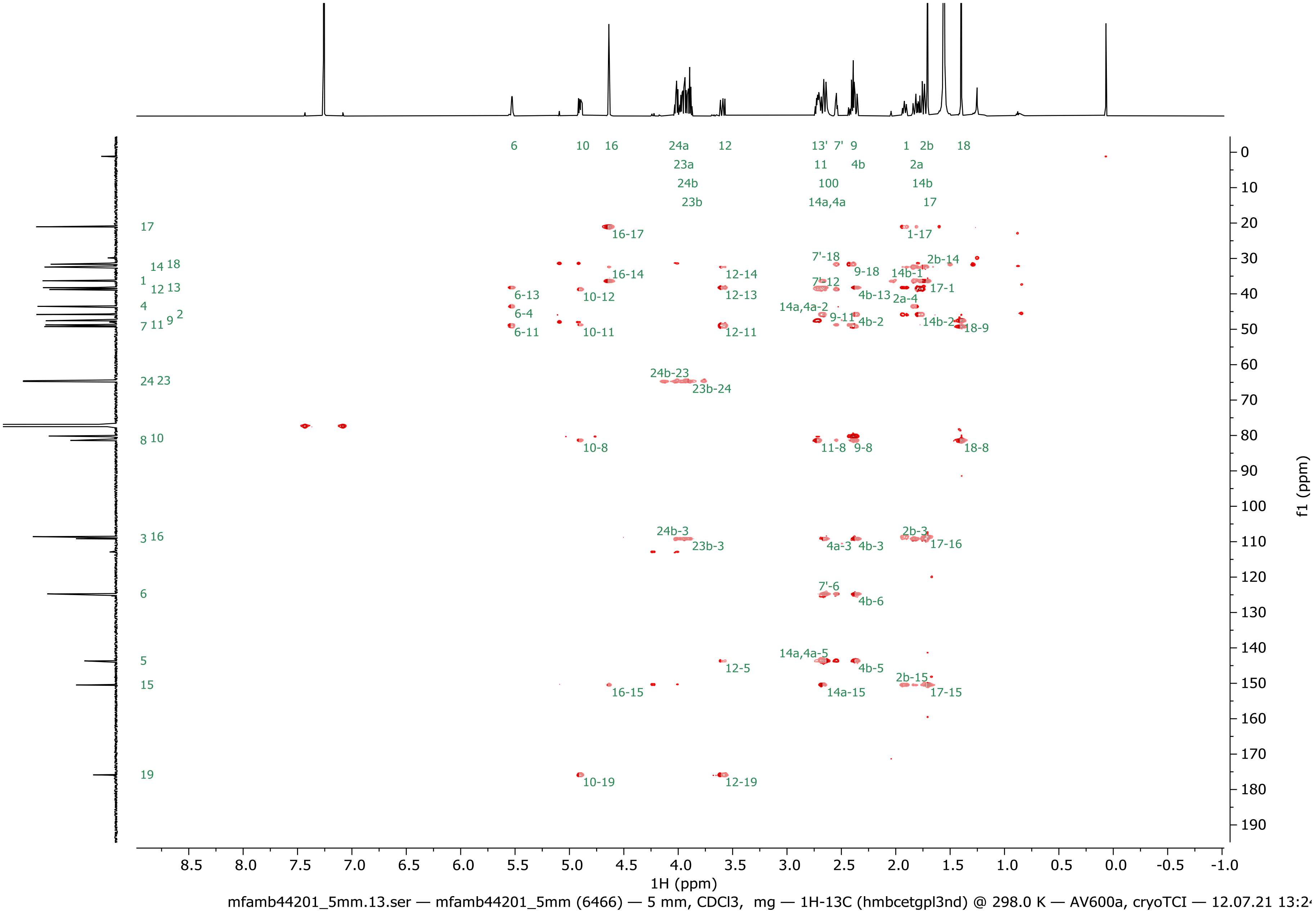

NOESY Spectrum of 29

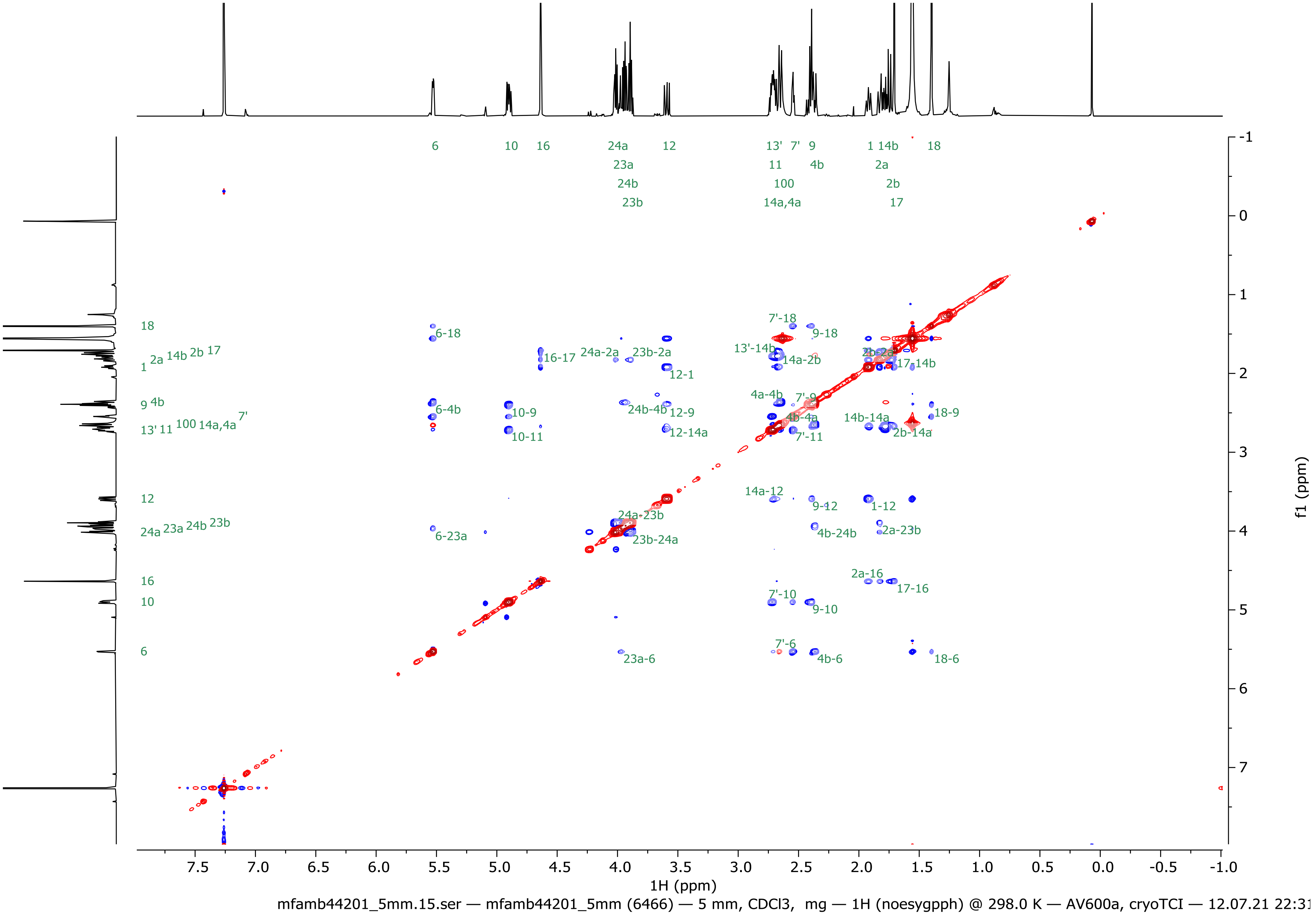

ROESY Spectrum of 29

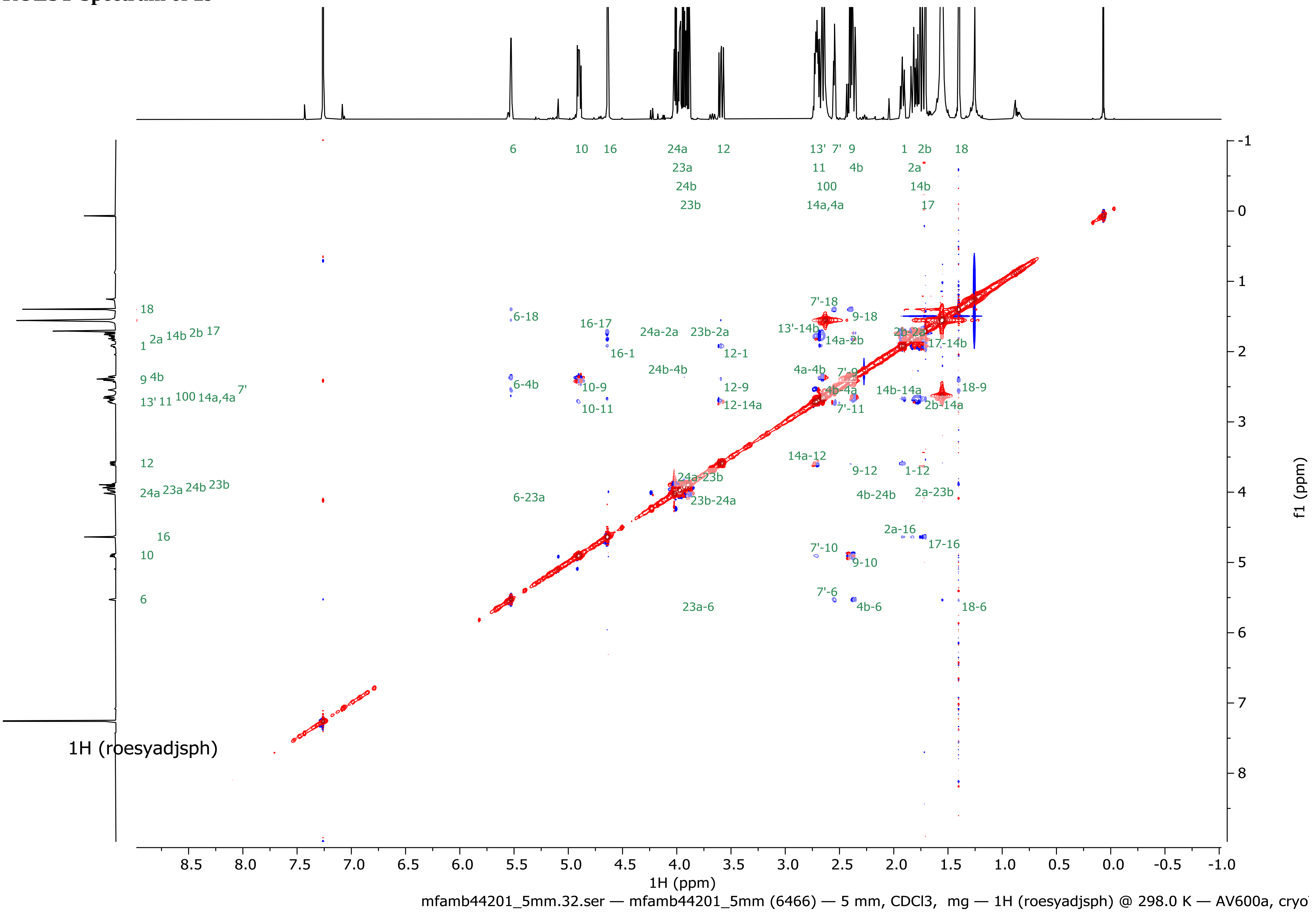

^{1}H NMR Spectrum of 31 (400 MHz, $CDCl_3$)

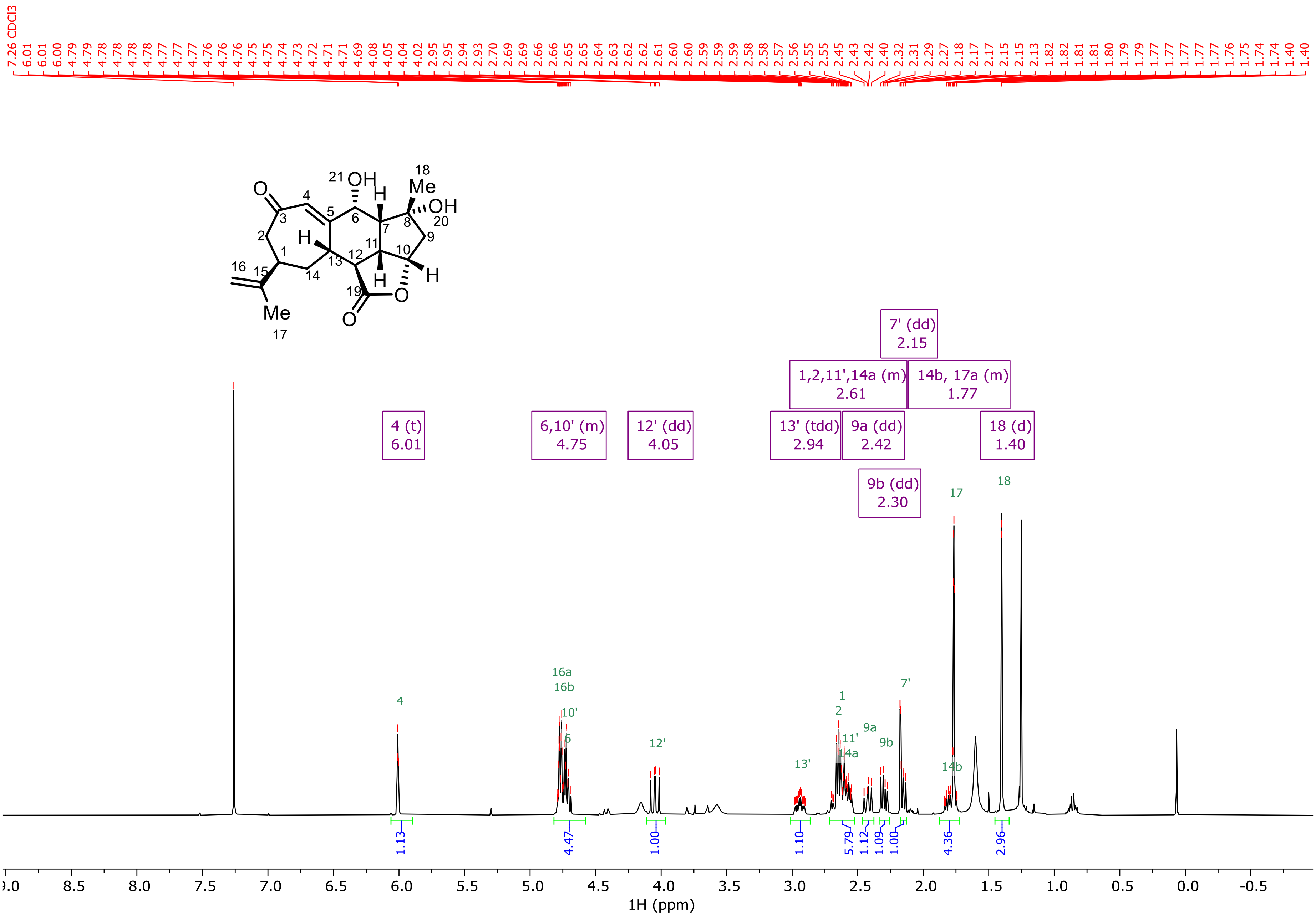

^{13}C NMR Spectrum of 31 (101 MHz, $CDCl_3$)

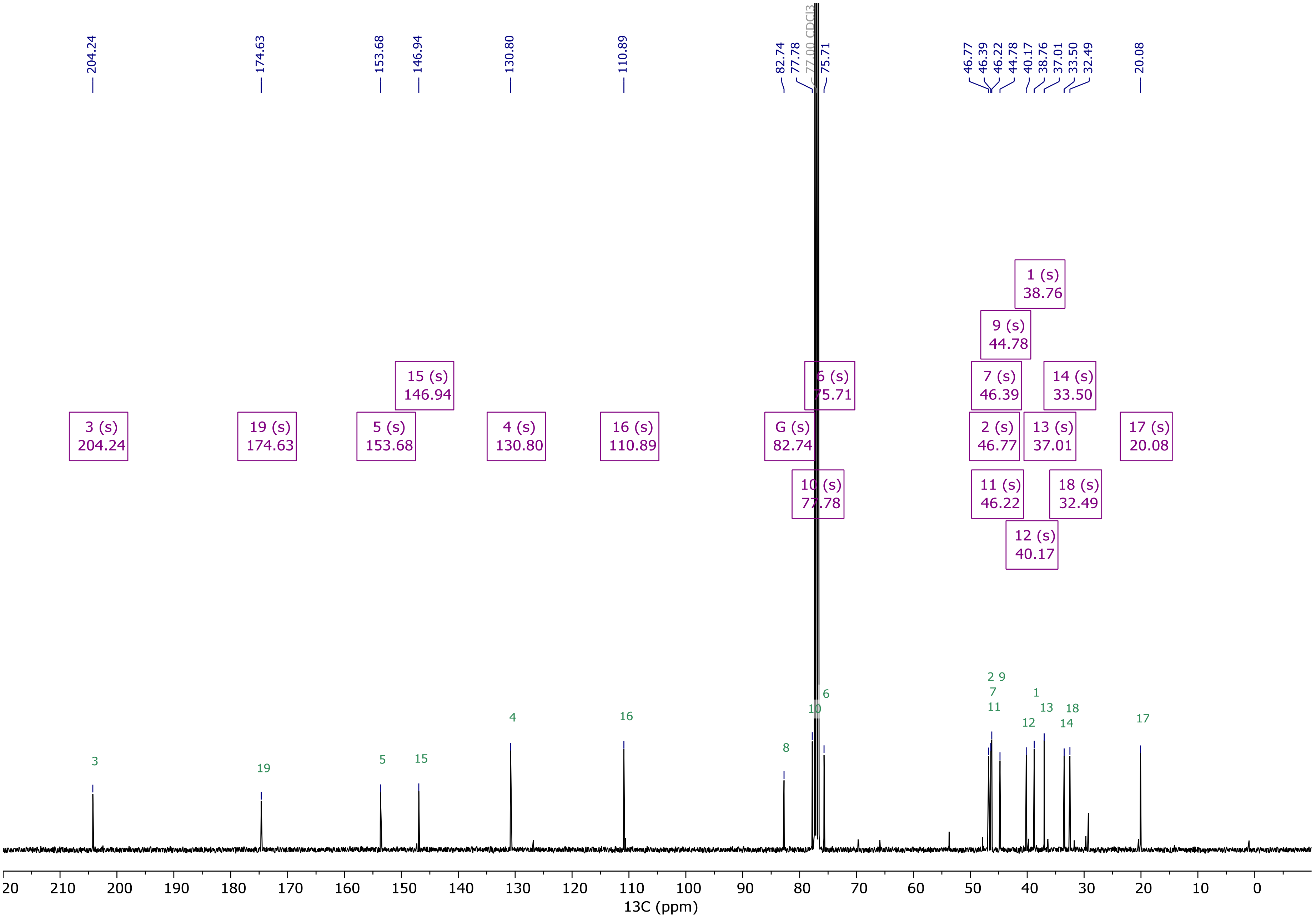

COSY Spectrum of 31

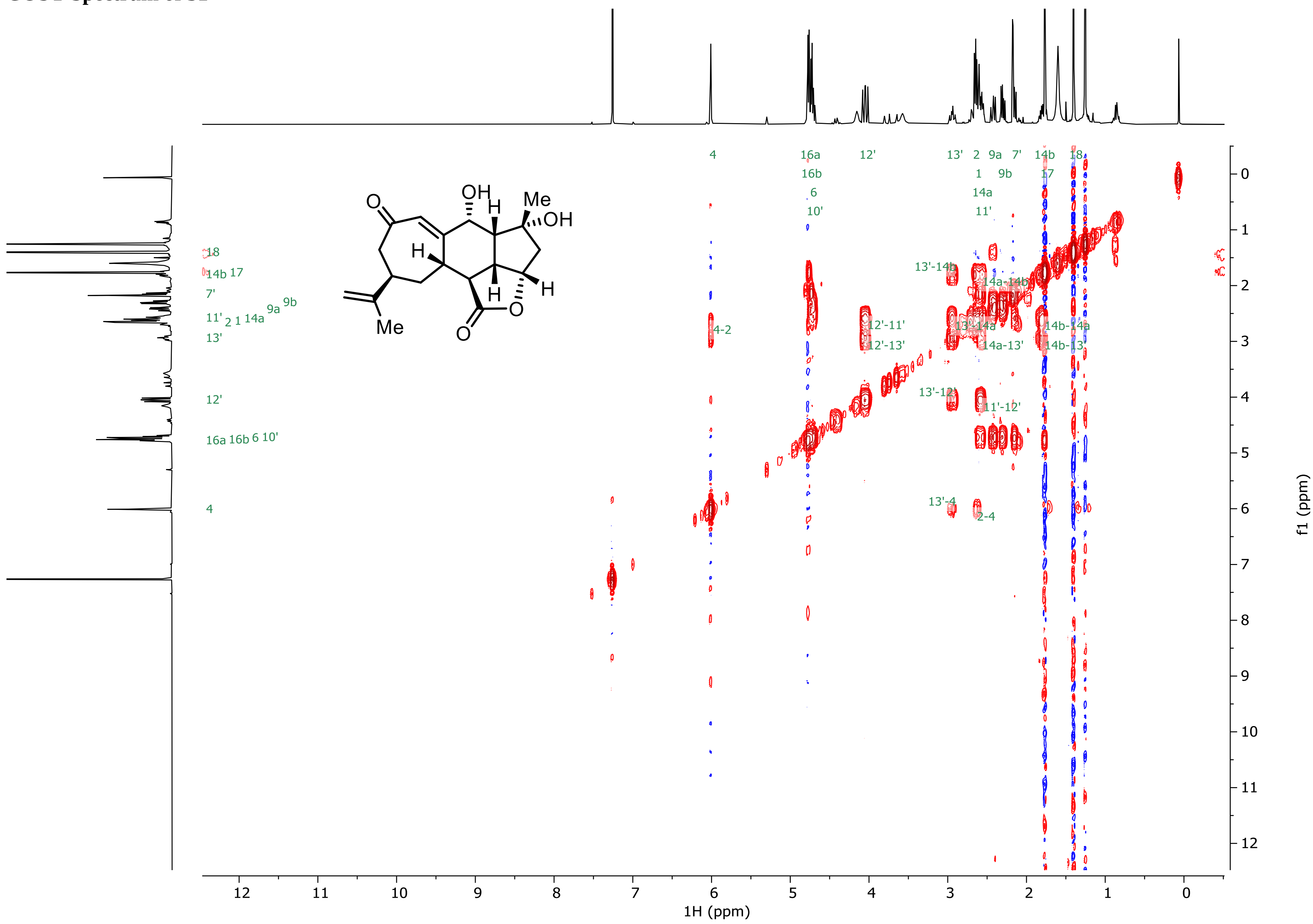

HSQC Spectrum of 31

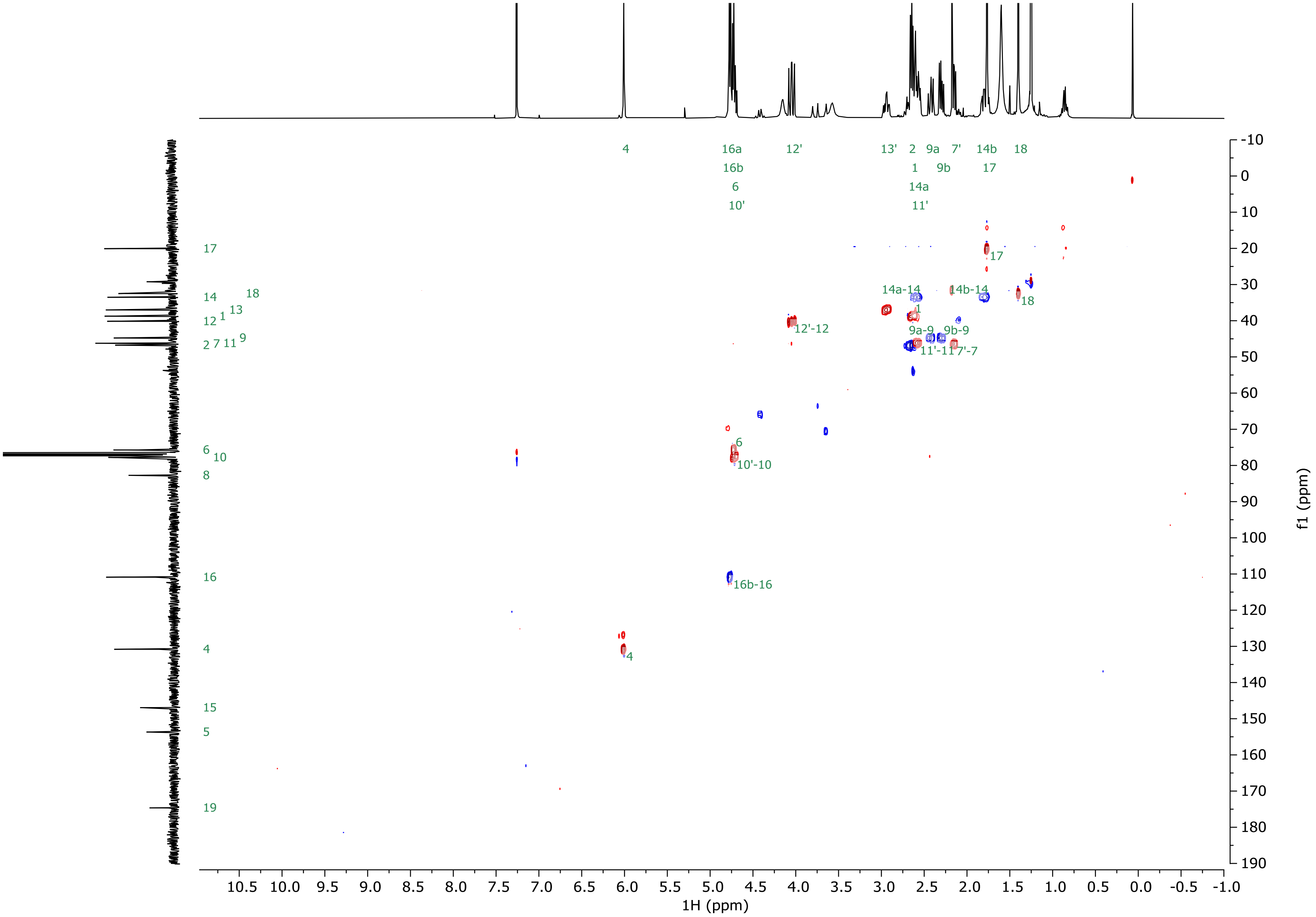

HMBC Spectrum of 31

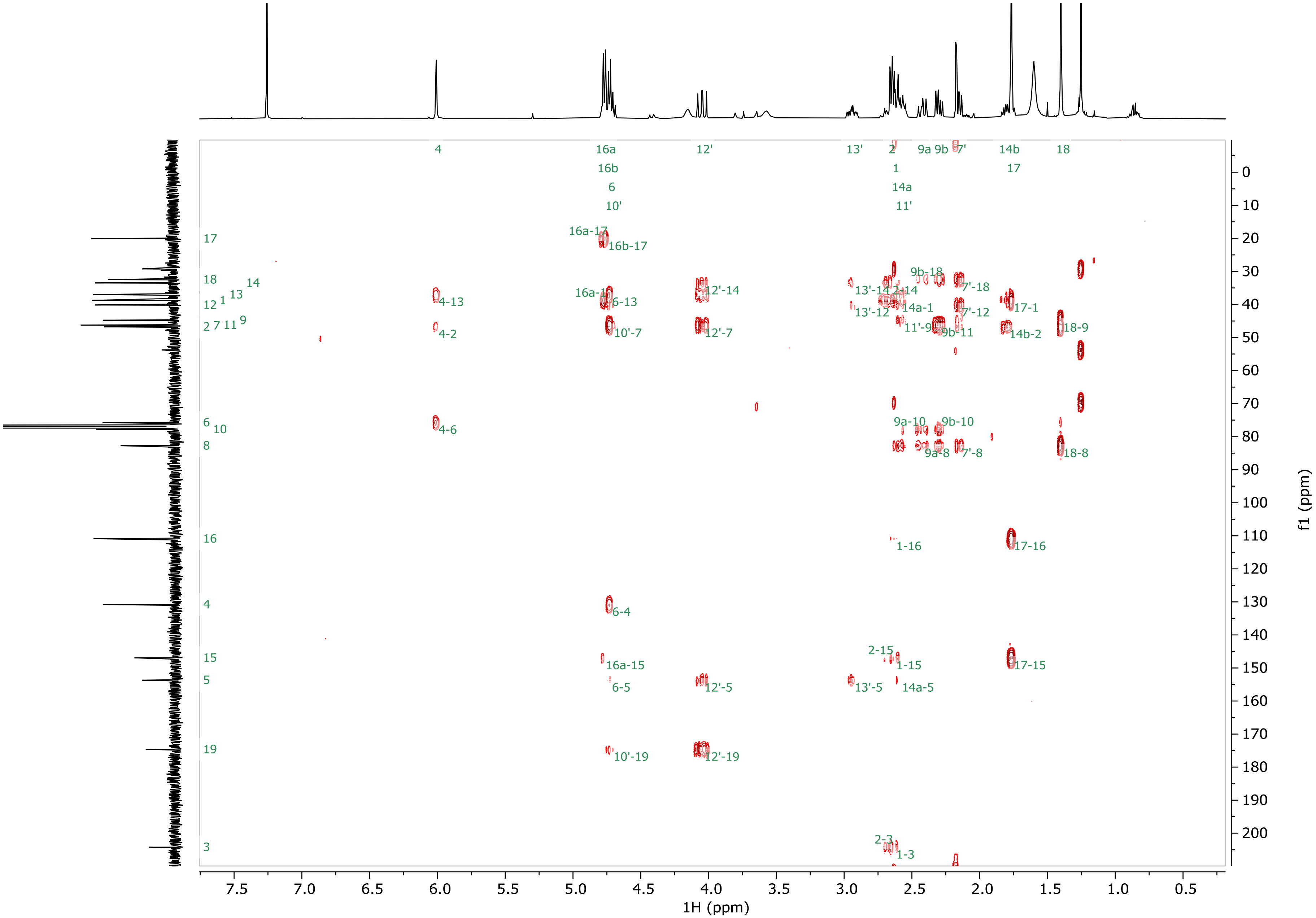

^{1}H NMR Spectrum of 12-*epi*-1 (600 MHz, $CDCl_3$)

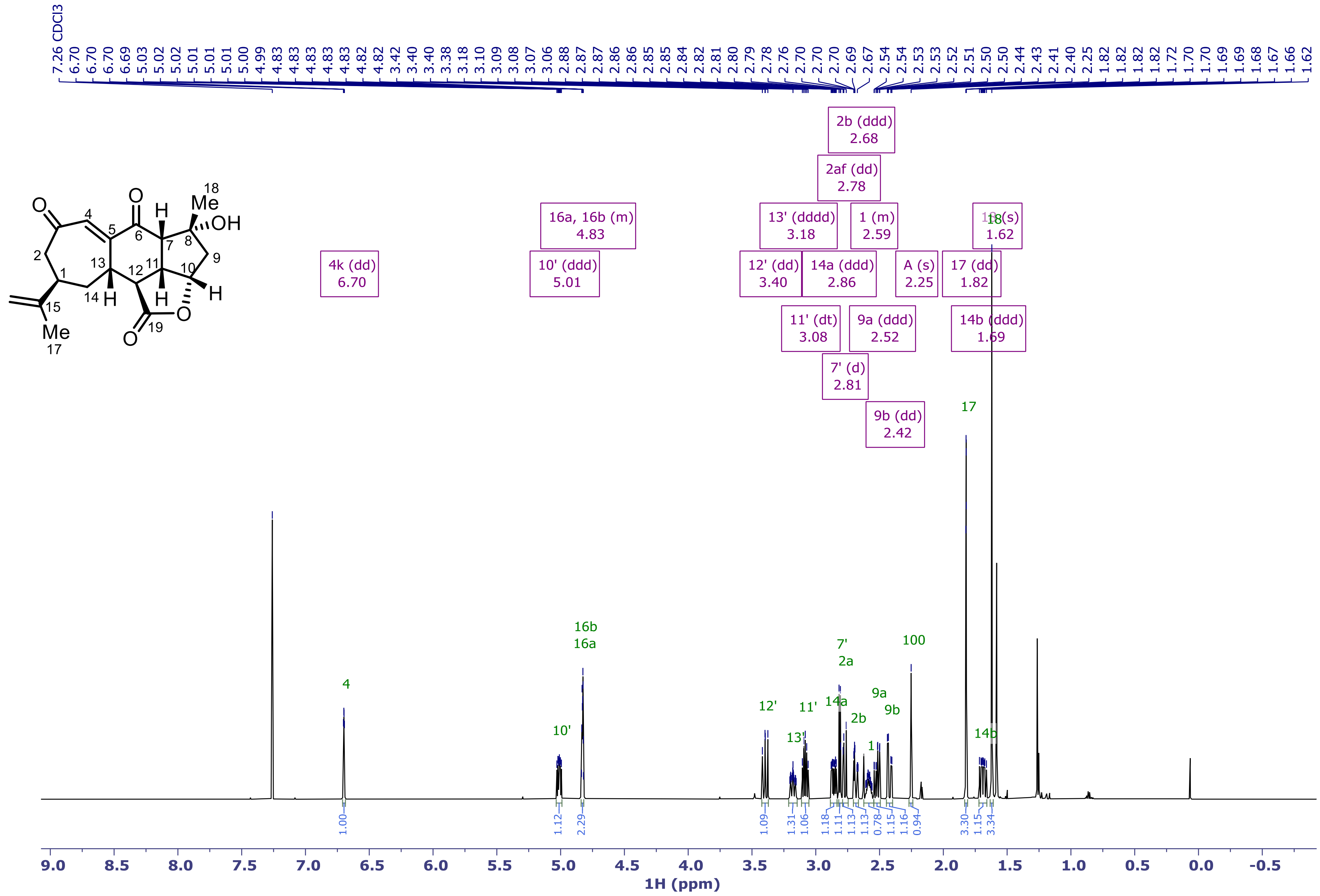

^{13}C NMR Spectrum of 12-*epi*-1 (151 MHz, $CDCl_3$)

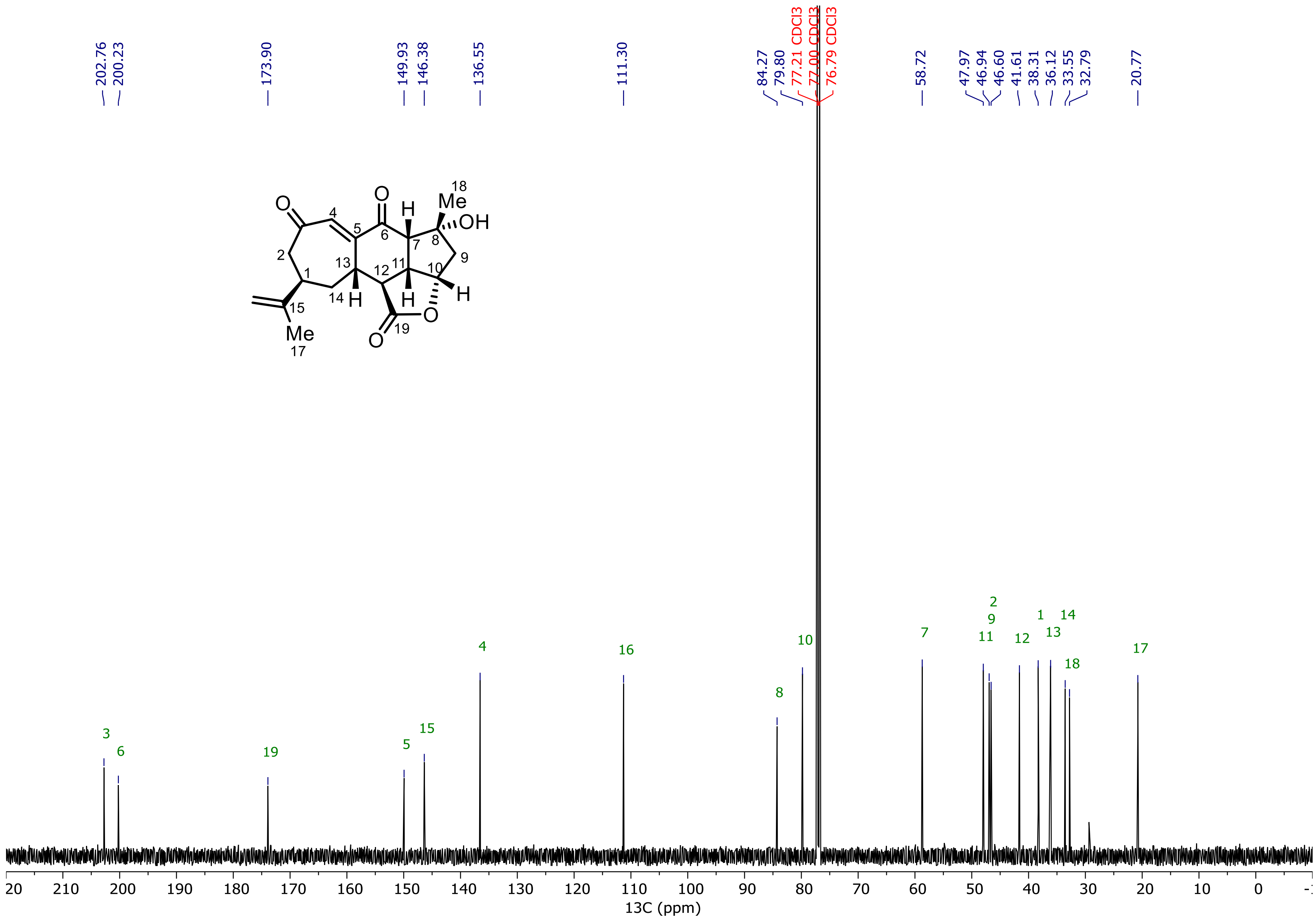

COSY Spectrum of 12-*epi*-1

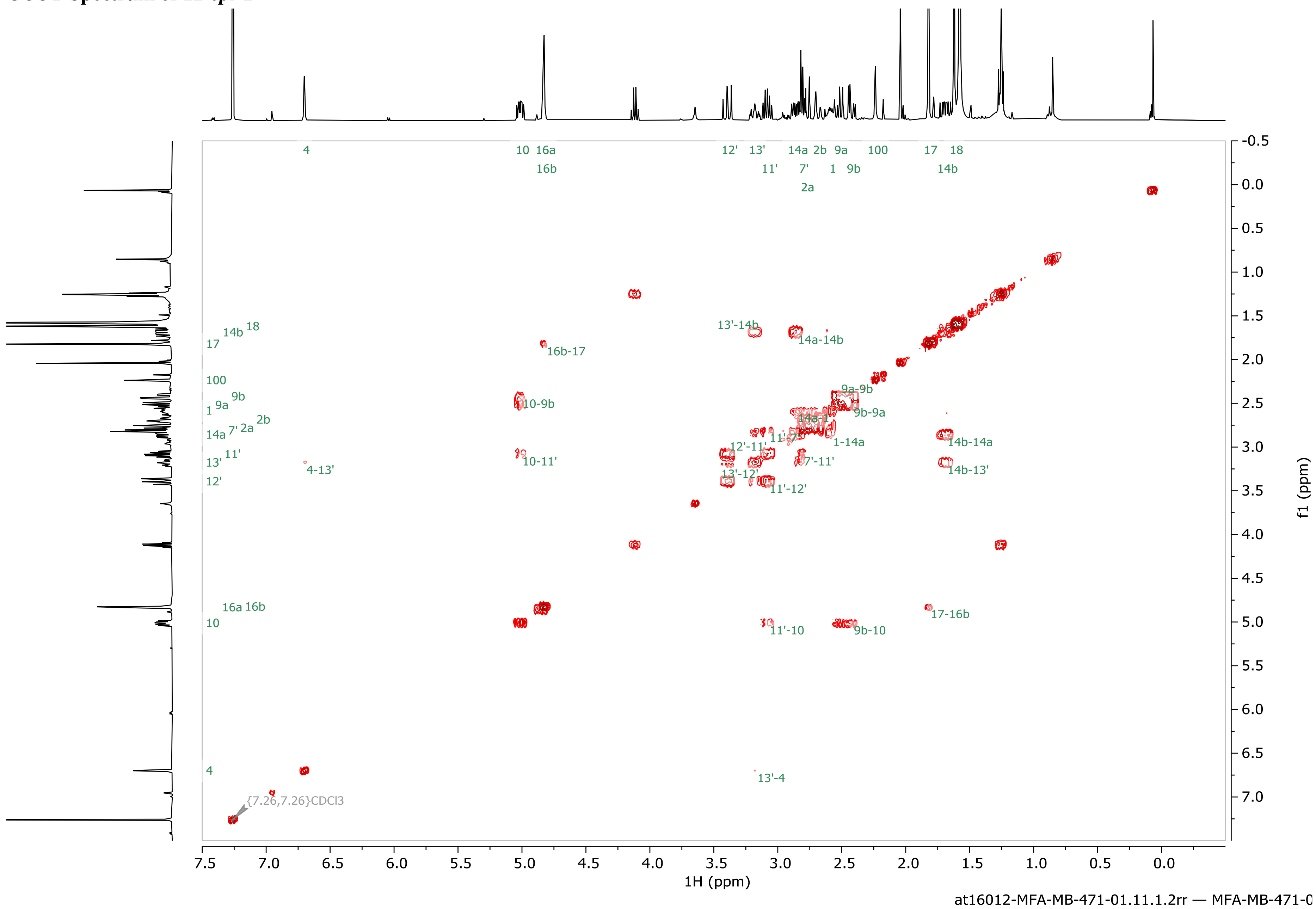

HSQC Spectrum of 12-*epi*-1

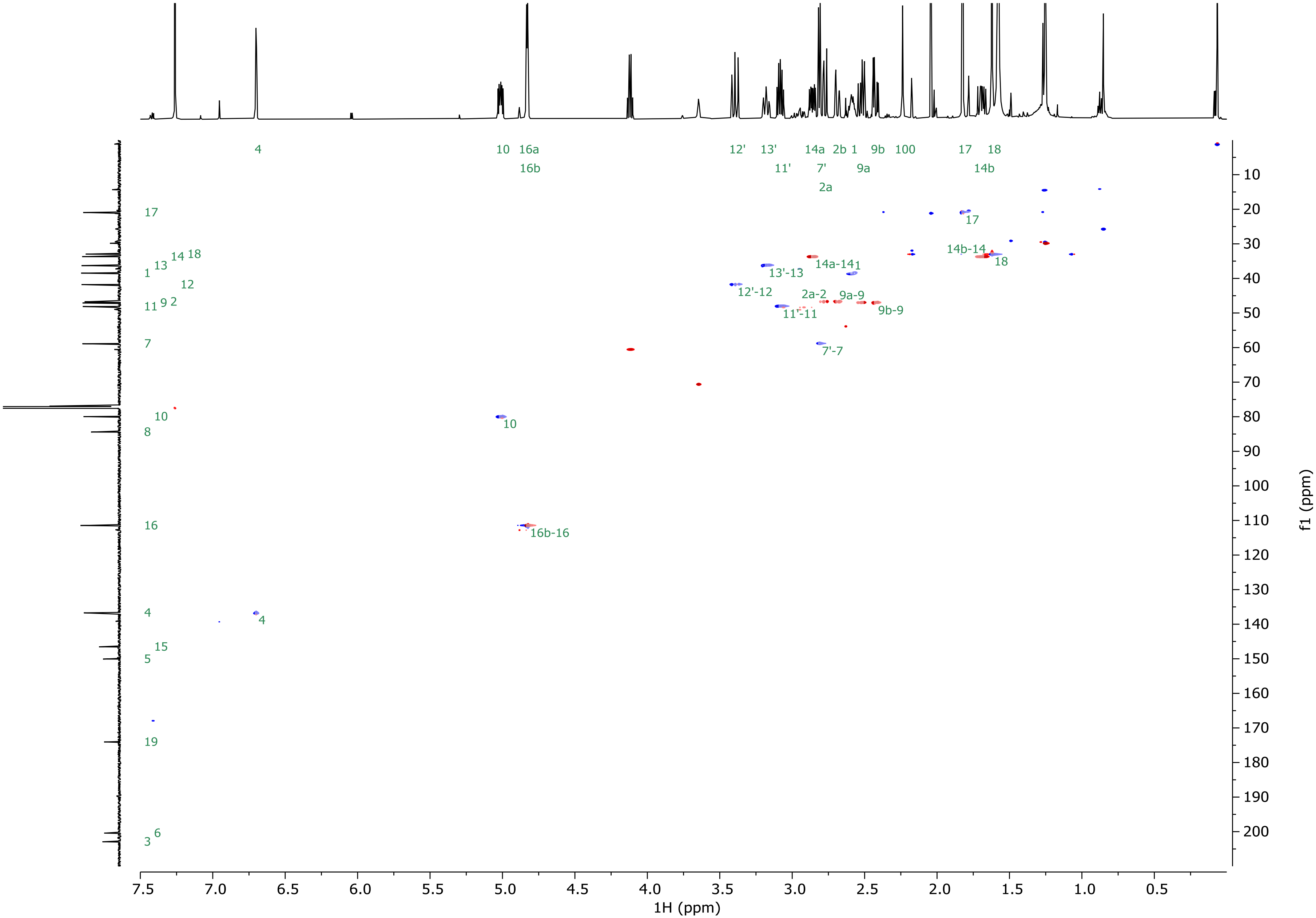

HMBC Spectrum of 12-*epi*-1

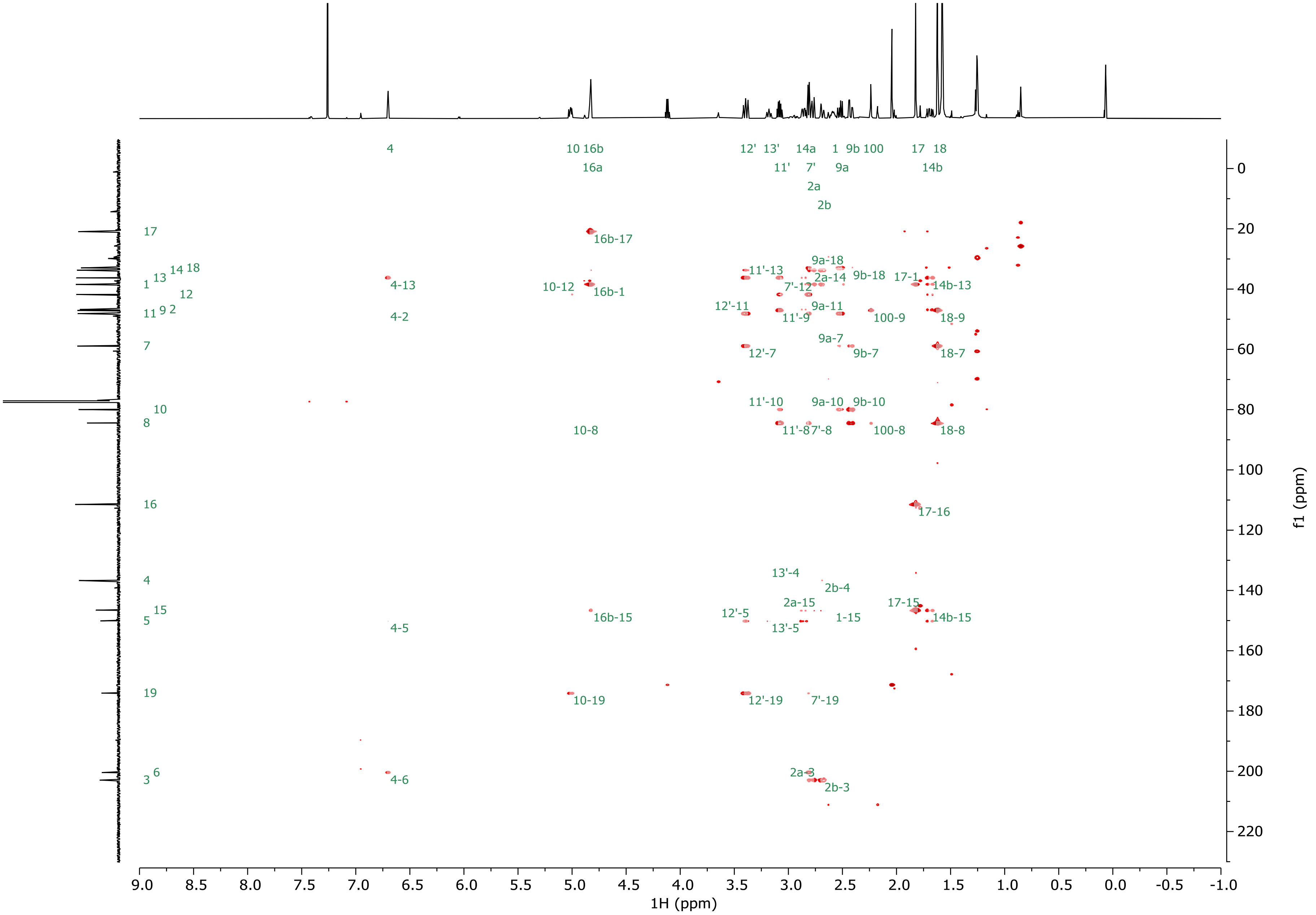

NOESY Spectrum of 12-*epi*-1

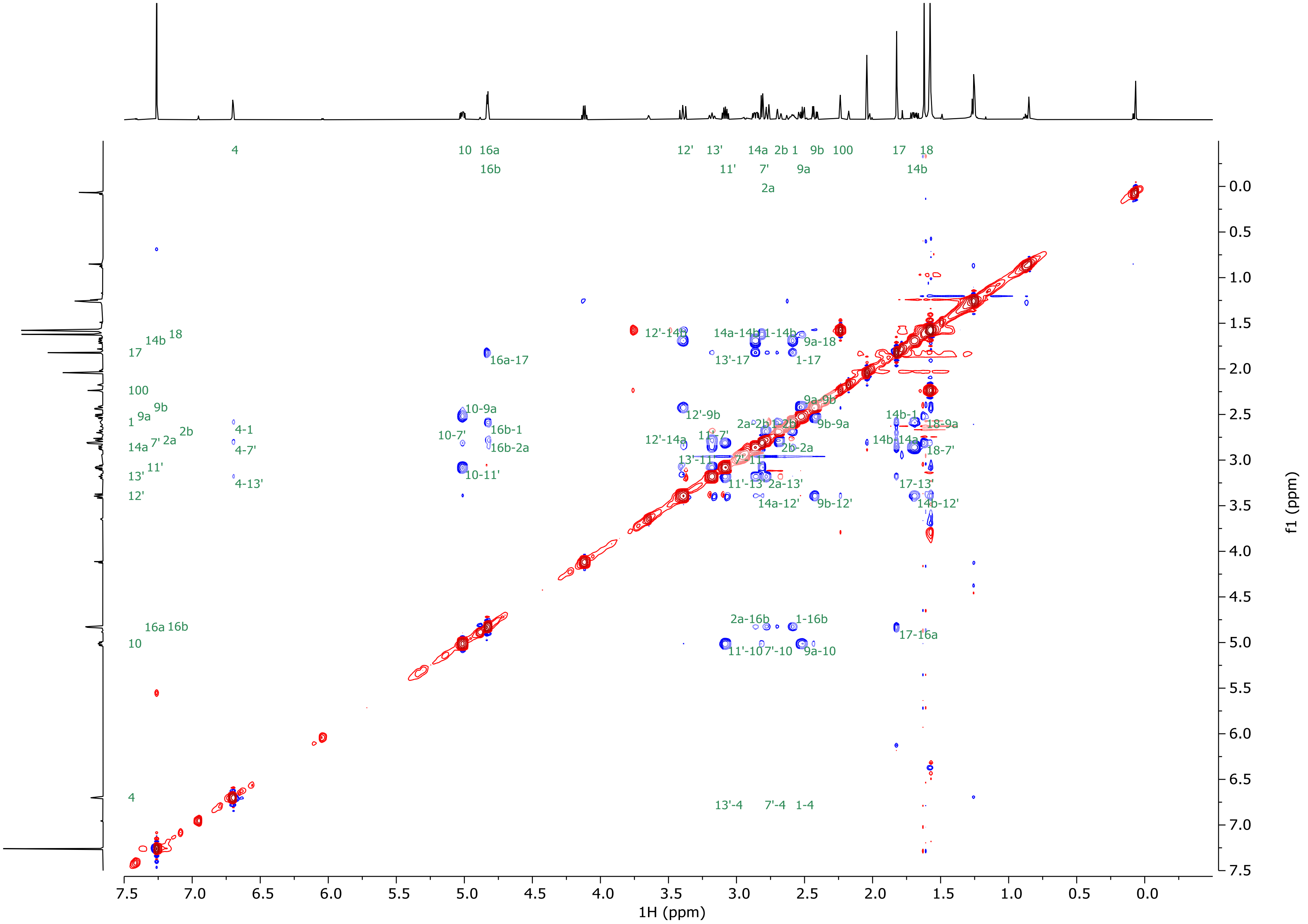

^{1}H NMR Spectrum of Nominal Scabrolide B (1) (600 MHz, $CDCl_3$)

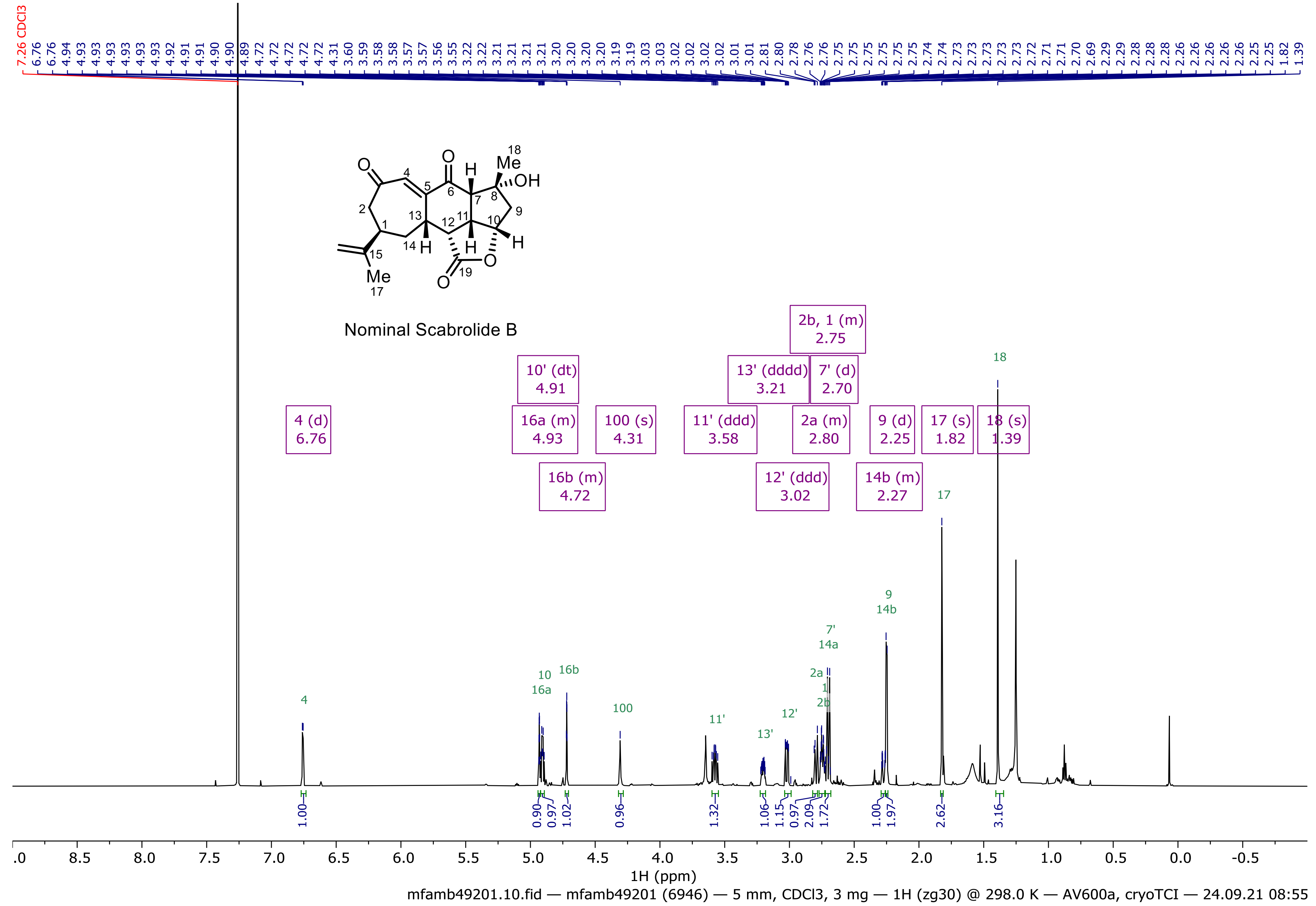

^{13}C NMR Spectrum of Nominal Scabrolide B (1) (151 MHz, $CDCl_3$)

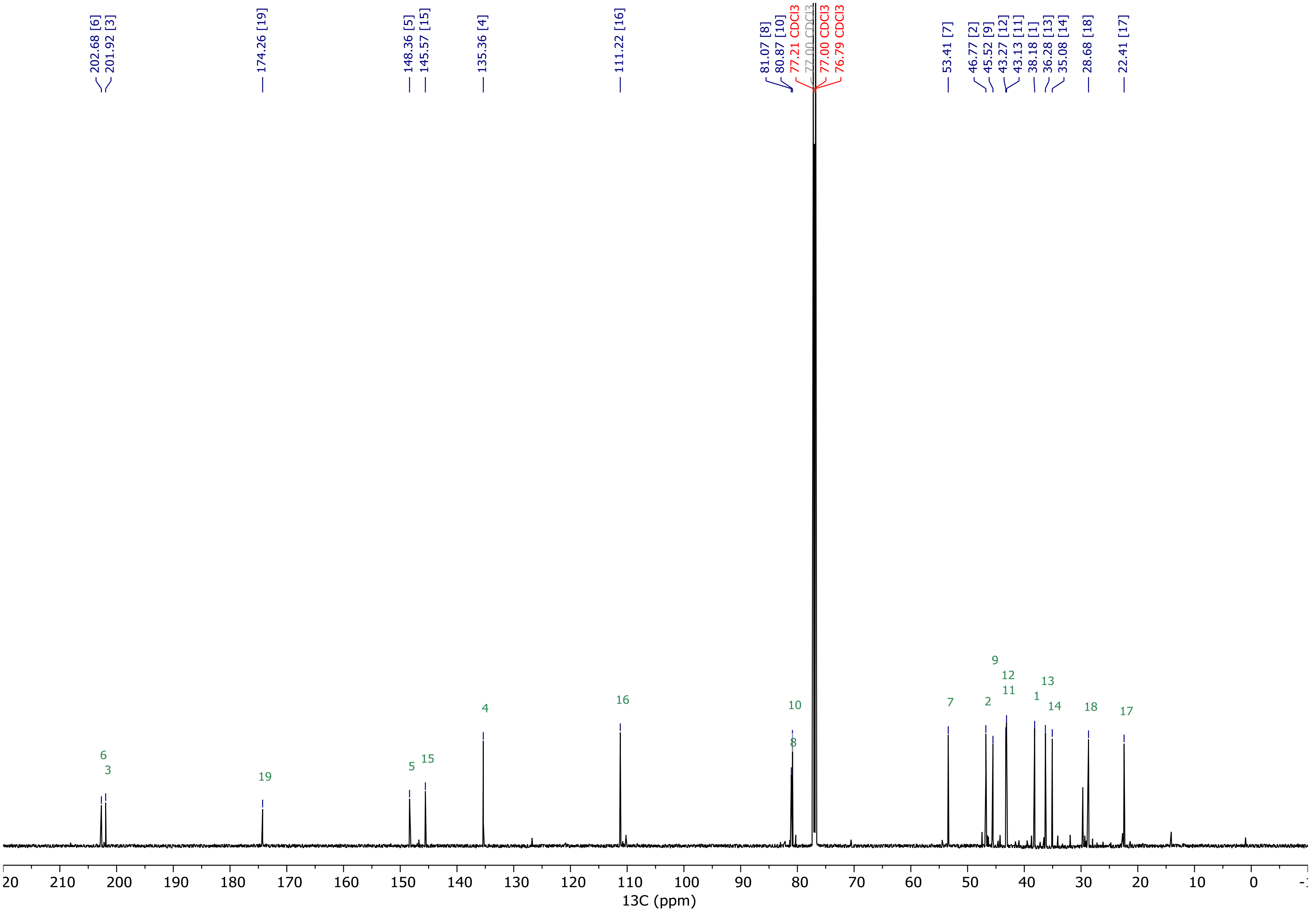

COSY spectrum of Nominal Scabrolide B (1)

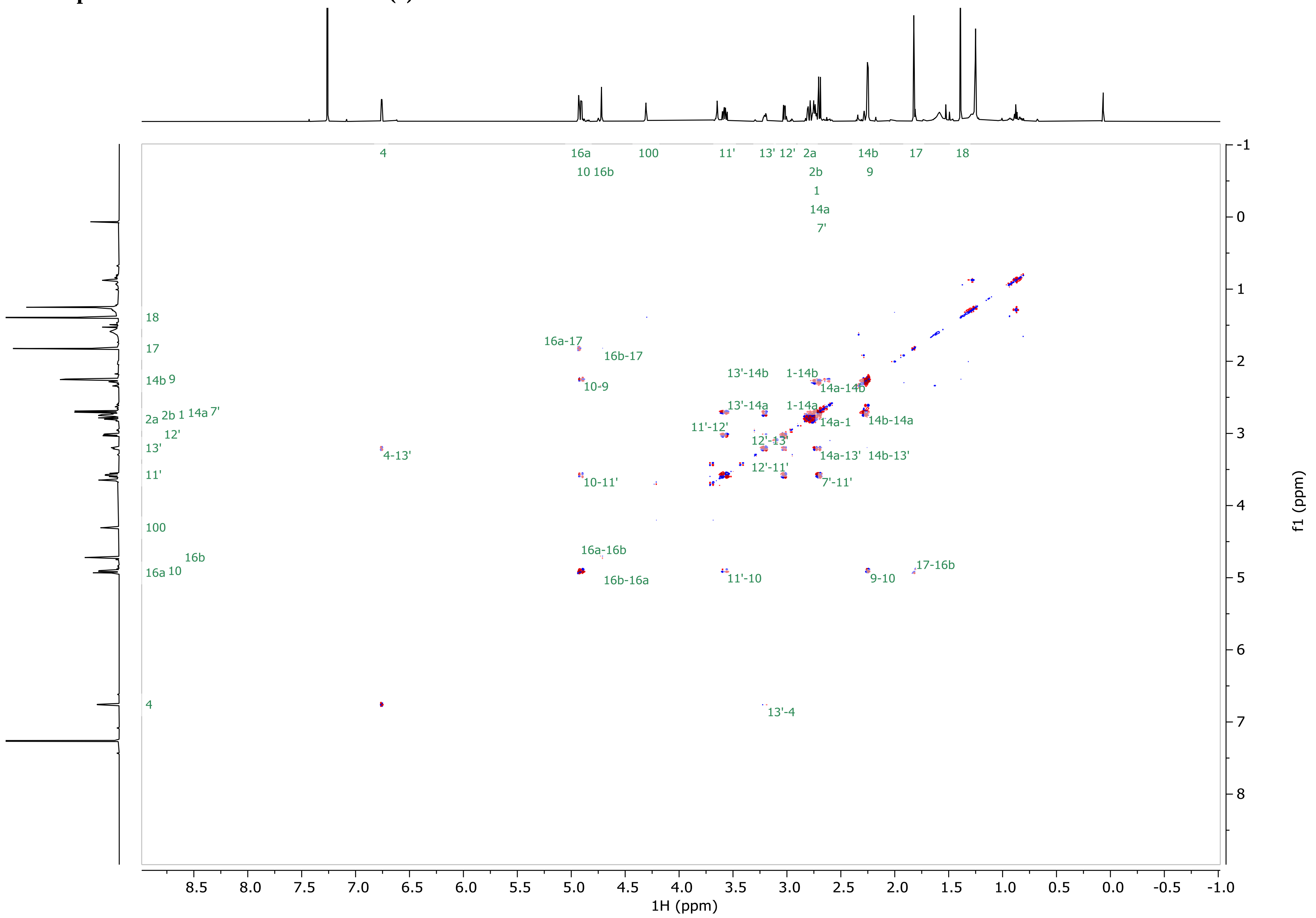

HSQC spectrum of Nominal Scabrolide B (1)

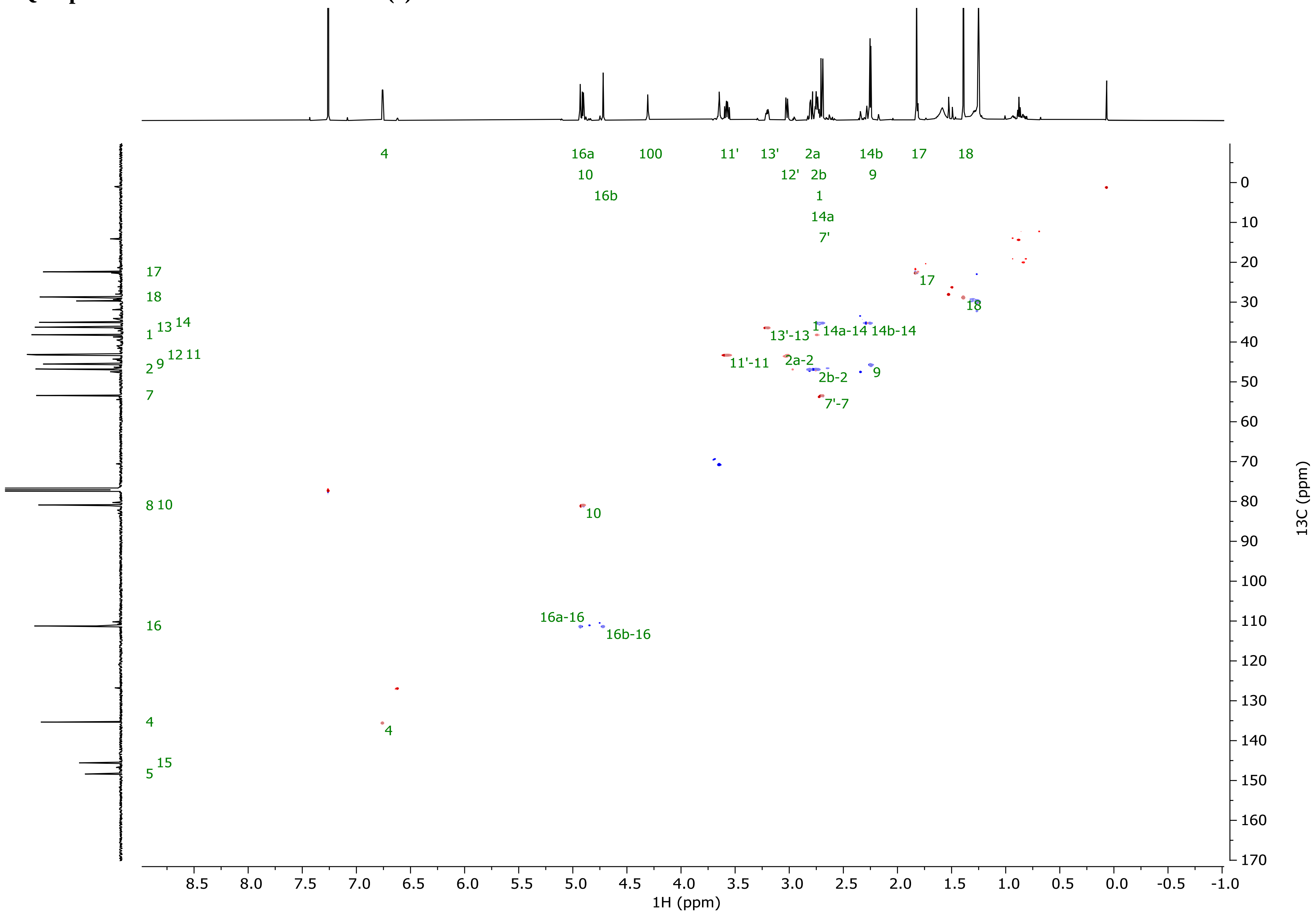

HMBC spectrum of Nominal Scabrolide B (1)

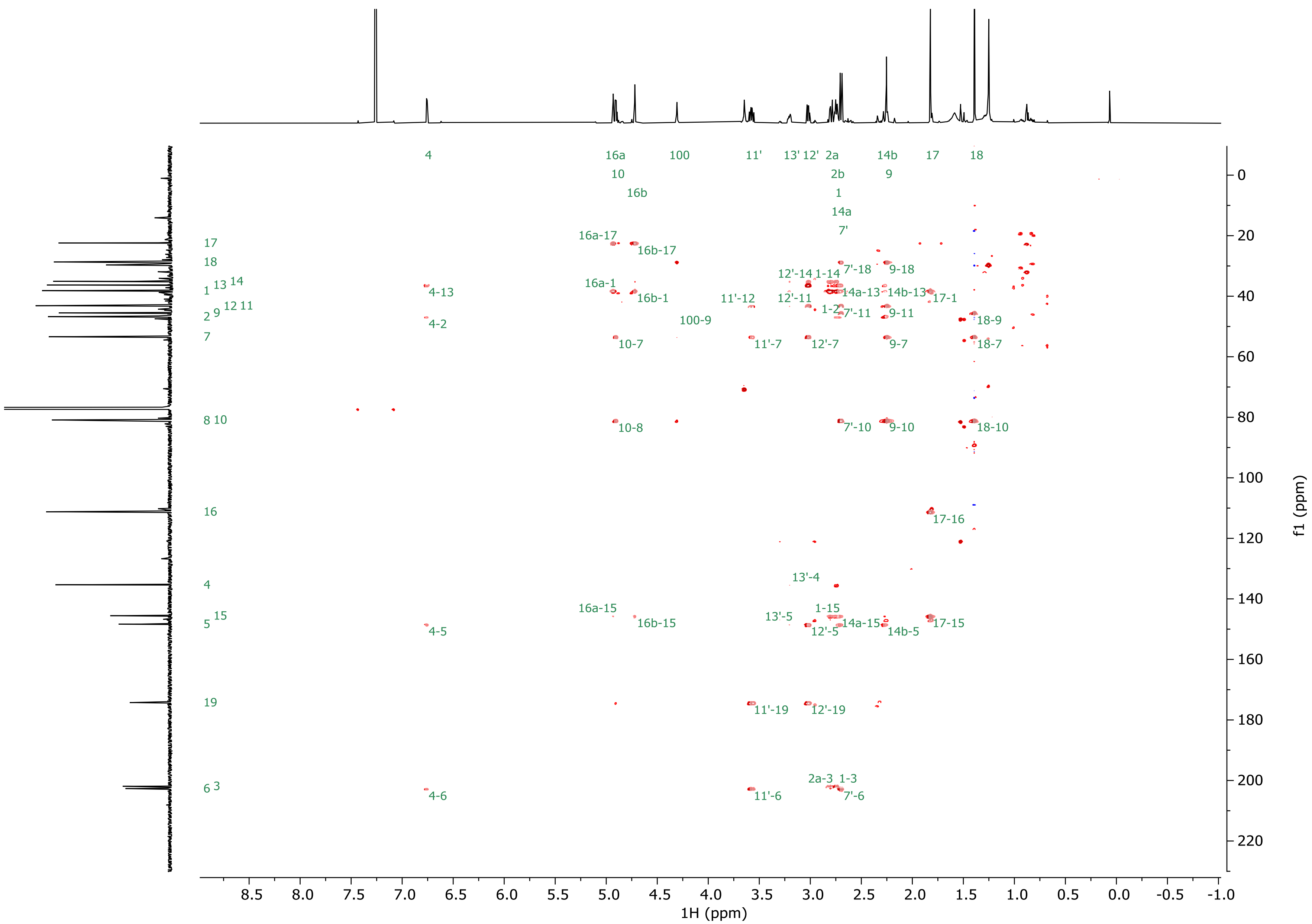

NOESY spectrum of Nominal Scabrolide B (1)

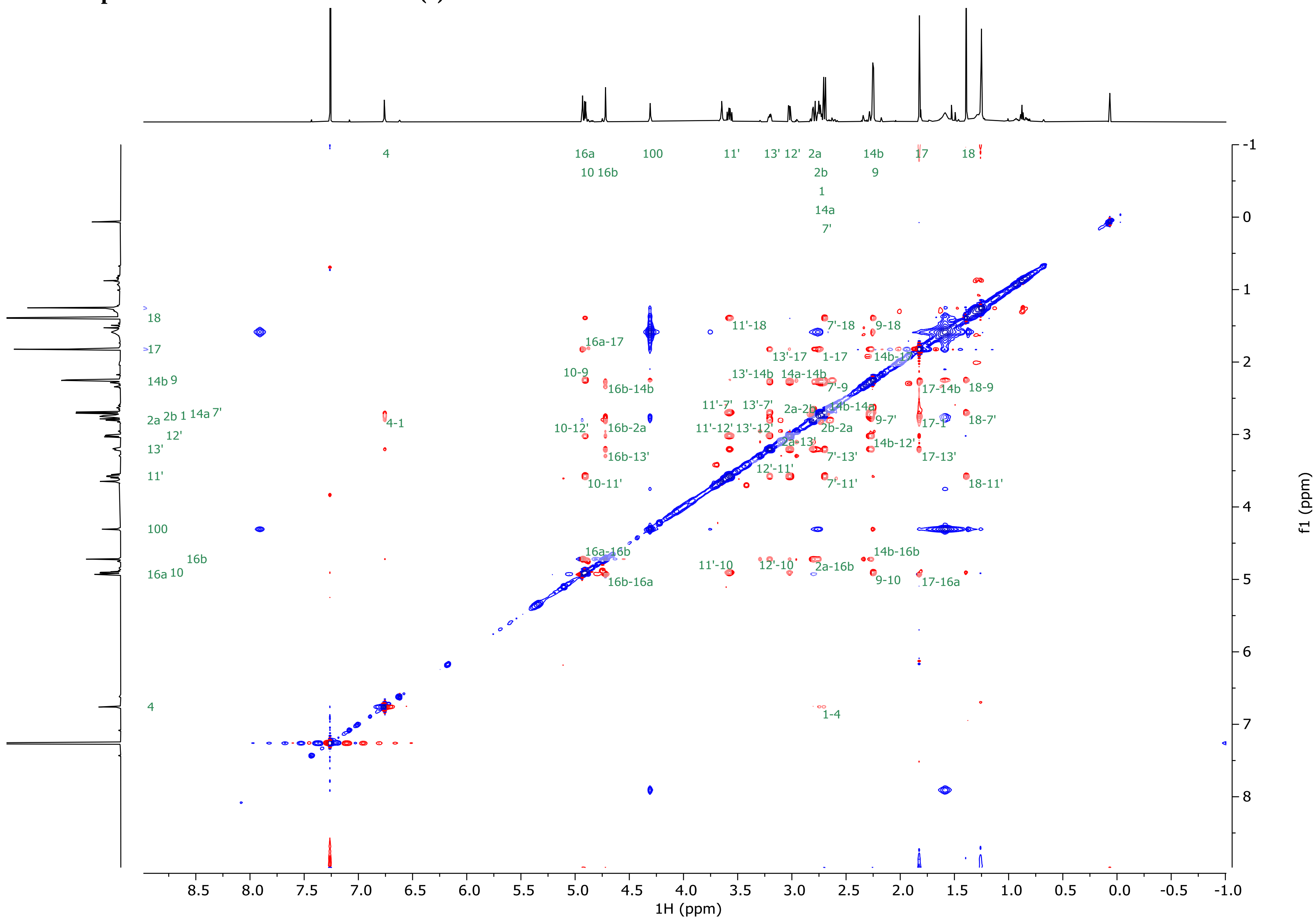

NMR Confirmation of Minor Enolized Isomer of Nominal Scabrolide B (1)

Comments:

The major product ("keto-form") of this sample is known from previous reports (eg MFA-MB-479 or MFA-MB-489), where it was fully assigned. The assignement within this report concentrates on the "first" minor product ("enol-form") of the sample. Since this is a minor product (~35%), a clean and complete assignment is not possible due to overlap and to weak signals. Furthermore, this compound was observed to be convert back to the major product over time and as observed from the 60% of its intensity after 1 day (see 1H comparison on page 4).

Two potential enol-structures are suggested, specifically at position C6 (Enol #1) or at position C3 (Enol #2). However, several observations indicate that the "first" minor product is the enol form #1:

-a "dd" pattern is observed for H11, indicating the absence of a 1H at the neighboring position C7 (the corresponding keto-form has a "ddd" pattern at H11).

-the enol carbon signal at 146.8 ppm is assigned to C6, with its long-range correlations to 1H assigned to positions C4 and C11; this is a large shift at position C6 compared to 202ppm (ketone) in the keto-form.

-a new signal assigned to a labile -OH appears at 7.92ppm which could belong to the alcoholic 1H at position 101.

-Different prediction methods (ACD, MNova) show that the 1H-shift and 13C-shift fit mostly to this enol. Still there are important discrepancies at several positions in the 6-ring. This is expected since these prediction methods do not take the configuration of the 8 chiral centers into consideration and are based on similarities with existing assignments. The webtool CSEARCH, which thoroughly verifies an assignment based on 13C-shifts, corroborates the uncertainties at several positions within the 6-ring. Still this tool rates the overall match of this assignment as "good".

Enol #2 can be ruled out. For one, a doublet would be expected for H12 instead of the observed dd. In addition, the long range correlation network does not match this structure. Using prediction tools, major deviations, namely at position C4 (13C shift of ~95-110 ppm and a 1H shift of ~5.5-6.0 ppm) would also reject this structure. It is noteworthy that a third compound in the sample with a even a smaller ratio than the first minor product is present as seen in the 13C and 1H.

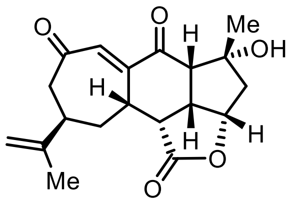

Major product
"keto"- form

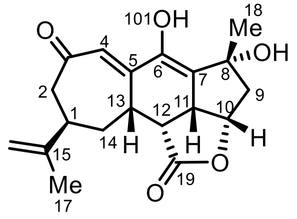

possible suggested Enol #1:
mostly supported

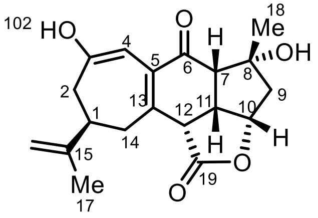

possible suggested Enol #2:
not supported

The compared spectra show the difference of the amount of the minor compound. It shows that the minor compound is disappearing. The spectra a scaled 1:1.
The spectrum above was the first proton measured and the spectrum below shows the spectrum one day later. Both were measured under the sam aquisition parameters and the same cirumstances.

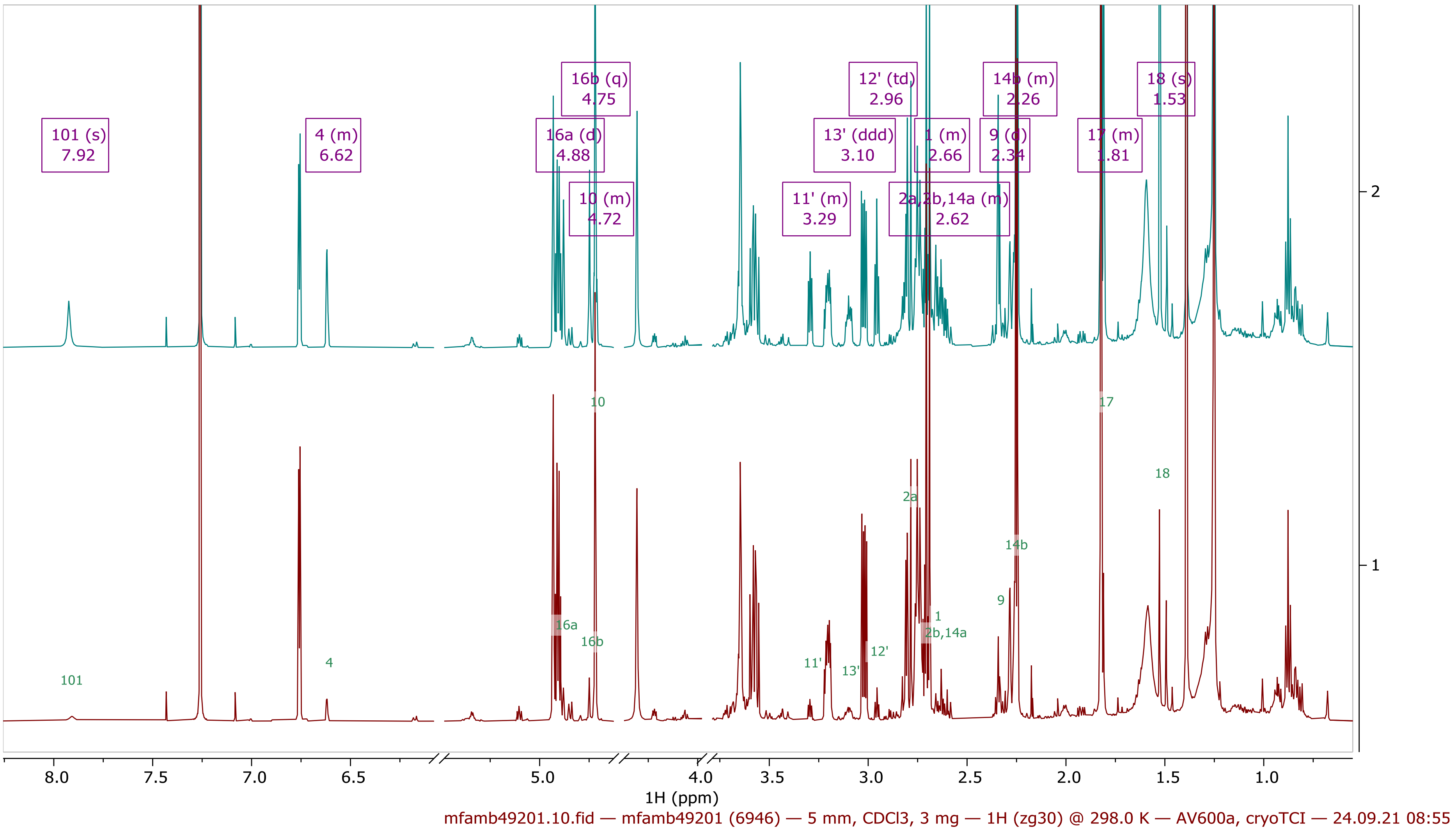

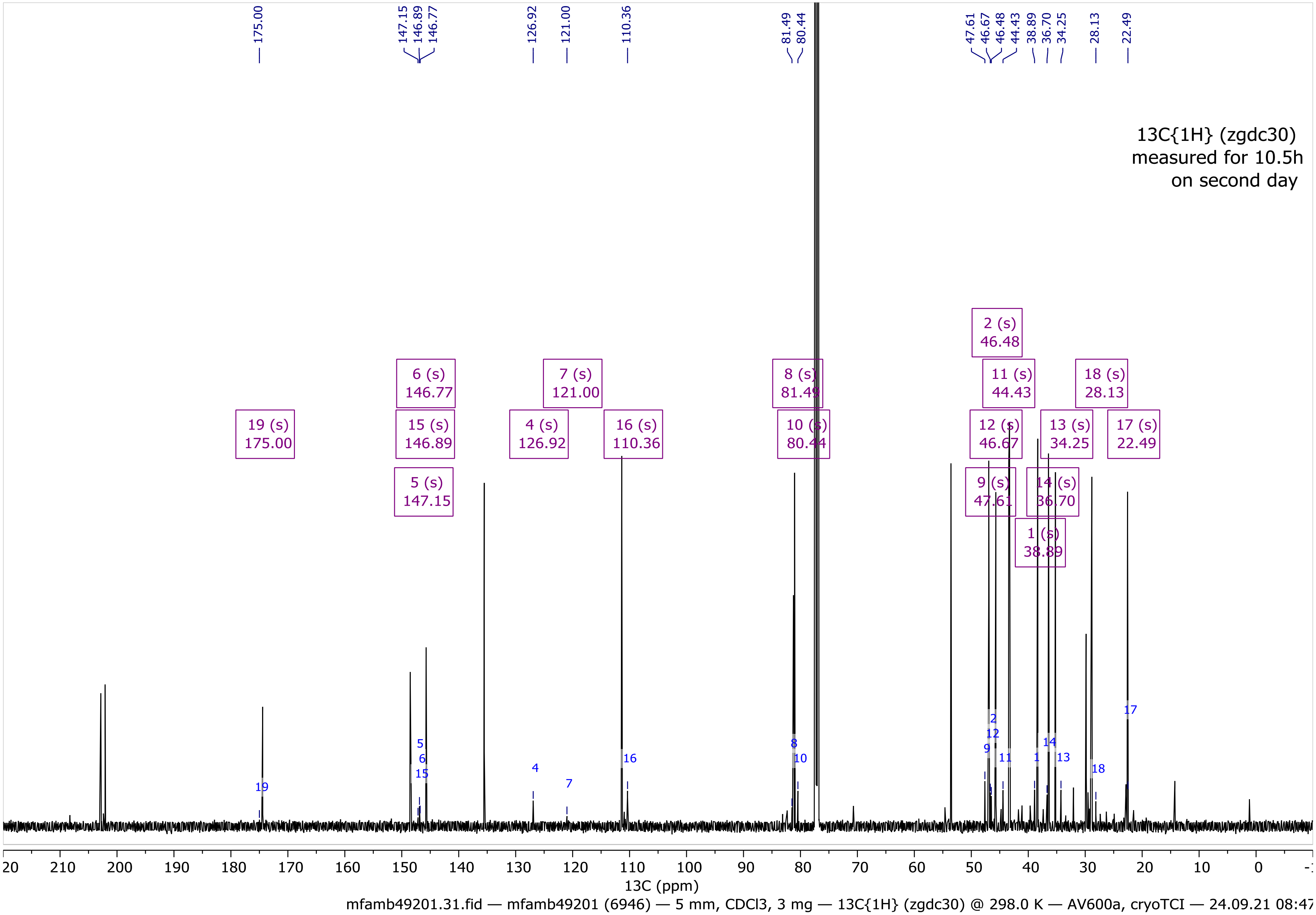
175.00
147.15
146.89
146.77
126.92
121.00
110.36
81.49
80.44
47.61
46.67
46.48
44.43
38.89
36.70
34.25
28.13
22.49
13C{1H} (zgdc30)
measured for 10.5h
on second day
19 (s) 175.00
6 (s) 146.77
15 (s) 146.89
5 (s) 147.15
7 (s) 121.00
4 (s) 126.92
16 (s) 110.36
8 (s) 81.49
10 (s) 80.44
2 (s) 46.48
11 (s) 44.43
12 (s) 46.67
9 (s) 47.61
18 (s) 28.13
13 (s) 34.25
14 (s) 36.70
1 (s) 38.89
17 (s) 22.49
13C (ppm)
mfamb49201.31.fid — mfamb49201 (6946) — 5 mm, CDCl3, 3 mg — 13C{1H} (zgdc30) @ 298.0 K — AV600a, cryoTCI — 24.09.21 08:47

COSY Spectrum

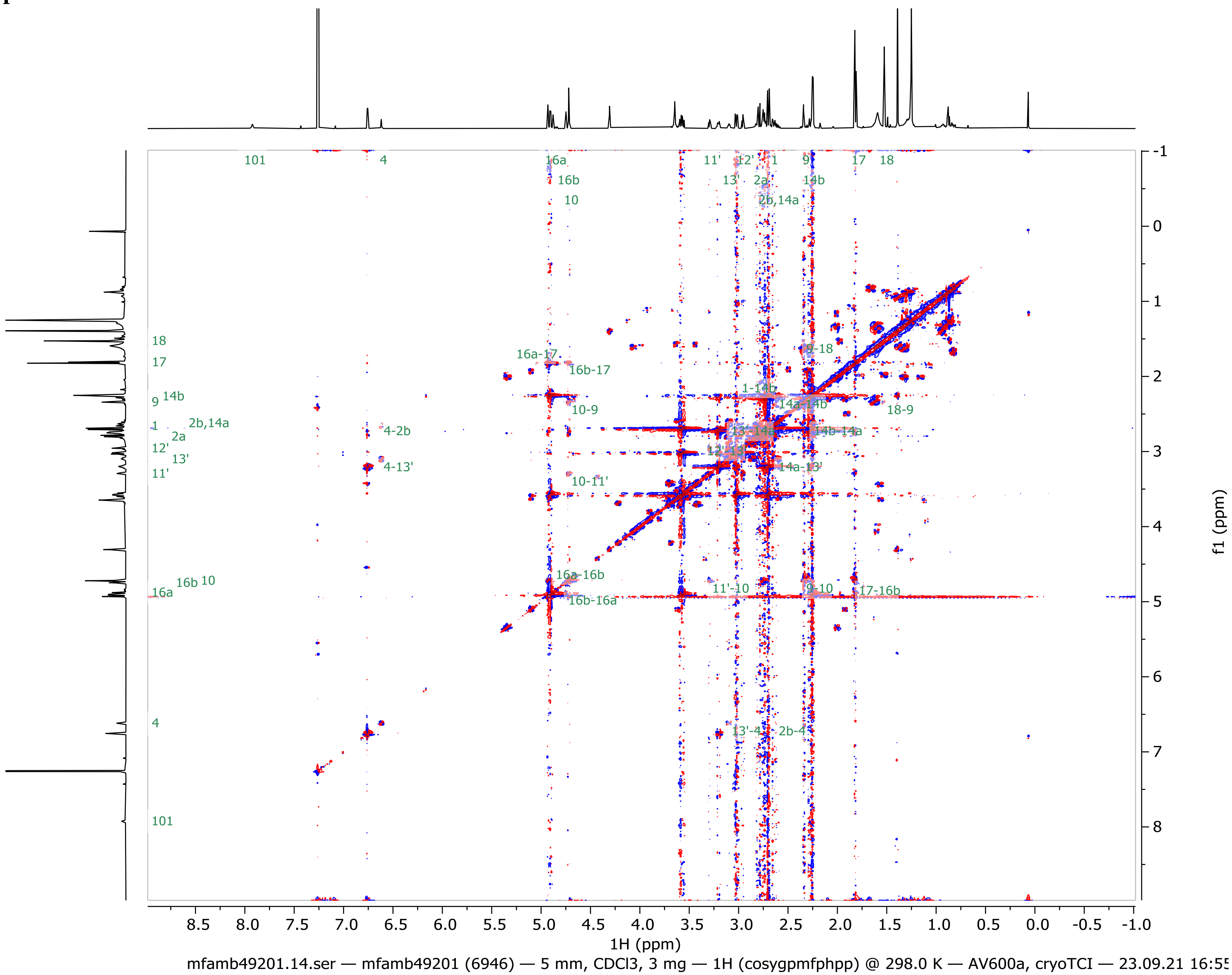

HSQC Spectrum

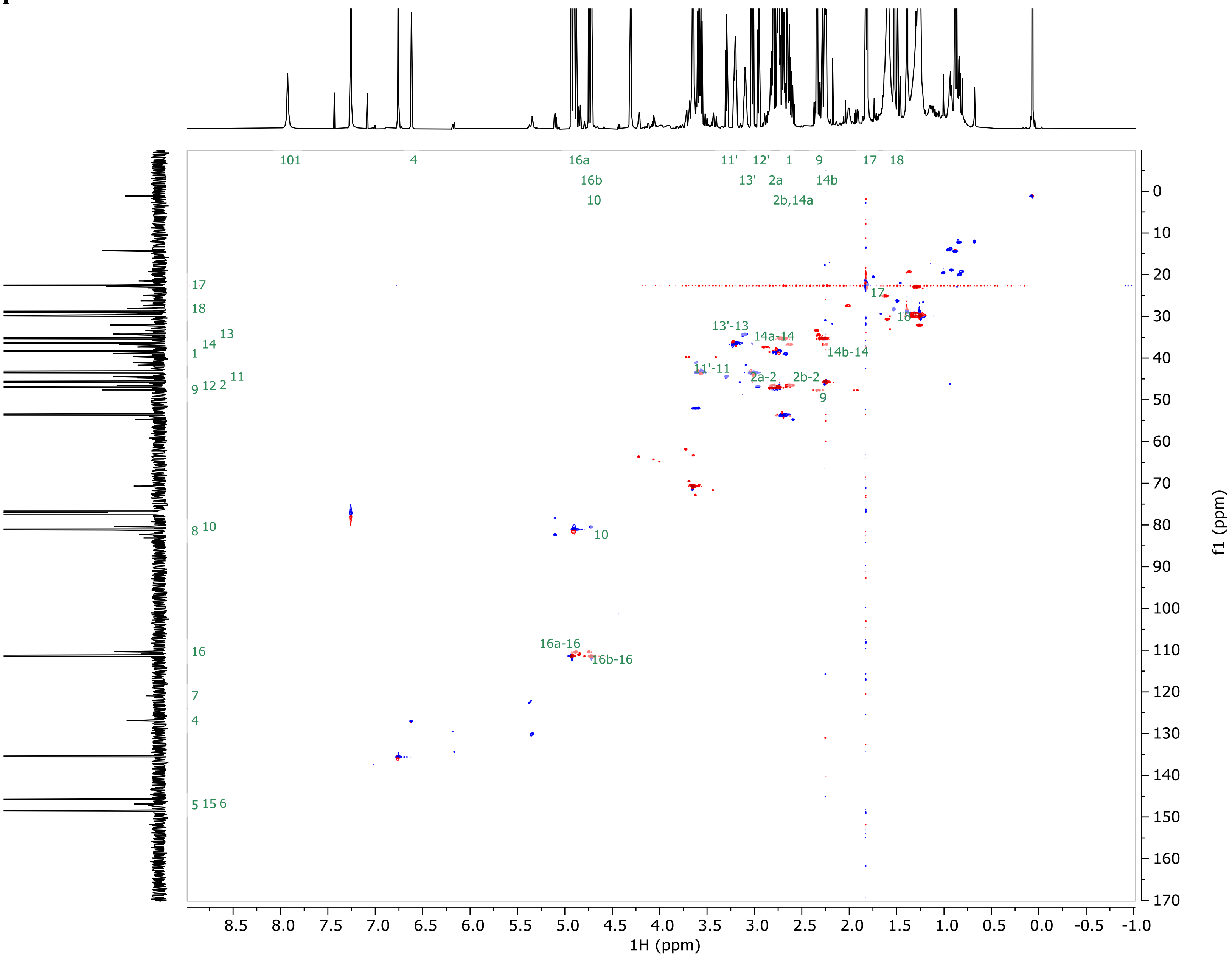

mfamb49201.12.ser — mfamb48601 (6880) — 5 mm, CDCl3, 2 mg — 1H-13C (hsqcedetgpsisp2.4) @ 298.0 K — AV600a, cryoTCI — 10.09.21 16:1[illegible]

HMBC Spectrum

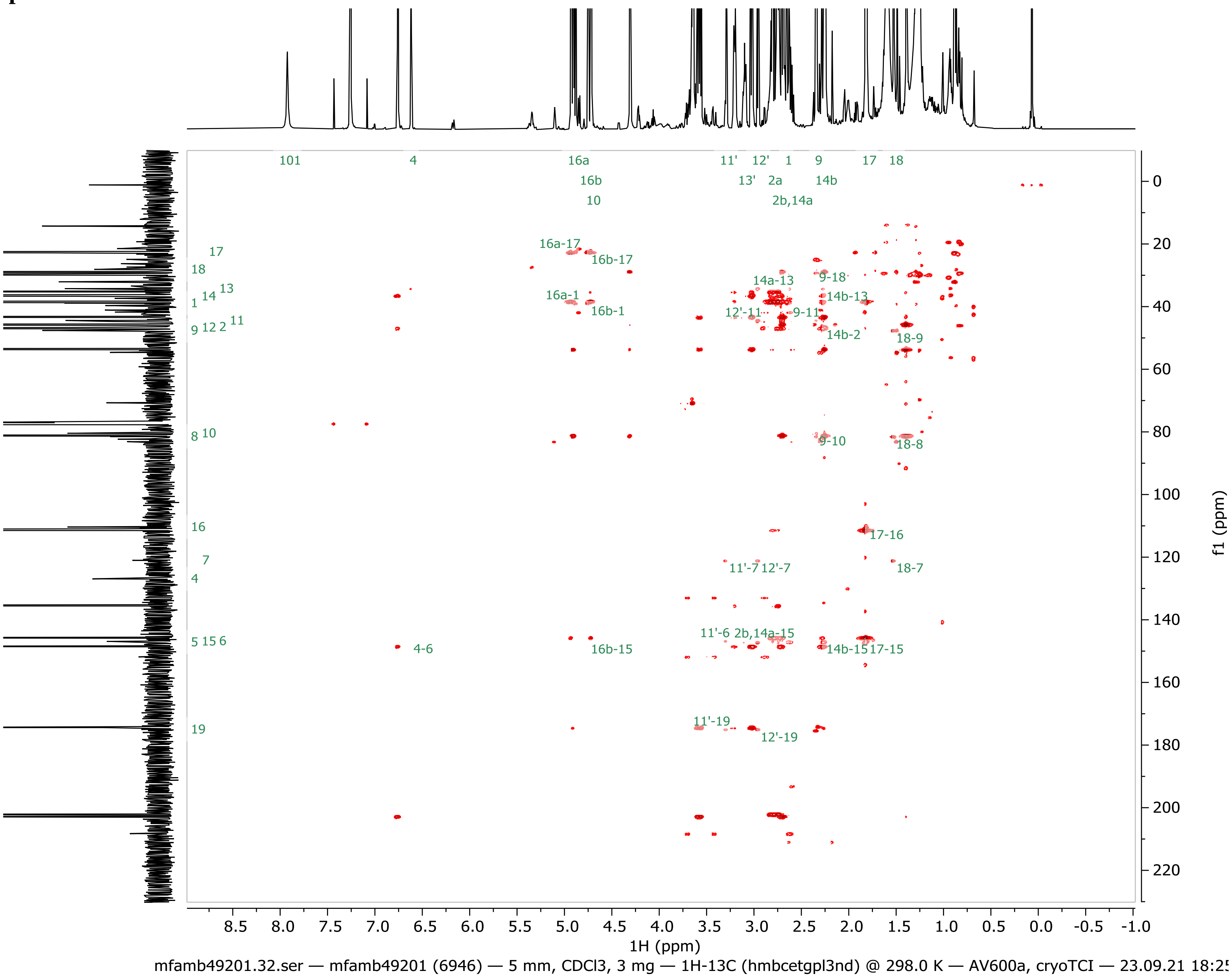

NOESY Spectrum

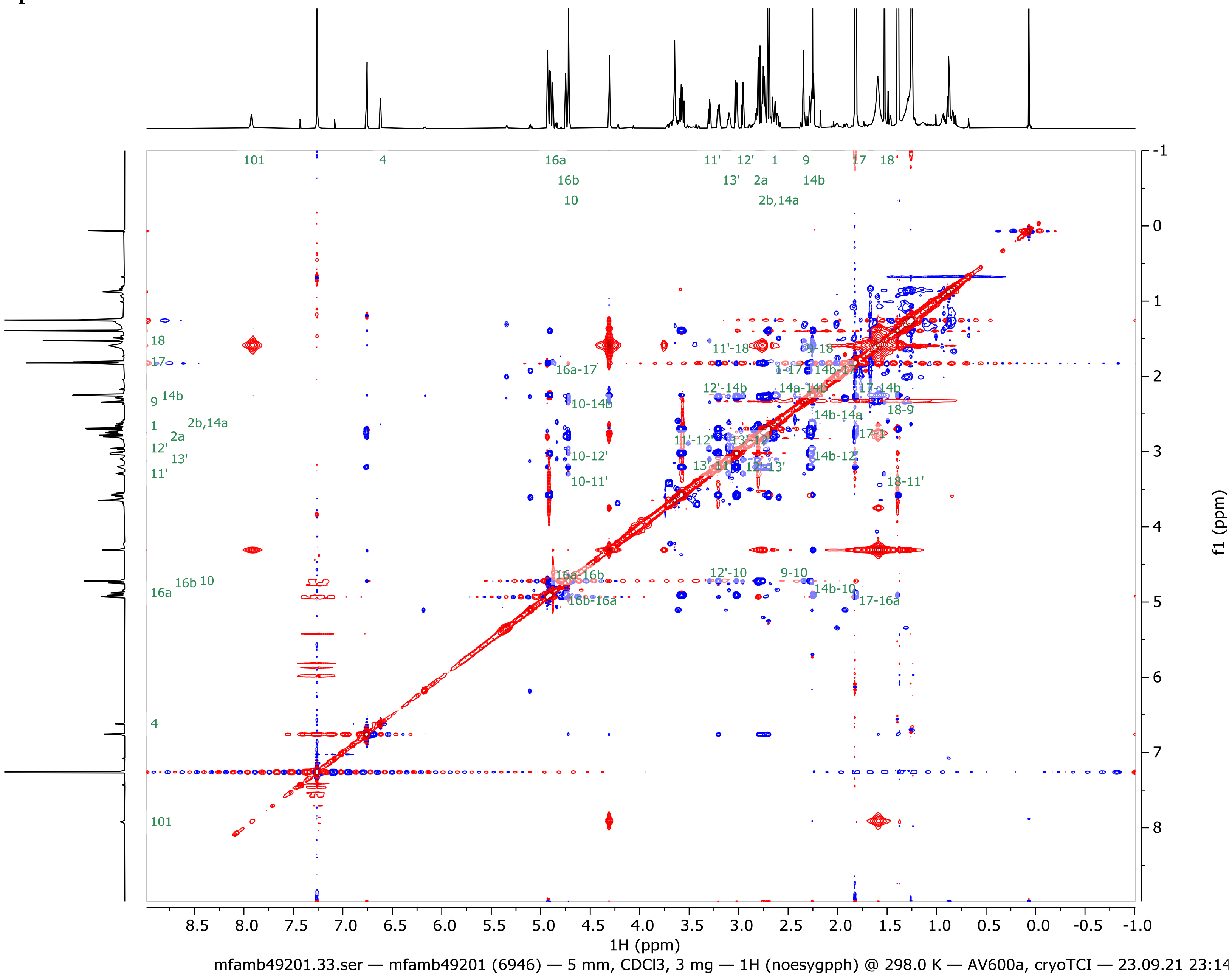

HSQC-TOCSY Spectrum

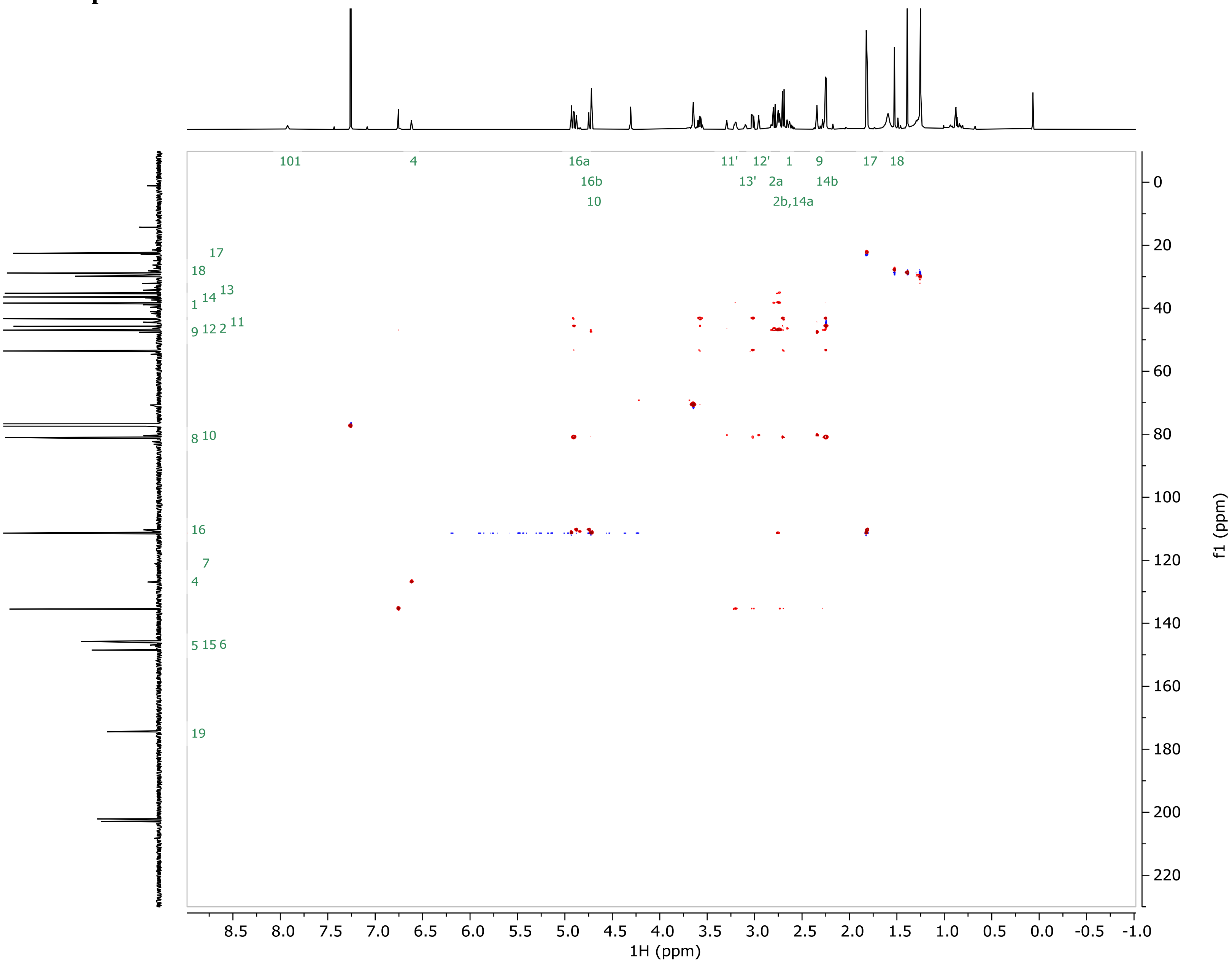

mfamb49201.17.ser — mfamb49201 (6946) — 5 mm, CDCl3, 3 mg — 1H-13C (hsqcetgpml) @ 297.9 K — AV600a, cryoTCI — 23.09.21 14:52

^{1}H NMR Spectrum of 34 (600 MHz, $CDCl_3$)

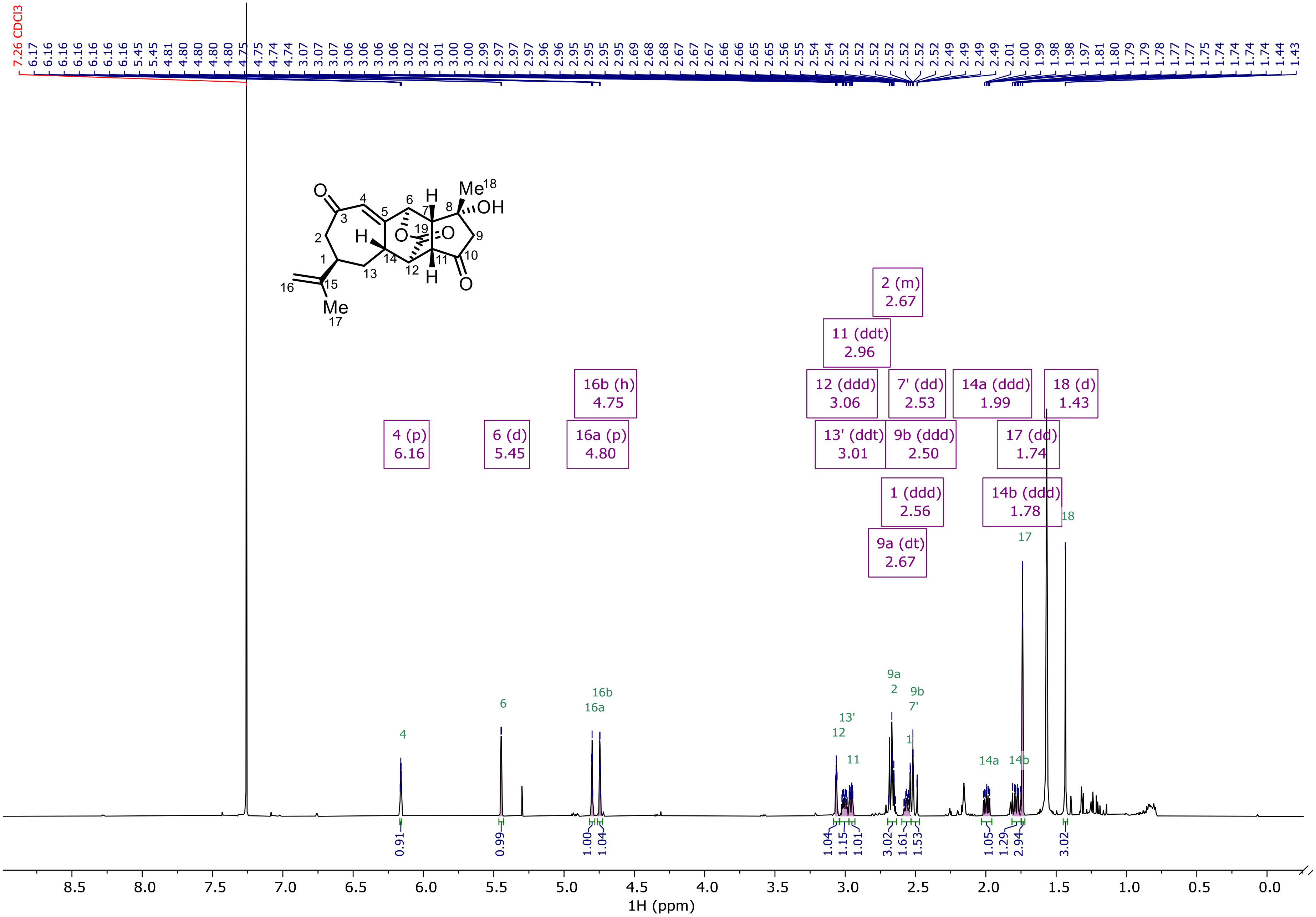

^{13}C NMR Spectrum of 34 (151 MHz, CDCl$_3$)

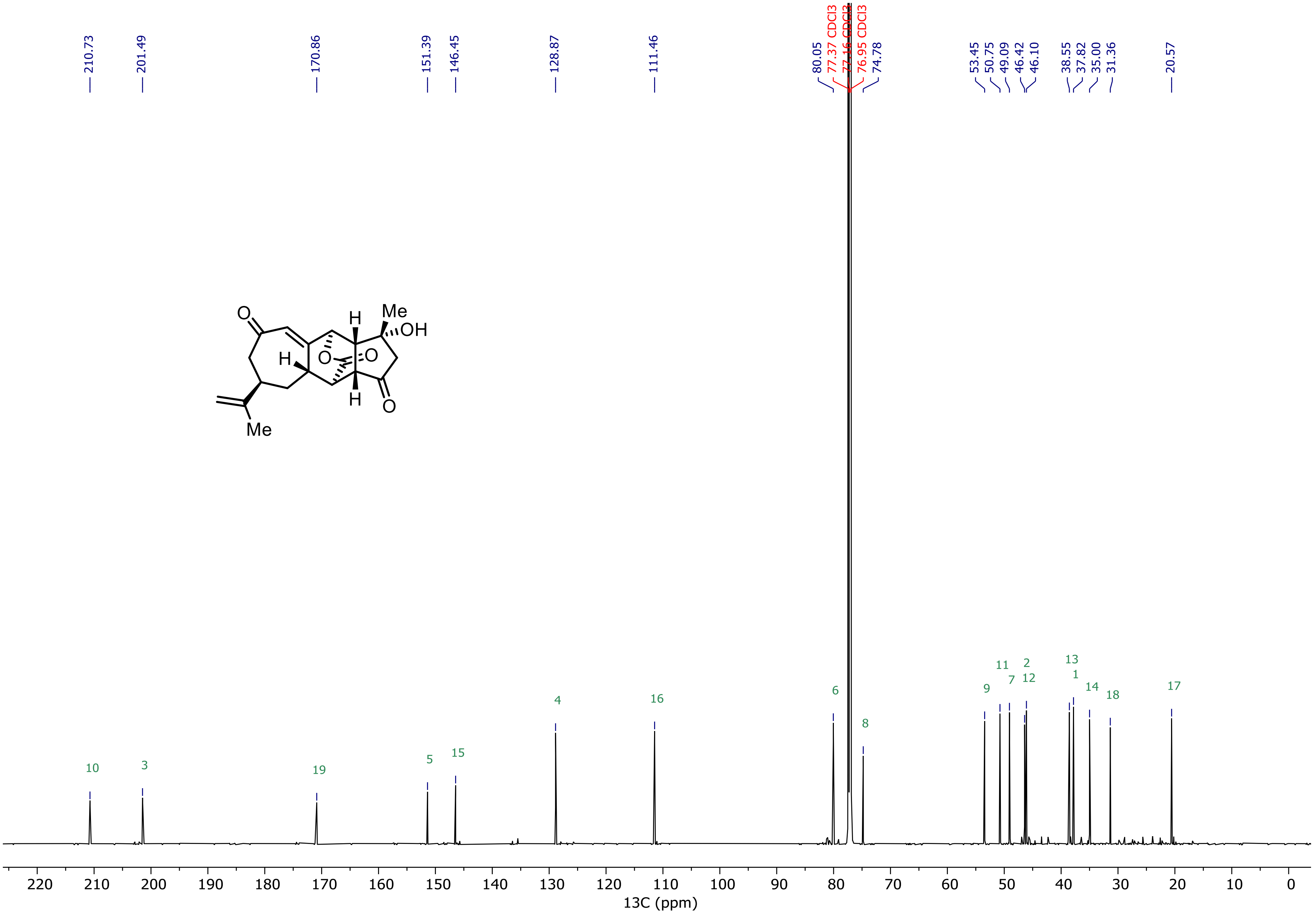

COSY Spectrum of 34

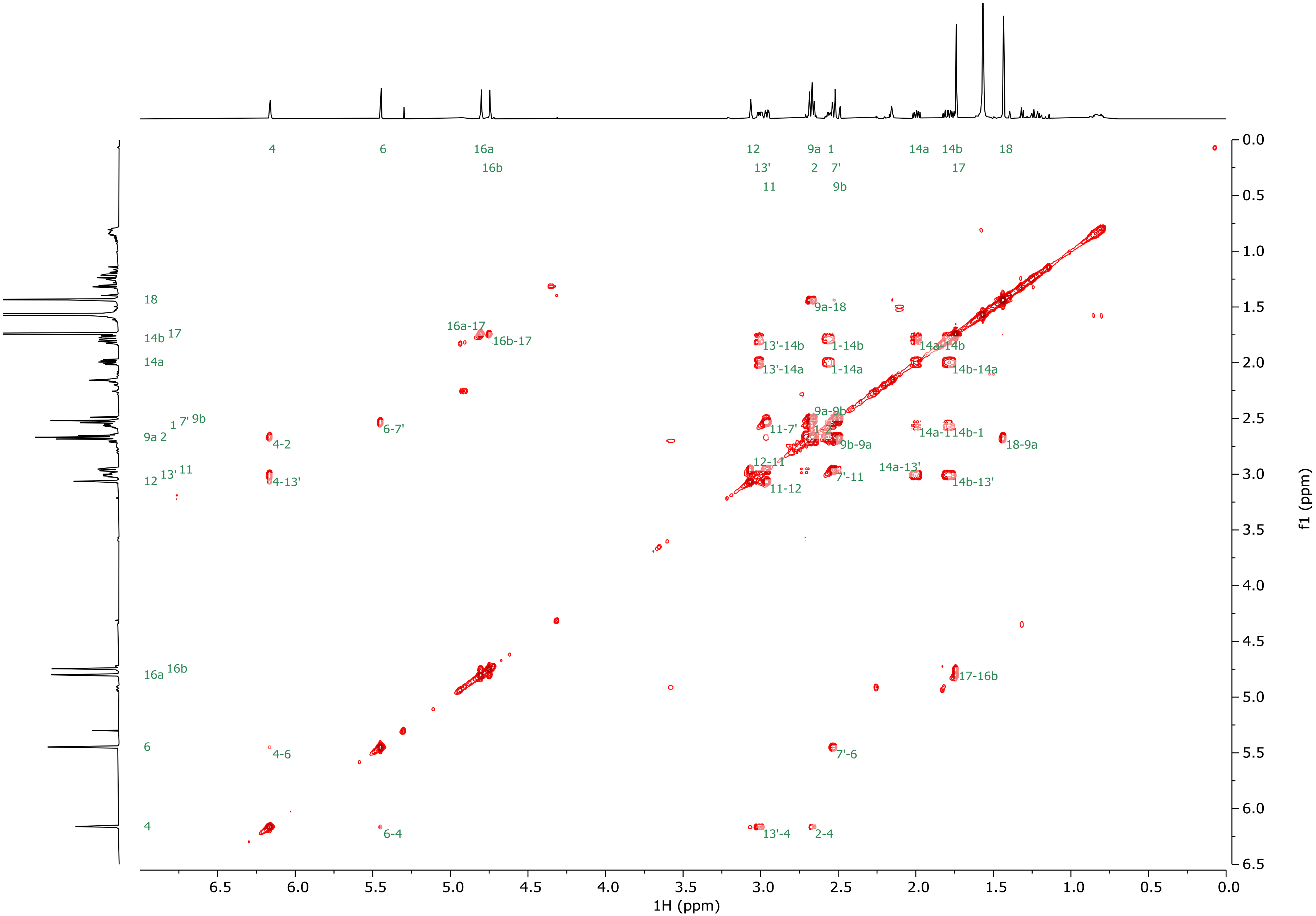

HMQC Spectrum of 34

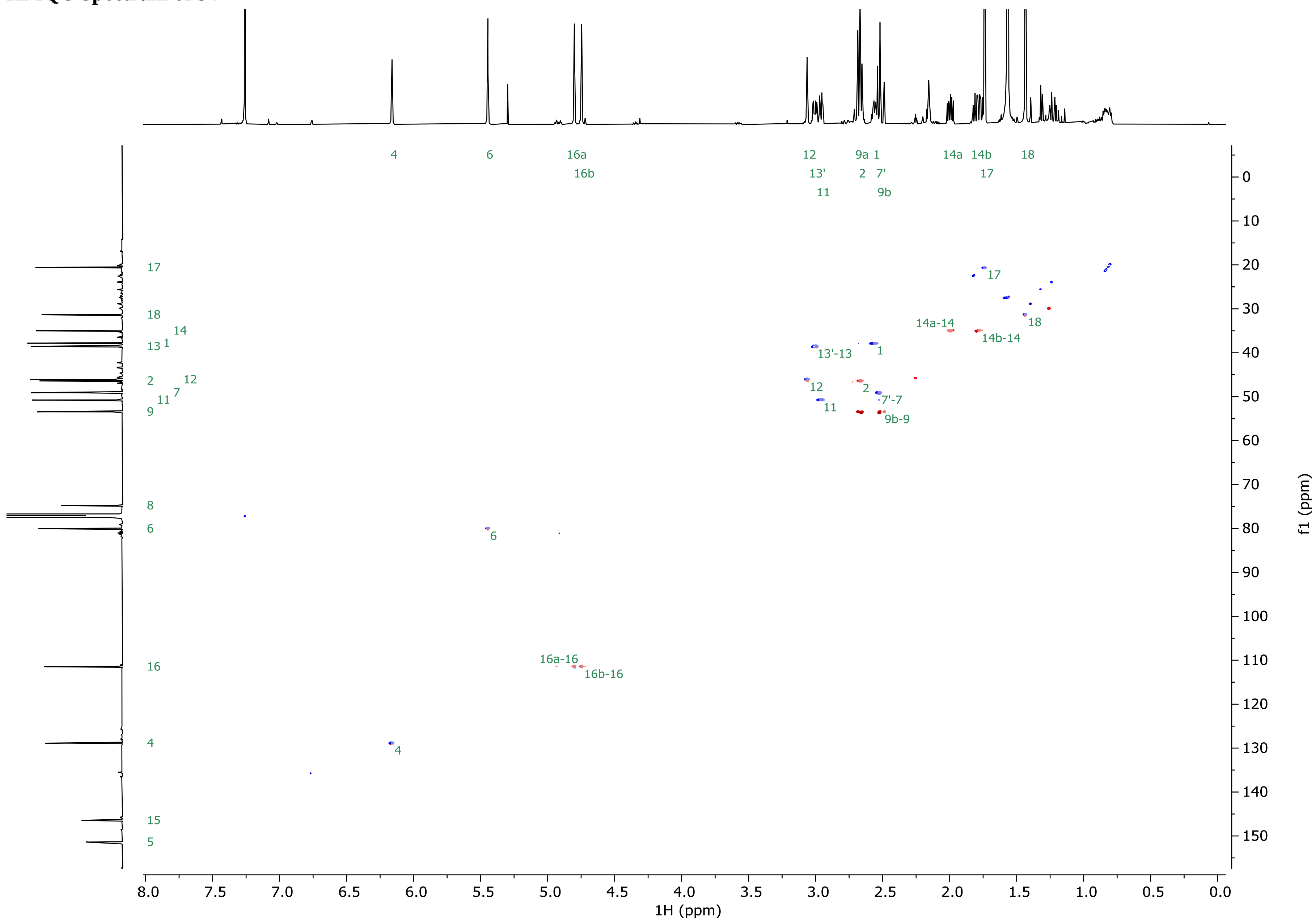

HMBC of Spectrum of 34

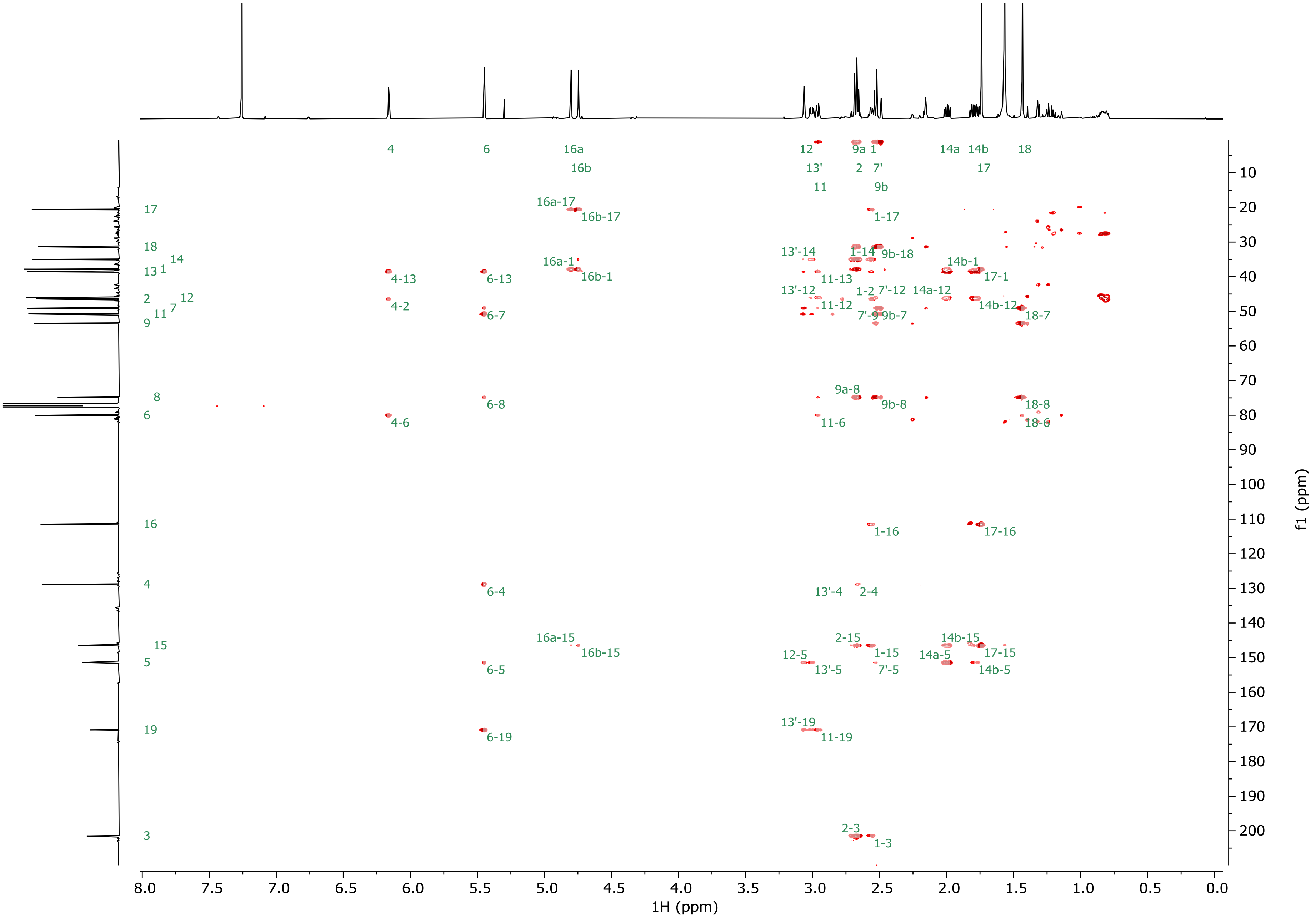

NOESY Spectrum of 34

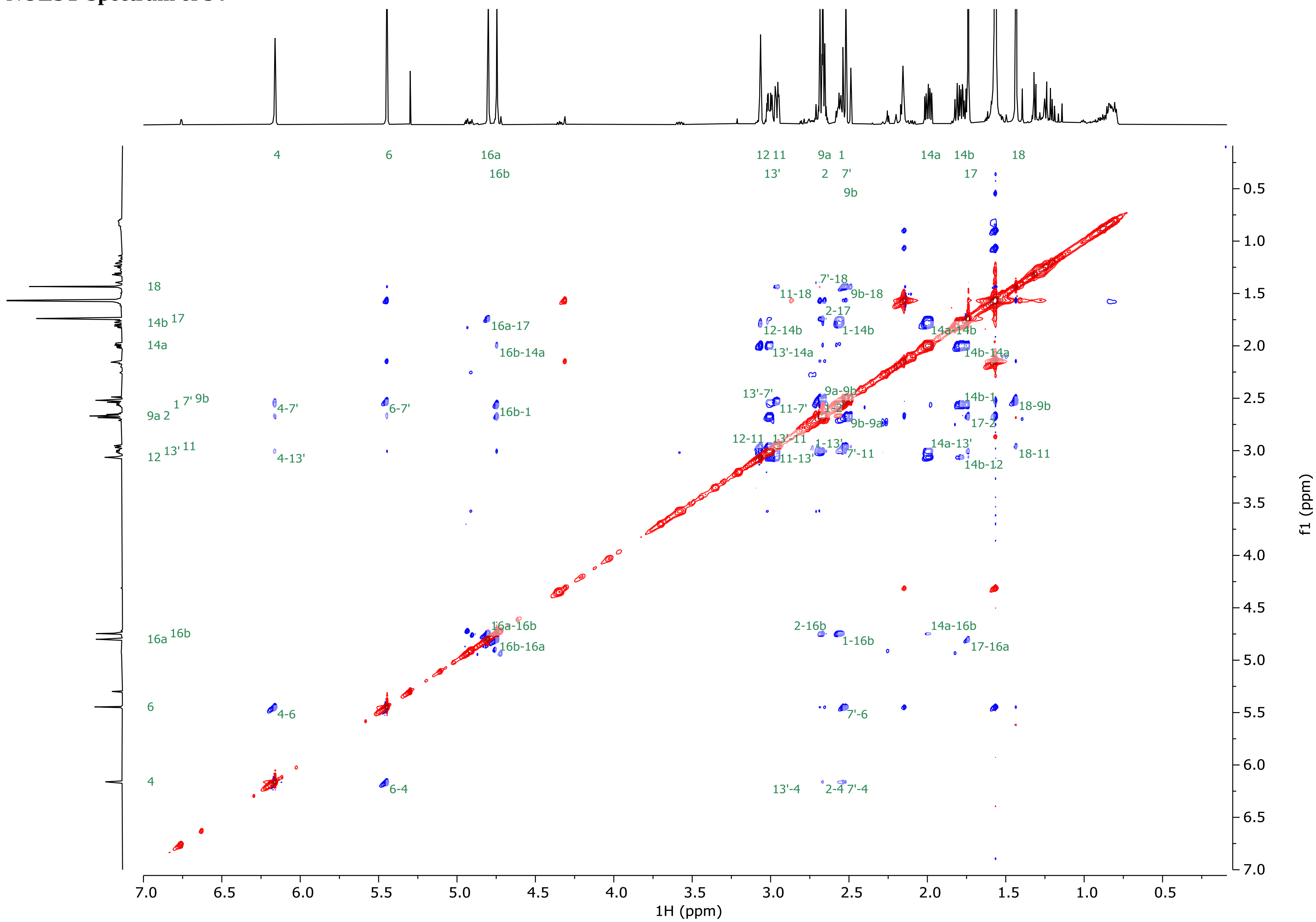

References

[1] Kim, S.; Kim, D.; Rajagopal, G. *Synthesis* **2004**, *2*, 213–216.

[2] Brocksom, T. J.; Brocksom, U.; Pergentino de Sousa, D.; Frederico, D. *Tetrahedron Asymm.* ***2005***, *16*, 3628–3632.

[3] Brill, Z. G.; Grover, H. K.; Maimone, T. J. *Science* **2016**, *352*, 1078−1082.

[4] Thach, D. Q.; Brill, Z. G.; Grover, H. K.; Esguerra, K. V.; Thompson, J. K.; Maimone, T. J. *Angew. Chem., Int. Ed*. **2020**, *59*, 1532−1536.

[5] Sheu, J.-H.; Ahmed, A. F.; Shiue, R.-T.; Dai, C.-F.; and Kuo, Y.-H. *J. Nat. Prod*. **2002**, *65*, 1904–1908.
